# A genomic view of the microbiome of coral reef demosponges

**DOI:** 10.1038/s41396-020-00876-9

**Published:** 2021-01-19

**Authors:** S. J. Robbins, W. Song, J. P. Engelberts, B. Glasl, B. M. Slaby, J. Boyd, E. Marangon, E. S. Botté, P. Laffy, T. Thomas, N. S. Webster

**Affiliations:** 1 Australian Centre for Ecogenomics, University of Queensland, Brisbane, QLD 4072 Australia; 2 Centre for Marine Science & Innovation, University of New South Wales, Kensington, NSW 2052 Australia; 3 Australian Institute of Marine Science, Townsville, QLD 4810 Australia; 4 GEOMAR Helmholtz Centre for Ocean Research Kiel, Düsternbrooker Weg 20, 24105 Kiel, Germany; 5 College of Science and Engineering, James Cook University, Townsville, QLD 4810 Australia

**Keywords:** Microbial ecology, Metagenomics, Symbiosis

## Abstract

Sponges underpin the productivity of coral reefs, yet few of their microbial symbionts have been functionally characterised. Here we present an analysis of ~1200 metagenome-assembled genomes (MAGs) spanning seven sponge species and 25 microbial phyla. Compared to MAGs derived from reef seawater, sponge-associated MAGs were enriched in glycosyl hydrolases targeting components of sponge tissue, coral mucus and macroalgae, revealing a critical role for sponge symbionts in cycling reef organic matter. Further, visualisation of the distribution of these genes amongst symbiont taxa uncovered functional guilds for reef organic matter degradation. Genes for the utilisation of sialic acids and glycosaminoglycans present in sponge tissue were found in specific microbial lineages that also encoded genes for attachment to sponge-derived fibronectins and cadherins, suggesting these lineages can utilise specific structural elements of sponge tissue. Further, genes encoding CRISPR and restriction-modification systems used in defence against mobile genetic elements were enriched in sponge symbionts, along with eukaryote-like gene motifs thought to be involved in maintaining host association. Finally, we provide evidence that many of these sponge-enriched genes are laterally transferred between microbial taxa, suggesting they confer a selective advantage within the sponge niche and therefore play a critical role in host ecology and evolution.

## Introduction

Coral reefs are among the most productive ecosystems in the world and are frequently referred to as ‘rainforests of the sea’ due to their immense biodiversity [1]. However, despite their exceptionally high primary productivity, nutrient levels on tropical reefs are typically low, necessitating efficient mechanisms for nutrient retention. Marine sponges are therefore an essential component of reef ecosystems because they filter large volumes of seawater (up to thousands of litres per day [2]) from which they retain the organic matter and transform it into biomass that can be consumed by detritivores, ultimately recycling it back into the reef system in a process known as the “sponge-loop” [3]. Reef-dwelling sponges harbour stable and diverse microbial communities that can account for up to 35% of sponge biomass and are hypothesised to carry out functions that support their host’s health and ecology, such as the transformation of carbon (e.g. polysaccharides) [4–6], nitrogen (e.g. archaeal ammonia oxidation) [7] and sulfur [8], as well as providing essential vitamins and amino acids to the host [9]. Sponges also play host to a diverse array of mobile genetic elements (MGE), such as viruses [10, 11], that may necessitate a diverse array of defence mechanisms like clustered regularly interspaced short palindromic repeat (CRISPR) and restriction-modification (RM) systems. The presence of such mobile elements raises the possibility of lateral gene transfer (LGT) between symbionts, though evidence for this is currently lacking.

Despite their importance for host health, few community-level functional investigations have been undertaken to capture the broad range of microbial taxa found in sponges [8, 12]. Instead, gene-centric studies have identified a number of interesting sponge symbiont traits but have been unable to link these features to specific microbial taxa. In addition, nearly all genome-centric characterisations of sponge-associated microbes have been restricted to a few lineages of interest [13–15] or have focussed on low-abundance microorganisms amenable to cultivation [16], with the majority of lineages remaining undescribed. This skew likely biases our understanding of the roles that each symbiont lineage plays within the microbiome and hinders our ability to identify features that underpin sponge-microbe symbiosis. To address this, we undertook an integrated analysis of 1188 metagenome-assembled genomes (MAGs) derived from seven marine demosponge species, spanning 25 microbial phyla and the vast majority of microbial taxa commonly found in marine sponges.

## Materials and methods

### Sample collection and enrichment of bacteria from sponges

This study comprised seven host species belonging to the class Demospongiae (hereafter referred to as sponges for simplicity), representing the sub-classes Heteroscleromorpha (*Cliona orientalis* and *Stylissa flabelliformis*), Verongimorpha (*Aplysina aerophoba*) and Dictyoceratida (*Rhopaloeides odorabile, Coscinoderma matthewsi, Ircinia ramosa* and *Carteriospongia foliascens*). Two individuals of *R. odorabile* were collected from Esk Island (18° 45.830′S; 146° 31.159′E) and one from Falcon Island (18° 46.116′S; 146° 32.201′E) on the 25th of October, 2018. Four individuals of *C. matthewsi*, *C. foliascens*, *S. flabelliformis*, *I. ramosa* and *C. orientalis* were collected from Davies Reef (18° 49.948′S; 147° 37.995′E) between the 22nd and the 23rd of December, 2015. All sponges were rinsed in filter-sterilised seawater before being snap-frozen in liquid nitrogen and stored at −80 °C. Cell fractionation was performed according to methods described in Botte et al. [17]. Briefly, sponges were rinsed twice in calcium-magnesium free seawater before being cut into 1 cm^3^ pieces and homogenised. Due to its bioeroding lifestyle, *C. orientalis* was crushed in liquid nitrogen with a mortar and pestle to separate sponge tissue from the coral skeleton. Sterile collagenase (Sigma-Aldrich) was added at a concentration of 0.5 g L^−1^ and samples were shaken at 150 rpm for 30 min. Samples were filtered through 100 μm sterile cell strainers and centrifuged at 100 × *g* for 1 min before recovering the supernatant and centrifuging at 300 × *g* for 15 min. This last step was repeated and the resulting supernatant was filtered sequentially through 8 and 5 μm filters. Microbial pellets were recovered by centrifuging at 8000 × g for 20 min, resuspended in Tris-HCl NaCl at pH 8.0 and kept at −20 °C. To minimize carry over of eukaryotic DNA from the sponge, the microbial cell pellet was treated with DNase (DNase I; New England Biolabs) prior to cell lysis for DNA extraction. In addition, 10 L of inshore coastal seawater was collected from the Sea Simulator (SeaSim) at the Australian Institute for Marine Science in October 2016 for metagenomic sequencing. Seawater was filtered first through a 5 μm prefilter and then collected onto a 0.2μm sterivex filter.

### Metagenomic sequencing

Sponge samples were extracted using the Qiagen MagAttract PowerSoil DNA kit as described by Marotz et al. [18] or the DNeasy PowerBiofilm kit (QIAGEN) for Rhopaloeides. Metagenomic DNA from *R. odorabile* was sequenced at the Ramaciotti Centre for Genomics (University of New South Wales, Sydney, Australia), while other sponge species were sequenced at the University of California San Diego as part of the Earth Microbiome Project (EMP). Metagenomic sequencing was performed over several iterations as part of the EMP using the Kapa HyperPlus and Nextera XT library prep kits, with sequencing performed on Illumina HiSeq 4000 and NovaSeq 600 machines (2 × 151 bp). For R. odorabile, library prep was performed using the Nextera DNA Flex library prep kit and sequenced on a NextSeq 500 machine.

Seawater from the SeaSim was extracted by adding 100 mg/mL lysozyme directly to the sterivex cartridge and incubating for 1 h at 37 °C with gentle rotation, followed by the addition of 20 mg/mL proteinase K and incubation for 1 h at 55 °C. DNA clean-up was performed by adding an equal volume of Phenol:Chloroform:Isoamyl alcohol (25:24:1) to the lysate in a separate tube, spinning at 16,000 × *g* for 10 min, and recovering the aqueous layer, then repeating using Chloroform:Isoamyl alcohol (24:1). DNA was precipitated by adding 0.8 volumes of isopropanol, mixing gently, incubating for 15 min at room temperature, then spinning at 20,000 × *g* at 4 °C for 20–30 min to pellet DNA. Isopropanol was removed and 500 µl 70% ethanol was added to the pellet, then spun at 20,000 × *g* for 10 min and removed. The remaining pellet was allowed to dry for 30 min, then resuspended in 30 µl PCR grade water and stored at −80 °C until metagenomic sequencing at the Australian Centre for Ecogenomics. Sequencing was carried out using the Nextera Flex library prep kit on the NextSeq500 platform (2 × 150 bp).

### Metagenome assembly, binning and taxonomic assignment of bacterial and archaeal MAGs

Sponge-associated reads belonging to the same sample and sequenced across multiple runs were concatenated into a single set of forward and reverse fastq files. Both sponge and SeaSim seawater samples were processed as follows: Adapter clipping of the reads was performed using seqpurge v0.1-852-g5a7f2d2 [19] and read sets from each sample were assembled using metaSPAdes v3.9.0 [20]. Reads from each sponge species, or seawater source, were mapped in an all-versus-all manner using BamM v1.7.3 (https://github.com/Ecogenomics/BamM), a wrapper script leveraging the BWA mapping algorithm [21], to obtain BAM files for differential coverage estimation. Binning was performed using uniteM v0.0.16 (https://github.com/dparks1134/UniteM). In brief, UniteM uses the binning algorithms maxbin v2 v2.2.4, metabat v1 v0.32.4 using all parameter sets (e.g. very sensitive, etc.), metabat v2 v2.12.1, and groopM2 v2 v2.0.0-1, and selects the best MAGs by their CheckM [22] quality scores. For *A. aerophoba*, six previously published [12] datasets (PRJNA366444-PRJNA366449 and PRJNA326328) were co-assembled using Megahit v1.1.3 [23] and binning was performed with metaWRAP v1.0.1, which uses metabat, metabat v2 and maxbin v2. Taxonomy was assigned to each MAG based on the Genome Taxonomy Database (GTDB) [24] (http://gtdb.ecogenomic.org) using GTDB-Tk [25], which classifies MAGs based on placement in a reference tree inferred using a set of 120 bacterial and 122 archaeal concatenated gene markers using a combination of FASTANI and pplacer [26, 27]. The relative abundance of each MAG from separate individuals was calculated with coverM v0.2.0 (https://github.com/wwood/CoverM), after de-replicating all MAGs at 95% identity with dRep v2.2.4 [28] to avoid arbitrary mapping between representatives of highly similar genomes. MAGs with an overall abundance >5% in at least one sample were included in the heat map visualised with R v3.5.1 [29] (Fig. S1). Additional MAGs from coral reef seawater were obtained from other studies [30, 31]. Information regarding the MAGs (taxonomy, MIMAG standard statistics, etc) presented in this study can be found in Table S1.

### Identification and analysis of CuMMO family genes

GraftM [32] was used to recover copper-dependent membrane-bound monooxygenase (CuMMO) family genes (GraftM package ID: 7.22) from the sponge-associated MAGs and a published metagenomic dataset from sponges where bacterial *amo* genes had previously been reported [33]. Briefly, GraftM uses gene-specific (e.g. ammonia monooxygenases (*amoA*)) hidden Markov models (HMMs) to identify and extract sequences from metagenomic reads or assemblies and inserts them into a reference tree to assign them to pre-defined functional clades. A phylogenetic tree was then inferred from these sequences with IQ-TREE v1.6.12 [34], including previously published CuMMO sequences [35, 36] after alignment with MAFFT v7.221 [37]. The tree was rooted between the domains Archaea and Bacteria, grouped into functional clades based on the grouping in Alves et al. (Fig. S2) [35], and refined in iTOL v4.2.3 [38].

### Gene annotation and statistics

To ensure that the observed presence/absence of genes was not influenced by MAG completeness, only a set of highly complete MAGs (>85% completeness) were annotated with the “annotate” function of EnrichM v0.2.1 (https://github.com/geronimp/enrichM) using the Pfam database to identify eukaryote-like repeat (ELR) proteins and the Kyoto Encyclopaedia of Genes and Genomes (KEGG) Orthologies (KOs) to reconstruct metabolic pathways [39, 40], and the HMMs from dbCAN [41] to identify carbohydrate-active enzymes (CAZY) like glycosyl hydrolases (GHs) and carbohydrate esterases [42] (Files. S1–S3). EnrichM’s “annotate” function was also used to identify orthologous clusters between highly complete MAGs (Fig. S3 and File. S4). For amino acid synthesis pathways, EnrichM’s “classify” function was used to calculate the completeness of KEGG modules, which are groups of genes organised by steps in a metabolic pathway as defined by KEGG. To identify genes and pathways enriched in sponge-associated MAGs (which may therefore may represent functions important for sponge-microbe symbiosis), EnrichM’s “enrichment” function (https://github.com/geronimp/enrichM) was used to perform statistical tests for the enrichment of KEGG modules, CAZY genes and Pfams between highly complete sponge and seawater-associated MAGs (Tables S2, S3 and S4). Enrichment of KOs within each module was calculated individually using a two-sided Mann–Whitney *U*-test and only modules for which >70% of KOs were enriched were further considered. For Pfam and CAZY enrichment calculations, EnrichM directly compares the number of proteins per MAG matching that Pfam using a two-sided *t*-test. For both KEGG and Pfam comparisons, a Benjamini–Hochberg correction was applied to control for the false discovery for multiple comparisons. The ggplot package was used through EnrichM to make PCA plots based on the gene content of each MAG (KEGG, Pfam, orthologous clusters of genes).

### Tree building and visualisation

**Fig. 1:**
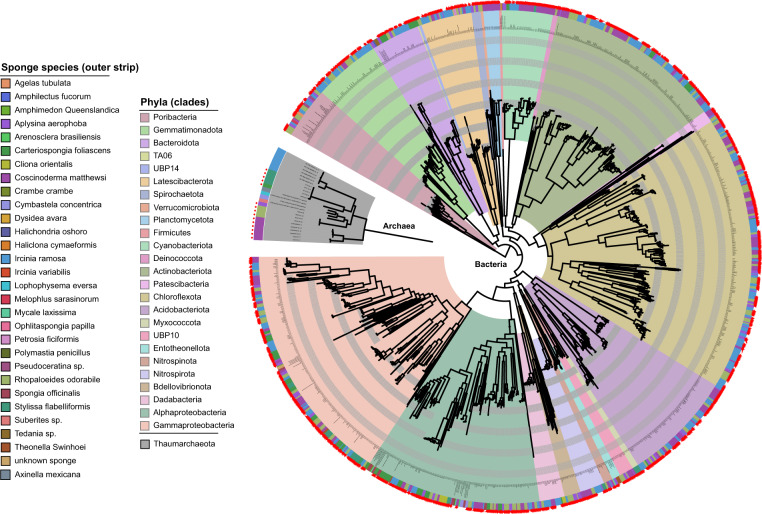
Phylogenetic tree showing all publicly available bacterial and archaeal MAGs and genomes (*N* = 1253 genomes) with >50% completeness and <10% contamination, recovered from 30 sponge species (Table S1). Inner clade colour denotes phylum affiliation except for the Proteobacteria, which is split by class into alpha and gamma-proteobacteria. Outer tree colour strip identifies the sponge species from with the genome or MAG originated. Red stars indicate MAGs produced in this study.

To visualise the phylogenetic distribution of the recovered MAGs, phylogenetic trees were constructed by supplying the bacterial and archaeal concatenated marker gene alignments produced by GTDB-Tk to IQ-TREE using the LG + G model [34]. Bacterial and archaeal trees were initially constructed from all sponge-associated MAGs with >50% completeness and <10% contamination, including those from previous studies (Fig. 1 and Table S1). To determine how much additional phylogenetic diversity the MAGs from this study added to the trees, phylogenetic distance, defined as the “total branch length spanned by a set of taxa” and phylogenetic gain, defined as the “additional branch length contributed by a set of taxa” [24], were calculated for both the bacterial and archaeal tree using GenomeTreeTk v0.0.41 (https://github.com/dparks1134/GenomeTreeTk). A second tree constructed from all MAGs with >85% completeness, including those from seawater, was used as a basis for visualising the distribution of genes-of-interest (e.g. GHs, ELR, etc) across lineages using iTOL [38] (Files. S5 and S6).

### Identification of lateral gene transfers

To identify laterally transferred genes within the sponge-associated microbial communities, MAGs associated with the same sponge species, as well as all seawater-derived MAGs, were first dereplicated with dRep v2.3.2 [28] at 99% average nucleotide identity. LGT analysis was performed using MetaCHIP v1.7.5 [43] on all dereplicated MAGs at phylum, class, order, family and genus levels. In brief, MetaCHIP first clusters query genomes according to their phylogenies, then performs an all-against-all BLASTN for all predicted genes. The BLASTN matches for each gene are then compared among taxa and a gene is considered to be a putative LGT if the best-match comes from a non-self taxa. A phylogenetic approach is then applied to putative LGTs for further validation using Ranger-DTL [44] and the direction of gene flow is identified. False-positive LGTs, as could be introduced through MAG contamination, were filtered out by removing LGTs marked by MetaCHIP as “full-length match” (LGT makes up large proportion of contig) or “end match” (LGT falls at end of contig). For the current dataset, LGTs on average made up only 12 ± 14% of the total length of their respective contigs, ensuring that binning was based on compositional information (e.g. k-mers as used by MetaBat) from the non-LGT portion of the contig. LGTs detected at all taxonomic levels were combined and dereplicated. The genetic divergence of the dereplicated LGTs, as well as the frequency of LGT transfer (the number of LGTs per Mbp sequence data) per MAG, were summarised on the basis of taxon or host of MAGs and visualised using the Circlize package in R.

## Results and discussion

Six sponge species, *R. odorabile*, *C. matthewsi*, *C. foliascens*, *S. flabelliformis*, *I. ramosa* and *C. orientalis* (a bioeroding sponge), were selected for metagenomic sequencing (7 ± 0.5 Gbp) as these species represent dominant habitat forming taxa on tropical and temperate Australian reefs and exhibit high intraspecies similarity in their microbiomes. In addition, previously published microbial MAGs from *I. ramosa* and *Aplysina aerophoba* were analysed [8, 12], including 62 additional unpublished MAGs from *A. aerophoba*. The recovered MAGs, averaging 86 ± 12% completeness and 2 ± 2% contamination, made up 72 ± 21% relative abundance of their respective communities (by read mapping) on average and spanned the vast majority of microbial lineages typically seen in marine sponges [45] (Fig. S1 and Table S1), including the bacterial phyla Proteobacteria (331 MAGs), Chloroflexota (242), Actinobacteriota (155), Acidobacteriota (97), Gemmatimonadota (60), Latescibacterota (44; including lineages Anck6, PAUC34 and SAUL), Cyanobacteria (43), Bacteroidota (38), Poribacteria (35), Dadabacteria (22; including SBR1093), Nitrospirota (22), Planctomycetota (15), UBP10 (14), Bdellovibrionota (13), Patescibacteria (9; includes Candidate Phylum Radiation), Spirochaetota (8), Nitrospinota (7), Myxococcota (4), Entotheonella (2) and the archaeal class Nitrososphaeria (21; phylum Crenarchaeota), hereafter referred to by their historical name “Thaumarchaeota” for name recognition. Mapping of the metagenomic reads to the recovered MAGs showed that the communities had high intraspecies similarity across replicates, consistent with previous 16S rRNA gene-based analyses (Fig. S1). In general, taxa present in *A. aerophoba*, *C. foliascens*, *C. orientalis* and *S. flabelliformis* appeared unique to those sponge species, with only one dominant lineage present in *C. orientalis* (order Parvibaculales). In contrast, several Actinobacteriota, Acidobacteriota and Cyanobacteria populations were shared across *C. matthewsi*, *R. odorabile* and *I. ramosa*. Further, members of the Thaumarchaeota were detected in all sponge species and were particularly abundant in *S. flabelliformis* at 12 ± 4% relative abundance (Fig. S1). Addition of these sponge MAGs to genome trees comprising all publicly available sponge symbionts (*N* = 1188 MAGs) resulted in a phylogenetic gain of 44 and 75% for Bacteria and Archaea, respectively, reflecting substantial novel genomic diversity (Fig. 1).

Comparative genomic analysis of the sponge-derived MAGs provided unique insights into the distribution of metabolic pathways across sponge symbiont taxa. For example, microbial oxidation of ammonia benefits the sponge host by preventing ammonia from accumulating to toxic levels [46], a process thought to be mediated by both symbiotic Bacteria and Archaea (i.e. Thaumarchaeota) [33]. Prior identification of ammonia oxidisers has been based on functional inference from phylogeny (16S rRNA gene amplicon surveys) [47] or homology to specific Pfams (metagenomes) [33]. However, the CuMMO gene family is diverse, encompassing functionally distinct relatives that include *amoA*, particulate methane monooxygenases and hydrocarbon monooxygenases that cannot be distinguished by homology alone [35]. We used GraftM [32] to recover CuMMO genes from the sponge MAGs and their metagenomic assemblies, as well as previously sequenced metagenomic assemblies from six additional sponge microbiomes where bacterial *amoA* gene sequences had been identified [33]. Phylogenetic analysis of the recovered CuMMO genes showed that all archaeal homologues came from Thaumarchaeota and fell within the archaeal *amoA* clade. In contrast, bacterial CuMMO sequences were identified exclusively in MAGs from the phylum UBP10 (formerly unclassified Deltaproteobacteria) and from an unknown taxonomic group in the previous metagenomic assemblies [33]. All recovered bacterial and taxonomically unidentified CuMMO placed within the Deltaproteobacteria/Actinobacteria *hmo* clade, indicating these genes are specific for hydrocarbons rather than ammonia (Fig. S2). The finding that Thaumarchaeota are the only microbes within any of the surveyed sponge species capable of oxidising ammonia, and their ubiquity across sponges, suggests they are a keystone species for this process.

To further investigate the distribution of functions within the sponge microbiome, a set of highly complete (>85%) sponge symbiont MAGs were grouped by principal components analysis based on their KEGG and Pfam annotations, as well as orthologous clusters that reflected all gene content. Similar analysis conducted on 37 MAGs from the sponge *Aplysina aerophoba* suggested the presence of functional guilds, with MAGs from disparate microbial phyla carrying out similar metabolic processes [12] (e.g. carnitine catabolism). Here, we find that MAGs clustered predominately by microbial taxonomy (phylum) rather than function in all three analyses (Fig. S3). While functional guilds could not be identified based on analysis of total genome content, this does not preclude the existence of such guilds based on more specific metabolic pathways.

To identify pathways enriched within the sponge microbiome, sponge-associated MAGs with >85% completeness (*N* = 798) were compared with a set of coral reef and coastal seawater MAGs (*N* = 86), 31 derived from published datasets [31] and 55 from this study (Table S1). Seawater MAGs with >85% genome completeness (93 ± 4% completeness and 2 ± 2% contamination; Table S1) spanned the bacterial phyla Proteobacteria (48 MAGs), Bacteroidota (13), Planctomycetota (5), Myxococcota (5), Gemmatimonadota (3), Marinisomatota (3), Actinobacteriota (3), Verrucomicrobiota (2), Cyanobacteriota (2), Bdellovibrionota (1) and the archaeal phylum Nanoarchaeota (1). Comparative analysis revealed that sponge symbionts were enriched in metabolic pathways for carbohydrate metabolism, defence against infection by MGE, amino acid synthesis, eukaryote-like gene repeat proteins (ELRs) and cell–cell attachment (Tables S2–S4).

**Fig. 2:**
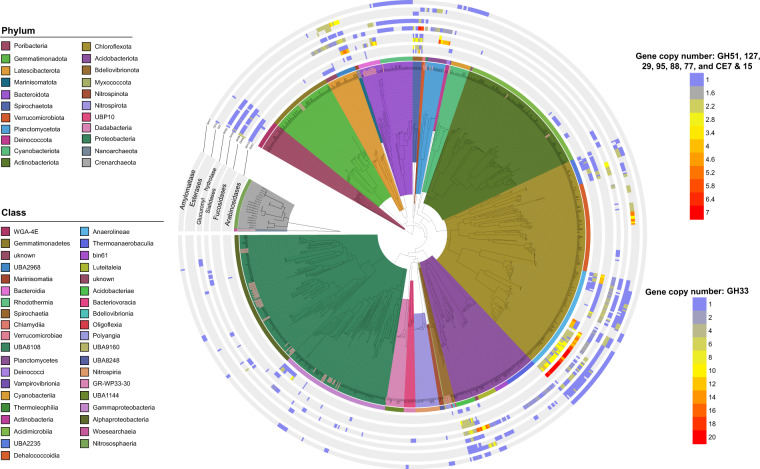
Phylogenetic tree showing the distribution of glycosyl hydrolases and esterases across MAGs with >85% completeness (*N* = 884). Values represent the copy number of each gene per MAG. Internal branches of the tree are coloured by phylum, while the outer strip is coloured by class. Both are listed clockwise in the order in which they appear. Seawater MAGs are denoted by grey labels with red text.

Genes belonging to GH and carbohydrate esterase (CE) families (Table S2) acting on starch (GH77), arabinose (CAZY families GH127 and GH51), fucose (GH95 and GH29) and xylan polymers (CE7 and CE15), were enriched in sponge-associated lineages, likely reflecting the hosts critical role in catabolising dissolved organic matter (DOM) present in reef seawater (Fig. 2). Microbial GHs from the GH77 family target starch, the main sugar storage compound in marine algae [48], whereas GHs from families 51 and 127 are known to act on plant arabinosaccharides, such as the hydroxyproline-linked arabinosaccharides found in algal extensin glycoproteins [49, 50]. GH127 enzymes are also required for microbial degradation of carrageenan, a complex heteropolysaccharide produced by red algae [51]. Members of the fucosidase GH95 and GH29 enzyme families are known to degrade fucoidan, a complex fucosaccharide prominent in brown algae [50, 52]. Notably, arabino- and fucopolysaccharides also make up a significant proportion of coral mucus, a major component of DOM in coral reefs that sponges have been shown to utilise [53, 54]. Supporting this observation, isotopic investigation of the fate of coral mucus and algal polysaccharides in sponges showed that the microbiome participates in metabolism of these compounds, particularly in sponges with high microbial abundance and diversity [4, 5]. Enzymes from the CE families 15 and 7 have been primarily characterised in terrestrial plants where they act as glucuronyl esterases and acetyl-xylan esterases, degrading lignocellulose and removing acetyl groups from hemicellulose [55] (e.g. xylans). Characterisation of CE15 and CE7 from marine microbes is rare, though activity on xylans, which are a structural component of marine algae, has previously been demonstrated [55–57].

GHs acting on sialic acids (GH33) and glycosaminoglycans (GH88) were also enriched in the sponge-associated MAGs and may act on compounds found within sponge tissue [13] (Fig. 2). In contrast, no genes for the degradation of collagen (collagenases), one of the main structural components of the sponge skeleton were identified. Sialic acid-linked residues are found in the sponge mesohyl [58], and although the impact of cleavage on the host is unknown, analogy can be made to other symbioses. For example, sialidases are common in the commensal bacteria present in the human gut where they are used to cleave and metabolise the sialic acid-containing mucins lining the gut wall [59]. Increased sialidase activity is associated with gut dysbiosis and inflammation [60] and careful control of sialidase-containing commensals is therefore necessary to maintain gut homoeostasis [59]. As glycosaminoglycans are also part of sponge tissue [13, 61], the same may apply to microorganisms encoding GH88 family enzymes. However, these genes are also implicated in the degradation of external sugar compounds, such as ulvans, a major sugar storage compound found in green algae that can make up to 30% of their dry weight [62]. Thus, the ecological role of GH88 family enzymes within the sponge microbiome requires further investigation.

Enrichment of GHs and CEs was largely restricted to the Poribacteria, Latescibacteria (class UBA2968), Spirochaetota, Chloroflexota (classes UBA2235 and Anaerolineae, but not Dehalococcoides) and Acidobacteriota (class Acidobacteriae). These findings corroborate previous targeted genomic characterisations of the Chloroflexota and Poribacteria [13, 14] but show that they are part of a larger set of polysaccharide-degrading lineages. Identification of disparately related microbial taxa across several sponge lineages (Figs. 1 and 2) that encode similar pathways for polysaccharide degradation, and therefore occupy a similar ecological niche, supports the existence of functional guilds within the sponge microbiome when viewed at the level of individual pathways. Given the fundamental role of marine sponges in recycling coral reef DOM, studies targeting these specific guilds are needed to quantify their contribution to reef DOM transformation.

Because sponges filter and retain biomass from an extensive range of reef taxa (eukaryotic algae, bacteria, archaea, etc), they are exposed to a greatly expanded variety of MGEs from these organisms, including viruses, transposable elements and plasmids [33, 63]. For this reason, sponge-associated microorganisms likely require a diverse toolbox of molecular mechanisms for resisting infection. Both RM and CRISPR systems are capable of recognising and cleaving MGEs as part of the bacterial immune repertoire. RM systems are part of the innate immune system of bacteria and archaea and are encoded by a single (Type II) or multiple proteins (Type I, III and IV) that recognise and cleave foreign DNA based on a defined target sequence. In contrast, CRISPR systems are part of the adaptive immune system of some bacteria and archaea and encode a target sequence derived from the genome of a previous infective agent that is used by a CRISPR-associated protein (CAS) to identify and cleave foreign DNA. RM (Fig. S4) and CAS (Fig. S5) genes were both enriched (Table S3) in the sponge-associated MAGs and relatively evenly distributed across taxa, with the exception of the Planctomycetota and Verrucomicrobiota, where they were largely absent. As these MAGS average 93 +/− 5% completeness, this result is not likely due to genome incompleteness. This finding contrasts with comparative investigations of Planctomycetota genomes from other environments [64] and additional research is required to ascertain the mechanisms used by sponge-associated Planctomycetota and Verrucomicrobiota to avoid infection. Although Type III RM genes were enriched in sponge MAGs, they were also present in all seawater MAGs. In contrast, Types I and II RM genes were present almost exclusively in the sponge-associated MAGs. In conjunction with an enrichment in CRISPR systems, this expanded repertoire of defence systems likely reflects the increased burden from MGEs associated with the hosts role in filtering and concentrating diverse sources of reef biomass. Supporting this hypothesis, metagenomic surveys of sponge-associated viruses revealed a more diverse viral population than what could be recovered from the surrounding seawater [63]. Further, we found that genes encoding toxin-antitoxin systems, which are present on MGEs, such as plasmids, were also enriched in sponge-associated MAGs. These observations suggest that RM and CRISPR systems are important features of microbe-sponge symbiosis, allowing the symbionts to colonise and persist within their host by avoiding viral infection or being overtaken by MGEs.

Pathways for the synthesis of amino acids were also enriched in the sponge microbiome. The inability of animals to produce several essential amino acids has been proposed as a primary reason that they harbor microbial symbionts [65–68] and it has long been thought that sponges acquire at least some of their essential amino acids from their microbiome [69, 70]. Further, gene-centric characterisation of the *Xestospongia muta* and *R. odorabile* microbiomes revealed pathways to synthesise and transport essential amino acids [33, 70]. However, these same amino acid pathways are also used catabolically by the microorganisms, and transporters could simply be importing amino acids into the microbial cell. Further, as sponges are almost constantly filter feeding, essential amino acids could be acquired through consumption of microorganisms present in seawater. Comparison of sponge MAGs with those from seawater revealed enrichment of specific pathways for the synthesis of lysine, arginine, histidine, threonine, valine and isoleucine (Table S4). However, visualisation of the distribution of these genes revealed that almost all MAGs in both sponges and seawater produce all amino acids, though specific lineages may use different pathways to achieve this (Fig. S6). The enrichment observed in the sponge MAGs was therefore ascribed to differences in pathway completeness between sponge-associated and seawater microbes, rather than an enhanced ability of sponge symbionts to produce any specific amino acid. In contrast, compounds, such as taurine, carnitine and creatine have also been proposed as important host-derived carbon sources for symbionts [69], but pathways for their catabolism were enriched in seawater rather than sponge-associated MAGs. While these findings do not invalidate the possibility that microbial communities play a role in amino acid provisioning to the host or that they utilise host-derived taurine, carnitine, or creatine, they suggest that these are not key processes mediating microbe-sponge symbiosis.

**Fig. 3:**
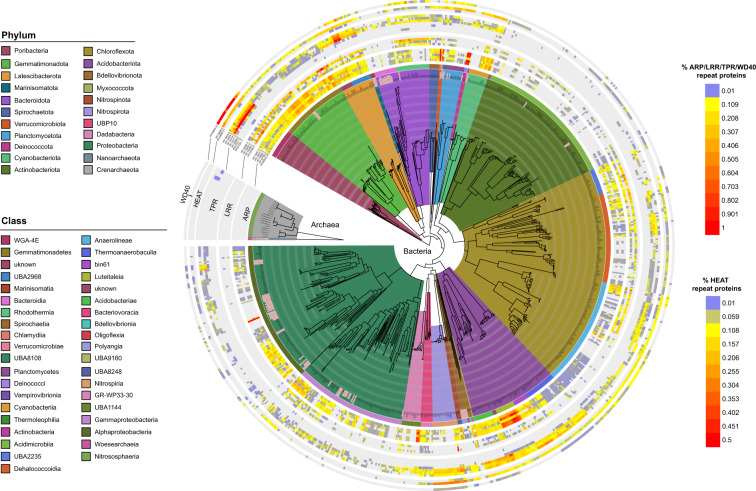
Phylogenetic tree showing the distribution of eukaryote-like repeat proteins—ankyrin (ARP), leucine-rich (LRR), tetratricopeptide (TPR), HEAT and WD40—across MAGS with >85% completeness (*N* = 884). Values represent the percentage of coding genes per MAG devoted to each gene class. Internal branches of the tree are coloured by phylum, while the outer strip is coloured by class, and both are listed clockwise in the order in which they appear. MAGs from seawater are denoted by grey labels with red text.

To form stable symbioses, bacteria must persist within the sponge tissue and avoid phagocytosis by host cells. Microbial proteins containing ELR motifs have been identified in a range of animal and plant-associated microbes and are thought to modulate the host’s intracellular processes to facilitate stable symbiotic associations [71, 72]. For example, ELR-containing proteins from sponge-associated microbes have been shown to confer the ability to evade host phagocytosis when experimentally expressed in *E. coli* [10, 73]. ELR-containing proteins from the ankyrin (ARP), leucine-rich, tetratricopeptide and HEAT repeat families were enriched in the sponge-associated MAGs. In contrast, WD40 repeats were not found to be enriched but are included here as they have previously been reported as abundant in Poribacteria and symbionts of other marine animals [13, 31]. Most ELRs were present across all taxa but were much more prevalent in specific lineages (Fig. 3). For example, sponge-associated Poribacteria, Latescibacterota and Acidobacteriota encoded a high proportion of all ELR types, while other lineages, such as the Gemmatimonadota (average 0.25% coding genes per sponge-associated MAG versus 0.09% in seawater MAGs), Verrucomicrobiota (2%), Deinococcota (0.85%), Acidobacteriota (0.20%; specifically class Luteitaleia at 0.55%) and Dadabacteria from *C. orientalis* (0.62%) encoded a comparatively high percentage of ARPs and Nitrospirota encoded a high percentage of HEAT_2 family proteins (0.55% versus 0.05% in seawater MAGs) relative to other taxa. In contrast, ELR abundances were substantially lower, or absent, in the Actinobacteriota, the class Bacteroidia within the phylum Bacteroidota, and the Thaumarchaeota, suggesting these microorganisms utilise alternative mechanisms to maintain their stable associations with the host.

The mechanisms by which ELRs interact with sponge cells remains largely unknown, although microbes in other host systems are known to deliver ELR-containing effector proteins into host cells via needle-like secretion systems (types III, IV and V) or extracellular contractile injection systems [74, 75], where they interact with the cellular machinery of the host to modify its behaviour. In sponges, it is also possible that ELRs could be secreted into the extracellular space by type I or II secretion systems. Interestingly, although most sponge MAGs encoded eukaryote-like proteins (Fig. 3), few lineages encoded the necessary genes to form secretion systems (Fig. S7). It is therefore unlikely that ELRs are introduced to the sponge host via traditional secretion pathways used in other animal-symbiont systems.

**Fig. 4:**
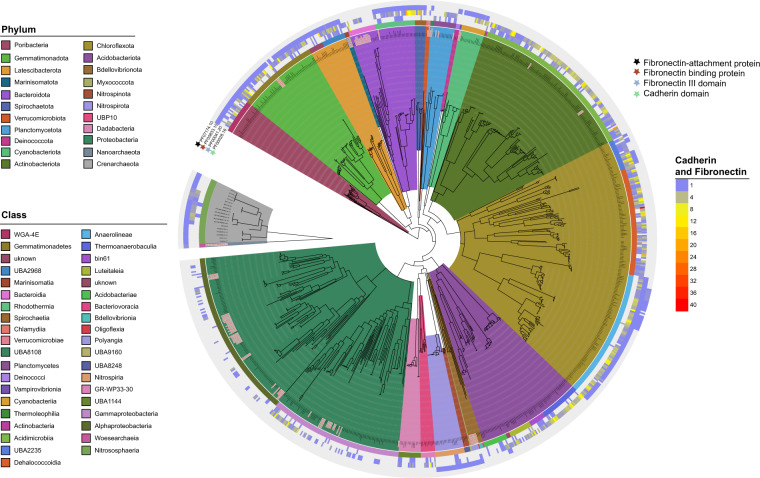
Phylogenetic tree showing the distribution of cadherins, fibronectins and fibronectin-binding proteins across MAGS with >85% completeness (*N* = 884). Values represent the copy number of each gene per MAG. Internal branches of the tree are coloured by phylum while the outer strip is coloured by class. Both are listed clockwise in the order in which they appear. Seawater MAGs are denoted by grey labels with red text.

Maintaining stable association with the sponge may also require mechanisms for attachment to the host tissue. For example, cadherin domains are Ca^2+^-dependent cell–cell adhesion proteins that are abundant in eukaryotes and have been found to serve the same function in bacteria [76]. Similarly, fibronectin III domains mediate cell adhesion in eukaryotes, but also occur in bacteria where they play various roles in carbohydrate binding and biofilm formation [77, 78]. In addition, some bacterial pathogens utilise fibronectin-binding proteins to gain entry into host tissue by binding to host fibronectin [77, 78]. Genes containing cadherin domains were enriched in the sponge-associated MAGs and were identified in most bacterial lineages, but were notably absent in the Cyanobacteriota and Verrucomicrobiota (Fig. 4). Genes containing fibronectin III domains and those for fibronectin-binding proteins were also enriched in sponge-associated MAGs and were distributed across most lineages, though were particularly abundant in the Actinobacteriota and Chloroflexota. However, although fibronectin III-containing genes were taxonomically widespread, those encoding fibronectin-binding proteins were restricted to the phyla Poribacteria, Gemmatimonadota, Latescibacterota, Cyanobacteriota, class Anaerolineae within the Chloroflexota (but not Dehalococcoidia), class Rhodothermia within the Bacteroidota, Spirochaetota, Nitrospirota and the archaeal phylum Thaumarchaeota. Interestingly, the taxonomic distribution of these genes shares significant overlap with lineages encoding the genes for sponge sialic acid and glyosaminoglycans degradation, suggesting that attachment to the host may be necessary for utilisation of these carbohydrates (Fig. 2). However, as the host, bacterial, and archaeal components of the sponge holobiont have fibronectin III domains, symbionts encoding fibronectin-binding proteins may use these to adhere to the host tissue or potentially to form biofilms (bacteria–bacteria attachment). In either case, the enrichment and wide distribution of cadherins, fibronectins and fibronectin-binding proteins in the sponge MAGs suggests that cell–cell adhesion is critical for successful establishment in the sponge niche.

**Fig. 5:**
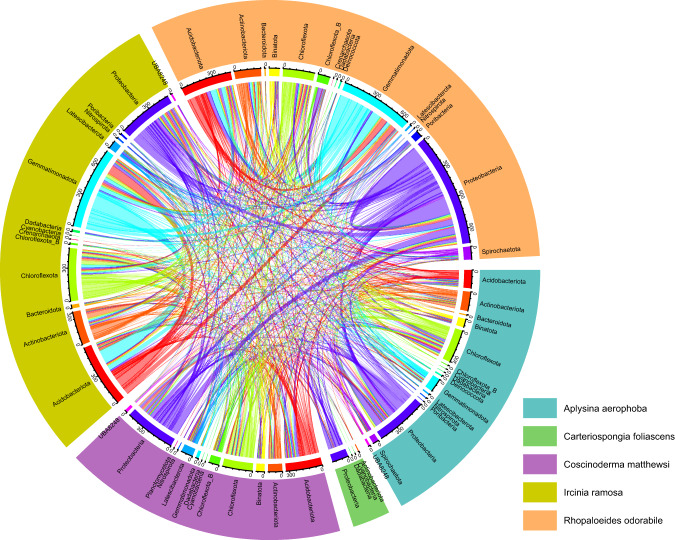
Visualisation of LGTs detected within the MAGs for the five sponges passing the cumulative MAG length criteria (>100 Mbp). The inner strip is coloured by phylum while the outer strip is coloured by host sponges. Bands connect donors and recipients, with their colour corresponding to the donors and the width correlating to the number of LGTs.

**Fig. 6:**
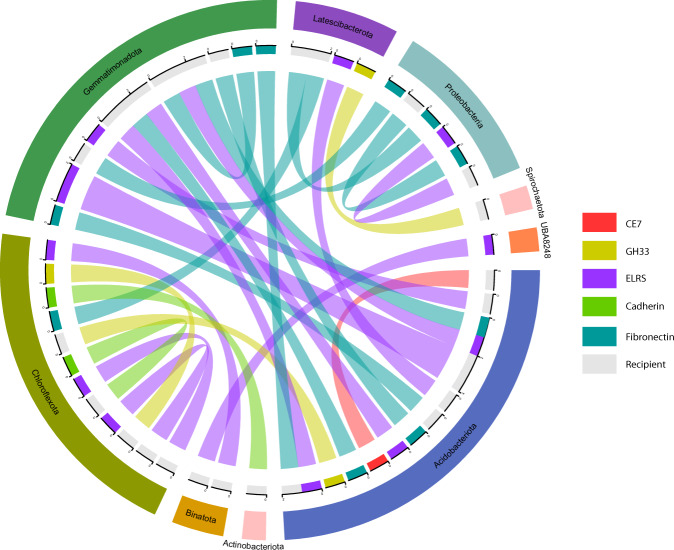
Visualisation of gene flow among microbial phyla for gene families enriched in sponge-associated MAGs. The inner ring and band connecting donor and recipient is coloured by protein family of the gene being transferred, with the width of the band correlating to the number of LGTs. Recipient MAGs are shown in grey. The outer ring is coloured by microbial phylum. Representation of RM and CAS gene LGTs can be found in Fig. S10.

Distribution of genes encoding ELRs, polysaccharide-degrading enzymes (GHs and CEs), cadherins, fibronectins, RMs and CRISPRs across distantly related taxa suggests that they were either acquired from a common ancestor or that they represent more recent LGT events, potentially mediated by MGEs, which are enriched in sponge-associated microbial communities [69]. Here, we identify 4963 LGTs from five sponges for which sufficient sequence data were available (>100 Mbp total sequence length across all MAGs), as well as 136 LGTs from seawater MAGs, averaging 1.64 and 0.52 LGTs per Mbp sequences, respectively (Fig. 5 and Table S5). Sequence similarity of LGTs from MAGs within a sponge species was higher than between sponge species, indicating relatively recent gene transfers (Fig. S8). A higher frequency (Fig. S9) and lower genetic divergence of LGTs among MAGs derived from the same sponge species likely results from the close physical distance between members of each microbiome, as has been observed in other host-symbiont systems [79]. The identification of lateral transfers between microbes from different sponge species may highlight the horizontal acquisition of these microbes or that a recent ancestor inhabited the same host. Notably, LGTs included a subset of genes that were enriched within the sponge-associated MAGs, such as GH33 (sialidases) and CE7 (acetyl-xylan esterases), attachment proteins (cadherins and fibronectin III), RM and CAS proteins, and members of all ELR families other than WD40 (Figs. 6 and S10). The observation that a significant number of sponge-enriched genes were laterally transferred between disparate microbial lineages suggests that the processes they mediate provide a strong selective advantage within the sponge niche, though further research is required to validate these findings.

**Fig. 7:**
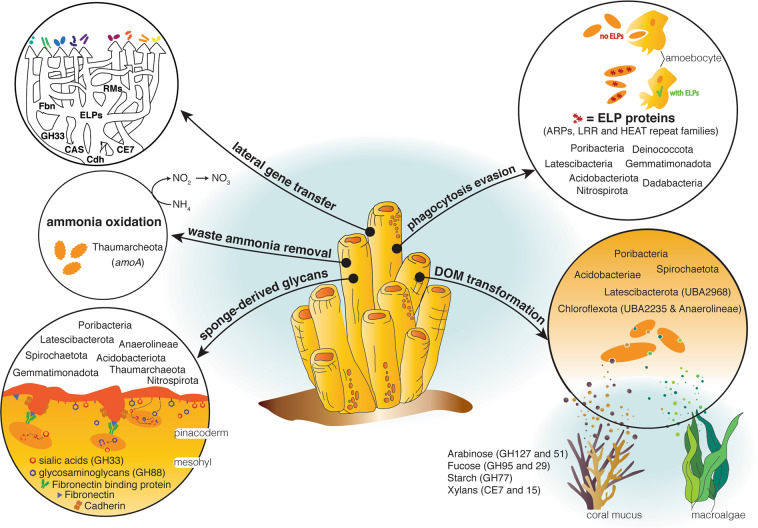
Schematic overview of microbial interactions with the host as inferred from the functional potential encoded by the sponge-associated microbial MAGs. Fbn fibronectin, cdh cadherins, RM restriction-modification systems, CAS CRISPR-associated proteins, ELP eukaryotic-like repeat proteins, CE7 carbohydrate esterase family 7, GH33 glycosyl hydrolase family 33.

Sponges are important constituents of coral reef ecosystems because of their critical role in DOM cycling and retention via the sponge-loop. Despite their importance, functional characterisation of sponge symbiont communities has been restricted to just a few lineages of interest, potentially biasing our view of sponge symbiosis. Here we present a comprehensive characterisation of sponge symbiont MAGs spanning the complete range of taxa found in marine sponges (Fig. 7), most of which were previously uncharacterised. We revealed enrichment in glycolytic enzymes (GHs and CEs) reflecting specific functional guilds capable of aiding the sponge in the degradation of reef DOM. Further, we identified several ELRs, CRISPRs and RMs that likely facilitate stable association with the sponge host, showing the specificity of ELR types with individual microbial lineages. We also clarified the role of Thaumarchaeota as a keystone taxon for ammonia oxidation across sponge species and showed that processes previously thought to be important, such as amino acid provisioning and taurine, creatine and carnitine metabolism are unlikely to be central mechanisms mediating sponge-microbe symbiosis. Many of the enriched genes are laterally transferred between microbial lineages, suggesting that LGT plays an important role in conferring a selective advantage to specific sponge-associated microorganisms. Taken together, these data illustrate how evolutionary processes have distributed and partitioned ecological functions across specific sponge symbiont lineages, allowing them to occupy or share specific niches and live symbiotically with their sponge hosts.

## Supplementary information

File S1

File S2

File S3

File S4

File S5

File S6

File S7

Supplementary Figures S1 to S10

Figure S1

Figure S2

Figure S3

Figure S4

Figure S5

Figure S6

Figure S7

Figure S8

Figure S9

Figure S10

Supplemental tables S1 to S5

## Data Availability

Metagenomic assemblies and MAGs from this study can be found under NCBI bioproject ID PRJNA602572. All GraftM packages can be found at https://data.ace.uq.edu.au/public/graftm/7/. Commands and scripts used to execute analyses and generate figures can be found in File. S7.
