# Supplementary material for: A genomic view of the microbiome of coral reef demosponges
Source: ISME J. 2021 Jan 19;15(6):1641–54. doi: 10.1038/s41396-020-00876-9 (PMC8163846; doi:10.1038/s41396-020-00876-9)
Supplement: Supplementary file 7 — File S7 [file 41396_2020_876_MOESM7_ESM.docx]

Commands used to generate, taxonomically identify, annotate, and statistically analyze the MAGs, along with the scripts necessary to generate the figures.

Note: most commands used to generate the MAGs were executed using GNU parallel to parallelize the processes.

**Because some libraries were made using Nextera and some using Truseq, ran seqpurge twice to remove both adapter sets.**

ls *<path_to_raw_read_directory>*/*_combined_R1.fastq.gz | sed 's/_combined_R1.fastq.gz//' | parallel -j7 SeqPurge -qcut 0 -ncut 0 -threads 8 -in1 {}_combined_R1.fastq.gz -in2 {}_combined_R2.fastq.gz -out1 <path_to_nextera_clipped_reads_directory>/clipped_reads/nexteraSeqpurged_{/}_combined_R1.fastq.gz -out2 *<path_to_nextera_clipped_reads_directory>* /clipped_reads/nexteraSeqpurged_{/}_combined_R2.fastq.gz -a1 CTGTCTCTTATACACATCTCCGAGCCCACGAGAC -a2 CTGTCTCTTATACACATCTGACGCTGCCGACGAT '&>' *<path_to_nextera_clipped_reads_directory>* /clipped_reads/nexteraSeqpurged_{/}.log || eval 'echo "seqpurge command failed to execute" 1>&2; exit 1'

ls *<path_to_nextera_clipped_reads>*/nexteraSeqpurged_*_combined_R1.fastq.gz | sed 's/_combined_R1.fastq.gz//' | parallel -j7 SeqPurge -qcut 0 -ncut 0 -threads 8 -in1 {}_combined_R1.fastq.gz -in2 {}_combined_R2.fastq.gz -out1 <path_to_nextera_and_truseq_clipped_reads>/truseqSeqpurged_{/}_combined_R1.fastq.gz -out2 *<path_to_nextera_and_truseq_clipped_reads>*/truseqSeqpurged_{/}_combined_R2.fastq.gz -a1 AGATCGGAAGAGCACACGTCTGAACTCCAGTCA -a2 AGATCGGAAGAGCGTCGTGTAGGGAAAGAGTGT '&>' *<path_to_nextera_and_truseq_clipped_reads>*/truseqSeqpurged_{/}.log || eval 'echo "seqpurge command failed to execute" 1>&2; exit 1'

**Running assembly using metaspades**

ls *<path_to_adapter_trimmed_reads>*/truseqSeqpurged_*_combined_R1.fastq.gz | grep -v tis_ | sed 's/_combined_R1.fastq.gz//' | parallel -j1 metaspades.py -t25 -m 990 --pe1-1 {}_combined_R1.fastq.gz --pe1-2 {}_combined_R2.fastq.gz -o *<path_to_metaspades_output_directory>*/metaspades_{/} '&>' *<path_to_metaspades_output_directory>*/metaspades_{/}.log || eval 'echo "metaspades command failed to execute" 1>&2; exit 1'

**Index assemblies for mapping using BWA**

ls *<path_to_metaspades_output_directory>*/metaspades_* | grep : | sed 's/://' | sed 's/metaspades_//' | parallel -j7 bwa index *<path_to_metaspades_output_directory>*/metaspades_{/}/scaffolds.fasta || eval 'echo "bwa index failed to execute" 1>&2; exit 1'

**Map reads back to assemblies**

ls *<path_to_metaspades_output_directory>*/metaspades_* | grep : | sed 's/://' | sed 's/metaspades_//' | parallel -j7 bamm make -K -p bammMapping_{=s: *<path_to_metaspades_output_directory>*/::==s:truseqSeqpurged_nexteraSeqpurged_::=}Assembly_ -o *<path_to_mapping_output_directory>* -t 8 -d *<path_to_metaspades_output_directory>*/metaspades_{/}/scaffolds.fasta -c *<path_to_raw_read_directory>*/{=s: *<path_to_metaspades_output_directory>*::==s:[1-4]bac::=}*bac_combined_R*.fastq.gz '&>' *<path_to_mapping_output_directory>* /mapping/bammMapping_{=s: *<path_to_metaspades_output_directory>*/::==s:truseqSeqpurged_nexteraSeqpurged_::=}Assembly.log || eval 'echo "bamm mapping failed to execute" 1>&2; exit 1'

**Bin all samples using uniteM**

ls *<path_to_metaspades_output_directory>*/metaspades_* | grep : | sed 's/://' | sed 's/metaspades_//' | parallel -j5 unitem bin *<path_to_metaspades_output_directory>*/metaspades_{/}/scaffolds.fasta *<path_to_output_binning_directory>*/unitemBin_{=s: *<path_to_metaspades_output_directory>*/::==s:truseqSeqpurged_nexteraSeqpurged_::=} -m2000 -c5 --max40 --max107 --mb2 --mb_verysensitive --mb_sensitive --mb_specific --mb_veryspecific --mb_superspecific --bam_files *<path_to_mapping_output_directory>*/bammMapping_{=s: *<path_to_metaspades_output_directory>*/::==s:truseqSeqpurged_nexteraSeqpurged_::=}*.bam '&>' *<path_to_output_binning_directory>*/unitemBin_{=s: *<path_to_metaspades_output_directory>*/::==s:truseqSeqpurged_nexteraSeqpurged_::=}.log || eval 'echo "unitem bin failed to execute" 1>&2; exit 1'

**Run UniteM profile to get marker genes and genome completeness information**

ls *<path_to_metaspades_output_directory>*/metaspades_* | grep : | sed 's/://' | sed 's/metaspades_//' | parallel -j5 unitem profile *<path_to_output_binning_directory>*/unitemProfile_{=s: *<path_to_metaspades_output_directory>*/::==s:truseqSeqpurged_nexteraSeqpurged_::=} -c6 -f *<path_to_output_binning_directory>*/unitemBin_{=s: *<path_to_metaspades_output_directory>*/::==s:truseqSeqpurged_nexteraSeqpurged_::=}/bin_dirs.tsv '&>' *<path_to_output_binning_directory>*/unitemProfile_{=s: *<path_to_metaspades_output_directory>*/::==s:truseqSeqpurged_nexteraSeqpurged_::=}.log || eval 'echo "unitem pfofile failed to execute" 1>&2; exit 1'

**Run UniteM Consensus to pick final set of best bins**

ls *<path_to_metaspades_output_directory>*/metaspades_* | grep : | sed 's/://' | sed 's/metaspades_//' | parallel -j10 unitem consensus *<path_to_output_binning_directory>*/unitemProfile_{=s: *<path_to_metaspades_output_directory>*/::==s:truseqSeqpurged_nexteraSeqpurged_::=} *<path_to_output_binning_directory>*/unitemConsensus_{=s: *<path_to_metaspades_output_directory>*/::==s:truseqSeqpurged_nexteraSeqpurged_::=} -w3 -f *<path_to_output_binning_directory>*/unitemBin_{=s: *<path_to_metaspades_output_directory>*/::==s:truseqSeqpurged_nexteraSeqpurged_::=}/bin_dirs.tsv '&>' *<path_to_output_binning_directory>*/unitemConsensus_{=s: *<path_to_metaspades_output_directory>*/::==s:truseqSeqpurged_nexteraSeqpurged_::=}.log || eval 'echo "unitem consensus failed to execute" 1>&2; exit 1'

**GTDBtk to assign taxonomy**

gtdbtk classify_wf --genome_dir *<path_to_genome_directory>* --out_dir *<output_directory_name>* --cpus 30

**Run IQTree to calculate trees (same settings used for all tree figures)**

iqtree -s *<msa_sequence_alignment_file_from_GTDBtk>* -m LG+G -nt AUTO -ntmax 20 > logfile.log

Note: newick tree file was then dropped into ITOL (https://itol.embl.de/) for manual addition of genome annotation metadata.

**Run enrichM annotate to annotate bins**

enrichm annotate --genome_directory *<path_to_directory_containing_MAGs>* --output *<output_directory_name>* --ko --pfam --tigrfam --cazy --ec --orthologs --threads 8 --cut_ga &> logfile.log

**Run enrichM enrichment to calculate sets of enriched genes**

enrichm enrichment --processes 25 --ko --annotate_output *<enrichm_annotate_directory>* --metadata *<enrichm_metadata_file>* --output *<enrichment_output_directory>* **Commands used to run Metachip**

MetaCHIP PI -i *<path_to_directory_contain_MAGs>* -taxon Sponge_MAGs.GTDB.classifications.tsv -p SpongeMAGs_dRep99 -r pcofg -x fna -t 12

MetaCHIP BP -p SpongeMAGs_dRep99 -r pcofg -t 12 -cov 90 -ei 80 -flk 20

Note: The id of the dereplicated MAGs subjected to HGT analysis can be found in attached file_in/dereplicated_939_bins.txt.

**Commands/scripts used to get HGT analyses related figures**

python3 scripts/Figure_5.py

python3 scripts/Figure_S8.py

python3 scripts/Figure_S9_S10.py

python3 scripts/Figure_S11.py

Note: A copy of the MAGs metadata in plain text format, all MetaCHIP detected HGTs as well as two HGT subsets with interested functions described in Figure 5 and S11 were provided together with the scripts. Figures were then modified manually in illustrator

**Scripts for LGT figure generation**

**Start python script Figure_5.py**

#!/usr/bin/env python3

import os

def Get_circlize_plot(multi_level_detection, output_prefix, pwd_candidates_file_PG_normal_txt, genome_to_taxon_dict, taxon_rank):

pwd_cir_plot_t1 = '%s_cir_plot_t1.txt' % (output_prefix)

pwd_cir_plot_t1_sorted = '%s_cir_plot_t1_sorted.txt' % (output_prefix)

pwd_cir_plot_t1_sorted_count = '%s_cir_plot_t1_sorted_count.txt' % (output_prefix)

pwd_cir_plot_matrix_filename = '%s_cir_plot_matrix.csv' % (output_prefix)

name2taxon_dict = {}

transfers = []

for each in open(pwd_candidates_file_PG_normal_txt):

if not each.startswith('Gene_1'):

each_split = each.strip().split('\t')

Gene_1 = each_split[0]

Gene_2 = each_split[1]

Genome_1 = '_'.join(Gene_1.split('_')[:-1])

Genome_2 = '_'.join(Gene_2.split('_')[:-1])

if Genome_1 in genome_to_taxon_dict:

Genome_1_taxon = genome_to_taxon_dict[Genome_1]

else:

Genome_1_taxon = '%s_' % taxon_rank

if Genome_2 in genome_to_taxon_dict:

Genome_2_taxon = genome_to_taxon_dict[Genome_2]

else:

Genome_2_taxon = '%s_' % taxon_rank

Direction = each_split[5]

if multi_level_detection == True:

Direction = each_split[6]

if '%)' in Direction:

Direction = Direction.split('(')[0]

if Genome_1 not in name2taxon_dict:

name2taxon_dict[Genome_1] = Genome_1_taxon

if Genome_2 not in name2taxon_dict:

name2taxon_dict[Genome_2] = Genome_2_taxon

transfers.append(Direction)

tmp1 = open(pwd_cir_plot_t1, 'w')

all_group_id = []

for each_t in transfers:

each_t_split = each_t.split('-->')

donor = each_t_split[0]

recipient = each_t_split[1]

donor_id = name2taxon_dict[donor]

recipient_id = name2taxon_dict[recipient]

if donor_id not in all_group_id:

all_group_id.append(donor_id)

if recipient_id not in all_group_id:

all_group_id.append(recipient_id)

tmp1.write('%s,%s\n' % (donor_id, recipient_id))

tmp1.close()

os.system('cat %s | sort > %s' % (pwd_cir_plot_t1, pwd_cir_plot_t1_sorted))

current_t = ''

count = 0

tmp2 = open(pwd_cir_plot_t1_sorted_count, 'w')

for each_t2 in open(pwd_cir_plot_t1_sorted):

each_t2 = each_t2.strip()

if current_t == '':

current_t = each_t2

count += 1

elif current_t == each_t2:

count += 1

elif current_t != each_t2:

tmp2.write('%s,%s\n' % (current_t, count))

current_t = each_t2

count = 1

tmp2.write('%s,%s\n' % (current_t, count))

tmp2.close()

# read in count as dict

transfer_count = {}

for each_3 in open(pwd_cir_plot_t1_sorted_count):

each_3_split = each_3.strip().split(',')

key = '%s,%s' % (each_3_split[0], each_3_split[1])

value = each_3_split[2]

transfer_count[key] = value

all_group_id = sorted(all_group_id)

matrix_file = open(pwd_cir_plot_matrix_filename, 'w')

matrix_file.write('\t' + '\t'.join(all_group_id) + '\n')

for each_1 in all_group_id:

row = [each_1]

for each_2 in all_group_id:

current_key = '%s,%s' % (each_2, each_1)

if current_key not in transfer_count:

row.append('0')

else:

row.append(transfer_count[current_key])

matrix_file.write('\t'.join(row) + '\n')

matrix_file.close()

# rm tmp files

os.system('rm %s' % pwd_cir_plot_t1)

os.system('rm %s' % pwd_cir_plot_t1_sorted)

os.system('rm %s' % pwd_cir_plot_t1_sorted_count)

MAG_to_taxon_dict = {'RHO3_bin_73': 'Gemmatimonadota', 'Ruegeria_spAU67': 'Proteobacteria', 'COS3_bin_18': 'Chloroflexota', 'COS3_bin_8': 'Nitrospirota', 'IRC_PAM_SB0661_bin_39': 'Chloroflexota', 'RHO2_bin_16': 'Bacteroidota', 'seawater_22112_bin_1': 'Bacteroidota', 'IRC1_bin_13': 'Gemmatimonadota', 'IRC4_bin_12': 'Acidobacteriota', 'COS1_bin_10': 'Chloroflexota', 'GCF_000156235': 'Proteobacteria', 'IRC_PAM_SB0662_bin_8': 'Gemmatimonadota', 'CAR3_bin_12': 'Proteobacteria', 'APA_bin_94': 'Spirochaetota', 'COS36405_bin_14': 'Chloroflexota', 'COS2_bin_10': 'Proteobacteria', 'seawater_22112_bin_15': 'Proteobacteria', 'seawater_bettina_36310_bin_3': 'Proteobacteria', 'IRC1_bin_25': 'Chloroflexota', 'IRC_PAM_SB0670_bin_19': 'Chloroflexota', 'CAR4_bin_9': 'Proteobacteria', 'IRC2_bin_4': 'Actinobacteriota', 'RHO1_bin_2': 'Proteobacteria', 'RHO2_bin_37': 'Actinobacteriota', 'COS4_bin_13': 'Chloroflexota', 'RHO1_bin_50': 'Gemmatimonadota', 'IRC_PAM_SB0661_bin_8': 'Acidobacteriota', 'IRC_PAM_SB0661_bin_29': 'Poribacteria', 'COS3_bin_5': 'Actinobacteriota', 'IRC_PAM_SB0661_bin_55': 'Actinobacteriota', 'RHO1_bin_60': 'Proteobacteria', 'CAR1_bin_15': 'Cyanobacteria', 'COS3_bin_17': 'Chloroflexota', 'CAR2_bin_9': 'Proteobacteria', 'COS4_bin_25': 'Actinobacteriota', 'CAR1_bin_6': 'Proteobacteria', 'APA_bin_52': 'Chloroflexota', 'seawater_seasim_SB9153_S2_bin_10': 'Proteobacteria', 'COS3_bin_2': 'Chloroflexota', 'COS4_bin_10': 'Chloroflexota', 'STY1_bin_5': 'Crenarchaeota', 'seawater_seasim_SB9154_S3_bin_3': 'Myxococcota', 'APA_bin_1': 'Chloroflexota', 'IRC2_bin_10': 'Actinobacteriota', 'RHO2_bin_59': 'Chloroflexota', 'COS36386_bin_9': 'Proteobacteria', 'CAR4_bin_15': 'Proteobacteria', 'RHO2_bin_67': 'Chloroflexota', 'RHO2_bin_36': 'Chloroflexota', 'STY2_bin_7': 'Cyanobacteria', 'COS36387_bin_22': 'Chloroflexota', 'seawater_seasim_SB9152_S1_bin_1': 'Proteobacteria', 'APA_bin_43': 'Chloroflexota', 'RHO2_bin_25': 'Crenarchaeota', 'GCA_000200715': 'Crenarchaeota', 'RHO1_bin_81': 'Acidobacteriota', 'APA_bin_89': 'Chloroflexota', 'COS36387_bin_6': 'Acidobacteriota', 'RHO2_bin_33': 'Proteobacteria', 'RHO3_bin_14': 'Proteobacteria', 'COS2_bin_5': 'Acidobacteriota', 'IRC_PAM_SB0676_bin_26': 'Gemmatimonadota', 'RHO2_bin_28': 'Bacteroidota', 'COS3_bin_15': 'Chloroflexota', 'RHO1_bin_3': 'Actinobacteriota', 'COS36386_bin_3': 'Proteobacteria', 'IRC_PAM_SB0661_bin_1': 'Acidobacteriota', 'COS36388_bin_17': 'Chloroflexota', 'CAR2_bin_6': 'Bacteroidota', 'RHO3_bin_32': 'Actinobacteriota', 'RHO1_bin_14': 'Chloroflexota', 'APA_bin_33': 'Chloroflexota', 'RHO3_bin_67': 'Chloroflexota', 'seawater_seasim_SB9152_S1_bin_19': 'Bdellovibrionota_B', 'CAR3_bin_4': 'Proteobacteria', 'IRC3_bin_2': 'Proteobacteria', 'COS4_bin_6': 'Acidobacteriota', 'COS3_bin_16': 'Actinobacteriota', 'IRC_PAM_SB0667_bin_14': 'Proteobacteria', 'CAR1_bin_14': 'Proteobacteria', 'IRC_PAM_SB0677_bin_16': 'Crenarchaeota', 'RHO1_bin_28': 'Latescibacterota', 'COS4_bin_22': 'Chloroflexota', 'GCA_000522425': 'Tectomicrobia', 'COS36405_bin_7': 'Actinobacteriota', 'APA_bin_86': 'Chloroflexota', 'IRC_PAM_SB0661_bin_5': 'Dadabacteria', 'CAR3_bin_7': 'Actinobacteriota', 'CAR4_bin_5': 'Proteobacteria', 'COS4_bin_12': 'Proteobacteria', 'IRC_PAM_SB0665_bin_1': 'Chloroflexota', 'CAR1_bin_1': 'Proteobacteria', 'COS1_bin_8': 'Proteobacteria', 'COS2_bin_6': 'Actinobacteriota', 'RHO2_bin_10': 'Actinobacteriota', 'RHO2_bin_50': 'Gemmatimonadota', 'IRC_PAM_SB0678_bin_2': 'Proteobacteria', 'RHO3_bin_23': 'Chloroflexota', 'COS36406_bin_6': 'Planctomycetota', 'RHO3_bin_13': 'Actinobacteriota', 'COS1_bin_2': 'Chloroflexota', 'IRC1_bin_17': 'Proteobacteria', 'IRC_PAM_SB0668_bin_20': 'Latescibacterota', 'RHO1_bin_62': 'Poribacteria', 'GCF_000158135': 'Proteobacteria', 'RHO1_bin_49': 'Gemmatimonadota', 'COS3_bin_12': 'Chloroflexota', 'seawater_seasim_SB9156_S5_bin_4': 'Marinisomatota', 'IRC1_bin_31': 'Chloroflexota', 'APA_bin_83': 'Poribacteria', 'APA_bin_22': 'Proteobacteria', 'CAR2_bin_1': 'Actinobacteriota', 'IRC3_bin_22': 'Chloroflexota', 'RHO3_bin_12': 'Acidobacteriota', 'IRC_PAM_SB0677_bin_15': 'Nitrospirota', 'RHO2_bin_42': 'Chloroflexota', 'CLI1_bin_3': 'Proteobacteria', 'COS36386_bin_6': 'Planctomycetota', 'CAR2_bin_7': 'Proteobacteria', 'RHO1_bin_20': 'Chloroflexota', 'IRC1_bin_11': 'Poribacteria', 'IRC_PAM_SB0665_bin_25': 'Chloroflexota', 'seawater_bettina_36310_bin_4': 'Proteobacteria', 'IRC_PAM_SB0664_bin_2': 'Proteobacteria', 'CAR4_bin_16': 'Proteobacteria', 'GCA_003635305': 'Poribacteria', 'RHO1_bin_69': 'Gemmatimonadota', 'IRC_PAM_SB0661_bin_11': 'Latescibacterota', 'seawater_42617_bin_1': 'Proteobacteria', 'RHO1_bin_25': 'Actinobacteriota', 'IRC4_bin_7': 'Actinobacteriota', 'STY2_bin_4': 'Crenarchaeota', 'RHO3_bin_68': 'Chloroflexota', 'RHO2_bin_31': 'Acidobacteriota', 'COS4_bin_1': 'Actinobacteriota', 'COS4_bin_7': 'Proteobacteria', 'CAR2_bin_10': 'Proteobacteria', 'IRC_PAM_SB0668_bin_14': 'Acidobacteriota', 'APA_bin_96': 'Chloroflexota', 'RHO1_bin_59': 'Proteobacteria', 'APA_bin_24': 'Nitrospirota', 'IRC_PAM_SB0661_bin_16': 'Chloroflexota', 'RHO1_bin_34': 'Acidobacteriota', 'COS3_bin_13': 'Acidobacteriota', 'RHO3_bin_35': 'Actinobacteriota', 'seawater_seasim_SB9152_S1_bin_2': 'Myxococcota', 'seawater_22112_bin_8': 'Proteobacteria', 'COS36388_bin_13': 'Proteobacteria', 'IRC_PAM_SB0662_bin_35': 'Poribacteria', 'IRC_PAM_SB0665_bin_19': 'Acidobacteriota', 'RHO3_bin_57': 'Proteobacteria', 'IRC_PAM_SB0673_bin_10': 'Cyanobacteria', 'RHO2_bin_38': 'Actinobacteriota', 'IRC3_bin_28': 'Gemmatimonadota', 'seawater_seasim_SB9156_S5_bin_14': 'Cyanobacteria', 'RHO2_bin_11': 'Nitrospirota', 'GCA_001542995': 'Nitrospinota', 'RHO1_bin_23': 'Dadabacteria', 'IRC1_bin_23': 'Latescibacterota', 'seawater_22112_bin_5': 'Planctomycetota', 'APA_bin_98': 'Actinobacteriota', 'CAR3_bin_9': 'Proteobacteria', 'IRC3_bin_20': 'Cyanobacteria', 'IRC_PAM_SB0662_bin_27': 'Chloroflexota', 'IRC_PAM_SB0675_bin_14': 'Latescibacterota', 'COS36406_bin_19': 'Binatota', 'APA_bin_6': 'Chloroflexota', 'GCF_900143565': 'Proteobacteria', 'CLI1_bin_1': 'Dadabacteria', 'IRC_PAM_SB0675_bin_16': 'Actinobacteriota', 'IRC1_bin_15': 'Chloroflexota', 'IRC3_bin_10': 'Chloroflexota', 'RHO2_bin_55': 'Acidobacteriota', 'GCF_900143545': 'Proteobacteria', 'RHO1_bin_29': 'Proteobacteria', 'COS36386_bin_1': 'Chloroflexota', 'COS36386_bin_2': 'Nitrospirota', 'GCA_001007665': 'Cyanobacteria', 'COS4_bin_47': 'Latescibacterota', 'CAR1_bin_12': 'Proteobacteria', 'IRC_PAM_SB0666_bin_1': 'Chloroflexota', 'IRC_PAM_SB0668_bin_11': 'Proteobacteria', 'seawater_bettina_36326_bin_7': 'Bacteroidota', 'IRC2_bin_5': 'Acidobacteriota', 'RHO3_bin_38': 'Gemmatimonadota', 'RHO1_bin_37': 'Crenarchaeota', 'COS36406_bin_2': 'Nitrospirota', 'COS36406_bin_9': 'Acidobacteriota', 'GCA_000583135': 'Proteobacteria', 'seawater_bettina_36309_bin_6': 'Proteobacteria', 'COS3_bin_6': 'Actinobacteriota', 'CAR1_bin_16': 'Bacteroidota', 'IRC3_bin_6': 'Chloroflexota', 'RHO3_bin_50': 'Chloroflexota', 'COS36406_bin_13': 'Crenarchaeota', 'IRC_PAM_SB0662_bin_1': 'Chloroflexota', 'IRC_PAM_SB0662_bin_49': 'Poribacteria', 'seawater_seasim_SB9153_S2_bin_2': 'Proteobacteria', 'CAR2_bin_20': 'Dadabacteria', 'seawater_seasim_SB9155_S4_bin_4': 'Proteobacteria', 'seawater_seasim_SB9152_S1_bin_4': 'Marinisomatota', 'APA_bin_97': 'Deinococcota', 'CAR3_bin_16': 'Bacteroidota', 'IRC3_bin_13': 'Chloroflexota', 'seawater_seasim_SB9156_S5_bin_8': 'Proteobacteria', 'IRC3_bin_15': 'Chloroflexota', 'CAR4_bin_3': 'Bacteroidota', 'seawater_seasim_SB9152_S1_bin_8': 'Proteobacteria', 'CAR2_bin_8': 'Proteobacteria', 'IRC_PAM_SB0661_bin_26': 'Actinobacteriota', 'seawater_42618_bin_3': 'Proteobacteria', 'seawater_22112_bin_9': 'Actinobacteriota', 'RHO2_bin_60': 'Chloroflexota', 'COS4_bin_9': 'Latescibacterota', 'seawater_bettina_36328_bin_3': 'Proteobacteria', 'APA_bin_68': 'Actinobacteriota', 'APA_bin_71': 'Proteobacteria', 'RHO1_bin_48': 'Proteobacteria', 'IRC_PAM_SB0675_bin_20': 'Gemmatimonadota', 'CHO1_bin_4': 'Proteobacteria', 'APA_bin_56': 'Crenarchaeota', 'IRC_PAM_SB0664_bin_24': 'Latescibacterota', 'APA_bin_58': 'Acidobacteriota', 'IRC_PAM_SB0675_bin_29': 'Chloroflexota', 'IRC1_bin_22': 'Gemmatimonadota', 'IRC_PAM_SB0666_bin_15': 'Crenarchaeota', 'RHO3_bin_1': 'Proteobacteria', 'aplysina_bin': 'Latescibacterota', 'IRC4_bin_20': 'Chloroflexota', 'COS4_bin_28': 'Proteobacteria', 'CAR2_bin_22': 'Proteobacteria', 'CAR3_bin_18': 'Proteobacteria', 'GCF_004168585': 'Proteobacteria', 'COS36387_bin_14': 'Proteobacteria', 'COS1_bin_20': 'Chloroflexota', 'GCA_001543005': 'Proteobacteria', 'COS36404_bin_9': 'Actinobacteriota', 'GCA_001007635': 'Cyanobacteria', 'seawater_42615_bin_2': 'Proteobacteria', 'CAR3_bin_11': 'Bacteroidota', 'RHO1_bin_21': 'Actinobacteriota', 'CAR4_bin_1': 'Proteobacteria', 'CAR3_bin_17': 'Proteobacteria', 'COS3_bin_1': 'Chloroflexota', 'IRC_PAM_SB0662_bin_21': 'Chloroflexota', 'IRC1_bin_1': 'Proteobacteria', 'IRC_PAM_SB0665_bin_12': 'Chloroflexota', 'GCA_003635265': 'Poribacteria', 'COS36388_bin_4': 'Actinobacteriota', 'RHO1_bin_72': 'Proteobacteria', 'IRC_PAM_SB0664_bin_11': 'Chloroflexota', 'IRC_PAM_SB0661_bin_6': 'Chloroflexota', 'IRC_PAM_SB0667_bin_9': 'Nitrospirota', 'APA_bin_19': 'Acidobacteriota', 'IRC_PAM_SB0664_bin_14': 'Actinobacteriota', 'IRC3_bin_25': 'Actinobacteriota', 'COS36406_bin_18': 'Binatota', 'RHO3_bin_15': 'Dadabacteria', 'IRC_PAM_SB0661_bin_14': 'Actinobacteriota', 'IRC3_bin_32': 'Acidobacteriota', 'APA_bin_8': 'Dadabacteria', 'IRC3_bin_18': 'Latescibacterota', 'COS3_bin_9': 'Chloroflexota', 'SPOO_karimiCosta_FZLQ01': 'Proteobacteria', 'seawater_seasim_SB9160_S9_bin_11': 'Gemmatimonadota', 'COS1_bin_9': 'Cyanobacteria', 'RHO1_bin_67': 'Acidobacteriota', 'COS36387_bin_4': 'Planctomycetota', 'COS3_bin_10': 'Chloroflexota', 'COS4_bin_21': 'Cyanobacteria', 'IRC_PAM_SB0662_bin_22': 'Acidobacteriota', 'GCF_001941685': 'Proteobacteria', 'IRC1_bin_12': 'Poribacteria', 'seawater_42615_bin_16': 'Bacteroidota', 'IRC_PAM_SB0662_bin_24': 'Bacteroidota', 'APA_bin_59': 'Binatota', 'RHO2_bin_2': 'Proteobacteria', 'COS36387_bin_18': 'Planctomycetota', 'RHO3_bin_20': 'Nitrospirota', 'IRC_PAM_SB0664_bin_25': 'Actinobacteriota', 'COS36405_bin_2': 'Planctomycetota', 'petrosia_ficiformis_bin': 'Latescibacterota', 'STY1_bin_1': 'Proteobacteria', 'RHO3_bin_28': 'Chloroflexota', 'IRC3_bin_9': 'Actinobacteriota', 'IRC4_bin_22': 'Chloroflexota', 'RHO1_bin_82': 'Proteobacteria', 'APA_bin_18': 'Actinobacteriota', 'CAR2_bin_26': 'Proteobacteria', 'RHO2_bin_26': 'Actinobacteriota', 'CAR4_bin_18': 'Proteobacteria', 'IRC_PAM_SB0675_bin_12': 'Acidobacteriota', 'IRC_PAM_SB0665_bin_24': 'Actinobacteriota', 'COS36388_bin_9': 'Acidobacteriota', 'COS36388_bin_5': 'Chloroflexota', 'STY4_bin_7': 'Crenarchaeota', 'RHO3_bin_64': 'Binatota', 'APA_bin_29': 'Acidobacteriota', 'IRC_PAM_SB0664_bin_9': 'Actinobacteriota', 'RHO1_bin_45': 'Spirochaetota', 'RHO3_bin_37': 'Acidobacteriota', 'RHO3_bin_80': 'Proteobacteria', 'COS1_bin_3': 'Chloroflexota', 'IRC_PAM_SB0665_bin_17': 'Actinobacteriota', 'APA_bin_102': 'Acidobacteriota', 'IRC_PAM_SB0664_bin_31': 'Acidobacteriota', 'CAR3_bin_14': 'Proteobacteria', 'IRC4_bin_39': 'Gemmatimonadota', 'RHO3_bin_72': 'Gemmatimonadota', 'IRC4_bin_2': 'Bacteroidota', 'CAR1_bin_11': 'Proteobacteria', 'IRC_PAM_SB0667_bin_13': 'Crenarchaeota', 'IRC_PAM_SB0661_bin_22': 'Chloroflexota', 'IRC4_bin_1': 'Acidobacteriota', 'GCF_002573675': 'Actinobacteriota', 'IRC_PAM_SB0670_bin_22': 'Latescibacterota', 'COS4_bin_49': 'Acidobacteriota', 'seawater_seasim_SB9152_S1_bin_18': 'Proteobacteria', 'GCF_900143635': 'Proteobacteria', 'RHO1_bin_7': 'Chloroflexota', 'RHO3_bin_10': 'Proteobacteria', 'RHO1_bin_32': 'Chloroflexota', 'seawater_bettina_36309_bin_5': 'Bacteroidota', 'IRC_PAM_SB0675_bin_5': 'Acidobacteriota', 'RHO3_bin_71': 'Gemmatimonadota', 'IRC1_bin_30': 'Chloroflexota', 'RHO1_bin_19': 'Acidobacteriota', 'IRC3_bin_31': 'Gemmatimonadota', 'STY3_bin_6': 'Crenarchaeota', 'IRC_PAM_SB0665_bin_2': 'Dadabacteria', 'GCF_001431305': 'Proteobacteria', 'IRC2_bin_13': 'Chloroflexota', 'APA_bin_34': 'Bacteroidota', 'COS36386_bin_20': 'Proteobacteria', 'RHO3_bin_24': 'Proteobacteria', 'COS4_bin_36': 'Chloroflexota', 'STY2_bin_2': 'Proteobacteria', 'IRC_PAM_SB0662_bin_12': 'Actinobacteriota', 'IRC_PAM_SB0677_bin_2': 'Proteobacteria', 'CAR2_bin_16': 'Proteobacteria', 'seawater_bettina_36328_bin_2': 'Proteobacteria', 'COS36388_bin_6': 'Chloroflexota', 'seawater_42618_bin_5': 'Proteobacteria', 'COS2_bin_12': 'Proteobacteria', 'seawater_bettina_36327_bin_7': 'Bacteroidota', 'IRC_PAM_SB0664_bin_16': 'Chloroflexota', 'COS4_bin_55': 'Proteobacteria', 'seawater_seasim_SB9155_S4_bin_12': 'Myxococcota', 'RHO3_bin_19': 'Proteobacteria', 'GCF_000743705': 'Proteobacteria', 'seawater_bettina_36327_bin_4': 'Bacteroidota', 'COS36388_bin_8': 'Planctomycetota', 'RHO3_bin_69': 'Poribacteria', 'COS36386_bin_37': 'Cyanobacteria', 'IRC_PAM_SB0673_bin_16': 'Latescibacterota', 'IRC_PAM_SB0678_bin_15': 'Proteobacteria', 'CAR3_bin_6': 'Bacteroidota', 'IRC4_bin_45': 'Latescibacterota', 'IRC4_bin_42': 'Proteobacteria', 'APA_bin_63': 'Chloroflexota', 'IRC1_bin_27': 'Latescibacterota', 'CAR2_bin_18': 'Proteobacteria', 'seawater_bettina_36327_bin_2': 'Cyanobacteria', 'RHO1_bin_24': 'Nitrospirota', 'COS36386_bin_13': 'Chloroflexota', 'APA_bin_12': 'Bdellovibrionota', 'COS4_bin_42': 'Chloroflexota', 'RHO1_bin_51': 'Gemmatimonadota', 'IRC_PAM_SB0665_bin_13': 'Acidobacteriota', 'RHO3_bin_4': 'Actinobacteriota', 'RHO2_bin_20': 'Poribacteria', 'COS1_bin_13': 'Acidobacteriota', 'IRC_PAM_SB0664_bin_22': 'Chloroflexota', 'RHO3_bin_59': 'Proteobacteria', 'RHO3_bin_22': 'Chloroflexota', 'IRC_PAM_SB0678_bin_5': 'Actinobacteriota', 'COS36386_bin_27': 'Binatota', 'COS36405_bin_12': 'Proteobacteria', 'RHO2_bin_3': 'Acidobacteriota', 'APA_bin_45': 'Proteobacteria', 'CAR4_bin_6': 'Cyanobacteria', 'seawater_bettina_36308_bin_5': 'Proteobacteria', 'COS36386_bin_4': 'Chloroflexota', 'IRC3_bin_11': 'Chloroflexota', 'IRC_PAM_SB0664_bin_27': 'Chloroflexota', 'APA_bin_4': 'Actinobacteriota', 'IRC4_bin_21': 'Chloroflexota', 'RHO3_bin_49': 'Chloroflexota', 'GCA_003635205': 'Poribacteria', 'COS4_bin_3': 'Acidobacteriota', 'GCA_003635315': 'Poribacteria', 'seawater_seasim_SB9152_S1_bin_12': 'Nanoarchaeota', 'CAR4_bin_8': 'Proteobacteria', 'IRC_PAM_SB0666_bin_11': 'Chloroflexota', 'RHO3_bin_18': 'Actinobacteriota', 'CAR1_bin_2': 'Proteobacteria', 'CAR2_bin_2': 'Proteobacteria', 'IRC4_bin_10': 'Actinobacteriota', 'seawater_42616_bin_2': 'Proteobacteria', 'IRC_PAM_SB0664_bin_21': 'Actinobacteriota', 'seawater_22112_bin_29': 'Proteobacteria', 'IRC_PAM_SB0668_bin_13': 'Cyanobacteria', 'CAR4_bin_2': 'Bacteroidota', 'RHO3_bin_25': 'Chloroflexota', 'IRC_PAM_SB0662_bin_19': 'Dadabacteria', 'IRC_PAM_SB0675_bin_2': 'Actinobacteriota', 'APA_bin_80': 'Chloroflexota', 'IRC4_bin_49': 'Gemmatimonadota', 'IRC_PAM_SB0661_bin_4': 'Actinobacteriota', 'COS36406_bin_4': 'Acidobacteriota', 'COS2_bin_7': 'Chloroflexota', 'seawater_42618_bin_29': 'Proteobacteria', 'RHO2_bin_8': 'Chloroflexota', 'IRC_PAM_SB0661_bin_45': 'Chloroflexota', 'RHO1_bin_33': 'Acidobacteriota', 'IRC_PAM_SB0665_bin_8': 'Latescibacterota', 'APA_bin_38': 'Poribacteria', 'IRC_PAM_SB0675_bin_19': 'Acidobacteriota', 'COS4_bin_51': 'Chloroflexota', 'IRC_PAM_SB0667_bin_3': 'Bacteroidota', 'Aquimarina_spAU474': 'Bacteroidota', 'RHO3_bin_33': 'Bdellovibrionota', 'COS4_bin_17': 'Crenarchaeota', 'COS3_bin_4': 'Chloroflexota', 'seawater_bettina_36326_bin_1': 'Proteobacteria', 'RHO3_bin_29': 'Actinobacteriota', 'CAR3_bin_2': 'Proteobacteria', 'RHO2_bin_35': 'Dadabacteria', 'RHO2_bin_54': 'Gemmatimonadota', 'IRC4_bin_8': 'Chloroflexota', 'IRC_PAM_SB0675_bin_18': 'Chloroflexota', 'COS4_bin_19': 'Proteobacteria', 'IRC_PAM_SB0664_bin_12': 'Proteobacteria', 'IRC1_bin_6': 'Bacteroidota', 'IRC4_bin_15': 'Chloroflexota', 'IRC_PAM_SB0661_bin_33': 'Gemmatimonadota', 'IRC_PAM_SB0661_bin_21': 'Chloroflexota', 'APA_bin_40': 'Proteobacteria', 'STY1_bin_8': 'Myxococcota', 'STY3_bin_1': 'Proteobacteria', 'seawater_22112_bin_3': 'Proteobacteria', 'seawater_bettina_36326_bin_26': 'Bacteroidota', 'RHO2_bin_5': 'Latescibacterota', 'GCF_900141785': 'Bacteroidota', 'IRC_PAM_SB0664_bin_33': 'Chloroflexota', 'COS4_bin_14': 'Actinobacteriota', 'COS2_bin_3': 'Nitrospirota', 'GCF_900149705': 'Proteobacteria', 'seawater_bettina_36326_bin_6': 'Proteobacteria', 'COS1_bin_18': 'Proteobacteria', 'IRC_PAM_SB0678_bin_6': 'Chloroflexota', 'RHO1_bin_66': 'Gemmatimonadota', 'IRC_PAM_SB0677_bin_19': 'Gemmatimonadota', 'IRC_PAM_SB0665_bin_20': 'Latescibacterota', 'RHO3_bin_7': 'Latescibacterota', 'IRC_PAM_SB0662_bin_39': 'Acidobacteriota', 'Pseudovibrio_spAU243': 'Proteobacteria', 'COS4_bin_18': 'Latescibacterota', 'IRC1_bin_21': 'Chloroflexota', 'RHO3_bin_31': 'Actinobacteriota', 'RHO3_bin_84': 'Spirochaetota', 'RHO3_bin_36': 'Acidobacteriota', 'IRC1_bin_2': 'Actinobacteriota', 'COS36386_bin_17': 'Actinobacteriota', 'COS4_bin_43': 'Chloroflexota', 'APA_bin_32': 'Chloroflexota', 'IRC4_bin_33': 'Chloroflexota', 'COS1_bin_12': 'Chloroflexota', 'COS36387_bin_15': 'Crenarchaeota', 'IRC_PAM_SB0662_bin_51': 'UBA8248', 'IRC_PAM_SB0672_bin_21': 'Gemmatimonadota', 'IRC4_bin_35': 'Actinobacteriota', 'seawater_bettina_36308_bin_3': 'Proteobacteria', 'COS36387_bin_9': 'Chloroflexota', 'RHO1_bin_40': 'Chloroflexota', 'CAR2_bin_4': 'Cyanobacteria', 'COS3_bin_14': 'Acidobacteriota', 'RHO3_bin_6': 'Poribacteria', 'RHO1_bin_85': 'Proteobacteria', 'RHO1_bin_17': 'Actinobacteriota', 'COS1_bin_4': 'Actinobacteriota', 'IRC4_bin_44': 'Latescibacterota', 'COS4_bin_29': 'Proteobacteria', 'COS4_bin_24': 'Dadabacteria', 'RHO1_bin_4': 'Chloroflexota', 'RHO2_bin_56': 'Actinobacteriota', 'GCA_003635195': 'Poribacteria', 'STY2_bin_1': 'Proteobacteria', 'APA_bin_3': 'Proteobacteria', 'seawater_42617_bin_6': 'Bacteroidota', 'APA_bin_55': 'Proteobacteria', 'COS36405_bin_20': 'Binatota', 'IRC3_bin_3': 'Bacteroidota', 'COS36405_bin_1': 'Nitrospirota', 'IRC_PAM_SB0664_bin_6': 'Chloroflexota', 'IRC1_bin_38': 'Actinobacteriota', 'COS36387_bin_11': 'Proteobacteria', 'CAR1_bin_18': 'Proteobacteria', 'RHO1_bin_39': 'Proteobacteria', 'COS4_bin_16': 'Proteobacteria', 'RHO1_bin_53': 'Proteobacteria', 'GCF_900079515': 'Proteobacteria', 'COS36386_bin_15': 'Proteobacteria', 'IRC_PAM_SB0670_bin_1': 'Actinobacteriota', 'COS4_bin_46': 'Latescibacterota', 'IRC_PAM_SB0677_bin_11': 'Actinobacteriota', 'IRC_PAM_SB0664_bin_4': 'Actinobacteriota', 'COS36386_bin_10': 'Planctomycetota', 'IRC4_bin_9': 'Actinobacteriota', 'CAR3_bin_5': 'Cyanobacteria', 'APA_bin_90': 'Binatota', 'CLI1_bin_4': 'Proteobacteria', 'RHO3_bin_58': 'Proteobacteria', 'RHO2_bin_52': 'Gemmatimonadota', 'RHO3_bin_21': 'Actinobacteriota', 'CHO1_bin_1': 'Proteobacteria', 'COS1_bin_11': 'Crenarchaeota', 'IRC_PAM_SB0672_bin_11': 'Acidobacteriota', 'COS36404_bin_2': 'Acidobacteriota', 'IRC1_bin_26': 'Actinobacteriota', 'seawater_seasim_SB9153_S2_bin_4': 'Myxococcota', 'RHO2_bin_19': 'Poribacteria', 'IRC_PAM_SB0662_bin_53': 'Proteobacteria', 'IRC_PAM_SB0664_bin_15': 'Chloroflexota', 'IRC_PAM_SB0662_bin_58': 'Proteobacteria', 'IRC_PAM_SB0675_bin_3': 'Proteobacteria', 'RHO2_bin_23': 'Proteobacteria', 'COS36404_bin_19': 'Acidobacteriota', 'IRC1_bin_19': 'Chloroflexota', 'COS3_bin_11': 'Gemmatimonadota', 'seawater_seasim_SB9156_S5_bin_6': 'Proteobacteria', 'IRC3_bin_35': 'Chloroflexota', 'IRC_PAM_SB0666_bin_10': 'Actinobacteriota', 'APA_bin_14': 'Gemmatimonadota', 'GCF_900109375': 'Bacteroidota', 'seawater_bettina_36309_bin_17': 'Proteobacteria', 'IRC_PAM_SB0668_bin_6': 'Actinobacteriota', 'seawater_22112_bin_2': 'Actinobacteriota', 'seawater_bettina_36327_bin_6': 'Bacteroidota', 'COS36388_bin_1': 'Acidobacteriota', 'IRC3_bin_7': 'Proteobacteria', 'RHO2_bin_65': 'Spirochaetota', 'IRC_PAM_SB0662_bin_23': 'Gemmatimonadota', 'RHO3_bin_51': 'Poribacteria', 'RHO1_bin_70': 'Gemmatimonadota', 'IRC_PAM_SB0662_bin_15': 'Latescibacterota', 'seawater_22112_bin_14': 'Planctomycetota', 'APA_bin_73': 'Binatota', 'IRC_PAM_SB0666_bin_6': 'Chloroflexota', 'RHO2_bin_13': 'Poribacteria', 'COS1_bin_19': 'Chloroflexota', 'COS36386_bin_28': 'Binatota', 'COS36386_bin_31': 'Chloroflexota', 'RHO3_bin_79': 'Chloroflexota', 'seawater_bettina_36309_bin_3': 'Proteobacteria', 'IRC_PAM_SB0661_bin_24': 'Bacteroidota', 'RHO1_bin_68': 'Chloroflexota', 'APA_bin_48': 'Chloroflexota', 'IRC2_bin_2': 'Actinobacteriota', 'APA_bin_5': 'Chloroflexota', 'RHO1_bin_42': 'Acidobacteriota', 'IRC_PAM_SB0662_bin_20': 'Chloroflexota', 'RHO2_bin_17': 'Poribacteria', 'IRC3_bin_21': 'Poribacteria', 'IRC4_bin_23': 'Proteobacteria', 'RHO3_bin_66': 'Spirochaetota', 'IRC_PAM_SB0662_bin_11': 'Gemmatimonadota', 'RHO3_bin_54': 'Chloroflexota', 'IRC1_bin_20': 'Chloroflexota', 'CLI1_bin_2': 'Proteobacteria', 'IRC_PAM_SB0661_bin_44': 'Chloroflexota', 'IRC_PAM_SB0662_bin_59': 'Proteobacteria', 'RHO1_bin_6': 'Proteobacteria', 'IRC_PAM_SB0661_bin_27': 'Latescibacterota', 'seawater_seasim_SB9155_S4_bin_5': 'Gemmatimonadota', 'IRC_PAM_SB0661_bin_40': 'Gemmatimonadota', 'seawater_seasim_SB9158_S7_bin_9': 'Proteobacteria', 'IRC_PAM_SB0678_bin_7': 'Proteobacteria', 'seawater_22112_bin_25': 'Verrucomicrobiota', 'COS36388_bin_18': 'Actinobacteriota', 'IRC4_bin_40': 'Acidobacteriota', 'IRC4_bin_25': 'Poribacteria', 'CYMC_moitinhoThomas_67496': 'Crenarchaeota', 'APA_bin_69': 'Proteobacteria', 'GCA_001541925': 'Crenarchaeota', 'IRC_PAM_SB0662_bin_37': 'Latescibacterota', 'APA_bin_62': 'Spirochaetota', 'GCF_900149695': 'Proteobacteria', 'RHO1_bin_15': 'Chloroflexota', 'COS1_bin_6': 'Actinobacteriota', 'IRC_PAM_SB0661_bin_38': 'Acidobacteriota', 'COS36405_bin_11': 'Proteobacteria', 'APA_bin_28': 'Acidobacteriota', 'seawater_22112_bin_4': 'Proteobacteria', 'CAR4_bin_4': 'Actinobacteriota', 'IRC4_bin_4': 'Bacteroidota', 'RHO3_bin_74': 'Chloroflexota', 'IRC_PAM_SB0670_bin_12': 'Dadabacteria', 'IRC_PAM_SB0662_bin_5': 'Chloroflexota', 'SPOO_karimiCosta_FZLS01': 'Proteobacteria', 'RHO3_bin_39': 'Deinococcota', 'IRC3_bin_4': 'Acidobacteriota', 'RHO1_bin_58': 'Proteobacteria', 'IRC2_bin_1': 'Bacteroidota', 'IRC4_bin_29': 'Bacteroidota', 'seawater_seasim_SB9152_S1_bin_3': 'Proteobacteria', 'APA_bin_81': 'Bacteroidota', 'APA_bin_93': 'Proteobacteria', 'RHO3_bin_11': 'Proteobacteria', 'RHO2_bin_12': 'Proteobacteria', 'RHO2_bin_9': 'Chloroflexota', 'APA_bin_70': 'Proteobacteria', 'RHO1_bin_18': 'Chloroflexota', 'APA_bin_15': 'Chloroflexota', 'seawater_seasim_SB9157_S6_bin_2': 'Marinisomatota', 'IRC_PAM_SB0662_bin_9': 'Chloroflexota', 'seawater_bettina_36328_bin_30': 'Proteobacteria', 'APA_bin_26': 'Bacteroidota', 'COS2_bin_16': 'Chloroflexota', 'COS36404_bin_6': 'Proteobacteria', 'IRC1_bin_16': 'Latescibacterota', 'IRC1_bin_34': 'Actinobacteriota', 'GCF_900149685': 'Proteobacteria', 'COS36404_bin_12': 'Planctomycetota', 'IRC2_bin_3': 'Chloroflexota', 'STY2_bin_6': 'Proteobacteria', 'seawater_seasim_SB9154_S3_bin_4': 'Gemmatimonadota', 'APA_bin_17': 'Actinobacteriota', 'IRC4_bin_38': 'Actinobacteriota', 'IRC_PAM_SB0677_bin_7': 'Proteobacteria', 'IRC_PAM_SB0665_bin_9': 'Acidobacteriota', 'COS2_bin_23': 'Acidobacteriota', 'seawater_seasim_SB9154_S3_bin_1': 'Proteobacteria', 'IRC4_bin_37': 'Actinobacteriota', 'seawater_seasim_SB9160_S9_bin_9': 'Verrucomicrobiota', 'IRC4_bin_13': 'Gemmatimonadota', 'RHO1_bin_43': 'Proteobacteria', 'CLI4_bin_2': 'Dadabacteria', 'RHO1_bin_46': 'Bacteroidota', 'APA_bin_27': 'Proteobacteria', 'APA_bin_13': 'Chloroflexota', 'COS2_bin_4': 'Chloroflexota', 'IRC1_bin_35': 'Actinobacteriota', 'COS36387_bin_19': 'Chloroflexota', 'IRC3_bin_19': 'Gemmatimonadota', 'seawater_bettina_36328_bin_8': 'Proteobacteria', 'COS4_bin_32': 'Chloroflexota', 'COS2_bin_22': 'Chloroflexota', 'COS4_bin_4': 'Proteobacteria', 'RHO2_bin_41': 'Chloroflexota', 'COS1_bin_1': 'Chloroflexota', 'IRC_PAM_SB0664_bin_5': 'Proteobacteria', 'APA_bin_82': 'Proteobacteria', 'RHO1_bin_12': 'Acidobacteriota', 'IRC2_bin_8': 'Chloroflexota', 'IRC3_bin_16': 'Acidobacteriota', 'COS2_bin_2': 'Actinobacteriota', 'IRC_PAM_SB0665_bin_5': 'Actinobacteriota', 'CAR1_bin_4': 'Proteobacteria', 'APA_bin_23': 'Acidobacteriota', 'GCF_900143525': 'Proteobacteria', 'IRC3_bin_5': 'Proteobacteria', 'IRC_PAM_SB0675_bin_4': 'Actinobacteriota', 'seawater_22112_bin_28': 'Proteobacteria', 'COS2_bin_8': 'Actinobacteriota', 'IRC_PAM_SB0670_bin_39': 'Acidobacteriota', 'IRC3_bin_17': 'Actinobacteriota', 'IRC_PAM_SB0668_bin_21': 'Chloroflexota', 'COS4_bin_27': 'Acidobacteriota', 'seawater_42618_bin_6': 'Actinobacteriota', 'RHO3_bin_53': 'Chloroflexota', 'IRC_PAM_SB0661_bin_30': 'Acidobacteriota', 'CAR1_bin_7': 'Proteobacteria', 'IRC4_bin_17': 'Chloroflexota', 'STY1_bin_7': 'Cyanobacteria', 'IRC_PAM_SB0666_bin_34': 'Gemmatimonadota', 'IRC4_bin_14': 'Poribacteria', 'IRC_PAM_SB0661_bin_37': 'Actinobacteriota', 'IRC_PAM_SB0668_bin_12': 'Gemmatimonadota', 'STY1_bin_6': 'Proteobacteria', 'IRC_PAM_SB0665_bin_16': 'Gemmatimonadota', 'STY1_bin_2': 'Proteobacteria', 'STY4_bin_9': 'Cyanobacteria', 'RHO1_bin_10': 'Acidobacteriota', 'CAR2_bin_13': 'Actinobacteriota', 'RHO3_bin_34': 'Chloroflexota', 'CAR3_bin_3': 'Actinobacteriota', 'IRC3_bin_14': 'Actinobacteriota', 'COS36387_bin_2': 'Chloroflexota', 'IRC_PAM_SB0664_bin_3': 'Acidobacteriota', 'IRC_PAM_SB0661_bin_41': 'Gemmatimonadota', 'RHO3_bin_16': 'Acidobacteriota', 'COS36386_bin_29': 'Cyanobacteria', 'IRC_PAM_SB0675_bin_22': 'Poribacteria', 'RHO1_bin_27': 'Deinococcota', 'seawater_42618_bin_25': 'Proteobacteria', 'COS36386_bin_18': 'Chloroflexota', 'CAR2_bin_5': 'Proteobacteria', 'CAR1_bin_13': 'Proteobacteria', 'IRC_PAM_SB0661_bin_32': 'Chloroflexota', 'CAR4_bin_14': 'Chloroflexota', 'COS4_bin_44': 'Proteobacteria', 'COS1_bin_15': 'Proteobacteria', 'RHO3_bin_27': 'Acidobacteriota', 'RHO1_bin_1': 'Proteobacteria', 'COS36387_bin_20': 'Chloroflexota', 'COS36405_bin_3': 'Acidobacteriota', 'RHO1_bin_13': 'Bacteroidota', 'CAR2_bin_3': 'Chloroflexota', 'APA_bin_85': 'Gemmatimonadota', 'COS36387_bin_10': 'Actinobacteriota', 'COS36386_bin_11': 'Planctomycetota', 'IRC1_bin_3': 'Dadabacteria', 'COS1_bin_7': 'Nitrospirota', 'CLI2_bin_1': 'Proteobacteria', 'RHO2_bin_49': 'Gemmatimonadota', 'IRC_PAM_SB0667_bin_1': 'Acidobacteriota', 'IRC_PAM_SB0675_bin_1': 'Proteobacteria', 'COS36404_bin_3': 'Chloroflexota', 'IRC_PAM_SB0661_bin_2': 'Gemmatimonadota', 'SPOO_karimiCosta_FZLR01': 'Proteobacteria', 'COS3_bin_3': 'Chloroflexota', 'IRC_PAM_SB0668_bin_1': 'Bacteroidota', 'STY4_bin_4': 'Proteobacteria', 'IRC4_bin_30': 'Chloroflexota', 'RHO1_bin_65': 'Actinobacteriota', 'IRC_PAM_SB0664_bin_7': 'Bacteroidota', 'CAR3_bin_15': 'Proteobacteria', 'COS36405_bin_5': 'Planctomycetota', 'IRC_PAM_SB0665_bin_10': 'Proteobacteria', 'seawater_bettina_36328_bin_7': 'Proteobacteria', 'PER4_bin_1': 'Cyanobacteria', 'STY4_bin_2': 'Nitrospirota', 'COS36388_bin_11': 'Chloroflexota', 'RHO2_bin_51': 'Gemmatimonadota', 'RHO3_bin_47': 'Gemmatimonadota', 'COS4_bin_8': 'Chloroflexota', 'RHO1_bin_22': 'Actinobacteriota', 'IRC_PAM_SB0667_bin_2': 'Actinobacteriota', 'GCA_003635255': 'Poribacteria', 'RHO1_bin_52': 'Proteobacteria', 'RHO2_bin_62': 'Proteobacteria', 'IRC_PAM_SB0661_bin_3': 'Acidobacteriota', 'RHO3_bin_5': 'Bacteroidota', 'RHO1_bin_55': 'Gemmatimonadota', 'COS4_bin_2': 'Gemmatimonadota', 'COS4_bin_11': 'Proteobacteria', 'APA_bin_10': 'Actinobacteriota', 'RHO3_bin_26': 'Chloroflexota', 'RHO3_bin_43': 'Crenarchaeota', 'COS36386_bin_5': 'Acidobacteriota', 'IRC_PAM_SB0675_bin_10': 'Proteobacteria', 'RHO2_bin_18': 'Chloroflexota', 'GCF_900143535': 'Proteobacteria', 'IRC_PAM_SB0662_bin_30': 'Actinobacteriota', 'RHO2_bin_22': 'Chloroflexota', 'RHO3_bin_2': 'Chloroflexota', 'seawater_22112_bin_17': 'Planctomycetota', 'COS4_bin_40': 'Chloroflexota', 'seawater_22112_bin_7': 'Bacteroidota', 'COS4_bin_15': 'Proteobacteria', 'IRC1_bin_7': 'Proteobacteria', 'STY4_bin_1': 'Proteobacteria', 'CAR2_bin_14': 'Proteobacteria', 'COS36386_bin_32': 'Chloroflexota', 'CAR2_bin_15': 'Bacteroidota', 'IRC_PAM_SB0665_bin_11': 'Chloroflexota', 'seawater_bettina_36327_bin_3': 'Proteobacteria', 'RHO1_bin_9': 'Chloroflexota', 'STY4_bin_8': 'Proteobacteria', 'APA_bin_7': 'Actinobacteriota', 'IRC_PAM_SB0677_bin_8': 'Bacteroidota', 'IRC_PAM_SB0662_bin_10': 'Chloroflexota', 'Aquimarina_spAU58': 'Bacteroidota', 'RHO2_bin_57': 'Actinobacteriota', 'RHO2_bin_64': 'Spirochaetota', 'IRC4_bin_11': 'Chloroflexota', 'CAR4_bin_13': 'Bacteroidota', 'IRC_PAM_SB0666_bin_9': 'Actinobacteriota', 'IRC_PAM_SB0676_bin_21': 'Acidobacteriota', 'RHO3_bin_48': 'Gemmatimonadota', 'seawater_seasim_SB9156_S5_bin_3': 'Proteobacteria', 'IRC_PAM_SB0663_bin_5': 'Crenarchaeota', 'RHO3_bin_8': 'Chloroflexota', 'GCA_002007405': 'Proteobacteria', 'seawater_seasim_SB9153_S2_bin_13': 'Myxococcota', 'IRC_PAM_SB0666_bin_22': 'Acidobacteriota', 'RHO2_bin_7': 'Poribacteria', 'COS36388_bin_16': 'Chloroflexota', 'seawater_22112_bin_16': 'Proteobacteria', 'RHO3_bin_70': 'Poribacteria', 'IRC_PAM_SB0670_bin_18': 'Gemmatimonadota', 'RHO2_bin_15': 'Latescibacterota', 'IRC4_bin_16': 'Actinobacteriota', 'seawater_seasim_SB9157_S6_bin_7': 'Proteobacteria', 'IRC_PAM_SB0668_bin_19': 'Actinobacteriota', 'RHO3_bin_41': 'Proteobacteria', 'IRC_PAM_SB0661_bin_17': 'Chloroflexota', 'STY1_bin_3': 'Nitrospirota', 'RHO1_bin_26': 'Chloroflexota', 'COS36386_bin_19': 'Crenarchaeota', 'APA_bin_53': 'Actinobacteriota', 'IRC_PAM_SB0662_bin_33': 'Crenarchaeota', 'CAR1_bin_10': 'Proteobacteria', 'IRC_PAM_SB0677_bin_1': 'Proteobacteria', 'CAR2_bin_11': 'Proteobacteria', 'GCA_002631715': 'Proteobacteria', 'APA_bin_72': 'Chloroflexota', 'IRC_PAM_SB0665_bin_27': 'Proteobacteria', 'COS36386_bin_12': 'Acidobacteriota', 'RHO3_bin_17': 'Bdellovibrionota', 'COS36386_bin_14': 'Actinobacteriota', 'RHO2_bin_27': 'Acidobacteriota', 'IRC1_bin_18': 'Chloroflexota', 'CLI4_bin_1': 'Verrucomicrobiota_A', 'CAR4_bin_10': 'Bacteroidota', 'STY3_bin_3': 'Nitrospirota', 'CLI3_bin_1': 'Dadabacteria', 'IRC_PAM_SB0664_bin_28': 'Poribacteria', 'RHO1_bin_31': 'Proteobacteria', 'RHO2_bin_24': 'Actinobacteriota', 'CAR2_bin_19': 'Actinobacteriota', 'seawater_seasim_SB9157_S6_bin_13': 'Proteobacteria', 'RHO1_bin_44': 'Spirochaetota', 'IRC2_bin_7': 'Actinobacteriota', 'COS4_bin_5': 'Nitrospirota', 'COS2_bin_17': 'Chloroflexota', 'Aquimarina_spAU119': 'Bacteroidota', 'COS4_bin_26': 'Proteobacteria', 'COS36404_bin_8': 'Actinobacteriota', 'IRC_PAM_SB0666_bin_21': 'Chloroflexota', 'IRC_PAM_SB0661_bin_34': 'Chloroflexota', 'CHO1_bin_2': 'Bacteroidota', 'IRC_PAM_SB0665_bin_4': 'Actinobacteriota', 'APA_bin_39': 'Gemmatimonadota', 'CAR1_bin_9': 'Actinobacteriota', 'RHO1_bin_11': 'Poribacteria', 'RHO2_bin_53': 'Gemmatimonadota', 'COS36406_bin_1': 'Chloroflexota', 'COS36387_bin_3': 'Acidobacteriota', 'IRC_PAM_SB0666_bin_13': 'Proteobacteria', 'APA_bin_61': 'Proteobacteria', 'seawater_22112_bin_10': 'Proteobacteria', 'IRC2_bin_11': 'Gemmatimonadota', 'COS1_bin_16': 'Acidobacteriota', 'RHO1_bin_64': 'Actinobacteriota', 'COS36405_bin_10': 'Acidobacteriota', 'COS36386_bin_8': 'Proteobacteria', 'COS36387_bin_1': 'Nitrospirota', 'GCF_900143555': 'Proteobacteria', 'seawater_42618_bin_9': 'Proteobacteria', 'RHO2_bin_1': 'Proteobacteria', 'RHO3_bin_9': 'Proteobacteria', 'GCF_000264395': 'Firmicutes', 'IRC_PAM_SB0661_bin_19': 'Chloroflexota', 'COS36388_bin_3': 'Nitrospirota', 'IRC_PAM_SB0670_bin_20': 'Cyanobacteria', 'COS2_bin_19': 'Chloroflexota', 'RHO3_bin_52': 'Chloroflexota', 'RHO2_bin_43': 'Chloroflexota', 'APA_bin_42': 'Chloroflexota', 'COS1_bin_5': 'Actinobacteriota', 'CAR3_bin_13': 'Proteobacteria', 'GCA_001543015': 'Crenarchaeota', 'IRC_PAM_SB0662_bin_34': 'Chloroflexota', 'COS4_bin_34': 'Actinobacteriota', 'COS2_bin_18': 'Chloroflexota', 'IRC2_bin_12': 'Gemmatimonadota', 'IRC3_bin_26': 'Actinobacteriota', 'IRC_PAM_SB0662_bin_26': 'Nitrospirota', 'IRC4_bin_3': 'Dadabacteria', 'RHO2_bin_39': 'Actinobacteriota', 'seawater_42618_bin_2': 'Planctomycetota', 'STY4_bin_3': 'Proteobacteria', 'IRC1_bin_9': 'Actinobacteriota', 'IRC_PAM_SB0662_bin_28': 'Chloroflexota', 'COS36406_bin_7': 'Proteobacteria', 'IRC_PAM_SB0661_bin_43': 'Chloroflexota', 'RHO2_bin_30': 'Latescibacterota', 'IRC3_bin_12': 'Actinobacteriota', 'IRC2_bin_6': 'Chloroflexota', 'RHO1_bin_56': 'Gemmatimonadota', 'COS36387_bin_16': 'Chloroflexota', 'IRC_PAM_SB0668_bin_27': 'Chloroflexota', 'COS36405_bin_8': 'Actinobacteriota', 'IRC_PAM_SB0661_bin_15': 'Chloroflexota', 'COS36404_bin_5': 'Nitrospirota', 'IRC3_bin_37': 'Gemmatimonadota', 'GCF_003676335': 'Proteobacteria', 'CAR3_bin_10': 'Bacteroidota', 'IRC_PAM_SB0668_bin_5': 'Actinobacteriota', 'APA_bin_74': 'Actinobacteriota', 'RHO1_bin_30': 'Poribacteria', 'IRC4_bin_24': 'Actinobacteriota', 'IRC4_bin_41': 'Proteobacteria', 'seawater_22112_bin_6': 'Proteobacteria', 'COS36404_bin_1': 'Planctomycetota', 'seawater_22112_bin_12': 'Proteobacteria', 'RHO1_bin_8': 'Chloroflexota', 'RHO3_bin_65': 'Binatota', 'IRC3_bin_8': 'Proteobacteria', 'IRC4_bin_6': 'Cyanobacteria', 'IRC1_bin_10': 'Chloroflexota', 'CAR1_bin_5': 'Bacteroidota', 'IRC_PAM_SB0675_bin_23': 'Nitrospirota', 'COS36386_bin_35': 'UBA8248', 'seawater_bettina_36308_bin_18': 'Proteobacteria', 'IRC_PAM_SB0668_bin_7': 'Actinobacteriota', 'GCA_001007625': 'Cyanobacteria', 'APA_bin_87': 'Proteobacteria', 'IRC_PAM_SB0678_bin_9': 'Bacteroidota', 'RHO2_bin_6': 'Chloroflexota', 'CAR3_bin_1': 'Proteobacteria', 'seawater_42617_bin_7': 'Bacteroidota', 'COS36388_bin_15': 'Planctomycetota', 'IRC_PAM_SB0661_bin_31': 'Gemmatimonadota', 'IRC_PAM_SB0666_bin_17': 'Dadabacteria', 'seawater_bettina_36327_bin_15': 'Bacteroidota', 'COS1_bin_17': 'Proteobacteria', 'IRC1_bin_14': 'Gemmatimonadota', 'COS1_bin_21': 'Chloroflexota', 'IRC1_bin_37': 'Actinobacteriota', 'APA_bin_41': 'Latescibacterota', 'CAR1_bin_3': 'Proteobacteria', 'RHO2_bin_40': 'Chloroflexota', 'IRC_PAM_SB0662_bin_36': 'Actinobacteriota', 'RHO3_bin_40': 'Acidobacteriota', 'COS4_bin_23': 'UBA8248', 'IRC_PAM_SB0664_bin_10': 'Gemmatimonadota', 'COS36387_bin_7': 'Planctomycetota', 'APA_bin_91': 'UBA8248', 'COS36404_bin_10': 'Acidobacteriota', 'IRC_PAM_SB0677_bin_14': 'Actinobacteriota', 'IRC_PAM_SB0662_bin_7': 'Proteobacteria', 'seawater_bettina_36328_bin_5': 'Planctomycetota', 'GCF_900143615': 'Proteobacteria', 'COS4_bin_20': 'Acidobacteriota', 'IRC1_bin_4': 'Bacteroidota', 'seawater_seasim_SB9156_S5_bin_20': 'Bacteroidota'}

taxon_to_group_id_dict = {'B': 'IRC3_bin_10', 'M': 'IRC1_bin_14', 'AI': 'RHO2_bin_50', 'N': 'IRC_PAM_SB0661_bin_30', 'U': 'IRC_PAM_SB0664_bin_33', 'W': 'IRC_PAM_SB0665_bin_11', 'X': 'IRC1_bin_9', 'AA': 'IRC_PAM_SB0675_bin_5', 'K': 'COS4_bin_6', 'AN': 'RHO3_bin_54', 'AP': 'RHO3_bin_58', 'AD': 'RHO1_bin_10', 'H': 'COS36386_bin_28', 'L': 'IRC_PAM_SB0672_bin_21', 'AK': 'RHO2_bin_55', 'AE': 'IRC4_bin_30', 'AC': 'IRC_PAM_SB0676_bin_21', 'D': 'IRC_PAM_SB0662_bin_37', 'Q': 'IRC_PAM_SB0661_bin_41', 'AL': 'RHO3_bin_38', 'S': 'IRC_PAM_SB0662_bin_15', 'Z': 'RHO1_bin_66', 'AH': 'RHO1_bin_53', 'AF': 'RHO1_bin_32', 'AO': 'APA_bin_73', 'A': 'APA_bin_1', 'AS': 'RHO3_bin_80', 'T': 'IRC_PAM_SB0661_bin_3', 'J': 'IRC_PAM_SB0662_bin_11', 'G': 'COS4_bin_23', 'R': 'IRC4_bin_12', 'V': 'COS4_bin_9', 'F': 'COS36387_bin_9', 'AR': 'RHO3_bin_73', 'P': 'IRC1_bin_19', 'I': 'COS4_bin_26', 'AJ': 'RHO3_bin_72', 'AT': 'RHO1_bin_82', 'C': 'APA_bin_94', 'AM': 'IRC_PAM_SB0668_bin_14', 'O': 'IRC3_bin_22', 'E': 'COS3_bin_9', 'Y': 'IRC_PAM_SB0672_bin_11', 'AG': 'COS3_bin_2', 'AB': 'COS1_bin_16', 'AQ': 'IRC_PAM_SB0675_bin_10'}

genome_to_taxon_dict = {'RHO3_bin_73': 'RHO3_bin_73', 'RHO1_bin_82': 'RHO1_bin_82', 'IRC1_bin_9': 'IRC1_bin_9', 'IRC_PAM_SB0675_bin_10': 'IRC_PAM_SB0675_bin_10', 'RHO3_bin_38': 'RHO3_bin_38', 'IRC_PAM_SB0662_bin_37': 'IRC_PAM_SB0662_bin_37', 'IRC_PAM_SB0661_bin_41': 'IRC_PAM_SB0661_bin_41', 'COS36387_bin_9': 'COS36387_bin_9', 'IRC4_bin_12': 'IRC4_bin_12', 'RHO3_bin_80': 'RHO3_bin_80', 'IRC_PAM_SB0676_bin_21': 'IRC_PAM_SB0676_bin_21', 'COS4_bin_6': 'COS4_bin_6', 'IRC_PAM_SB0668_bin_14': 'IRC_PAM_SB0668_bin_14', 'APA_bin_94': 'APA_bin_94', 'IRC_PAM_SB0665_bin_11': 'IRC_PAM_SB0665_bin_11', 'COS4_bin_26': 'COS4_bin_26', 'COS3_bin_2': 'COS3_bin_2', 'IRC_PAM_SB0672_bin_11': 'IRC_PAM_SB0672_bin_11', 'IRC_PAM_SB0662_bin_15': 'IRC_PAM_SB0662_bin_15', 'IRC_PAM_SB0672_bin_21': 'IRC_PAM_SB0672_bin_21', 'COS3_bin_9': 'COS3_bin_9', 'IRC_PAM_SB0661_bin_30': 'IRC_PAM_SB0661_bin_30', 'COS4_bin_9': 'COS4_bin_9', 'COS36386_bin_28': 'COS36386_bin_28', 'RHO1_bin_32': 'RHO1_bin_32', 'RHO2_bin_50': 'RHO2_bin_50', 'IRC_PAM_SB0675_bin_5': 'IRC_PAM_SB0675_bin_5', 'COS1_bin_16': 'COS1_bin_16', 'IRC_PAM_SB0664_bin_33': 'IRC_PAM_SB0664_bin_33', 'RHO1_bin_10': 'RHO1_bin_10', 'IRC4_bin_30': 'IRC4_bin_30', 'RHO1_bin_53': 'RHO1_bin_53', 'RHO1_bin_66': 'RHO1_bin_66', 'APA_bin_1': 'APA_bin_1', 'COS4_bin_23': 'COS4_bin_23', 'RHO3_bin_54': 'RHO3_bin_54', 'IRC1_bin_19': 'IRC1_bin_19', 'IRC3_bin_10': 'IRC3_bin_10', 'RHO2_bin_55': 'RHO2_bin_55', 'IRC_PAM_SB0661_bin_3': 'IRC_PAM_SB0661_bin_3', 'RHO3_bin_58': 'RHO3_bin_58', 'IRC3_bin_22': 'IRC3_bin_22', 'APA_bin_73': 'APA_bin_73', 'IRC1_bin_14': 'IRC1_bin_14', 'IRC_PAM_SB0662_bin_11': 'IRC_PAM_SB0662_bin_11', 'RHO3_bin_72': 'RHO3_bin_72'}

multi_level_detection = True

Get_circlize_plot(multi_level_detection,

'Figure_5',

'HGT_subset_with_interested_functions_Figure_5.txt',

genome_to_taxon_dict,

'c')

cir_plot_matrix = 'Figure_5_cir_plot_matrix.csv'

cir_plot_matrix_with_phylum = 'Figure_5_cir_plot_matrix_with_phylum.csv'

cir_plot_matrix_with_phylum_handle = open(cir_plot_matrix_with_phylum, 'w')

n = 0

for line in open(cir_plot_matrix):

line_split = line.strip().split('\t')

if n == 0:

header_list = ['%s__%s' % (MAG_to_taxon_dict[i], i) for i in line_split]

cir_plot_matrix_with_phylum_handle.write('\t%s\n' % '\t'.join(header_list))

else:

cir_plot_matrix_with_phylum_handle.write('%s__%s' % (MAG_to_taxon_dict[line_split[0]], line))

n += 1

cir_plot_matrix_with_phylum_handle.close()

os.system('Rscript circos_HGT_Figure_5.R -m %s -p Figure_5.pdf -s __' % (cir_plot_matrix_with_phylum))

**End script Figure_5.py

Start R script circos_HGT_Figure_5.R**

group_list = c()

for (element in list_in_split){

group_list = c(group_list, element[1])

}

return(group_list)

}

get_taxon_list = function(list_in, sep_symbol){

list_in_split = strsplit(list_in, sep_symbol)

taxon_list = c()

for (element in list_in_split){

taxon_list = c(taxon_list, element[2])

}

return(taxon_list)

}

remove_zero_rows_cols = function(matrix_in){

remove_zero_rows = function(matrix_in){

row_sum = rowSums(matrix_in)

mat_with_row_sum = cbind(matrix_in, row_sum)

mat_non_zero_row_tmp = mat_with_row_sum[mat_with_row_sum$row_sum > 0, ]

mat_non_zero_row = subset(mat_non_zero_row_tmp, select = -c(row_sum))

return(mat_non_zero_row)

}

mat_non_zero_row = remove_zero_rows(mat)

mat_non_zero_row_t = as.data.frame(t(mat_non_zero_row))

mat_non_zero_row_col_t = remove_zero_rows(mat_non_zero_row_t)

mat_non_zero_row_col = t(mat_non_zero_row_col_t)

return(mat_non_zero_row_col)

}

####################################### install packages #######################################

packages<-c("optparse", "circlize")

invisible(suppressMessages(check.packages(packages)))

####################################### argument parser ######################################

options(warn=-1)

option_list = list(

make_option(c("-m", "--matrix"), type="character", help="input matrix"),

make_option(c("-s", "--sep"), type="character", help="label separator"),

make_option(c("-p", "--plot"), type="character", help="output plot"));

within_group_gap = 1

between_group_gap = 4

opt_parser = OptionParser(option_list=option_list);

opt = parse_args(opt_parser);

group_separator = opt$sep

# reads in file

mat = read.table(opt$matrix, header = TRUE)

######################################## prepare plot ########################################

#png(filename=opt$plot, units="in", width=25, height=25, pointsize=12, res=150)

#cairo_ps(filename=opt$plot, width=15, height=15, pointsize=12)

#svg(filename=opt$plot, width=15, height=15, pointsize=12)

pdf(opt$plot, width=15, height=15, pointsize=12)

grid.col = c(A = 'brown1', B = 'lawngreen', C = 'mediumorchid', D = 'mediumslateblue', E = 'royalblue', F = 'sandybrown')

par(mar = rep(0,4), cex = 1.2)

# set label_order

label_order = sort(union(rownames(mat), colnames(mat)))

############################### Set larger gaps between groups ###############################

# Set larger gaps between groups

label_order_on_plot = sort(union(rownames(remove_zero_rows_cols(mat)), colnames(remove_zero_rows_cols(mat))))

group_list_on_plot = get_group_list(label_order_on_plot, group_separator)

taxon_list_on_plot = get_taxon_list(label_order_on_plot, group_separator)

gap_between_group_list = c()

last_group = 'None'

for(group_id in group_list_on_plot){

if (last_group == 'None'){

last_group = group_id

} else if (group_id == last_group){

gap_between_group_list = c(gap_between_group_list, within_group_gap)

} else if (group_id != last_group){

gap_between_group_list = c(gap_between_group_list, between_group_gap)

last_group = group_id

}

}

if (group_list_on_plot[[1]] == group_list_on_plot[[length(group_list_on_plot)]]){

gap_between_group_list = c(gap_between_group_list, within_group_gap)

} else {

gap_between_group_list = c(gap_between_group_list, between_group_gap)

}

# Set larger gaps between groups

circos.par(gap.after = gap_between_group_list)

################################## set same color for same taxon ##################################

# color_key_for_protein_family

# GH33 #CCCC00

# CE7 #FF3333

# ELRS #9933FF

# Cadherin #66CC00

# Fibronectin #009999

# RMs Type I #004C99

# RMs Type II #0080FF

# RMs Type III #3399FF

# RMs Type IV #66B2FF

# RMs Not typed #99CCFF

# CRISPRS Cas1 #994C00

# CRISPRS Cas2 #CC6600

# CRISPRS Cas5 #FF8000

# CRISPRS Cas7 #FF9933

# CRISPRS Cas_GSU0053 #FFB266

# CRISPRS Csd1 #FFCC99

# set bin color

grid.col = c(Acidobacteriota__IRC_PAM_SB0672_bin_11 = '#9933FF',

Acidobacteriota__IRC_PAM_SB0676_bin_21 = '#009999',

Acidobacteriota__IRC_PAM_SB0661_bin_30 = 'grey90',

Gemmatimonadota__IRC_PAM_SB0672_bin_21 = '#9933FF',

Proteobacteria__IRC_PAM_SB0675_bin_10 = 'grey90',

Latescibacterota__COS4_bin_9 = 'grey90',

Gemmatimonadota__RHO3_bin_73 = '#009999',

Spirochaetota__APA_bin_94 = 'grey90',

Proteobacteria__RHO3_bin_58 = '#009999',

Acidobacteriota__RHO2_bin_55 = '#9933FF', # grey90

Chloroflexota__IRC3_bin_22 = '#66CC00',

Gemmatimonadota__RHO3_bin_72 = '#009999',

Acidobacteriota__COS1_bin_16 = 'grey90',

Acidobacteriota__IRC4_bin_12 = '#009999', # grey90 #9933FF

Chloroflexota__RHO3_bin_54 = '#9933FF',

Chloroflexota__RHO1_bin_32 = '#CCCC00',

Gemmatimonadota__IRC_PAM_SB0661_bin_41 = '#9933FF',

Chloroflexota__APA_bin_1 = 'grey90',

Gemmatimonadota__RHO1_bin_66 = 'grey90',

Chloroflexota__IRC3_bin_10 = '#9933FF',

Chloroflexota__IRC_PAM_SB0665_bin_11 = '#66CC00',

Gemmatimonadota__RHO3_bin_38 = 'grey90',

Gemmatimonadota__IRC1_bin_14 = '#009999',

Proteobacteria__RHO1_bin_82 = '#9933FF',

Acidobacteriota__IRC_PAM_SB0668_bin_14 = '#009999',

Acidobacteriota__RHO1_bin_10 = '#CCCC00',

Chloroflexota__IRC_PAM_SB0664_bin_33 = '#009999',

Chloroflexota__COS3_bin_2 = 'grey90',

Chloroflexota__COS36387_bin_9 = 'grey90',

Chloroflexota__IRC1_bin_19 = 'grey90',

UBA8248__COS4_bin_23 = '#9933FF',

Acidobacteriota__IRC_PAM_SB0675_bin_5 = '#FF3333',

Binatota__COS36386_bin_28 = 'grey90',

Gemmatimonadota__IRC_PAM_SB0662_bin_11 = 'grey90',

Latescibacterota__IRC_PAM_SB0662_bin_15 = '#9933FF',

Chloroflexota__IRC4_bin_30 = 'grey90',

Latescibacterota__IRC_PAM_SB0662_bin_37 = '#CCCC00',

Proteobacteria__COS4_bin_26 = '#009999',

Proteobacteria__RHO1_bin_53 = '#009999',

Chloroflexota__COS3_bin_9 = '#9933FF',

Acidobacteriota__IRC_PAM_SB0661_bin_3 = 'grey90',

Acidobacteriota__COS4_bin_6 = 'grey90',

Gemmatimonadota__RHO2_bin_50 = 'grey90',

Proteobacteria__RHO3_bin_80 = 'grey90',

Actinobacteriota__IRC1_bin_9 = 'grey90',

Binatota__APA_bin_73 = 'grey90')

taxon_list_on_plot_uniq = unique(taxon_list_on_plot)

track_color_list = rainbow(length(taxon_list_on_plot_uniq))

# get color for each taxon

n = 1

taxon_to_color_dict = list()

for (each_taxon in taxon_list_on_plot_uniq){

taxon_to_color_dict[each_taxon] = track_color_list[n]

n = n + 1

}

# set color for all labels

label_color = c()

for (each_label in label_order_on_plot){

each_label_split = strsplit(each_label, group_separator)

each_label_color = taxon_to_color_dict[each_label_split[[1]][2]][[1]]

label_color[each_label] = each_label_color

}

######################################## plot chordDiagram ########################################

# edit initialising parameters

circos.par(canvas.ylim=c(-1.5,1.5), # edit canvas size

track.margin = c(0.01, 0.05), # adjust bottom and top margin

# track.margin = c(0.01, 0.1)

track.height = 0.1)

# plot chordDiagram

chordDiagram(t(mat), order = label_order, annotationTrack = "grid", annotationTrackHeight = c(0.03, 0.01), preAllocateTracks = 1, grid.col = grid.col)

######################################## add group track ########################################

color_list = list(Acidobacteriota = '#1E90FF',

Binatota = '#CCCC00',

Chloroflexota = '#66CC00',

Chloroflexota_B = '#CCFFFF',

Gemmatimonadota = '#00CC66',

Latescibacterota = '#6666FF',

Proteobacteria = '#66FFFF',

UBA8248 = '#FFB266')

current_group = 'None'

current_group_member = c()

for (label in label_order_on_plot){

label_split = strsplit(label, group_separator)

label_group = label_split[[1]][1]

if (current_group == 'None'){

current_group = label_group

current_group_member = c(current_group_member, label)

} else if (label_group == current_group){

current_group_member = c(current_group_member, label)

} else if (label_group != current_group){

group_name = strsplit(current_group_member, group_separator)[[1]][1]

highlight.sector(current_group_member, track.index = 1, col = color_list[[group_name]], padding=c(0, 0, 0, 0), niceFacing = TRUE)

#highlight.sector(current_group_member, track.index = 1, text = group_name, facing = "bending.inside", niceFacing = TRUE, cex = 1.2) # cex controls font size

#PDF

highlight.sector(current_group_member, track.index = 1, text = group_name, facing = "bending.inside", niceFacing = TRUE, cex = 0.7) # cex controls font size

current_group = label_group

current_group_member = c(label)

}

}

group_name = strsplit(current_group_member, group_separator)[[1]][1]

highlight.sector(current_group_member, track.index = 1, track.height = 0.02, col = color_list[[group_name]], padding=c(0, 0, 0, 0), niceFacing = TRUE)

#highlight.sector(current_group_member, track.index = 1, text = group_name, facing = "bending.inside", niceFacing = TRUE, cex = 1.2) # cex controls font size

#pdf

highlight.sector(current_group_member, track.index = 1, text = group_name, facing = "bending.inside", niceFacing = TRUE, cex = 0.7) # cex controls font size

######################################## rotate label ########################################

circos.trackPlotRegion(track.index = 1, panel.fun = function(x, y) {

xlim = get.cell.meta.data("xlim")

ylim = get.cell.meta.data("ylim")

sector.name = get.cell.meta.data("sector.index")

label_text = strsplit(sector.name, group_separator)[[1]][2]

#circos.text(mean(xlim), ylim[1] + .1, sector.name, facing = "inside", niceFacing = TRUE, adj = c(0.5, 0), cex = 0.6 )

#circos.text(mean(xlim), ylim[1] + .1, label_text, font = par('bold'), facing = "bending.inside", niceFacing = TRUE, adj = c(0.5, 7), cex = 0.7 )

# EPS

#circos.text(mean(xlim), ylim[1] + .1, label_text, facing = "bending.inside", niceFacing = TRUE, adj = c(0.5, 6), cex = 0.45 )

#PDF

circos.text(mean(xlim), ylim[1] + .1, label_text, facing = "bending.inside", niceFacing = TRUE, adj = c(0.5, 10.5), cex = 0.3 )

#circos.axis(h = "top", labels.cex = 0.6, major.tick.percentage = 1, sector.index = sector.name, track.index = 2)

#PDF

circos.axis(h = "top", labels.cex = 0.3, major.tick.percentage = 1, minor.ticks = 0, sector.index = sector.name, track.index = 2)

}, bg.border = NA)

######################################## clear variables ########################################

#circos.track(circos.text, facing = "clockwise")

invisible(dev.off())

circos.clear()

rm(list=ls())

**End R script circos_HGT_Figure_5.R**

**Start python script name Figure_S8.py**

#!/usr/bin/env python3

import os

def Get_circlize_plot(pwd_candidates_file_PG_normal_txt, genome_to_taxon_dict, pwd_matrix_file, taxon_rank, bin_to_source2_dict, sponge_with_high_HGT_preferences_list):

pwd_cir_plot_t1 = 'cir_plot_t1.txt'

pwd_cir_plot_t1_sorted = 'cir_plot_t1_sorted.txt'

pwd_cir_plot_t1_sorted_count = 'cir_plot_t1_sorted_count.txt'

name2taxon_dict = {}

transfers = []

for each in open(pwd_candidates_file_PG_normal_txt):

if not each.startswith('Gene_1'):

each_split = each.strip().split('\t')

gene_1 = each_split[0]

gene_2 = each_split[1]

Genome_1 = '_'.join(gene_1.split('_')[:-1])

Genome_2 = '_'.join(gene_2.split('_')[:-1])

Genome_1_raw_name = new2raw_name_dict[Genome_1]

Genome_2_raw_name = new2raw_name_dict[Genome_2]

Genome_1_source2 = bin_to_source2_dict[Genome_1_raw_name]

Genome_2_source2 = bin_to_source2_dict[Genome_2_raw_name]

# get direction

direction = each_split[6]

if '%)' in direction:

direction = direction.split('(')[0]

if (Genome_1_source2 in sponge_with_high_HGT_preferences_list) and (Genome_2_source2 in sponge_with_high_HGT_preferences_list):

if Genome_1 in genome_to_taxon_dict:

Genome_1_taxon = genome_to_taxon_dict[Genome_1]

else:

Genome_1_taxon = '%s_' % taxon_rank

if Genome_2 in genome_to_taxon_dict:

Genome_2_taxon = genome_to_taxon_dict[Genome_2]

else:

Genome_2_taxon = '%s_' % taxon_rank

if Genome_1 not in name2taxon_dict:

name2taxon_dict[Genome_1] = Genome_1_taxon

if Genome_2 not in name2taxon_dict:

name2taxon_dict[Genome_2] = Genome_2_taxon

transfers.append(direction)

tmp1 = open(pwd_cir_plot_t1, 'w')

all_group_id = []

for each_t in transfers:

each_t_split = each_t.split('-->')

donor = each_t_split[0]

recipient = each_t_split[1]

donor_id = name2taxon_dict[donor]

recipient_id = name2taxon_dict[recipient]

if donor_id not in all_group_id:

all_group_id.append(donor_id)

if recipient_id not in all_group_id:

all_group_id.append(recipient_id)

tmp1.write('%s,%s\n' % (donor_id, recipient_id))

tmp1.close()

os.system('cat %s | sort > %s' % (pwd_cir_plot_t1, pwd_cir_plot_t1_sorted))

current_t = ''

count = 0

tmp2 = open(pwd_cir_plot_t1_sorted_count, 'w')

for each_t2 in open(pwd_cir_plot_t1_sorted):

each_t2 = each_t2.strip()

if current_t == '':

current_t = each_t2

count += 1

elif current_t == each_t2:

count += 1

elif current_t != each_t2:

tmp2.write('%s,%s\n' % (current_t, count))

current_t = each_t2

count = 1

tmp2.write('%s,%s\n' % (current_t, count))

tmp2.close()

# read in count as dict

transfer_count = {}

for each_3 in open(pwd_cir_plot_t1_sorted_count):

each_3_split = each_3.strip().split(',')

key = '%s,%s' % (each_3_split[0], each_3_split[1])

value = each_3_split[2]

transfer_count[key] = value

all_group_id = sorted(all_group_id)

matrix_file = open(pwd_matrix_file, 'w')

matrix_file.write('\t' + '\t'.join(all_group_id) + '\n')

for each_1 in all_group_id:

row = [each_1]

for each_2 in all_group_id:

current_key = '%s,%s' % (each_2, each_1)

if current_key not in transfer_count:

row.append('0')

else:

row.append(transfer_count[current_key])

matrix_file.write('\t'.join(row) + '\n')

matrix_file.close()

# rm tmp files

os.system('rm %s' % pwd_cir_plot_t1)

os.system('rm %s' % pwd_cir_plot_t1_sorted)

os.system('rm %s' % pwd_cir_plot_t1_sorted_count)

######################################################## dRep99 ########################################################

# file in

detected_HGTs_pcofg = 'MetaCHIP_detected_HGTs.txt'

########################################################################################################################

# read in name correlation

new2raw_name_dict = {'CAR4_bin_6': 'CAR4_bin_6', 'COS2_bin_4': 'COS2_bin_4', 'COS4_bin_21': 'COS4_bin_21', 'COS2_bin_10': 'COS2_bin_10', 'IRC4_bin_41': 'IRC4_bin_41', 'IRC_PAM_SB0675_bin_5': 'IRC_PAM_SB0675_bin_5', 'RHO2_bin_49': 'RHO2_bin_49', 'IRC_PAM_SB0665_bin_25': 'IRC_PAM_SB0665_bin_25', 'COS2_bin_7': 'COS2_bin_7', 'IRC_PAM_SB0667_bin_1': 'IRC_PAM_SB0667_bin_1', 'RHO3_bin_84': 'RHO3_bin_84', 'IRC3_bin_26': 'IRC3_bin_26', 'IRC3_bin_28': 'IRC3_bin_28', 'RHO1_bin_56': 'RHO1_bin_56', 'IRC_PAM_SB0668_bin_19': 'IRC_PAM_SB0668_bin_19', 'IRC_PAM_SB0666_bin_34': 'IRC_PAM_SB0666_bin_34', 'seawater_bettina_36328_bin_3': 'seawater_bettina_36328_bin_3', 'COS36405_bin_3': 'COS36405_bin_3', 'RHO1_bin_37': 'RHO1_bin_37', 'IRC_PAM_SB0661_bin_2': 'IRC_PAM_SB0661_bin_2', 'RHO1_bin_65': 'RHO1_bin_65', 'IRC_PAM_SB0665_bin_4': 'IRC_PAM_SB0665_bin_4', 'IRC3_bin_18': 'IRC3_bin_18', 'RHO1_bin_53': 'RHO1_bin_53', 'COS3_bin_9': 'COS3_bin_9', 'RHO2_bin_42': 'RHO2_bin_42', 'CAR1_bin_10': 'CAR1_bin_10', 'IRC_PAM_SB0661_bin_17': 'IRC_PAM_SB0661_bin_17', 'IRC4_bin_3': 'IRC4_bin_3', 'COS36388_bin_13': 'COS36388_bin_13', 'APA_bin_59': 'APA_bin_59', 'APA_bin_42': 'APA_bin_42', 'IRC_PAM_SB0665_bin_27': 'IRC_PAM_SB0665_bin_27', 'CAR2_bin_5': 'CAR2_bin_5', 'seawater_bettina_36310_bin_3': 'seawater_bettina_36310_bin_3', 'Aquimarina_spAU474': 'TED_estevesThomas2016_Aquimarina_spAU474_2606217188', 'IRC4_bin_29': 'IRC4_bin_29', 'IRC_PAM_SB0661_bin_5': 'IRC_PAM_SB0661_bin_5', 'COS36387_bin_19': 'COS36387_bin_19', 'IRC_PAM_SB0661_bin_22': 'IRC_PAM_SB0661_bin_22', 'seawater_22112_bin_25': 'seawater_22112_bin_25', 'RHO3_bin_32': 'RHO3_bin_32', 'IRC_PAM_SB0665_bin_9': 'IRC_PAM_SB0665_bin_9', 'IRC1_bin_14': 'IRC1_bin_14', 'CAR2_bin_13': 'CAR2_bin_13', 'COS36388_bin_11': 'COS36388_bin_11', 'IRC_PAM_SB0661_bin_16': 'IRC_PAM_SB0661_bin_16', 'APA_bin_13': 'APA_bin_13', 'IRC_PAM_SB0677_bin_19': 'IRC_PAM_SB0677_bin_19', 'COS36386_bin_13': 'COS36386_bin_13', 'RHO3_bin_28': 'RHO3_bin_28', 'APA_bin_102': 'APA_bin_102', 'CAR1_bin_9': 'CAR1_bin_9', 'IRC4_bin_33': 'IRC4_bin_33', 'APA_bin_91': 'APA_bin_91', 'COS4_bin_42': 'COS4_bin_42', 'COS1_bin_11': 'COS1_bin_11', 'APA_bin_52': 'APA_bin_52', 'COS36386_bin_2': 'COS36386_bin_2', 'APA_bin_68': 'APA_bin_68', 'RHO3_bin_59': 'RHO3_bin_59', 'RHO1_bin_25': 'RHO1_bin_25', 'CLI1_bin_3': 'CLI1_bin_3', 'IRC1_bin_27': 'IRC1_bin_27', 'STY1_bin_6': 'STY1_bin_6', 'IRC_PAM_SB0675_bin_4': 'IRC_PAM_SB0675_bin_4', 'COS4_bin_7': 'COS4_bin_7', 'APA_bin_93': 'APA_bin_93', 'RHO1_bin_13': 'RHO1_bin_13', 'IRC_PAM_SB0677_bin_15': 'IRC_PAM_SB0677_bin_15', 'STY3_bin_6': 'STY3_bin_6', 'GCA_003635255': 'PSE_podellAllen_GCA_003635255.1_ASM363525v1_genomic', 'APA_bin_5': 'APA_bin_5', 'IRC_PAM_SB0673_bin_10': 'IRC_PAM_SB0673_bin_10', 'seawater_bettina_36309_bin_5': 'seawater_bettina_36309_bin_5', 'IRC_PAM_SB0678_bin_5': 'IRC_PAM_SB0678_bin_5', 'COS36404_bin_12': 'COS36404_bin_12', 'IRC_PAM_SB0677_bin_1': 'IRC_PAM_SB0677_bin_1', 'COS36387_bin_20': 'COS36387_bin_20', 'CAR2_bin_10': 'CAR2_bin_10', 'IRC_PAM_SB0662_bin_27': 'IRC_PAM_SB0662_bin_27', 'IRC1_bin_21': 'IRC1_bin_21', 'IRC1_bin_34': 'IRC1_bin_34', 'IRC_PAM_SB0678_bin_7': 'IRC_PAM_SB0678_bin_7', 'STY1_bin_5': 'STY1_bin_5', 'IRC_PAM_SB0675_bin_1': 'IRC_PAM_SB0675_bin_1', 'RHO3_bin_71': 'RHO3_bin_71', 'IRC4_bin_7': 'IRC4_bin_7', 'IRC_PAM_SB0677_bin_16': 'IRC_PAM_SB0677_bin_16', 'GCF_900149685': 'SPOO_karimiCosta2019_GCF_900149685.1_Erythrobacter_sp._Alg231_14_genomic', 'IRC2_bin_10': 'IRC2_bin_10', 'seawater_22112_bin_29': 'seawater_22112_bin_29', 'COS36386_bin_32': 'COS36386_bin_32', 'seawater_seasim_SB9160_S9_bin_9': 'seawater_seasim_SB9160_S9_bin_9', 'seawater_bettina_36328_bin_8': 'seawater_bettina_36328_bin_8', 'seawater_seasim_SB9156_S5_bin_6': 'seawater_seasim_SB9156_S5_bin_6', 'IRC_PAM_SB0664_bin_4': 'IRC_PAM_SB0664_bin_4', 'RHO1_bin_23': 'RHO1_bin_23', 'COS36388_bin_15': 'COS36388_bin_15', 'GCA_001541925': 'SUB_tianQian_GCA_001541925.1_ASM154192v1_genomic', 'COS2_bin_3': 'COS2_bin_3', 'CHO1_bin_2': 'CHO1_bin_2', 'APA_bin_80': 'APA_bin_80', 'Aquimarina_spAU58': 'TED_estevesThomas2016_Aquimarina_spAU58_IMGid2606217182', 'COS2_bin_6': 'COS2_bin_6', 'CLI1_bin_1': 'CLI1_bin_1', 'seawater_42618_bin_29': 'seawater_42618_bin_29', 'COS3_bin_6': 'COS3_bin_6', 'seawater_seasim_SB9152_S1_bin_8': 'seawater_seasim_SB9152_S1_bin_8', 'seawater_bettina_36308_bin_5': 'seawater_bettina_36308_bin_5', 'IRC_PAM_SB0677_bin_11': 'IRC_PAM_SB0677_bin_11', 'RHO1_bin_18': 'RHO1_bin_18', 'RHO3_bin_40': 'RHO3_bin_40', 'IRC2_bin_3': 'IRC2_bin_3', 'IRC3_bin_13': 'IRC3_bin_13', 'RHO3_bin_36': 'RHO3_bin_36', 'COS3_bin_14': 'COS3_bin_14', 'IRC2_bin_11': 'IRC2_bin_11', 'seawater_22112_bin_6': 'seawater_22112_bin_6', 'RHO2_bin_67': 'RHO2_bin_67', 'COS1_bin_8': 'COS1_bin_8', 'CAR1_bin_1': 'CAR1_bin_1', 'IRC_PAM_SB0662_bin_7': 'IRC_PAM_SB0662_bin_7', 'IRC_PAM_SB0668_bin_1': 'IRC_PAM_SB0668_bin_1', 'IRC_PAM_SB0677_bin_7': 'IRC_PAM_SB0677_bin_7', 'seawater_22112_bin_7': 'seawater_22112_bin_7', 'COS1_bin_4': 'COS1_bin_4', 'APA_bin_83': 'APA_bin_83', 'seawater_42617_bin_7': 'seawater_42617_bin_7', 'seawater_42618_bin_3': 'seawater_42618_bin_3', 'GCA_002631715': 'THES_lavyIlan_GCA_002631715.1_ASM263171v1_genomic', 'IRC2_bin_5': 'IRC2_bin_5', 'RHO2_bin_36': 'RHO2_bin_36', 'COS36386_bin_1': 'COS36386_bin_1', 'APA_bin_27': 'APA_bin_27', 'CAR2_bin_16': 'CAR2_bin_16', 'COS2_bin_19': 'COS2_bin_19', 'seawater_22112_bin_16': 'seawater_22112_bin_16', 'RHO1_bin_3': 'RHO1_bin_3', 'RHO3_bin_65': 'RHO3_bin_65', 'RHO3_bin_67': 'RHO3_bin_67', 'IRC4_bin_14': 'IRC4_bin_14', 'CLI3_bin_1': 'CLI3_bin_1', 'RHO3_bin_68': 'RHO3_bin_68', 'CAR2_bin_3': 'CAR2_bin_3', 'COS4_bin_6': 'COS4_bin_6', 'IRC1_bin_13': 'IRC1_bin_13', 'CAR2_bin_18': 'CAR2_bin_18', 'GCA_003635205': 'PSE_podellAllen_GCA_003635205.1_ASM363520v1_genomic', 'COS36388_bin_6': 'COS36388_bin_6', 'seawater_bettina_36308_bin_3': 'seawater_bettina_36308_bin_3', 'CAR2_bin_15': 'CAR2_bin_15', 'CLI2_bin_1': 'CLI2_bin_1', 'CAR4_bin_5': 'CAR4_bin_5', 'seawater_22112_bin_15': 'seawater_22112_bin_15', 'IRC4_bin_10': 'IRC4_bin_10', 'RHO3_bin_53': 'RHO3_bin_53', 'GCA_003635265': 'PSE_podellAllen_GCA_003635265.1_ASM363526v1_genomic', 'seawater_seasim_SB9152_S1_bin_3': 'seawater_seasim_SB9152_S1_bin_3', 'IRC_PAM_SB0665_bin_24': 'IRC_PAM_SB0665_bin_24', 'GCA_003635305': 'AGET_podellAllen_GCA_003635305.1_ASM363530v1_genomic', 'IRC_PAM_SB0662_bin_9': 'IRC_PAM_SB0662_bin_9', 'IRC_PAM_SB0664_bin_21': 'IRC_PAM_SB0664_bin_21', 'CAR3_bin_12': 'CAR3_bin_12', 'IRC4_bin_38': 'IRC4_bin_38', 'CAR4_bin_3': 'CAR4_bin_3', 'RHO3_bin_34': 'RHO3_bin_34', 'COS36387_bin_4': 'COS36387_bin_4', 'IRC1_bin_12': 'IRC1_bin_12', 'COS4_bin_40': 'COS4_bin_40', 'STY4_bin_1': 'STY4_bin_1', 'RHO1_bin_19': 'RHO1_bin_19', 'COS4_bin_24': 'COS4_bin_24', 'COS36386_bin_17': 'COS36386_bin_17', 'IRC_PAM_SB0675_bin_10': 'IRC_PAM_SB0675_bin_10', 'APA_bin_73': 'APA_bin_73', 'RHO2_bin_1': 'RHO2_bin_1', 'COS36386_bin_27': 'COS36386_bin_27', 'COS3_bin_13': 'COS3_bin_13', 'IRC_PAM_SB0665_bin_10': 'IRC_PAM_SB0665_bin_10', 'RHO3_bin_13': 'RHO3_bin_13', 'IRC_PAM_SB0665_bin_13': 'IRC_PAM_SB0665_bin_13', 'APA_bin_24': 'APA_bin_24', 'RHO3_bin_64': 'RHO3_bin_64', 'Ruegeria_spAU67': 'TED_estevesThomas2016_Ruegeria_spAU67_2606217183', 'IRC_PAM_SB0668_bin_6': 'IRC_PAM_SB0668_bin_6', 'RHO1_bin_62': 'RHO1_bin_62', 'IRC_PAM_SB0661_bin_45': 'IRC_PAM_SB0661_bin_45', 'IRC_PAM_SB0662_bin_26': 'IRC_PAM_SB0662_bin_26', 'COS1_bin_19': 'COS1_bin_19', 'RHO3_bin_11': 'RHO3_bin_11', 'RHO1_bin_46': 'RHO1_bin_46', 'RHO1_bin_14': 'RHO1_bin_14', 'IRC_PAM_SB0664_bin_31': 'IRC_PAM_SB0664_bin_31', 'IRC2_bin_6': 'IRC2_bin_6', 'COS1_bin_17': 'COS1_bin_17', 'IRC3_bin_19': 'IRC3_bin_19', 'GCF_900143525': 'SPOO_karimiCosta2019_GCF_900143525.1_Ruegeria_sp._Alg231_54_genomic', 'RHO1_bin_45': 'RHO1_bin_45', 'IRC_PAM_SB0664_bin_16': 'IRC_PAM_SB0664_bin_16', 'RHO2_bin_65': 'RHO2_bin_65', 'RHO2_bin_9': 'RHO2_bin_9', 'seawater_22112_bin_5': 'seawater_22112_bin_5', 'IRC_PAM_SB0670_bin_12': 'IRC_PAM_SB0670_bin_12', 'CAR4_bin_10': 'CAR4_bin_10', 'GCF_900109375': 'AMPF_kennedyDobson_GCF_900109375.1_IMGID2622736580_genomic', 'RHO3_bin_16': 'RHO3_bin_16', 'IRC_PAM_SB0677_bin_2': 'IRC_PAM_SB0677_bin_2', 'CYMC_moitinhoThomas_67496': 'CYMC_moitinhoThomas_67496.assembled', 'RHO1_bin_48': 'RHO1_bin_48', 'IRC_PAM_SB0665_bin_1': 'IRC_PAM_SB0665_bin_1', 'APA_bin_14': 'APA_bin_14', 'renamed': 'raw_name', 'COS4_bin_5': 'COS4_bin_5', 'IRC_PAM_SB0662_bin_20': 'IRC_PAM_SB0662_bin_20', 'COS1_bin_6': 'COS1_bin_6', 'IRC_PAM_SB0661_bin_4': 'IRC_PAM_SB0661_bin_4', 'RHO1_bin_1': 'RHO1_bin_1', 'GCF_900143535': 'SPOO_karimiCosta2019_GCF_900143535.1_Tateyamaria_sp._Alg231_49_genomic', 'RHO3_bin_31': 'RHO3_bin_31', 'RHO2_bin_51': 'RHO2_bin_51', 'seawater_bettina_36327_bin_2': 'seawater_bettina_36327_bin_2', 'IRC3_bin_31': 'IRC3_bin_31', 'IRC_PAM_SB0661_bin_37': 'IRC_PAM_SB0661_bin_37', 'aplysina_bin': 'APA_garciaTyalor_SAUL_aplysina_bin', 'CAR3_bin_6': 'CAR3_bin_6', 'COS36387_bin_6': 'COS36387_bin_6', 'RHO2_bin_37': 'RHO2_bin_37', 'COS4_bin_3': 'COS4_bin_3', 'COS3_bin_2': 'COS3_bin_2', 'CHO1_bin_1': 'CHO1_bin_1', 'COS4_bin_19': 'COS4_bin_19', 'IRC_PAM_SB0662_bin_33': 'IRC_PAM_SB0662_bin_33', 'IRC_PAM_SB0675_bin_19': 'IRC_PAM_SB0675_bin_19', 'COS36404_bin_1': 'COS36404_bin_1', 'RHO3_bin_58': 'RHO3_bin_58', 'CAR3_bin_1': 'CAR3_bin_1', 'CAR3_bin_7': 'CAR3_bin_7', 'IRC_PAM_SB0662_bin_5': 'IRC_PAM_SB0662_bin_5', 'IRC_PAM_SB0662_bin_22': 'IRC_PAM_SB0662_bin_22', 'APA_bin_26': 'APA_bin_26', 'CAR4_bin_18': 'CAR4_bin_18', 'COS36386_bin_19': 'COS36386_bin_19', 'IRC_PAM_SB0661_bin_41': 'IRC_PAM_SB0661_bin_41', 'IRC_PAM_SB0664_bin_25': 'IRC_PAM_SB0664_bin_25', 'IRC_PAM_SB0670_bin_20': 'IRC_PAM_SB0670_bin_20', 'APA_bin_81': 'APA_bin_81', 'IRC_PAM_SB0676_bin_21': 'IRC_PAM_SB0676_bin_21', 'COS4_bin_43': 'COS4_bin_43', 'RHO2_bin_18': 'RHO2_bin_18', 'APA_bin_33': 'APA_bin_33', 'CAR2_bin_20': 'CAR2_bin_20', 'seawater_bettina_36309_bin_17': 'seawater_bettina_36309_bin_17', 'CAR2_bin_2': 'CAR2_bin_2', 'IRC_PAM_SB0664_bin_27': 'IRC_PAM_SB0664_bin_27', 'RHO1_bin_85': 'RHO1_bin_85', 'seawater_bettina_36310_bin_4': 'seawater_bettina_36310_bin_4', 'COS1_bin_20': 'COS1_bin_20', 'IRC_PAM_SB0662_bin_58': 'IRC_PAM_SB0662_bin_58', 'CAR1_bin_12': 'CAR1_bin_12', 'seawater_22112_bin_10': 'seawater_22112_bin_10', 'CAR1_bin_3': 'CAR1_bin_3', 'IRC4_bin_8': 'IRC4_bin_8', 'IRC_PAM_SB0675_bin_2': 'IRC_PAM_SB0675_bin_2', 'IRC_PAM_SB0665_bin_2': 'IRC_PAM_SB0665_bin_2', 'IRC_PAM_SB0670_bin_22': 'IRC_PAM_SB0670_bin_22', 'IRC_PAM_SB0664_bin_22': 'IRC_PAM_SB0664_bin_22', 'seawater_seasim_SB9152_S1_bin_1': 'seawater_seasim_SB9152_S1_bin_1', 'RHO3_bin_69': 'RHO3_bin_69', 'seawater_seasim_SB9153_S2_bin_2': 'seawater_seasim_SB9153_S2_bin_2', 'IRC_PAM_SB0666_bin_17': 'IRC_PAM_SB0666_bin_17', 'IRC_PAM_SB0661_bin_3': 'IRC_PAM_SB0661_bin_3', 'IRC_PAM_SB0673_bin_16': 'IRC_PAM_SB0673_bin_16', 'IRC_PAM_SB0662_bin_35': 'IRC_PAM_SB0662_bin_35', 'RHO3_bin_35': 'RHO3_bin_35', 'COS2_bin_17': 'COS2_bin_17', 'IRC4_bin_23': 'IRC4_bin_23', 'RHO2_bin_30': 'RHO2_bin_30', 'IRC4_bin_24': 'IRC4_bin_24', 'seawater_22112_bin_28': 'seawater_22112_bin_28', 'CAR2_bin_7': 'CAR2_bin_7', 'APA_bin_94': 'APA_bin_94', 'APA_bin_85': 'APA_bin_85', 'IRC1_bin_17': 'IRC1_bin_17', 'COS36404_bin_9': 'COS36404_bin_9', 'COS36404_bin_2': 'COS36404_bin_2', 'CAR3_bin_16': 'CAR3_bin_16', 'IRC4_bin_44': 'IRC4_bin_44', 'seawater_22112_bin_3': 'seawater_22112_bin_3', 'CAR1_bin_11': 'CAR1_bin_11', 'APA_bin_17': 'APA_bin_17', 'RHO2_bin_59': 'RHO2_bin_59', 'seawater_seasim_SB9155_S4_bin_12': 'seawater_seasim_SB9155_S4_bin_12', 'GCF_001941685': 'AREB_froesThompson_GCF_001941685.1_ASM194168v1_genomic', 'IRC1_bin_35': 'IRC1_bin_35', 'RHO2_bin_33': 'RHO2_bin_33', 'IRC1_bin_15': 'IRC1_bin_15', 'RHO2_bin_54': 'RHO2_bin_54', 'RHO3_bin_7': 'RHO3_bin_7', 'COS4_bin_44': 'COS4_bin_44', 'petrosia_ficiformis_bin': 'PETF_garciaTaylor_SAUL_petrosia_ficiformis_bin', 'APA_bin_70': 'APA_bin_70', 'RHO2_bin_7': 'RHO2_bin_7', 'IRC_PAM_SB0664_bin_9': 'IRC_PAM_SB0664_bin_9', 'RHO3_bin_47': 'RHO3_bin_47', 'COS36387_bin_1': 'COS36387_bin_1', 'IRC3_bin_12': 'IRC3_bin_12', 'COS1_bin_12': 'COS1_bin_12', 'CAR4_bin_2': 'CAR4_bin_2', 'CAR4_bin_8': 'CAR4_bin_8', 'IRC_PAM_SB0666_bin_10': 'IRC_PAM_SB0666_bin_10', 'IRC_PAM_SB0662_bin_11': 'IRC_PAM_SB0662_bin_11', 'COS3_bin_18': 'COS3_bin_18', 'GCF_003676335': 'unknown_alexAntunes_GCF_003676335.1_ASM367633v1_genomic', 'IRC_PAM_SB0678_bin_9': 'IRC_PAM_SB0678_bin_9', 'RHO1_bin_4': 'RHO1_bin_4', 'RHO3_bin_72': 'RHO3_bin_72', 'RHO1_bin_67': 'RHO1_bin_67', 'RHO1_bin_44': 'RHO1_bin_44', 'IRC4_bin_2': 'IRC4_bin_2', 'IRC_PAM_SB0676_bin_26': 'IRC_PAM_SB0676_bin_26', 'CAR4_bin_9': 'CAR4_bin_9', 'seawater_seasim_SB9160_S9_bin_11': 'seawater_seasim_SB9160_S9_bin_11', 'IRC_PAM_SB0675_bin_14': 'IRC_PAM_SB0675_bin_14', 'RHO1_bin_32': 'RHO1_bin_32', 'COS4_bin_2': 'COS4_bin_2', 'RHO2_bin_38': 'RHO2_bin_38', 'COS36387_bin_2': 'COS36387_bin_2', 'COS3_bin_8': 'COS3_bin_8', 'IRC_PAM_SB0661_bin_29': 'IRC_PAM_SB0661_bin_29', 'COS3_bin_12': 'COS3_bin_12', 'RHO3_bin_41': 'RHO3_bin_41', 'GCF_900143615': 'SPOO_karimiCosta2019_GCF_900143615.1_Rhodobacteraceae_bacterium_Alg231_30_genomic', 'RHO3_bin_38': 'RHO3_bin_38', 'GCF_900149695': 'SPOO_karimiCosta2019_GCF_900149695.1_Anderseniella_sp._Alg231_50_genomic', 'IRC_PAM_SB0672_bin_11': 'IRC_PAM_SB0672_bin_11', 'STY3_bin_3': 'STY3_bin_3', 'IRC1_bin_1': 'IRC1_bin_1', 'IRC_PAM_SB0662_bin_12': 'IRC_PAM_SB0662_bin_12', 'seawater_seasim_SB9157_S6_bin_2': 'seawater_seasim_SB9157_S6_bin_2', 'RHO1_bin_40': 'RHO1_bin_40', 'IRC2_bin_8': 'IRC2_bin_8', 'seawater_22112_bin_14': 'seawater_22112_bin_14', 'seawater_42618_bin_2': 'seawater_42618_bin_2', 'COS4_bin_20': 'COS4_bin_20', 'IRC1_bin_3': 'IRC1_bin_3', 'RHO3_bin_66': 'RHO3_bin_66', 'CAR3_bin_18': 'CAR3_bin_18', 'COS1_bin_7': 'COS1_bin_7', 'GCA_001007625': 'IRCvar_burgsdorfSteindler_GCA_001007625.1_ASM100762v1_genomic', 'RHO2_bin_23': 'RHO2_bin_23', 'IRC1_bin_16': 'IRC1_bin_16', 'GCA_001543005': 'LOPHE_tianQian_GCA_001543005.1_ASM154300v1_genomic', 'CAR2_bin_6': 'CAR2_bin_6', 'IRC3_bin_25': 'IRC3_bin_25', 'IRC4_bin_6': 'IRC4_bin_6', 'IRC1_bin_10': 'IRC1_bin_10', 'COS36404_bin_19': 'COS36404_bin_19', 'RHO3_bin_12': 'RHO3_bin_12', 'CAR3_bin_3': 'CAR3_bin_3', 'IRC4_bin_37': 'IRC4_bin_37', 'IRC_PAM_SB0662_bin_49': 'IRC_PAM_SB0662_bin_49', 'COS1_bin_1': 'COS1_bin_1', 'IRC_PAM_SB0662_bin_59': 'IRC_PAM_SB0662_bin_59', 'RHO3_bin_6': 'RHO3_bin_6', 'CAR2_bin_14': 'CAR2_bin_14', 'GCA_001542995': 'LOPHE_tianQian_GCA_001542995.1_ASM154299v1_genomic', 'RHO3_bin_48': 'RHO3_bin_48', 'RHO3_bin_37': 'RHO3_bin_37', 'COS4_bin_14': 'COS4_bin_14', 'APA_bin_4': 'APA_bin_4', 'RHO2_bin_24': 'RHO2_bin_24', 'GCF_000158135': 'MYCL_zanHill_GCF_000158135.1_ASM15813v1_genomic', 'APA_bin_82': 'APA_bin_82', 'APA_bin_38': 'APA_bin_38', 'IRC_PAM_SB0675_bin_20': 'IRC_PAM_SB0675_bin_20', 'RHO3_bin_5': 'RHO3_bin_5', 'IRC_PAM_SB0661_bin_38': 'IRC_PAM_SB0661_bin_38', 'COS3_bin_5': 'COS3_bin_5', 'RHO3_bin_10': 'RHO3_bin_10', 'CAR2_bin_9': 'CAR2_bin_9', 'IRC_PAM_SB0661_bin_15': 'IRC_PAM_SB0661_bin_15', 'APA_bin_90': 'APA_bin_90', 'IRC_PAM_SB0670_bin_18': 'IRC_PAM_SB0670_bin_18', 'APA_bin_87': 'APA_bin_87', 'COS36405_bin_7': 'COS36405_bin_7', 'RHO2_bin_64': 'RHO2_bin_64', 'STY4_bin_2': 'STY4_bin_2', 'IRC2_bin_2': 'IRC2_bin_2', 'STY4_bin_9': 'STY4_bin_9', 'APA_bin_58': 'APA_bin_58', 'COS4_bin_32': 'COS4_bin_32', 'seawater_seasim_SB9156_S5_bin_8': 'seawater_seasim_SB9156_S5_bin_8', 'COS2_bin_22': 'COS2_bin_22', 'COS36405_bin_20': 'COS36405_bin_20', 'IRC_PAM_SB0662_bin_51': 'IRC_PAM_SB0662_bin_51', 'IRC_PAM_SB0665_bin_8': 'IRC_PAM_SB0665_bin_8', 'RHO3_bin_54': 'RHO3_bin_54', 'IRC_PAM_SB0666_bin_21': 'IRC_PAM_SB0666_bin_21', 'RHO2_bin_3': 'RHO2_bin_3', 'seawater_42618_bin_25': 'seawater_42618_bin_25', 'RHO2_bin_57': 'RHO2_bin_57', 'seawater_bettina_36328_bin_2': 'seawater_bettina_36328_bin_2', 'COS4_bin_11': 'COS4_bin_11', 'RHO1_bin_42': 'RHO1_bin_42', 'IRC1_bin_6': 'IRC1_bin_6', 'COS36386_bin_3': 'COS36386_bin_3', 'RHO2_bin_20': 'RHO2_bin_20', 'GCF_900149705': 'SPOO_karimiCosta2019_GCF_900149705.1_Sphingorhabdus_sp._Alg231_15_genomic', 'IRC_PAM_SB0661_bin_43': 'IRC_PAM_SB0661_bin_43', 'CAR4_bin_15': 'CAR4_bin_15', 'IRC1_bin_7': 'IRC1_bin_7', 'IRC_PAM_SB0661_bin_8': 'IRC_PAM_SB0661_bin_8', 'seawater_bettina_36328_bin_5': 'seawater_bettina_36328_bin_5', 'STY2_bin_4': 'STY2_bin_4', 'GCF_900143545': 'SPOO_karimiCosta2019_GCF_900143545.1_Loktanella_sp._Alg231_35_genomic', 'IRC_PAM_SB0664_bin_10': 'IRC_PAM_SB0664_bin_10', 'GCF_900143635': 'SPOO_karimiCosta2019_GCF_900143635.1_Rhodobacteraceae_bacterium_Alg231_04_genomic', 'COS4_bin_16': 'COS4_bin_16', 'RHO1_bin_60': 'RHO1_bin_60', 'IRC_PAM_SB0668_bin_20': 'IRC_PAM_SB0668_bin_20', 'RHO2_bin_16': 'RHO2_bin_16', 'RHO3_bin_79': 'RHO3_bin_79', 'CAR1_bin_13': 'CAR1_bin_13', 'COS1_bin_15': 'COS1_bin_15', 'IRC_PAM_SB0666_bin_1': 'IRC_PAM_SB0666_bin_1', 'CAR1_bin_2': 'CAR1_bin_2', 'IRC_PAM_SB0664_bin_3': 'IRC_PAM_SB0664_bin_3', 'COS4_bin_29': 'COS4_bin_29', 'IRC_PAM_SB0670_bin_1': 'IRC_PAM_SB0670_bin_1', 'IRC1_bin_18': 'IRC1_bin_18', 'IRC4_bin_22': 'IRC4_bin_22', 'COS36404_bin_6': 'COS36404_bin_6', 'IRC2_bin_13': 'IRC2_bin_13', 'APA_bin_18': 'APA_bin_18', 'COS36406_bin_4': 'COS36406_bin_4', 'IRC1_bin_11': 'IRC1_bin_11', 'seawater_bettina_36327_bin_15': 'seawater_bettina_36327_bin_15', 'COS1_bin_2': 'COS1_bin_2', 'seawater_seasim_SB9154_S3_bin_4': 'seawater_seasim_SB9154_S3_bin_4', 'COS36386_bin_8': 'COS36386_bin_8', 'RHO1_bin_49': 'RHO1_bin_49', 'IRC1_bin_4': 'IRC1_bin_4', 'IRC_PAM_SB0662_bin_15': 'IRC_PAM_SB0662_bin_15', 'IRC4_bin_20': 'IRC4_bin_20', 'seawater_bettina_36326_bin_1': 'seawater_bettina_36326_bin_1', 'IRC_PAM_SB0661_bin_30': 'IRC_PAM_SB0661_bin_30', 'GCF_004168585': 'OPHP_alexAntunes_GCF_004168585.1_ASM416858v1_genomic', 'RHO2_bin_11': 'RHO2_bin_11', 'IRC_PAM_SB0662_bin_37': 'IRC_PAM_SB0662_bin_37', 'RHO3_bin_2': 'RHO3_bin_2', 'COS36386_bin_18': 'COS36386_bin_18', 'IRC_PAM_SB0661_bin_24': 'IRC_PAM_SB0661_bin_24', 'seawater_bettina_36326_bin_26': 'seawater_bettina_36326_bin_26', 'IRC_PAM_SB0664_bin_15': 'IRC_PAM_SB0664_bin_15', 'COS36406_bin_19': 'COS36406_bin_19', 'COS36386_bin_10': 'COS36386_bin_10', 'IRC_PAM_SB0663_bin_5': 'IRC_PAM_SB0663_bin_5', 'IRC_PAM_SB0662_bin_21': 'IRC_PAM_SB0662_bin_21', 'COS2_bin_16': 'COS2_bin_16', 'IRC3_bin_20': 'IRC3_bin_20', 'IRC_PAM_SB0661_bin_44': 'IRC_PAM_SB0661_bin_44', 'IRC4_bin_25': 'IRC4_bin_25', 'CAR2_bin_11': 'CAR2_bin_11', 'APA_bin_72': 'APA_bin_72', 'COS4_bin_8': 'COS4_bin_8', 'seawater_seasim_SB9155_S4_bin_5': 'seawater_seasim_SB9155_S4_bin_5', 'IRC_PAM_SB0667_bin_9': 'IRC_PAM_SB0667_bin_9', 'STY2_bin_6': 'STY2_bin_6', 'STY3_bin_1': 'STY3_bin_1', 'IRC4_bin_1': 'IRC4_bin_1', 'seawater_bettina_36327_bin_4': 'seawater_bettina_36327_bin_4', 'RHO1_bin_58': 'RHO1_bin_58', 'IRC_PAM_SB0661_bin_33': 'IRC_PAM_SB0661_bin_33', 'IRC3_bin_5': 'IRC3_bin_5', 'seawater_seasim_SB9156_S5_bin_14': 'seawater_seasim_SB9156_S5_bin_14', 'IRC1_bin_22': 'IRC1_bin_22', 'COS36388_bin_4': 'COS36388_bin_4', 'RHO1_bin_9': 'RHO1_bin_9', 'RHO1_bin_66': 'RHO1_bin_66', 'APA_bin_43': 'APA_bin_43', 'RHO2_bin_28': 'RHO2_bin_28', 'seawater_seasim_SB9156_S5_bin_4': 'seawater_seasim_SB9156_S5_bin_4', 'RHO3_bin_21': 'RHO3_bin_21', 'IRC1_bin_2': 'IRC1_bin_2', 'IRC_PAM_SB0678_bin_15': 'IRC_PAM_SB0678_bin_15', 'CLI1_bin_4': 'CLI1_bin_4', 'IRC1_bin_25': 'IRC1_bin_25', 'COS36388_bin_18': 'COS36388_bin_18', 'IRC_PAM_SB0666_bin_22': 'IRC_PAM_SB0666_bin_22', 'IRC_PAM_SB0661_bin_11': 'IRC_PAM_SB0661_bin_11', 'CAR2_bin_4': 'CAR2_bin_4', 'CLI4_bin_1': 'CLI4_bin_1', 'IRC_PAM_SB0677_bin_8': 'IRC_PAM_SB0677_bin_8', 'RHO2_bin_53': 'RHO2_bin_53', 'seawater_seasim_SB9158_S7_bin_9': 'seawater_seasim_SB9158_S7_bin_9', 'COS36387_bin_18': 'COS36387_bin_18', 'seawater_42618_bin_5': 'seawater_42618_bin_5', 'COS36386_bin_29': 'COS36386_bin_29', 'RHO3_bin_51': 'RHO3_bin_51', 'RHO1_bin_72': 'RHO1_bin_72', 'COS3_bin_11': 'COS3_bin_11', 'RHO1_bin_6': 'RHO1_bin_6', 'IRC4_bin_4': 'IRC4_bin_4', 'STY2_bin_7': 'STY2_bin_7', 'RHO2_bin_35': 'RHO2_bin_35', 'RHO3_bin_20': 'RHO3_bin_20', 'APA_bin_45': 'APA_bin_45', 'IRC4_bin_35': 'IRC4_bin_35', 'COS36387_bin_9': 'COS36387_bin_9', 'IRC3_bin_22': 'IRC3_bin_22', 'RHO3_bin_26': 'RHO3_bin_26', 'CAR2_bin_26': 'CAR2_bin_26', 'CAR3_bin_13': 'CAR3_bin_13', 'IRC3_bin_7': 'IRC3_bin_7', 'RHO1_bin_30': 'RHO1_bin_30', 'RHO3_bin_14': 'RHO3_bin_14', 'COS36386_bin_6': 'COS36386_bin_6', 'RHO2_bin_43': 'RHO2_bin_43', 'IRC_PAM_SB0664_bin_5': 'IRC_PAM_SB0664_bin_5', 'RHO2_bin_15': 'RHO2_bin_15', 'RHO1_bin_2': 'RHO1_bin_2', 'IRC3_bin_10': 'IRC3_bin_10', 'RHO3_bin_18': 'RHO3_bin_18', 'APA_bin_7': 'APA_bin_7', 'COS1_bin_10': 'COS1_bin_10', 'IRC_PAM_SB0668_bin_27': 'IRC_PAM_SB0668_bin_27', 'RHO2_bin_12': 'RHO2_bin_12', 'COS36386_bin_4': 'COS36386_bin_4', 'IRC_PAM_SB0661_bin_32': 'IRC_PAM_SB0661_bin_32', 'RHO1_bin_27': 'RHO1_bin_27', 'GCA_003635315': 'MELS_podellAllen_GCA_GCA_003635315.1_ASM363531v1_genomic', 'IRC_PAM_SB0666_bin_13': 'IRC_PAM_SB0666_bin_13', 'STY2_bin_2': 'STY2_bin_2', 'RHO3_bin_17': 'RHO3_bin_17', 'seawater_seasim_SB9152_S1_bin_19': 'seawater_seasim_SB9152_S1_bin_19', 'RHO2_bin_60': 'RHO2_bin_60', 'RHO3_bin_9': 'RHO3_bin_9', 'STY4_bin_8': 'STY4_bin_8', 'seawater_42617_bin_1': 'seawater_42617_bin_1', 'COS36386_bin_35': 'COS36386_bin_35', 'COS2_bin_12': 'COS2_bin_12', 'seawater_22112_bin_12': 'seawater_22112_bin_12', 'seawater_seasim_SB9152_S1_bin_12': 'seawater_seasim_SB9152_S1_bin_12', 'CAR2_bin_8': 'CAR2_bin_8', 'CAR3_bin_5': 'CAR3_bin_5', 'COS4_bin_34': 'COS4_bin_34', 'IRC4_bin_45': 'IRC4_bin_45', 'STY4_bin_7': 'STY4_bin_7', 'IRC_PAM_SB0675_bin_12': 'IRC_PAM_SB0675_bin_12', 'COS4_bin_4': 'COS4_bin_4', 'seawater_bettina_36309_bin_3': 'seawater_bettina_36309_bin_3', 'IRC_PAM_SB0670_bin_39': 'IRC_PAM_SB0670_bin_39', 'IRC_PAM_SB0667_bin_3': 'IRC_PAM_SB0667_bin_3', 'IRC_PAM_SB0678_bin_6': 'IRC_PAM_SB0678_bin_6', 'COS36404_bin_8': 'COS36404_bin_8', 'seawater_22112_bin_1': 'seawater_22112_bin_1', 'CAR4_bin_14': 'CAR4_bin_14', 'COS36388_bin_5': 'COS36388_bin_5', 'seawater_22112_bin_17': 'seawater_22112_bin_17', 'COS36388_bin_17': 'COS36388_bin_17', 'COS36386_bin_9': 'COS36386_bin_9', 'GCA_001007635': 'APA_burgsdorfSteindler_GCA_001007635.1_ASM100763v1_genomic', 'APA_bin_39': 'APA_bin_39', 'COS36405_bin_11': 'COS36405_bin_11', 'RHO2_bin_50': 'RHO2_bin_50', 'RHO2_bin_26': 'RHO2_bin_26', 'IRC3_bin_35': 'IRC3_bin_35', 'RHO1_bin_7': 'RHO1_bin_7', 'IRC3_bin_14': 'IRC3_bin_14', 'RHO1_bin_21': 'RHO1_bin_21', 'IRC_PAM_SB0675_bin_18': 'IRC_PAM_SB0675_bin_18', 'APA_bin_15': 'APA_bin_15', 'APA_bin_86': 'APA_bin_86', 'seawater_bettina_36326_bin_7': 'seawater_bettina_36326_bin_7', 'RHO1_bin_50': 'RHO1_bin_50', 'RHO3_bin_15': 'RHO3_bin_15', 'COS36406_bin_6': 'COS36406_bin_6', 'IRC_PAM_SB0661_bin_14': 'IRC_PAM_SB0661_bin_14', 'COS4_bin_51': 'COS4_bin_51', 'IRC_PAM_SB0662_bin_53': 'IRC_PAM_SB0662_bin_53', 'COS3_bin_10': 'COS3_bin_10', 'RHO1_bin_70': 'RHO1_bin_70', 'IRC_PAM_SB0677_bin_14': 'IRC_PAM_SB0677_bin_14', 'COS4_bin_13': 'COS4_bin_13', 'APA_bin_96': 'APA_bin_96', 'COS36404_bin_10': 'COS36404_bin_10', 'STY4_bin_4': 'STY4_bin_4', 'COS2_bin_2': 'COS2_bin_2', 'RHO3_bin_52': 'RHO3_bin_52', 'COS4_bin_27': 'COS4_bin_27', 'APA_bin_41': 'APA_bin_41', 'IRC_PAM_SB0665_bin_5': 'IRC_PAM_SB0665_bin_5', 'Aquimarina_spAU119': 'TED_estevesThomas2016_Aquimarina_spAU119_2606217184', 'COS4_bin_17': 'COS4_bin_17', 'COS36388_bin_9': 'COS36388_bin_9', 'IRC_PAM_SB0661_bin_21': 'IRC_PAM_SB0661_bin_21', 'IRC_PAM_SB0662_bin_8': 'IRC_PAM_SB0662_bin_8', 'IRC_PAM_SB0664_bin_14': 'IRC_PAM_SB0664_bin_14', 'COS36386_bin_20': 'COS36386_bin_20', 'APA_bin_29': 'APA_bin_29', 'IRC4_bin_12': 'IRC4_bin_12', 'COS1_bin_13': 'COS1_bin_13', 'IRC_PAM_SB0675_bin_29': 'IRC_PAM_SB0675_bin_29', 'COS36387_bin_16': 'COS36387_bin_16', 'IRC_PAM_SB0661_bin_26': 'IRC_PAM_SB0661_bin_26', 'COS4_bin_55': 'COS4_bin_55', 'IRC_PAM_SB0668_bin_12': 'IRC_PAM_SB0668_bin_12', 'APA_bin_98': 'APA_bin_98', 'IRC_PAM_SB0665_bin_12': 'IRC_PAM_SB0665_bin_12', 'IRC3_bin_11': 'IRC3_bin_11', 'APA_bin_55': 'APA_bin_55', 'seawater_seasim_SB9152_S1_bin_4': 'seawater_seasim_SB9152_S1_bin_4', 'COS36386_bin_12': 'COS36386_bin_12', 'seawater_bettina_36328_bin_30': 'seawater_bettina_36328_bin_30', 'COS36388_bin_8': 'COS36388_bin_8', 'COS2_bin_5': 'COS2_bin_5', 'GCF_900143555': 'SPOO_karimiCosta2019_GCF_900143555.1_Labrenzia_sp._Alg231_36_genomic', 'RHO1_bin_81': 'RHO1_bin_81', 'CLI4_bin_2': 'CLI4_bin_2', 'APA_bin_63': 'APA_bin_63', 'SPOO_karimiCosta_FZLS01': 'SPOO_karimiCosta_FZLS01', 'APA_bin_69': 'APA_bin_69', 'IRC4_bin_21': 'IRC4_bin_21', 'IRC_PAM_SB0666_bin_6': 'IRC_PAM_SB0666_bin_6', 'RHO2_bin_52': 'RHO2_bin_52', 'COS3_bin_3': 'COS3_bin_3', 'RHO1_bin_33': 'RHO1_bin_33', 'RHO2_bin_10': 'RHO2_bin_10', 'COS2_bin_23': 'COS2_bin_23', 'COS36387_bin_22': 'COS36387_bin_22', 'PER4_bin_1': 'PER4_bin_1', 'APA_bin_48': 'APA_bin_48', 'RHO3_bin_27': 'RHO3_bin_27', 'seawater_seasim_SB9153_S2_bin_10': 'seawater_seasim_SB9153_S2_bin_10', 'IRC_PAM_SB0661_bin_19': 'IRC_PAM_SB0661_bin_19', 'RHO1_bin_34': 'RHO1_bin_34', 'IRC3_bin_6': 'IRC3_bin_6', 'COS36386_bin_15': 'COS36386_bin_15', 'CAR3_bin_4': 'CAR3_bin_4', 'APA_bin_32': 'APA_bin_32', 'CAR1_bin_6': 'CAR1_bin_6', 'RHO1_bin_15': 'RHO1_bin_15', 'RHO3_bin_39': 'RHO3_bin_39', 'IRC1_bin_30': 'IRC1_bin_30', 'RHO1_bin_11': 'RHO1_bin_11', 'GCA_002007405': 'SUB_tianQian_GCA_002007405.1_ASM200740v1_genomic', 'IRC_PAM_SB0664_bin_24': 'IRC_PAM_SB0664_bin_24', 'IRC_PAM_SB0662_bin_36': 'IRC_PAM_SB0662_bin_36', 'IRC_PAM_SB0661_bin_31': 'IRC_PAM_SB0661_bin_31', 'IRC3_bin_32': 'IRC3_bin_32', 'COS36406_bin_13': 'COS36406_bin_13', 'IRC2_bin_4': 'IRC2_bin_4', 'GCF_002573675': 'TED_braunBugni_GCF_002573675.1_ASM257367v1_genomic', 'IRC3_bin_4': 'IRC3_bin_4', 'COS36386_bin_11': 'COS36386_bin_11', 'COS2_bin_18': 'COS2_bin_18', 'COS36405_bin_5': 'COS36405_bin_5', 'seawater_bettina_36326_bin_6': 'seawater_bettina_36326_bin_6', 'IRC3_bin_9': 'IRC3_bin_9', 'APA_bin_40': 'APA_bin_40', 'seawater_seasim_SB9157_S6_bin_13': 'seawater_seasim_SB9157_S6_bin_13', 'CAR3_bin_2': 'CAR3_bin_2', 'seawater_42616_bin_2': 'seawater_42616_bin_2', 'RHO1_bin_39': 'RHO1_bin_39', 'RHO1_bin_64': 'RHO1_bin_64', 'IRC1_bin_37': 'IRC1_bin_37', 'IRC_PAM_SB0662_bin_28': 'IRC_PAM_SB0662_bin_28', 'COS4_bin_22': 'COS4_bin_22', 'COS36405_bin_14': 'COS36405_bin_14', 'COS4_bin_47': 'COS4_bin_47', 'COS3_bin_4': 'COS3_bin_4', 'RHO1_bin_28': 'RHO1_bin_28', 'IRC_PAM_SB0668_bin_11': 'IRC_PAM_SB0668_bin_11', 'STY1_bin_8': 'STY1_bin_8', 'RHO1_bin_22': 'RHO1_bin_22', 'COS4_bin_28': 'COS4_bin_28', 'APA_bin_12': 'APA_bin_12', 'seawater_42615_bin_2': 'seawater_42615_bin_2', 'IRC_PAM_SB0665_bin_19': 'IRC_PAM_SB0665_bin_19', 'COS36405_bin_2': 'COS36405_bin_2', 'COS36405_bin_10': 'COS36405_bin_10', 'COS36386_bin_28': 'COS36386_bin_28', 'RHO3_bin_70': 'RHO3_bin_70', 'seawater_bettina_36309_bin_6': 'seawater_bettina_36309_bin_6', 'IRC_PAM_SB0667_bin_2': 'IRC_PAM_SB0667_bin_2', 'APA_bin_34': 'APA_bin_34', 'IRC_PAM_SB0665_bin_20': 'IRC_PAM_SB0665_bin_20', 'IRC1_bin_23': 'IRC1_bin_23', 'COS3_bin_1': 'COS3_bin_1', 'RHO2_bin_40': 'RHO2_bin_40', 'COS4_bin_10': 'COS4_bin_10', 'COS36387_bin_10': 'COS36387_bin_10', 'GCA_000583135': 'HALC_tianQian2014_GCA_000583135.1_Thioalkalivibrio_spongium_HK1_genomic', 'RHO3_bin_50': 'RHO3_bin_50', 'IRC_PAM_SB0666_bin_15': 'IRC_PAM_SB0666_bin_15', 'COS36387_bin_7': 'COS36387_bin_7', 'GCF_900141785': 'HALO_yoonOh_GCF_900141785.1_IMGID2622736502_genomic', 'COS4_bin_26': 'COS4_bin_26', 'RHO2_bin_41': 'RHO2_bin_41', 'APA_bin_71': 'APA_bin_71', 'COS1_bin_18': 'COS1_bin_18', 'COS36406_bin_18': 'COS36406_bin_18', 'IRC_PAM_SB0662_bin_19': 'IRC_PAM_SB0662_bin_19', 'APA_bin_53': 'APA_bin_53', 'IRC_PAM_SB0666_bin_9': 'IRC_PAM_SB0666_bin_9', 'RHO2_bin_8': 'RHO2_bin_8', 'RHO2_bin_31': 'RHO2_bin_31', 'STY1_bin_1': 'STY1_bin_1', 'APA_bin_56': 'APA_bin_56', 'APA_bin_89': 'APA_bin_89', 'RHO2_bin_62': 'RHO2_bin_62', 'IRC1_bin_38': 'IRC1_bin_38', 'COS2_bin_8': 'COS2_bin_8', 'GCA_000522425': 'THES_wilsonPiel_GCA_000522425.1_v3_genomic', 'seawater_bettina_36328_bin_7': 'seawater_bettina_36328_bin_7', 'IRC4_bin_40': 'IRC4_bin_40', 'Pseudovibrio_spAU243': 'CYMC_estevesThomas2016_Pseudovibrio_spAU243_2606217185', 'IRC_PAM_SB0668_bin_14': 'IRC_PAM_SB0668_bin_14', 'COS4_bin_25': 'COS4_bin_25', 'RHO1_bin_8': 'RHO1_bin_8', 'GCA_001543015': 'LOPHE_tianQian_GCA_001543015.1_ASM154301v1_genomic', 'GCA_001007665': 'THES_burgsdorfSteindler_GCA_001007665.1_ASM100766v1_genomic', 'IRC_PAM_SB0664_bin_12': 'IRC_PAM_SB0664_bin_12', 'COS1_bin_3': 'COS1_bin_3', 'IRC4_bin_15': 'IRC4_bin_15', 'CAR2_bin_1': 'CAR2_bin_1', 'IRC3_bin_15': 'IRC3_bin_15', 'CAR1_bin_18': 'CAR1_bin_18', 'RHO1_bin_12': 'RHO1_bin_12', 'IRC_PAM_SB0665_bin_11': 'IRC_PAM_SB0665_bin_11', 'RHO2_bin_22': 'RHO2_bin_22', 'RHO1_bin_20': 'RHO1_bin_20', 'STY4_bin_3': 'STY4_bin_3', 'IRC4_bin_9': 'IRC4_bin_9', 'STY1_bin_3': 'STY1_bin_3', 'COS1_bin_5': 'COS1_bin_5', 'seawater_seasim_SB9156_S5_bin_20': 'seawater_seasim_SB9156_S5_bin_20', 'RHO2_bin_25': 'RHO2_bin_25', 'IRC_PAM_SB0667_bin_14': 'IRC_PAM_SB0667_bin_14', 'RHO2_bin_13': 'RHO2_bin_13', 'IRC_PAM_SB0662_bin_23': 'IRC_PAM_SB0662_bin_23', 'seawater_seasim_SB9157_S6_bin_7': 'seawater_seasim_SB9157_S6_bin_7', 'RHO3_bin_8': 'RHO3_bin_8', 'seawater_22112_bin_8': 'seawater_22112_bin_8', 'IRC_PAM_SB0664_bin_33': 'IRC_PAM_SB0664_bin_33', 'COS36386_bin_37': 'COS36386_bin_37', 'RHO2_bin_5': 'RHO2_bin_5', 'RHO2_bin_19': 'RHO2_bin_19', 'IRC_PAM_SB0662_bin_24': 'IRC_PAM_SB0662_bin_24', 'GCF_000743705': 'CRAC_dobervaLami_GCF_000743705.1_ASM74370v1_genomic', 'seawater_42618_bin_9': 'seawater_42618_bin_9', 'IRC4_bin_49': 'IRC4_bin_49', 'IRC_PAM_SB0668_bin_21': 'IRC_PAM_SB0668_bin_21', 'APA_bin_28': 'APA_bin_28', 'COS36406_bin_2': 'COS36406_bin_2', 'APA_bin_61': 'APA_bin_61', 'IRC_PAM_SB0678_bin_2': 'IRC_PAM_SB0678_bin_2', 'COS3_bin_17': 'COS3_bin_17', 'IRC_PAM_SB0664_bin_11': 'IRC_PAM_SB0664_bin_11', 'seawater_seasim_SB9152_S1_bin_2': 'seawater_seasim_SB9152_S1_bin_2', 'seawater_22112_bin_9': 'seawater_22112_bin_9', 'RHO3_bin_33': 'RHO3_bin_33', 'IRC4_bin_39': 'IRC4_bin_39', 'SPOO_karimiCosta_FZLR01': 'SPOO_karimiCosta_FZLR01', 'RHO1_bin_82': 'RHO1_bin_82', 'CAR4_bin_13': 'CAR4_bin_13', 'IRC3_bin_17': 'IRC3_bin_17', 'CAR2_bin_22': 'CAR2_bin_22', 'SPOO_karimiCosta_FZLQ01': 'SPOO_karimiCosta_FZLQ01', 'IRC_PAM_SB0675_bin_22': 'IRC_PAM_SB0675_bin_22', 'IRC_PAM_SB0664_bin_6': 'IRC_PAM_SB0664_bin_6', 'APA_bin_1': 'APA_bin_1', 'RHO1_bin_51': 'RHO1_bin_51', 'COS36386_bin_31': 'COS36386_bin_31', 'IRC2_bin_12': 'IRC2_bin_12', 'APA_bin_74': 'APA_bin_74', 'IRC_PAM_SB0661_bin_34': 'IRC_PAM_SB0661_bin_34', 'APA_bin_8': 'APA_bin_8', 'COS36406_bin_1': 'COS36406_bin_1', 'RHO3_bin_4': 'RHO3_bin_4', 'CAR4_bin_4': 'CAR4_bin_4', 'RHO2_bin_27': 'RHO2_bin_27', 'COS36406_bin_9': 'COS36406_bin_9', 'COS36388_bin_1': 'COS36388_bin_1', 'IRC2_bin_1': 'IRC2_bin_1', 'COS36388_bin_16': 'COS36388_bin_16', 'APA_bin_23': 'APA_bin_23', 'APA_bin_62': 'APA_bin_62', 'CAR1_bin_16': 'CAR1_bin_16', 'IRC2_bin_7': 'IRC2_bin_7', 'CAR1_bin_4': 'CAR1_bin_4', 'IRC1_bin_19': 'IRC1_bin_19', 'CAR3_bin_9': 'CAR3_bin_9', 'COS4_bin_49': 'COS4_bin_49', 'IRC3_bin_37': 'IRC3_bin_37', 'CAR3_bin_15': 'CAR3_bin_15', 'RHO1_bin_55': 'RHO1_bin_55', 'RHO1_bin_59': 'RHO1_bin_59', 'IRC_PAM_SB0661_bin_1': 'IRC_PAM_SB0661_bin_1', 'CAR3_bin_10': 'CAR3_bin_10', 'COS4_bin_12': 'COS4_bin_12', 'seawater_seasim_SB9152_S1_bin_18': 'seawater_seasim_SB9152_S1_bin_18', 'seawater_bettina_36327_bin_6': 'seawater_bettina_36327_bin_6', 'IRC3_bin_8': 'IRC3_bin_8', 'IRC4_bin_17': 'IRC4_bin_17', 'CAR1_bin_5': 'CAR1_bin_5', 'RHO3_bin_22': 'RHO3_bin_22', 'IRC_PAM_SB0675_bin_3': 'IRC_PAM_SB0675_bin_3', 'seawater_seasim_SB9154_S3_bin_1': 'seawater_seasim_SB9154_S3_bin_1', 'COS4_bin_1': 'COS4_bin_1', 'GCA_000200715': 'AXIM_hallam_GCA_000200715.1_genomic', 'IRC_PAM_SB0661_bin_40': 'IRC_PAM_SB0661_bin_40', 'RHO1_bin_52': 'RHO1_bin_52', 'IRC4_bin_30': 'IRC4_bin_30', 'RHO1_bin_17': 'RHO1_bin_17', 'COS3_bin_16': 'COS3_bin_16', 'GCF_900079515': 'SPOO_alexAntunes_GCF_900079515.1_Shewanella_sp.Alg231_23_genomic', 'GCF_000156235': 'MYCL_bondarevVogt_GCF_000156235.1_ASM15623v1_genomic', 'seawater_42615_bin_16': 'seawater_42615_bin_16', 'RHO3_bin_19': 'RHO3_bin_19', 'CLI1_bin_2': 'CLI1_bin_2', 'IRC1_bin_31': 'IRC1_bin_31', 'IRC_PAM_SB0665_bin_17': 'IRC_PAM_SB0665_bin_17', 'seawater_bettina_36327_bin_3': 'seawater_bettina_36327_bin_3', 'COS36387_bin_14': 'COS36387_bin_14', 'COS4_bin_9': 'COS4_bin_9', 'seawater_bettina_36308_bin_18': 'seawater_bettina_36308_bin_18', 'COS1_bin_16': 'COS1_bin_16', 'RHO2_bin_17': 'RHO2_bin_17', 'COS36404_bin_5': 'COS36404_bin_5', 'STY1_bin_7': 'STY1_bin_7', 'IRC_PAM_SB0662_bin_30': 'IRC_PAM_SB0662_bin_30', 'RHO2_bin_2': 'RHO2_bin_2', 'IRC_PAM_SB0662_bin_1': 'IRC_PAM_SB0662_bin_1', 'RHO3_bin_29': 'RHO3_bin_29', 'COS36388_bin_3': 'COS36388_bin_3', 'seawater_seasim_SB9154_S3_bin_3': 'seawater_seasim_SB9154_S3_bin_3', 'IRC_PAM_SB0664_bin_7': 'IRC_PAM_SB0664_bin_7', 'COS36387_bin_15': 'COS36387_bin_15', 'IRC4_bin_11': 'IRC4_bin_11', 'IRC4_bin_42': 'IRC4_bin_42', 'IRC4_bin_13': 'IRC4_bin_13', 'RHO1_bin_43': 'RHO1_bin_43', 'CAR2_bin_19': 'CAR2_bin_19', 'IRC1_bin_9': 'IRC1_bin_9', 'IRC3_bin_3': 'IRC3_bin_3', 'IRC_PAM_SB0675_bin_23': 'IRC_PAM_SB0675_bin_23', 'seawater_seasim_SB9156_S5_bin_3': 'seawater_seasim_SB9156_S5_bin_3', 'RHO3_bin_57': 'RHO3_bin_57', 'IRC_PAM_SB0662_bin_34': 'IRC_PAM_SB0662_bin_34', 'CAR3_bin_17': 'CAR3_bin_17', 'IRC_PAM_SB0672_bin_21': 'IRC_PAM_SB0672_bin_21', 'RHO2_bin_39': 'RHO2_bin_39', 'COS4_bin_18': 'COS4_bin_18', 'IRC_PAM_SB0668_bin_13': 'IRC_PAM_SB0668_bin_13', 'seawater_22112_bin_4': 'seawater_22112_bin_4', 'IRC_PAM_SB0665_bin_16': 'IRC_PAM_SB0665_bin_16', 'COS3_bin_15': 'COS3_bin_15', 'RHO1_bin_69': 'RHO1_bin_69', 'RHO1_bin_26': 'RHO1_bin_26', 'IRC_PAM_SB0661_bin_39': 'IRC_PAM_SB0661_bin_39', 'COS36386_bin_5': 'COS36386_bin_5', 'seawater_seasim_SB9153_S2_bin_13': 'seawater_seasim_SB9153_S2_bin_13', 'GCF_000264395': 'DYSA_liuLi2012_GCF_000264395.1_C89_version_1_genomic', 'RHO2_bin_56': 'RHO2_bin_56', 'APA_bin_6': 'APA_bin_6', 'IRC_PAM_SB0661_bin_6': 'IRC_PAM_SB0661_bin_6', 'seawater_bettina_36327_bin_7': 'seawater_bettina_36327_bin_7', 'CHO1_bin_4': 'CHO1_bin_4', 'APA_bin_10': 'APA_bin_10', 'RHO1_bin_10': 'RHO1_bin_10', 'IRC_PAM_SB0668_bin_7': 'IRC_PAM_SB0668_bin_7', 'COS36387_bin_11': 'COS36387_bin_11', 'IRC_PAM_SB0670_bin_19': 'IRC_PAM_SB0670_bin_19', 'RHO1_bin_29': 'RHO1_bin_29', 'COS36405_bin_1': 'COS36405_bin_1', 'COS1_bin_21': 'COS1_bin_21', 'IRC_PAM_SB0661_bin_55': 'IRC_PAM_SB0661_bin_55', 'COS4_bin_36': 'COS4_bin_36', 'STY2_bin_1': 'STY2_bin_1', 'IRC_PAM_SB0662_bin_39': 'IRC_PAM_SB0662_bin_39', 'RHO2_bin_55': 'RHO2_bin_55', 'APA_bin_97': 'APA_bin_97', 'COS4_bin_46': 'COS4_bin_46', 'COS4_bin_15': 'COS4_bin_15', 'IRC_PAM_SB0664_bin_2': 'IRC_PAM_SB0664_bin_2', 'IRC3_bin_2': 'IRC3_bin_2', 'seawater_seasim_SB9155_S4_bin_4': 'seawater_seasim_SB9155_S4_bin_4', 'seawater_42617_bin_6': 'seawater_42617_bin_6', 'COS36386_bin_14': 'COS36386_bin_14', 'IRC_PAM_SB0675_bin_16': 'IRC_PAM_SB0675_bin_16', 'IRC_PAM_SB0667_bin_13': 'IRC_PAM_SB0667_bin_13', 'RHO2_bin_6': 'RHO2_bin_6', 'COS36406_bin_7': 'COS36406_bin_7', 'IRC1_bin_26': 'IRC1_bin_26', 'RHO3_bin_1': 'RHO3_bin_1', 'IRC_PAM_SB0662_bin_10': 'IRC_PAM_SB0662_bin_10', 'RHO3_bin_24': 'RHO3_bin_24', 'IRC4_bin_16': 'IRC4_bin_16', 'APA_bin_22': 'APA_bin_22', 'CAR1_bin_7': 'CAR1_bin_7', 'RHO3_bin_80': 'RHO3_bin_80', 'seawater_42618_bin_6': 'seawater_42618_bin_6', 'COS36387_bin_3': 'COS36387_bin_3', 'CAR1_bin_15': 'CAR1_bin_15', 'RHO3_bin_25': 'RHO3_bin_25', 'CAR1_bin_14': 'CAR1_bin_14', 'STY1_bin_2': 'STY1_bin_2', 'APA_bin_3': 'APA_bin_3', 'RHO1_bin_24': 'RHO1_bin_24', 'IRC1_bin_20': 'IRC1_bin_20', 'IRC_PAM_SB0668_bin_5': 'IRC_PAM_SB0668_bin_5', 'seawater_22112_bin_2': 'seawater_22112_bin_2', 'IRC3_bin_16': 'IRC3_bin_16', 'RHO3_bin_23': 'RHO3_bin_23', 'CAR3_bin_14': 'CAR3_bin_14', 'CAR4_bin_16': 'CAR4_bin_16', 'CAR4_bin_1': 'CAR4_bin_1', 'GCF_001431305': 'POLP_alexAntunes_GCF_001431305.1_ASM143130v1_genomic', 'COS36405_bin_8': 'COS36405_bin_8', 'COS4_bin_23': 'COS4_bin_23', 'GCF_900143565': 'SPOO_karimiCosta2019_GCF_900143565.1_Pseudovibrio_sp._Alg231_02_genomic', 'APA_bin_19': 'APA_bin_19', 'RHO3_bin_49': 'RHO3_bin_49', 'CAR3_bin_11': 'CAR3_bin_11', 'COS1_bin_9': 'COS1_bin_9', 'RHO1_bin_68': 'RHO1_bin_68', 'IRC3_bin_21': 'IRC3_bin_21', 'IRC_PAM_SB0666_bin_11': 'IRC_PAM_SB0666_bin_11', 'RHO3_bin_74': 'RHO3_bin_74', 'RHO1_bin_31': 'RHO1_bin_31', 'COS36405_bin_12': 'COS36405_bin_12', 'GCA_003635195': 'PSE_podellAllen_GCA_003635195.1_ASM363519v1_genomic', 'RHO3_bin_43': 'RHO3_bin_43', 'seawater_seasim_SB9153_S2_bin_4': 'seawater_seasim_SB9153_S2_bin_4', 'IRC_PAM_SB0664_bin_28': 'IRC_PAM_SB0664_bin_28', 'RHO3_bin_73': 'RHO3_bin_73', 'IRC_PAM_SB0661_bin_27': 'IRC_PAM_SB0661_bin_27', 'COS36404_bin_3': 'COS36404_bin_3'}

raw2new_name_dict = {'CAR4_bin_6': 'CAR4_bin_6', 'COS2_bin_4': 'COS2_bin_4', 'COS4_bin_21': 'COS4_bin_21', 'COS2_bin_10': 'COS2_bin_10', 'IRC4_bin_41': 'IRC4_bin_41', 'IRC_PAM_SB0675_bin_5': 'IRC_PAM_SB0675_bin_5', 'RHO2_bin_49': 'RHO2_bin_49', 'IRC_PAM_SB0665_bin_25': 'IRC_PAM_SB0665_bin_25', 'COS2_bin_7': 'COS2_bin_7', 'IRC_PAM_SB0667_bin_1': 'IRC_PAM_SB0667_bin_1', 'RHO3_bin_84': 'RHO3_bin_84', 'IRC3_bin_26': 'IRC3_bin_26', 'IRC3_bin_28': 'IRC3_bin_28', 'RHO1_bin_56': 'RHO1_bin_56', 'IRC_PAM_SB0668_bin_19': 'IRC_PAM_SB0668_bin_19', 'IRC_PAM_SB0666_bin_34': 'IRC_PAM_SB0666_bin_34', 'seawater_bettina_36328_bin_3': 'seawater_bettina_36328_bin_3', 'COS36405_bin_3': 'COS36405_bin_3', 'RHO1_bin_37': 'RHO1_bin_37', 'PSE_podellAllen_GCA_003635205.1_ASM363520v1_genomic': 'GCA_003635205', 'IRC_PAM_SB0661_bin_2': 'IRC_PAM_SB0661_bin_2', 'RHO1_bin_65': 'RHO1_bin_65', 'IRC_PAM_SB0665_bin_4': 'IRC_PAM_SB0665_bin_4', 'IRC3_bin_18': 'IRC3_bin_18', 'RHO1_bin_53': 'RHO1_bin_53', 'COS3_bin_9': 'COS3_bin_9', 'RHO2_bin_42': 'RHO2_bin_42', 'CAR1_bin_10': 'CAR1_bin_10', 'IRC_PAM_SB0661_bin_17': 'IRC_PAM_SB0661_bin_17', 'IRC4_bin_3': 'IRC4_bin_3', 'COS36388_bin_13': 'COS36388_bin_13', 'APA_bin_59': 'APA_bin_59', 'APA_bin_42': 'APA_bin_42', 'IRC_PAM_SB0665_bin_27': 'IRC_PAM_SB0665_bin_27', 'CAR2_bin_5': 'CAR2_bin_5', 'seawater_bettina_36310_bin_3': 'seawater_bettina_36310_bin_3', 'OPHP_alexAntunes_GCF_004168585.1_ASM416858v1_genomic': 'GCF_004168585', 'IRC4_bin_29': 'IRC4_bin_29', 'IRC_PAM_SB0661_bin_5': 'IRC_PAM_SB0661_bin_5', 'COS36387_bin_19': 'COS36387_bin_19', 'IRC_PAM_SB0661_bin_22': 'IRC_PAM_SB0661_bin_22', 'SPOO_alexAntunes_GCF_900079515.1_Shewanella_sp.Alg231_23_genomic': 'GCF_900079515', 'seawater_22112_bin_25': 'seawater_22112_bin_25', 'RHO3_bin_32': 'RHO3_bin_32', 'IRC_PAM_SB0665_bin_9': 'IRC_PAM_SB0665_bin_9', 'IRC1_bin_14': 'IRC1_bin_14', 'CAR2_bin_13': 'CAR2_bin_13', 'COS36388_bin_11': 'COS36388_bin_11', 'IRC_PAM_SB0661_bin_16': 'IRC_PAM_SB0661_bin_16', 'APA_bin_13': 'APA_bin_13', 'IRC_PAM_SB0677_bin_19': 'IRC_PAM_SB0677_bin_19', 'COS36386_bin_13': 'COS36386_bin_13', 'RHO3_bin_28': 'RHO3_bin_28', 'APA_bin_102': 'APA_bin_102', 'CAR1_bin_9': 'CAR1_bin_9', 'APA_bin_91': 'APA_bin_91', 'COS4_bin_42': 'COS4_bin_42', 'COS1_bin_11': 'COS1_bin_11', 'APA_bin_52': 'APA_bin_52', 'COS36386_bin_2': 'COS36386_bin_2', 'RHO2_bin_15': 'RHO2_bin_15', 'IRC_PAM_SB0664_bin_16': 'IRC_PAM_SB0664_bin_16', 'RHO3_bin_59': 'RHO3_bin_59', 'RHO1_bin_25': 'RHO1_bin_25', 'CLI1_bin_3': 'CLI1_bin_3', 'IRC1_bin_27': 'IRC1_bin_27', 'STY1_bin_6': 'STY1_bin_6', 'IRC_PAM_SB0675_bin_4': 'IRC_PAM_SB0675_bin_4', 'COS4_bin_7': 'COS4_bin_7', 'APA_bin_93': 'APA_bin_93', 'RHO1_bin_13': 'RHO1_bin_13', 'IRC_PAM_SB0677_bin_15': 'IRC_PAM_SB0677_bin_15', 'STY3_bin_6': 'STY3_bin_6', 'APA_bin_5': 'APA_bin_5', 'IRC_PAM_SB0673_bin_10': 'IRC_PAM_SB0673_bin_10', 'seawater_bettina_36309_bin_5': 'seawater_bettina_36309_bin_5', 'IRC_PAM_SB0678_bin_5': 'IRC_PAM_SB0678_bin_5', 'COS36404_bin_12': 'COS36404_bin_12', 'IRC_PAM_SB0677_bin_1': 'IRC_PAM_SB0677_bin_1', 'COS36387_bin_20': 'COS36387_bin_20', 'CAR2_bin_10': 'CAR2_bin_10', 'IRC_PAM_SB0662_bin_27': 'IRC_PAM_SB0662_bin_27', 'IRC1_bin_21': 'IRC1_bin_21', 'IRC1_bin_34': 'IRC1_bin_34', 'IRC_PAM_SB0678_bin_7': 'IRC_PAM_SB0678_bin_7', 'STY1_bin_5': 'STY1_bin_5', 'IRC_PAM_SB0675_bin_1': 'IRC_PAM_SB0675_bin_1', 'RHO3_bin_71': 'RHO3_bin_71', 'IRC4_bin_7': 'IRC4_bin_7', 'IRC_PAM_SB0677_bin_16': 'IRC_PAM_SB0677_bin_16', 'IRC2_bin_10': 'IRC2_bin_10', 'seawater_22112_bin_29': 'seawater_22112_bin_29', 'COS36386_bin_32': 'COS36386_bin_32', 'seawater_seasim_SB9160_S9_bin_9': 'seawater_seasim_SB9160_S9_bin_9', 'seawater_bettina_36328_bin_8': 'seawater_bettina_36328_bin_8', 'seawater_seasim_SB9156_S5_bin_6': 'seawater_seasim_SB9156_S5_bin_6', 'IRC_PAM_SB0664_bin_4': 'IRC_PAM_SB0664_bin_4', 'RHO1_bin_23': 'RHO1_bin_23', 'COS36388_bin_15': 'COS36388_bin_15', 'COS2_bin_3': 'COS2_bin_3', 'CHO1_bin_2': 'CHO1_bin_2', 'APA_bin_80': 'APA_bin_80', 'COS2_bin_6': 'COS2_bin_6', 'CLI1_bin_1': 'CLI1_bin_1', 'seawater_42618_bin_29': 'seawater_42618_bin_29', 'COS3_bin_6': 'COS3_bin_6', 'seawater_seasim_SB9152_S1_bin_8': 'seawater_seasim_SB9152_S1_bin_8', 'seawater_bettina_36308_bin_5': 'seawater_bettina_36308_bin_5', 'IRC_PAM_SB0677_bin_11': 'IRC_PAM_SB0677_bin_11', 'RHO1_bin_18': 'RHO1_bin_18', 'RHO3_bin_40': 'RHO3_bin_40', 'IRC_PAM_SB0662_bin_11': 'IRC_PAM_SB0662_bin_11', 'IRC2_bin_3': 'IRC2_bin_3', 'IRC3_bin_13': 'IRC3_bin_13', 'RHO3_bin_36': 'RHO3_bin_36', 'DYSA_liuLi2012_GCF_000264395.1_C89_version_1_genomic': 'GCF_000264395', 'IRC2_bin_11': 'IRC2_bin_11', 'seawater_22112_bin_6': 'seawater_22112_bin_6', 'RHO2_bin_67': 'RHO2_bin_67', 'COS1_bin_8': 'COS1_bin_8', 'CAR1_bin_1': 'CAR1_bin_1', 'IRC_PAM_SB0662_bin_7': 'IRC_PAM_SB0662_bin_7', 'IRC_PAM_SB0668_bin_1': 'IRC_PAM_SB0668_bin_1', 'IRC_PAM_SB0677_bin_7': 'IRC_PAM_SB0677_bin_7', 'seawater_22112_bin_7': 'seawater_22112_bin_7', 'COS1_bin_4': 'COS1_bin_4', 'COS3_bin_8': 'COS3_bin_8', 'seawater_42617_bin_7': 'seawater_42617_bin_7', 'seawater_42618_bin_3': 'seawater_42618_bin_3', 'IRC2_bin_5': 'IRC2_bin_5', 'RHO2_bin_36': 'RHO2_bin_36', 'COS36386_bin_1': 'COS36386_bin_1', 'APA_bin_27': 'APA_bin_27', 'CAR2_bin_16': 'CAR2_bin_16', 'COS2_bin_19': 'COS2_bin_19', 'seawater_22112_bin_16': 'seawater_22112_bin_16', 'RHO1_bin_3': 'RHO1_bin_3', 'RHO3_bin_65': 'RHO3_bin_65', 'RHO3_bin_67': 'RHO3_bin_67', 'IRC4_bin_14': 'IRC4_bin_14', 'CLI3_bin_1': 'CLI3_bin_1', 'RHO3_bin_68': 'RHO3_bin_68', 'CAR2_bin_3': 'CAR2_bin_3', 'COS4_bin_6': 'COS4_bin_6', 'IRC1_bin_13': 'IRC1_bin_13', 'CAR2_bin_18': 'CAR2_bin_18', 'RHO2_bin_13': 'RHO2_bin_13', 'LOPHE_tianQian_GCA_001543015.1_ASM154301v1_genomic': 'GCA_001543015', 'COS36388_bin_6': 'COS36388_bin_6', 'seawater_bettina_36308_bin_3': 'seawater_bettina_36308_bin_3', 'CAR2_bin_15': 'CAR2_bin_15', 'COS3_bin_13': 'COS3_bin_13', 'CLI2_bin_1': 'CLI2_bin_1', 'CAR4_bin_5': 'CAR4_bin_5', 'seawater_22112_bin_15': 'seawater_22112_bin_15', 'IRC4_bin_10': 'IRC4_bin_10', 'RHO3_bin_53': 'RHO3_bin_53', 'RHO1_bin_85': 'RHO1_bin_85', 'seawater_seasim_SB9152_S1_bin_3': 'seawater_seasim_SB9152_S1_bin_3', 'IRC_PAM_SB0665_bin_24': 'IRC_PAM_SB0665_bin_24', 'RHO2_bin_30': 'RHO2_bin_30', 'IRC_PAM_SB0662_bin_9': 'IRC_PAM_SB0662_bin_9', 'IRC_PAM_SB0664_bin_21': 'IRC_PAM_SB0664_bin_21', 'CAR3_bin_12': 'CAR3_bin_12', 'IRC4_bin_38': 'IRC4_bin_38', 'CAR4_bin_3': 'CAR4_bin_3', 'RHO3_bin_34': 'RHO3_bin_34', 'COS36387_bin_4': 'COS36387_bin_4', 'IRC1_bin_12': 'IRC1_bin_12', 'COS4_bin_40': 'COS4_bin_40', 'RHO1_bin_19': 'RHO1_bin_19', 'COS4_bin_24': 'COS4_bin_24', 'COS36386_bin_17': 'COS36386_bin_17', 'IRC_PAM_SB0675_bin_10': 'IRC_PAM_SB0675_bin_10', 'APA_bin_73': 'APA_bin_73', 'RHO2_bin_1': 'RHO2_bin_1', 'COS36386_bin_27': 'COS36386_bin_27', 'LOPHE_tianQian_GCA_001542995.1_ASM154299v1_genomic': 'GCA_001542995', 'IRC_PAM_SB0665_bin_10': 'IRC_PAM_SB0665_bin_10', 'RHO3_bin_13': 'RHO3_bin_13', 'IRC_PAM_SB0665_bin_13': 'IRC_PAM_SB0665_bin_13', 'APA_bin_24': 'APA_bin_24', 'RHO3_bin_64': 'RHO3_bin_64', 'IRC_PAM_SB0668_bin_6': 'IRC_PAM_SB0668_bin_6', 'RHO1_bin_62': 'RHO1_bin_62', 'IRC_PAM_SB0661_bin_45': 'IRC_PAM_SB0661_bin_45', 'IRC_PAM_SB0662_bin_26': 'IRC_PAM_SB0662_bin_26', 'COS1_bin_19': 'COS1_bin_19', 'RHO3_bin_11': 'RHO3_bin_11', 'RHO1_bin_46': 'RHO1_bin_46', 'TED_braunBugni_GCF_002573675.1_ASM257367v1_genomic': 'GCF_002573675', 'RHO1_bin_14': 'RHO1_bin_14', 'IRC_PAM_SB0664_bin_31': 'IRC_PAM_SB0664_bin_31', 'IRC2_bin_6': 'IRC2_bin_6', 'COS1_bin_17': 'COS1_bin_17', 'IRC3_bin_19': 'IRC3_bin_19', 'RHO1_bin_45': 'RHO1_bin_45', 'CAR4_bin_8': 'CAR4_bin_8', 'RHO2_bin_65': 'RHO2_bin_65', 'RHO2_bin_9': 'RHO2_bin_9', 'seawater_22112_bin_5': 'seawater_22112_bin_5', 'IRC_PAM_SB0670_bin_12': 'IRC_PAM_SB0670_bin_12', 'CAR4_bin_10': 'CAR4_bin_10', 'RHO3_bin_16': 'RHO3_bin_16', 'IRC_PAM_SB0677_bin_2': 'IRC_PAM_SB0677_bin_2', 'IRC4_bin_6': 'IRC4_bin_6', 'RHO1_bin_48': 'RHO1_bin_48', 'IRC_PAM_SB0665_bin_1': 'IRC_PAM_SB0665_bin_1', 'APA_bin_14': 'APA_bin_14', 'COS4_bin_5': 'COS4_bin_5', 'IRC_PAM_SB0662_bin_20': 'IRC_PAM_SB0662_bin_20', 'COS1_bin_6': 'COS1_bin_6', 'IRC_PAM_SB0661_bin_4': 'IRC_PAM_SB0661_bin_4', 'RHO1_bin_1': 'RHO1_bin_1', 'RHO3_bin_31': 'RHO3_bin_31', 'RHO2_bin_51': 'RHO2_bin_51', 'HALC_tianQian2014_GCA_000583135.1_Thioalkalivibrio_spongium_HK1_genomic': 'GCA_000583135', 'IRC3_bin_31': 'IRC3_bin_31', 'IRC_PAM_SB0661_bin_37': 'IRC_PAM_SB0661_bin_37', 'CAR3_bin_6': 'CAR3_bin_6', 'COS36387_bin_6': 'COS36387_bin_6', 'RHO2_bin_37': 'RHO2_bin_37', 'COS4_bin_3': 'COS4_bin_3', 'COS3_bin_2': 'COS3_bin_2', 'CHO1_bin_1': 'CHO1_bin_1', 'COS4_bin_19': 'COS4_bin_19', 'IRC_PAM_SB0662_bin_33': 'IRC_PAM_SB0662_bin_33', 'THES_burgsdorfSteindler_GCA_001007665.1_ASM100766v1_genomic': 'GCA_001007665', 'IRC_PAM_SB0675_bin_19': 'IRC_PAM_SB0675_bin_19', 'COS36404_bin_1': 'COS36404_bin_1', 'RHO3_bin_58': 'RHO3_bin_58', 'CAR3_bin_1': 'CAR3_bin_1', 'PSE_podellAllen_GCA_003635195.1_ASM363519v1_genomic': 'GCA_003635195', 'IRC_PAM_SB0662_bin_5': 'IRC_PAM_SB0662_bin_5', 'IRC_PAM_SB0662_bin_22': 'IRC_PAM_SB0662_bin_22', 'APA_bin_26': 'APA_bin_26', 'CAR4_bin_18': 'CAR4_bin_18', 'COS36386_bin_19': 'COS36386_bin_19', 'IRC_PAM_SB0661_bin_41': 'IRC_PAM_SB0661_bin_41', 'IRC_PAM_SB0664_bin_25': 'IRC_PAM_SB0664_bin_25', 'IRC_PAM_SB0670_bin_20': 'IRC_PAM_SB0670_bin_20', 'SUB_tianQian_GCA_002007405.1_ASM200740v1_genomic': 'GCA_002007405', 'APA_bin_81': 'APA_bin_81', 'IRC_PAM_SB0676_bin_21': 'IRC_PAM_SB0676_bin_21', 'COS4_bin_43': 'COS4_bin_43', 'RHO2_bin_18': 'RHO2_bin_18', 'APA_bin_33': 'APA_bin_33', 'CAR2_bin_20': 'CAR2_bin_20', 'seawater_bettina_36309_bin_17': 'seawater_bettina_36309_bin_17', 'CAR2_bin_2': 'CAR2_bin_2', 'IRC_PAM_SB0664_bin_27': 'IRC_PAM_SB0664_bin_27', 'seawater_bettina_36310_bin_4': 'seawater_bettina_36310_bin_4', 'COS1_bin_20': 'COS1_bin_20', 'IRC_PAM_SB0662_bin_58': 'IRC_PAM_SB0662_bin_58', 'CAR1_bin_12': 'CAR1_bin_12', 'seawater_22112_bin_10': 'seawater_22112_bin_10', 'CAR1_bin_3': 'CAR1_bin_3', 'IRC4_bin_8': 'IRC4_bin_8', 'IRC_PAM_SB0675_bin_2': 'IRC_PAM_SB0675_bin_2', 'IRC_PAM_SB0665_bin_2': 'IRC_PAM_SB0665_bin_2', 'IRC_PAM_SB0670_bin_22': 'IRC_PAM_SB0670_bin_22', 'IRC_PAM_SB0664_bin_22': 'IRC_PAM_SB0664_bin_22', 'seawater_seasim_SB9152_S1_bin_1': 'seawater_seasim_SB9152_S1_bin_1', 'RHO3_bin_69': 'RHO3_bin_69', 'RHO1_bin_44': 'RHO1_bin_44', 'seawater_seasim_SB9153_S2_bin_2': 'seawater_seasim_SB9153_S2_bin_2', 'IRC_PAM_SB0666_bin_17': 'IRC_PAM_SB0666_bin_17', 'MELS_podellAllen_GCA_GCA_003635315.1_ASM363531v1_genomic': 'GCA_003635315', 'IRC_PAM_SB0661_bin_3': 'IRC_PAM_SB0661_bin_3', 'IRC_PAM_SB0673_bin_16': 'IRC_PAM_SB0673_bin_16', 'IRC_PAM_SB0662_bin_35': 'IRC_PAM_SB0662_bin_35', 'RHO3_bin_35': 'RHO3_bin_35', 'COS2_bin_17': 'COS2_bin_17', 'unknown_alexAntunes_GCF_003676335.1_ASM367633v1_genomic': 'GCF_003676335', 'IRC4_bin_23': 'IRC4_bin_23', 'PSE_podellAllen_GCA_003635255.1_ASM363525v1_genomic': 'GCA_003635255', 'IRC4_bin_24': 'IRC4_bin_24', 'seawater_22112_bin_28': 'seawater_22112_bin_28', 'CAR2_bin_7': 'CAR2_bin_7', 'APA_bin_94': 'APA_bin_94', 'APA_bin_85': 'APA_bin_85', 'IRC1_bin_17': 'IRC1_bin_17', 'COS36404_bin_9': 'COS36404_bin_9', 'COS36404_bin_2': 'COS36404_bin_2', 'CAR3_bin_16': 'CAR3_bin_16', 'IRC4_bin_44': 'IRC4_bin_44', 'seawater_22112_bin_3': 'seawater_22112_bin_3', 'CAR1_bin_11': 'CAR1_bin_11', 'APA_bin_17': 'APA_bin_17', 'RHO2_bin_59': 'RHO2_bin_59', 'seawater_seasim_SB9155_S4_bin_12': 'seawater_seasim_SB9155_S4_bin_12', 'RHO2_bin_33': 'RHO2_bin_33', 'TED_estevesThomas2016_Aquimarina_spAU474_2606217188': 'Aquimarina_spAU474', 'IRC1_bin_15': 'IRC1_bin_15', 'RHO2_bin_54': 'RHO2_bin_54', 'RHO3_bin_7': 'RHO3_bin_7', 'COS4_bin_44': 'COS4_bin_44', 'RHO3_bin_73': 'RHO3_bin_73', 'APA_bin_70': 'APA_bin_70', 'RHO2_bin_7': 'RHO2_bin_7', 'IRC_PAM_SB0664_bin_9': 'IRC_PAM_SB0664_bin_9', 'RHO3_bin_47': 'RHO3_bin_47', 'COS36387_bin_1': 'COS36387_bin_1', 'IRC3_bin_12': 'IRC3_bin_12', 'COS1_bin_12': 'COS1_bin_12', 'CAR4_bin_2': 'CAR4_bin_2', 'IRC4_bin_33': 'IRC4_bin_33', 'IRC_PAM_SB0666_bin_10': 'IRC_PAM_SB0666_bin_10', 'APA_bin_68': 'APA_bin_68', 'COS3_bin_18': 'COS3_bin_18', 'IRC_PAM_SB0678_bin_9': 'IRC_PAM_SB0678_bin_9', 'RHO1_bin_4': 'RHO1_bin_4', 'RHO3_bin_72': 'RHO3_bin_72', 'RHO1_bin_67': 'RHO1_bin_67', 'IRC_PAM_SB0661_bin_39': 'IRC_PAM_SB0661_bin_39', 'IRC4_bin_2': 'IRC4_bin_2', 'IRC_PAM_SB0676_bin_26': 'IRC_PAM_SB0676_bin_26', 'CAR4_bin_9': 'CAR4_bin_9', 'seawater_seasim_SB9160_S9_bin_11': 'seawater_seasim_SB9160_S9_bin_11', 'IRC_PAM_SB0675_bin_14': 'IRC_PAM_SB0675_bin_14', 'RHO1_bin_32': 'RHO1_bin_32', 'COS4_bin_2': 'COS4_bin_2', 'RHO2_bin_38': 'RHO2_bin_38', 'COS36387_bin_2': 'COS36387_bin_2', 'seawater_seasim_SB9155_S4_bin_4': 'seawater_seasim_SB9155_S4_bin_4', 'APA_bin_83': 'APA_bin_83', 'IRC_PAM_SB0661_bin_29': 'IRC_PAM_SB0661_bin_29', 'COS3_bin_12': 'COS3_bin_12', 'RHO3_bin_41': 'RHO3_bin_41', 'SUB_tianQian_GCA_001541925.1_ASM154192v1_genomic': 'GCA_001541925', 'RHO3_bin_38': 'RHO3_bin_38', 'STY4_bin_1': 'STY4_bin_1', 'IRC_PAM_SB0672_bin_11': 'IRC_PAM_SB0672_bin_11', 'STY3_bin_3': 'STY3_bin_3', 'IRC1_bin_1': 'IRC1_bin_1', 'IRC_PAM_SB0662_bin_12': 'IRC_PAM_SB0662_bin_12', 'seawater_seasim_SB9157_S6_bin_2': 'seawater_seasim_SB9157_S6_bin_2', 'RHO1_bin_40': 'RHO1_bin_40', 'IRC2_bin_8': 'IRC2_bin_8', 'seawater_22112_bin_14': 'seawater_22112_bin_14', 'seawater_42618_bin_2': 'seawater_42618_bin_2', 'COS4_bin_20': 'COS4_bin_20', 'IRC1_bin_3': 'IRC1_bin_3', 'RHO3_bin_66': 'RHO3_bin_66', 'CAR3_bin_18': 'CAR3_bin_18', 'COS1_bin_7': 'COS1_bin_7', 'CYMC_estevesThomas2016_Pseudovibrio_spAU243_2606217185': 'Pseudovibrio_spAU243', 'CYMC_moitinhoThomas_67496.assembled': 'CYMC_moitinhoThomas_67496', 'RHO2_bin_23': 'RHO2_bin_23', 'IRC1_bin_16': 'IRC1_bin_16', 'IRC1_bin_35': 'IRC1_bin_35', 'CAR2_bin_6': 'CAR2_bin_6', 'IRC3_bin_25': 'IRC3_bin_25', 'IRC1_bin_10': 'IRC1_bin_10', 'COS36404_bin_19': 'COS36404_bin_19', 'RHO3_bin_12': 'RHO3_bin_12', 'CAR3_bin_3': 'CAR3_bin_3', 'IRC4_bin_37': 'IRC4_bin_37', 'IRC_PAM_SB0662_bin_49': 'IRC_PAM_SB0662_bin_49', 'COS1_bin_1': 'COS1_bin_1', 'IRC_PAM_SB0662_bin_59': 'IRC_PAM_SB0662_bin_59', 'RHO3_bin_6': 'RHO3_bin_6', 'CAR2_bin_14': 'CAR2_bin_14', 'RHO3_bin_48': 'RHO3_bin_48', 'RHO3_bin_37': 'RHO3_bin_37', 'SPOO_karimiCosta2019_GCF_900143635.1_Rhodobacteraceae_bacterium_Alg231_04_genomic': 'GCF_900143635', 'COS4_bin_14': 'COS4_bin_14', 'APA_bin_4': 'APA_bin_4', 'RHO2_bin_24': 'RHO2_bin_24', 'APA_bin_82': 'APA_bin_82', 'APA_bin_38': 'APA_bin_38', 'MYCL_zanHill_GCF_000158135.1_ASM15813v1_genomic': 'GCF_000158135', 'PSE_podellAllen_GCA_003635265.1_ASM363526v1_genomic': 'GCA_003635265', 'IRC_PAM_SB0675_bin_20': 'IRC_PAM_SB0675_bin_20', 'RHO3_bin_5': 'RHO3_bin_5', 'IRC_PAM_SB0661_bin_38': 'IRC_PAM_SB0661_bin_38', 'COS3_bin_5': 'COS3_bin_5', 'RHO3_bin_10': 'RHO3_bin_10', 'CAR2_bin_9': 'CAR2_bin_9', 'IRC_PAM_SB0661_bin_15': 'IRC_PAM_SB0661_bin_15', 'APA_bin_90': 'APA_bin_90', 'SPOO_karimiCosta2019_GCF_900143555.1_Labrenzia_sp._Alg231_36_genomic': 'GCF_900143555', 'IRC_PAM_SB0670_bin_18': 'IRC_PAM_SB0670_bin_18', 'APA_bin_87': 'APA_bin_87', 'COS36405_bin_7': 'COS36405_bin_7', 'RHO2_bin_64': 'RHO2_bin_64', 'STY4_bin_2': 'STY4_bin_2', 'IRC2_bin_2': 'IRC2_bin_2', 'STY4_bin_9': 'STY4_bin_9', 'APA_bin_58': 'APA_bin_58', 'COS4_bin_32': 'COS4_bin_32', 'seawater_seasim_SB9156_S5_bin_8': 'seawater_seasim_SB9156_S5_bin_8', 'COS2_bin_22': 'COS2_bin_22', 'COS36405_bin_20': 'COS36405_bin_20', 'IRC_PAM_SB0662_bin_51': 'IRC_PAM_SB0662_bin_51', 'SPOO_karimiCosta2019_GCF_900143615.1_Rhodobacteraceae_bacterium_Alg231_30_genomic': 'GCF_900143615', 'POLP_alexAntunes_GCF_001431305.1_ASM143130v1_genomic': 'GCF_001431305', 'RHO3_bin_54': 'RHO3_bin_54', 'IRC_PAM_SB0666_bin_21': 'IRC_PAM_SB0666_bin_21', 'SPOO_karimiCosta2019_GCF_900149695.1_Anderseniella_sp._Alg231_50_genomic': 'GCF_900149695', 'RHO2_bin_3': 'RHO2_bin_3', 'seawater_42618_bin_25': 'seawater_42618_bin_25', 'RHO2_bin_57': 'RHO2_bin_57', 'seawater_bettina_36328_bin_2': 'seawater_bettina_36328_bin_2', 'COS4_bin_11': 'COS4_bin_11', 'COS36387_bin_22': 'COS36387_bin_22', 'IRC1_bin_6': 'IRC1_bin_6', 'COS36386_bin_3': 'COS36386_bin_3', 'RHO2_bin_20': 'RHO2_bin_20', 'SPOO_karimiCosta2019_GCF_900149705.1_Sphingorhabdus_sp._Alg231_15_genomic': 'GCF_900149705', 'IRC_PAM_SB0661_bin_43': 'IRC_PAM_SB0661_bin_43', 'CAR4_bin_15': 'CAR4_bin_15', 'IRC1_bin_7': 'IRC1_bin_7', 'IRC_PAM_SB0661_bin_8': 'IRC_PAM_SB0661_bin_8', 'seawater_bettina_36328_bin_5': 'seawater_bettina_36328_bin_5', 'STY2_bin_4': 'STY2_bin_4', 'IRC_PAM_SB0664_bin_10': 'IRC_PAM_SB0664_bin_10', 'COS4_bin_16': 'COS4_bin_16', 'RHO1_bin_60': 'RHO1_bin_60', 'IRC_PAM_SB0668_bin_20': 'IRC_PAM_SB0668_bin_20', 'seawater_bettina_36327_bin_2': 'seawater_bettina_36327_bin_2', 'RHO2_bin_16': 'RHO2_bin_16', 'RHO3_bin_79': 'RHO3_bin_79', 'CAR1_bin_13': 'CAR1_bin_13', 'COS1_bin_15': 'COS1_bin_15', 'IRC_PAM_SB0666_bin_1': 'IRC_PAM_SB0666_bin_1', 'CAR1_bin_2': 'CAR1_bin_2', 'IRC_PAM_SB0664_bin_3': 'IRC_PAM_SB0664_bin_3', 'COS4_bin_29': 'COS4_bin_29', 'IRC_PAM_SB0670_bin_1': 'IRC_PAM_SB0670_bin_1', 'IRC1_bin_18': 'IRC1_bin_18', 'IRC4_bin_22': 'IRC4_bin_22', 'IRC2_bin_13': 'IRC2_bin_13', 'APA_bin_18': 'APA_bin_18', 'COS36406_bin_4': 'COS36406_bin_4', 'IRC1_bin_11': 'IRC1_bin_11', 'seawater_bettina_36327_bin_15': 'seawater_bettina_36327_bin_15', 'COS1_bin_2': 'COS1_bin_2', 'seawater_seasim_SB9154_S3_bin_4': 'seawater_seasim_SB9154_S3_bin_4', 'COS36386_bin_8': 'COS36386_bin_8', 'RHO1_bin_49': 'RHO1_bin_49', 'IRC1_bin_4': 'IRC1_bin_4', 'IRC_PAM_SB0662_bin_15': 'IRC_PAM_SB0662_bin_15', 'IRC4_bin_20': 'IRC4_bin_20', 'seawater_bettina_36326_bin_1': 'seawater_bettina_36326_bin_1', 'IRC_PAM_SB0661_bin_30': 'IRC_PAM_SB0661_bin_30', 'RHO2_bin_11': 'RHO2_bin_11', 'IRC_PAM_SB0662_bin_37': 'IRC_PAM_SB0662_bin_37', 'RHO3_bin_2': 'RHO3_bin_2', 'COS36386_bin_18': 'COS36386_bin_18', 'IRC_PAM_SB0661_bin_24': 'IRC_PAM_SB0661_bin_24', 'seawater_bettina_36326_bin_26': 'seawater_bettina_36326_bin_26', 'IRC_PAM_SB0664_bin_15': 'IRC_PAM_SB0664_bin_15', 'COS36406_bin_19': 'COS36406_bin_19', 'COS36386_bin_10': 'COS36386_bin_10', 'IRC_PAM_SB0663_bin_5': 'IRC_PAM_SB0663_bin_5', 'IRC_PAM_SB0662_bin_21': 'IRC_PAM_SB0662_bin_21', 'COS2_bin_16': 'COS2_bin_16', 'IRC3_bin_20': 'IRC3_bin_20', 'IRC_PAM_SB0661_bin_44': 'IRC_PAM_SB0661_bin_44', 'IRC4_bin_25': 'IRC4_bin_25', 'CAR2_bin_11': 'CAR2_bin_11', 'APA_bin_72': 'APA_bin_72', 'COS4_bin_8': 'COS4_bin_8', 'seawater_seasim_SB9155_S4_bin_5': 'seawater_seasim_SB9155_S4_bin_5', 'IRC_PAM_SB0667_bin_9': 'IRC_PAM_SB0667_bin_9', 'STY2_bin_6': 'STY2_bin_6', 'STY3_bin_1': 'STY3_bin_1', 'IRC4_bin_1': 'IRC4_bin_1', 'seawater_bettina_36327_bin_4': 'seawater_bettina_36327_bin_4', 'RHO1_bin_58': 'RHO1_bin_58', 'IRC_PAM_SB0661_bin_33': 'IRC_PAM_SB0661_bin_33', 'IRC3_bin_5': 'IRC3_bin_5', 'seawater_seasim_SB9156_S5_bin_14': 'seawater_seasim_SB9156_S5_bin_14', 'IRC1_bin_22': 'IRC1_bin_22', 'COS36388_bin_4': 'COS36388_bin_4', 'RHO1_bin_9': 'RHO1_bin_9', 'RHO1_bin_66': 'RHO1_bin_66', 'APA_bin_43': 'APA_bin_43', 'RHO2_bin_28': 'RHO2_bin_28', 'seawater_seasim_SB9156_S5_bin_4': 'seawater_seasim_SB9156_S5_bin_4', 'RHO3_bin_21': 'RHO3_bin_21', 'IRC1_bin_2': 'IRC1_bin_2', 'IRC_PAM_SB0678_bin_15': 'IRC_PAM_SB0678_bin_15', 'CLI1_bin_4': 'CLI1_bin_4', 'IRC1_bin_25': 'IRC1_bin_25', 'COS36388_bin_18': 'COS36388_bin_18', 'IRC_PAM_SB0666_bin_22': 'IRC_PAM_SB0666_bin_22', 'IRC_PAM_SB0661_bin_11': 'IRC_PAM_SB0661_bin_11', 'CAR2_bin_4': 'CAR2_bin_4', 'CLI4_bin_1': 'CLI4_bin_1', 'IRC_PAM_SB0677_bin_8': 'IRC_PAM_SB0677_bin_8', 'RHO2_bin_53': 'RHO2_bin_53', 'seawater_seasim_SB9158_S7_bin_9': 'seawater_seasim_SB9158_S7_bin_9', 'COS36387_bin_18': 'COS36387_bin_18', 'seawater_42618_bin_5': 'seawater_42618_bin_5', 'COS36386_bin_29': 'COS36386_bin_29', 'RHO3_bin_51': 'RHO3_bin_51', 'RHO1_bin_72': 'RHO1_bin_72', 'COS3_bin_11': 'COS3_bin_11', 'RHO1_bin_6': 'RHO1_bin_6', 'IRC4_bin_4': 'IRC4_bin_4', 'STY2_bin_7': 'STY2_bin_7', 'RHO2_bin_35': 'RHO2_bin_35', 'RHO3_bin_20': 'RHO3_bin_20', 'APA_bin_45': 'APA_bin_45', 'IRC4_bin_35': 'IRC4_bin_35', 'COS36387_bin_9': 'COS36387_bin_9', 'IRC3_bin_22': 'IRC3_bin_22', 'RHO3_bin_26': 'RHO3_bin_26', 'CAR2_bin_26': 'CAR2_bin_26', 'CAR3_bin_13': 'CAR3_bin_13', 'IRC3_bin_7': 'IRC3_bin_7', 'RHO1_bin_30': 'RHO1_bin_30', 'RHO3_bin_14': 'RHO3_bin_14', 'COS36386_bin_6': 'COS36386_bin_6', 'RHO2_bin_43': 'RHO2_bin_43', 'IRC_PAM_SB0664_bin_5': 'IRC_PAM_SB0664_bin_5', 'seawater_bettina_36327_bin_6': 'seawater_bettina_36327_bin_6', 'RHO1_bin_2': 'RHO1_bin_2', 'IRC3_bin_10': 'IRC3_bin_10', 'APA_burgsdorfSteindler_GCA_001007635.1_ASM100763v1_genomic': 'GCA_001007635', 'RHO3_bin_18': 'RHO3_bin_18', 'APA_bin_7': 'APA_bin_7', 'COS1_bin_10': 'COS1_bin_10', 'IRC_PAM_SB0668_bin_27': 'IRC_PAM_SB0668_bin_27', 'RHO2_bin_12': 'RHO2_bin_12', 'COS36386_bin_4': 'COS36386_bin_4', 'IRC_PAM_SB0661_bin_32': 'IRC_PAM_SB0661_bin_32', 'RHO1_bin_27': 'RHO1_bin_27', 'THES_lavyIlan_GCA_002631715.1_ASM263171v1_genomic': 'GCA_002631715', 'IRC_PAM_SB0666_bin_13': 'IRC_PAM_SB0666_bin_13', 'STY2_bin_2': 'STY2_bin_2', 'RHO3_bin_17': 'RHO3_bin_17', 'seawater_seasim_SB9152_S1_bin_19': 'seawater_seasim_SB9152_S1_bin_19', 'RHO2_bin_60': 'RHO2_bin_60', 'RHO3_bin_9': 'RHO3_bin_9', 'STY4_bin_8': 'STY4_bin_8', 'seawater_42617_bin_1': 'seawater_42617_bin_1', 'COS36386_bin_35': 'COS36386_bin_35', 'COS2_bin_12': 'COS2_bin_12', 'seawater_22112_bin_12': 'seawater_22112_bin_12', 'seawater_seasim_SB9152_S1_bin_12': 'seawater_seasim_SB9152_S1_bin_12', 'CAR2_bin_8': 'CAR2_bin_8', 'CAR3_bin_5': 'CAR3_bin_5', 'COS4_bin_34': 'COS4_bin_34', 'IRC1_bin_38': 'IRC1_bin_38', 'IRC4_bin_45': 'IRC4_bin_45', 'STY4_bin_7': 'STY4_bin_7', 'IRC_PAM_SB0675_bin_12': 'IRC_PAM_SB0675_bin_12', 'COS4_bin_4': 'COS4_bin_4', 'CAR3_bin_7': 'CAR3_bin_7', 'IRC_PAM_SB0670_bin_39': 'IRC_PAM_SB0670_bin_39', 'IRC_PAM_SB0667_bin_3': 'IRC_PAM_SB0667_bin_3', 'IRC_PAM_SB0678_bin_6': 'IRC_PAM_SB0678_bin_6', 'COS36404_bin_8': 'COS36404_bin_8', 'seawater_22112_bin_1': 'seawater_22112_bin_1', 'CAR4_bin_14': 'CAR4_bin_14', 'COS36388_bin_5': 'COS36388_bin_5', 'seawater_22112_bin_17': 'seawater_22112_bin_17', 'COS36388_bin_17': 'COS36388_bin_17', 'COS36386_bin_9': 'COS36386_bin_9', 'APA_bin_39': 'APA_bin_39', 'COS36405_bin_11': 'COS36405_bin_11', 'RHO2_bin_50': 'RHO2_bin_50', 'RHO2_bin_26': 'RHO2_bin_26', 'IRC3_bin_35': 'IRC3_bin_35', 'RHO1_bin_7': 'RHO1_bin_7', 'IRC3_bin_14': 'IRC3_bin_14', 'RHO1_bin_21': 'RHO1_bin_21', 'SPOO_karimiCosta2019_GCF_900149685.1_Erythrobacter_sp._Alg231_14_genomic': 'GCF_900149685', 'IRC_PAM_SB0675_bin_18': 'IRC_PAM_SB0675_bin_18', 'APA_bin_15': 'APA_bin_15', 'APA_bin_86': 'APA_bin_86', 'seawater_bettina_36326_bin_7': 'seawater_bettina_36326_bin_7', 'IRC_PAM_SB0665_bin_8': 'IRC_PAM_SB0665_bin_8', 'RHO1_bin_50': 'RHO1_bin_50', 'RHO3_bin_15': 'RHO3_bin_15', 'COS36406_bin_6': 'COS36406_bin_6', 'IRC_PAM_SB0661_bin_14': 'IRC_PAM_SB0661_bin_14', 'SPOO_karimiCosta2019_GCF_900143565.1_Pseudovibrio_sp._Alg231_02_genomic': 'GCF_900143565', 'COS4_bin_51': 'COS4_bin_51', 'IRC_PAM_SB0662_bin_53': 'IRC_PAM_SB0662_bin_53', 'COS3_bin_10': 'COS3_bin_10', 'AMPF_kennedyDobson_GCF_900109375.1_IMGID2622736580_genomic': 'GCF_900109375', 'THES_wilsonPiel_GCA_000522425.1_v3_genomic': 'GCA_000522425', 'RHO1_bin_70': 'RHO1_bin_70', 'IRC_PAM_SB0677_bin_14': 'IRC_PAM_SB0677_bin_14', 'COS4_bin_13': 'COS4_bin_13', 'APA_bin_96': 'APA_bin_96', 'COS36404_bin_10': 'COS36404_bin_10', 'STY4_bin_4': 'STY4_bin_4', 'COS2_bin_2': 'COS2_bin_2', 'RHO3_bin_52': 'RHO3_bin_52', 'COS4_bin_27': 'COS4_bin_27', 'APA_bin_41': 'APA_bin_41', 'IRC_PAM_SB0665_bin_5': 'IRC_PAM_SB0665_bin_5', 'COS4_bin_17': 'COS4_bin_17', 'SPOO_karimiCosta2019_GCF_900143525.1_Ruegeria_sp._Alg231_54_genomic': 'GCF_900143525', 'COS36388_bin_9': 'COS36388_bin_9', 'IRC_PAM_SB0661_bin_21': 'IRC_PAM_SB0661_bin_21', 'IRC_PAM_SB0662_bin_8': 'IRC_PAM_SB0662_bin_8', 'IRC_PAM_SB0664_bin_14': 'IRC_PAM_SB0664_bin_14', 'COS36386_bin_20': 'COS36386_bin_20', 'APA_bin_29': 'APA_bin_29', 'IRC4_bin_12': 'IRC4_bin_12', 'COS1_bin_13': 'COS1_bin_13', 'IRC_PAM_SB0675_bin_29': 'IRC_PAM_SB0675_bin_29', 'COS36387_bin_16': 'COS36387_bin_16', 'IRC_PAM_SB0661_bin_26': 'IRC_PAM_SB0661_bin_26', 'COS4_bin_55': 'COS4_bin_55', 'IRC_PAM_SB0668_bin_12': 'IRC_PAM_SB0668_bin_12', 'APA_bin_98': 'APA_bin_98', 'IRC_PAM_SB0665_bin_12': 'IRC_PAM_SB0665_bin_12', 'IRC3_bin_11': 'IRC3_bin_11', 'APA_bin_55': 'APA_bin_55', 'seawater_seasim_SB9152_S1_bin_4': 'seawater_seasim_SB9152_S1_bin_4', 'COS36386_bin_12': 'COS36386_bin_12', 'seawater_bettina_36328_bin_30': 'seawater_bettina_36328_bin_30', 'COS36388_bin_8': 'COS36388_bin_8', 'COS2_bin_5': 'COS2_bin_5', 'RHO1_bin_81': 'RHO1_bin_81', 'CLI4_bin_2': 'CLI4_bin_2', 'APA_bin_63': 'APA_bin_63', 'SPOO_karimiCosta_FZLS01': 'SPOO_karimiCosta_FZLS01', 'APA_bin_69': 'APA_bin_69', 'IRC4_bin_21': 'IRC4_bin_21', 'IRC_PAM_SB0666_bin_6': 'IRC_PAM_SB0666_bin_6', 'RHO2_bin_52': 'RHO2_bin_52', 'COS3_bin_3': 'COS3_bin_3', 'RHO1_bin_33': 'RHO1_bin_33', 'COS1_bin_5': 'COS1_bin_5', 'seawater_bettina_36309_bin_3': 'seawater_bettina_36309_bin_3', 'RHO2_bin_10': 'RHO2_bin_10', 'COS2_bin_23': 'COS2_bin_23', 'RHO1_bin_42': 'RHO1_bin_42', 'PER4_bin_1': 'PER4_bin_1', 'APA_bin_48': 'APA_bin_48', 'RHO3_bin_27': 'RHO3_bin_27', 'seawater_seasim_SB9153_S2_bin_10': 'seawater_seasim_SB9153_S2_bin_10', 'IRC_PAM_SB0661_bin_19': 'IRC_PAM_SB0661_bin_19', 'RHO1_bin_34': 'RHO1_bin_34', 'IRC3_bin_6': 'IRC3_bin_6', 'SPOO_karimiCosta2019_GCF_900143545.1_Loktanella_sp._Alg231_35_genomic': 'GCF_900143545', 'COS36386_bin_15': 'COS36386_bin_15', 'CAR3_bin_4': 'CAR3_bin_4', 'APA_bin_32': 'APA_bin_32', 'CAR1_bin_6': 'CAR1_bin_6', 'RHO1_bin_15': 'RHO1_bin_15', 'RHO3_bin_39': 'RHO3_bin_39', 'IRC1_bin_30': 'IRC1_bin_30', 'RHO1_bin_11': 'RHO1_bin_11', 'MYCL_bondarevVogt_GCF_000156235.1_ASM15623v1_genomic': 'GCF_000156235', 'IRC_PAM_SB0664_bin_24': 'IRC_PAM_SB0664_bin_24', 'IRC_PAM_SB0662_bin_36': 'IRC_PAM_SB0662_bin_36', 'IRC_PAM_SB0661_bin_31': 'IRC_PAM_SB0661_bin_31', 'IRC3_bin_32': 'IRC3_bin_32', 'COS36406_bin_13': 'COS36406_bin_13', 'IRC2_bin_4': 'IRC2_bin_4', 'IRC3_bin_4': 'IRC3_bin_4', 'COS36386_bin_11': 'COS36386_bin_11', 'COS2_bin_18': 'COS2_bin_18', 'COS36405_bin_5': 'COS36405_bin_5', 'seawater_bettina_36326_bin_6': 'seawater_bettina_36326_bin_6', 'IRC3_bin_9': 'IRC3_bin_9', 'APA_bin_40': 'APA_bin_40', 'seawater_seasim_SB9157_S6_bin_13': 'seawater_seasim_SB9157_S6_bin_13', 'CAR3_bin_2': 'CAR3_bin_2', 'seawater_42616_bin_2': 'seawater_42616_bin_2', 'RHO1_bin_39': 'RHO1_bin_39', 'RHO1_bin_64': 'RHO1_bin_64', 'IRC1_bin_37': 'IRC1_bin_37', 'IRC_PAM_SB0662_bin_28': 'IRC_PAM_SB0662_bin_28', 'COS4_bin_22': 'COS4_bin_22', 'COS36405_bin_14': 'COS36405_bin_14', 'COS4_bin_47': 'COS4_bin_47', 'AXIM_hallam_GCA_000200715.1_genomic': 'GCA_000200715', 'RHO1_bin_28': 'RHO1_bin_28', 'IRC_PAM_SB0668_bin_11': 'IRC_PAM_SB0668_bin_11', 'STY1_bin_8': 'STY1_bin_8', 'RHO1_bin_22': 'RHO1_bin_22', 'COS4_bin_28': 'COS4_bin_28', 'APA_bin_12': 'APA_bin_12', 'seawater_42615_bin_2': 'seawater_42615_bin_2', 'COS36405_bin_2': 'COS36405_bin_2', 'COS36405_bin_10': 'COS36405_bin_10', 'COS36386_bin_28': 'COS36386_bin_28', 'RHO3_bin_70': 'RHO3_bin_70', 'seawater_bettina_36309_bin_6': 'seawater_bettina_36309_bin_6', 'IRC_PAM_SB0667_bin_2': 'IRC_PAM_SB0667_bin_2', 'APA_bin_34': 'APA_bin_34', 'IRC_PAM_SB0665_bin_20': 'IRC_PAM_SB0665_bin_20', 'IRC1_bin_23': 'IRC1_bin_23', 'COS3_bin_1': 'COS3_bin_1', 'RHO2_bin_40': 'RHO2_bin_40', 'COS4_bin_10': 'COS4_bin_10', 'COS36387_bin_10': 'COS36387_bin_10', 'IRC3_bin_8': 'IRC3_bin_8', 'RHO3_bin_50': 'RHO3_bin_50', 'IRC_PAM_SB0666_bin_15': 'IRC_PAM_SB0666_bin_15', 'COS36387_bin_7': 'COS36387_bin_7', 'COS4_bin_26': 'COS4_bin_26', 'RHO2_bin_41': 'RHO2_bin_41', 'APA_bin_71': 'APA_bin_71', 'COS1_bin_18': 'COS1_bin_18', 'COS36406_bin_18': 'COS36406_bin_18', 'IRC_PAM_SB0662_bin_19': 'IRC_PAM_SB0662_bin_19', 'APA_bin_53': 'APA_bin_53', 'IRC_PAM_SB0666_bin_9': 'IRC_PAM_SB0666_bin_9', 'RHO2_bin_8': 'RHO2_bin_8', 'RHO2_bin_31': 'RHO2_bin_31', 'STY1_bin_1': 'STY1_bin_1', 'APA_bin_56': 'APA_bin_56', 'TED_estevesThomas2016_Ruegeria_spAU67_2606217183': 'Ruegeria_spAU67', 'APA_bin_89': 'APA_bin_89', 'RHO2_bin_62': 'RHO2_bin_62', 'AREB_froesThompson_GCF_001941685.1_ASM194168v1_genomic': 'GCF_001941685', 'COS2_bin_8': 'COS2_bin_8', 'seawater_bettina_36328_bin_7': 'seawater_bettina_36328_bin_7', 'IRC4_bin_40': 'IRC4_bin_40', 'COS3_bin_14': 'COS3_bin_14', 'IRC_PAM_SB0668_bin_14': 'IRC_PAM_SB0668_bin_14', 'COS4_bin_25': 'COS4_bin_25', 'RHO1_bin_8': 'RHO1_bin_8', 'IRC_PAM_SB0664_bin_12': 'IRC_PAM_SB0664_bin_12', 'COS1_bin_3': 'COS1_bin_3', 'IRC4_bin_15': 'IRC4_bin_15', 'CAR2_bin_1': 'CAR2_bin_1', 'IRC3_bin_15': 'IRC3_bin_15', 'CAR1_bin_18': 'CAR1_bin_18', 'RHO1_bin_12': 'RHO1_bin_12', 'IRC_PAM_SB0665_bin_11': 'IRC_PAM_SB0665_bin_11', 'RHO2_bin_22': 'RHO2_bin_22', 'RHO1_bin_20': 'RHO1_bin_20', 'STY4_bin_3': 'STY4_bin_3', 'IRC4_bin_9': 'IRC4_bin_9', 'STY1_bin_3': 'STY1_bin_3', 'COS3_bin_4': 'COS3_bin_4', 'seawater_seasim_SB9156_S5_bin_20': 'seawater_seasim_SB9156_S5_bin_20', 'RHO2_bin_25': 'RHO2_bin_25', 'IRC_PAM_SB0667_bin_14': 'IRC_PAM_SB0667_bin_14', 'COS3_bin_17': 'COS3_bin_17', 'IRC_PAM_SB0662_bin_23': 'IRC_PAM_SB0662_bin_23', 'seawater_seasim_SB9157_S6_bin_7': 'seawater_seasim_SB9157_S6_bin_7', 'RHO3_bin_8': 'RHO3_bin_8', 'seawater_22112_bin_8': 'seawater_22112_bin_8', 'IRC_PAM_SB0664_bin_33': 'IRC_PAM_SB0664_bin_33', 'COS36386_bin_37': 'COS36386_bin_37', 'RHO2_bin_5': 'RHO2_bin_5', 'RHO2_bin_19': 'RHO2_bin_19', 'IRC_PAM_SB0662_bin_24': 'IRC_PAM_SB0662_bin_24', 'seawater_42618_bin_9': 'seawater_42618_bin_9', 'IRC4_bin_49': 'IRC4_bin_49', 'IRC_PAM_SB0668_bin_21': 'IRC_PAM_SB0668_bin_21', 'APA_bin_28': 'APA_bin_28', 'COS36406_bin_2': 'COS36406_bin_2', 'APA_bin_61': 'APA_bin_61', 'IRC_PAM_SB0678_bin_2': 'IRC_PAM_SB0678_bin_2', 'IRC_PAM_SB0665_bin_19': 'IRC_PAM_SB0665_bin_19', 'IRC_PAM_SB0664_bin_11': 'IRC_PAM_SB0664_bin_11', 'seawater_seasim_SB9152_S1_bin_2': 'seawater_seasim_SB9152_S1_bin_2', 'seawater_22112_bin_9': 'seawater_22112_bin_9', 'RHO3_bin_33': 'RHO3_bin_33', 'IRC4_bin_39': 'IRC4_bin_39', 'SPOO_karimiCosta_FZLR01': 'SPOO_karimiCosta_FZLR01', 'RHO1_bin_82': 'RHO1_bin_82', 'CAR4_bin_13': 'CAR4_bin_13', 'IRC3_bin_17': 'IRC3_bin_17', 'CAR2_bin_22': 'CAR2_bin_22', 'SPOO_karimiCosta_FZLQ01': 'SPOO_karimiCosta_FZLQ01', 'TED_estevesThomas2016_Aquimarina_spAU119_2606217184': 'Aquimarina_spAU119', 'IRC_PAM_SB0675_bin_22': 'IRC_PAM_SB0675_bin_22', 'IRC_PAM_SB0664_bin_6': 'IRC_PAM_SB0664_bin_6', 'APA_bin_1': 'APA_bin_1', 'RHO1_bin_51': 'RHO1_bin_51', 'COS36386_bin_31': 'COS36386_bin_31', 'IRC2_bin_12': 'IRC2_bin_12', 'APA_bin_74': 'APA_bin_74', 'IRC_PAM_SB0661_bin_34': 'IRC_PAM_SB0661_bin_34', 'APA_bin_8': 'APA_bin_8', 'COS36406_bin_1': 'COS36406_bin_1', 'RHO3_bin_4': 'RHO3_bin_4', 'CAR4_bin_4': 'CAR4_bin_4', 'RHO2_bin_27': 'RHO2_bin_27', 'COS36406_bin_9': 'COS36406_bin_9', 'COS36388_bin_1': 'COS36388_bin_1', 'IRC2_bin_1': 'IRC2_bin_1', 'HALO_yoonOh_GCF_900141785.1_IMGID2622736502_genomic': 'GCF_900141785', 'COS36388_bin_16': 'COS36388_bin_16', 'APA_bin_23': 'APA_bin_23', 'APA_bin_62': 'APA_bin_62', 'CAR1_bin_16': 'CAR1_bin_16', 'IRC2_bin_7': 'IRC2_bin_7', 'CAR1_bin_4': 'CAR1_bin_4', 'IRC1_bin_19': 'IRC1_bin_19', 'CAR3_bin_9': 'CAR3_bin_9', 'COS4_bin_49': 'COS4_bin_49', 'IRC3_bin_37': 'IRC3_bin_37', 'CAR3_bin_15': 'CAR3_bin_15', 'RHO1_bin_55': 'RHO1_bin_55', 'RHO1_bin_59': 'RHO1_bin_59', 'IRC_PAM_SB0661_bin_1': 'IRC_PAM_SB0661_bin_1', 'CAR3_bin_10': 'CAR3_bin_10', 'COS4_bin_12': 'COS4_bin_12', 'seawater_seasim_SB9152_S1_bin_18': 'seawater_seasim_SB9152_S1_bin_18', 'IRC4_bin_17': 'IRC4_bin_17', 'SPOO_karimiCosta2019_GCF_900143535.1_Tateyamaria_sp._Alg231_49_genomic': 'GCF_900143535', 'CAR1_bin_5': 'CAR1_bin_5', 'RHO3_bin_22': 'RHO3_bin_22', 'IRC_PAM_SB0675_bin_3': 'IRC_PAM_SB0675_bin_3', 'seawater_seasim_SB9154_S3_bin_1': 'seawater_seasim_SB9154_S3_bin_1', 'COS4_bin_1': 'COS4_bin_1', 'IRCvar_burgsdorfSteindler_GCA_001007625.1_ASM100762v1_genomic': 'GCA_001007625', 'IRC_PAM_SB0661_bin_40': 'IRC_PAM_SB0661_bin_40', 'RHO1_bin_52': 'RHO1_bin_52', 'IRC4_bin_30': 'IRC4_bin_30', 'RHO1_bin_17': 'RHO1_bin_17', 'COS3_bin_16': 'COS3_bin_16', 'seawater_42615_bin_16': 'seawater_42615_bin_16', 'LOPHE_tianQian_GCA_001543005.1_ASM154300v1_genomic': 'GCA_001543005', 'RHO3_bin_19': 'RHO3_bin_19', 'CLI1_bin_2': 'CLI1_bin_2', 'IRC1_bin_31': 'IRC1_bin_31', 'IRC_PAM_SB0665_bin_17': 'IRC_PAM_SB0665_bin_17', 'seawater_bettina_36327_bin_3': 'seawater_bettina_36327_bin_3', 'COS36387_bin_14': 'COS36387_bin_14', 'COS4_bin_9': 'COS4_bin_9', 'seawater_bettina_36308_bin_18': 'seawater_bettina_36308_bin_18', 'COS1_bin_16': 'COS1_bin_16', 'RHO2_bin_17': 'RHO2_bin_17', 'COS36404_bin_5': 'COS36404_bin_5', 'STY1_bin_7': 'STY1_bin_7', 'IRC_PAM_SB0662_bin_30': 'IRC_PAM_SB0662_bin_30', 'RHO2_bin_2': 'RHO2_bin_2', 'IRC_PAM_SB0662_bin_1': 'IRC_PAM_SB0662_bin_1', 'RHO3_bin_29': 'RHO3_bin_29', 'COS36388_bin_3': 'COS36388_bin_3', 'seawater_seasim_SB9154_S3_bin_3': 'seawater_seasim_SB9154_S3_bin_3', 'IRC_PAM_SB0664_bin_7': 'IRC_PAM_SB0664_bin_7', 'COS36387_bin_15': 'COS36387_bin_15', 'IRC4_bin_11': 'IRC4_bin_11', 'IRC4_bin_42': 'IRC4_bin_42', 'IRC4_bin_13': 'IRC4_bin_13', 'RHO1_bin_43': 'RHO1_bin_43', 'CAR2_bin_19': 'CAR2_bin_19', 'IRC1_bin_9': 'IRC1_bin_9', 'IRC3_bin_3': 'IRC3_bin_3', 'IRC_PAM_SB0675_bin_23': 'IRC_PAM_SB0675_bin_23', 'seawater_seasim_SB9156_S5_bin_3': 'seawater_seasim_SB9156_S5_bin_3', 'RHO3_bin_57': 'RHO3_bin_57', 'IRC_PAM_SB0662_bin_34': 'IRC_PAM_SB0662_bin_34', 'CAR3_bin_17': 'CAR3_bin_17', 'IRC_PAM_SB0672_bin_21': 'IRC_PAM_SB0672_bin_21', 'RHO2_bin_39': 'RHO2_bin_39', 'COS4_bin_18': 'COS4_bin_18', 'IRC_PAM_SB0668_bin_13': 'IRC_PAM_SB0668_bin_13', 'seawater_22112_bin_4': 'seawater_22112_bin_4', 'IRC_PAM_SB0665_bin_16': 'IRC_PAM_SB0665_bin_16', 'COS3_bin_15': 'COS3_bin_15', 'RHO1_bin_69': 'RHO1_bin_69', 'RHO1_bin_26': 'RHO1_bin_26', 'COS36404_bin_6': 'COS36404_bin_6', 'COS36386_bin_5': 'COS36386_bin_5', 'seawater_seasim_SB9153_S2_bin_13': 'seawater_seasim_SB9153_S2_bin_13', 'RHO2_bin_56': 'RHO2_bin_56', 'APA_bin_6': 'APA_bin_6', 'IRC_PAM_SB0661_bin_6': 'IRC_PAM_SB0661_bin_6', 'seawater_bettina_36327_bin_7': 'seawater_bettina_36327_bin_7', 'CHO1_bin_4': 'CHO1_bin_4', 'APA_bin_10': 'APA_bin_10', 'RHO1_bin_10': 'RHO1_bin_10', 'IRC_PAM_SB0668_bin_7': 'IRC_PAM_SB0668_bin_7', 'COS36387_bin_11': 'COS36387_bin_11', 'IRC_PAM_SB0670_bin_19': 'IRC_PAM_SB0670_bin_19', 'RHO1_bin_29': 'RHO1_bin_29', 'COS36405_bin_1': 'COS36405_bin_1', 'COS1_bin_21': 'COS1_bin_21', 'IRC_PAM_SB0661_bin_55': 'IRC_PAM_SB0661_bin_55', 'COS4_bin_36': 'COS4_bin_36', 'STY2_bin_1': 'STY2_bin_1', 'IRC_PAM_SB0662_bin_39': 'IRC_PAM_SB0662_bin_39', 'RHO2_bin_55': 'RHO2_bin_55', 'APA_bin_97': 'APA_bin_97', 'CRAC_dobervaLami_GCF_000743705.1_ASM74370v1_genomic': 'GCF_000743705', 'COS4_bin_15': 'COS4_bin_15', 'IRC_PAM_SB0664_bin_2': 'IRC_PAM_SB0664_bin_2', 'IRC3_bin_2': 'IRC3_bin_2', 'APA_garciaTyalor_SAUL_aplysina_bin': 'aplysina_bin', 'seawater_42617_bin_6': 'seawater_42617_bin_6', 'COS36386_bin_14': 'COS36386_bin_14', 'IRC_PAM_SB0675_bin_16': 'IRC_PAM_SB0675_bin_16', 'IRC_PAM_SB0667_bin_13': 'IRC_PAM_SB0667_bin_13', 'RHO2_bin_6': 'RHO2_bin_6', 'COS36406_bin_7': 'COS36406_bin_7', 'IRC1_bin_26': 'IRC1_bin_26', 'AGET_podellAllen_GCA_003635305.1_ASM363530v1_genomic': 'GCA_003635305', 'IRC_PAM_SB0662_bin_10': 'IRC_PAM_SB0662_bin_10', 'RHO3_bin_24': 'RHO3_bin_24', 'IRC4_bin_16': 'IRC4_bin_16', 'APA_bin_22': 'APA_bin_22', 'CAR1_bin_7': 'CAR1_bin_7', 'RHO3_bin_80': 'RHO3_bin_80', 'seawater_42618_bin_6': 'seawater_42618_bin_6', 'COS36387_bin_3': 'COS36387_bin_3', 'CAR1_bin_15': 'CAR1_bin_15', 'RHO3_bin_25': 'RHO3_bin_25', 'CAR1_bin_14': 'CAR1_bin_14', 'STY1_bin_2': 'STY1_bin_2', 'APA_bin_3': 'APA_bin_3', 'RHO1_bin_24': 'RHO1_bin_24', 'IRC1_bin_20': 'IRC1_bin_20', 'IRC_PAM_SB0668_bin_5': 'IRC_PAM_SB0668_bin_5', 'seawater_22112_bin_2': 'seawater_22112_bin_2', 'IRC3_bin_16': 'IRC3_bin_16', 'RHO3_bin_1': 'RHO3_bin_1', 'RHO3_bin_23': 'RHO3_bin_23', 'CAR3_bin_14': 'CAR3_bin_14', 'CAR4_bin_16': 'CAR4_bin_16', 'CAR4_bin_1': 'CAR4_bin_1', 'COS36405_bin_8': 'COS36405_bin_8', 'TED_estevesThomas2016_Aquimarina_spAU58_IMGid2606217182': 'Aquimarina_spAU58', 'COS4_bin_23': 'COS4_bin_23', 'raw_name': 'renamed', 'APA_bin_19': 'APA_bin_19', 'RHO3_bin_49': 'RHO3_bin_49', 'CAR3_bin_11': 'CAR3_bin_11', 'COS1_bin_9': 'COS1_bin_9', 'RHO1_bin_68': 'RHO1_bin_68', 'IRC3_bin_21': 'IRC3_bin_21', 'IRC_PAM_SB0666_bin_11': 'IRC_PAM_SB0666_bin_11', 'RHO3_bin_74': 'RHO3_bin_74', 'PETF_garciaTaylor_SAUL_petrosia_ficiformis_bin': 'petrosia_ficiformis_bin', 'RHO1_bin_31': 'RHO1_bin_31', 'COS36405_bin_12': 'COS36405_bin_12', 'COS4_bin_46': 'COS4_bin_46', 'RHO3_bin_43': 'RHO3_bin_43', 'seawater_seasim_SB9153_S2_bin_4': 'seawater_seasim_SB9153_S2_bin_4', 'IRC_PAM_SB0664_bin_28': 'IRC_PAM_SB0664_bin_28', 'IRC_PAM_SB0661_bin_27': 'IRC_PAM_SB0661_bin_27', 'COS36404_bin_3': 'COS36404_bin_3'}

bin_to_source2_dict = {'seawater_bettina_36308_bin_5': 'seawater', 'RHO3_bin_71': 'Rhopaloides odorabile', 'CAR1_bin_15': 'Carteriospongia foliascens', 'IRC2_bin_6': 'Ircinia ramosa', 'RHO3_bin_39': 'Rhopaloides odorabile', 'APA_bin_81': 'Aplysina aerophoba', 'COS36386_bin_17': 'Coscinoderma matthewsi', 'IRC_PAM_SB0664_bin_15': 'Ircinia ramosa', 'RHO2_bin_15': 'Rhopaloides odorabile', 'CAR1_bin_16': 'Carteriospongia foliascens', 'COS2_bin_10': 'Coscinoderma matthewsi', 'IRC_PAM_SB0661_bin_37': 'Ircinia ramosa', 'seawater_seasim_SB9155_S4_bin_12': 'seawater', 'CAR1_bin_14': 'Carteriospongia foliascens', 'COS36404_bin_5': 'Coscinoderma matthewsi', 'LOPHE_tianQian_GCA_001543015.1_ASM154301v1_genomic': 'Lophophysema eversa', 'IRC_PAM_SB0661_bin_3': 'Ircinia ramosa', 'COS3_bin_13': 'Coscinoderma matthewsi', 'IRC_PAM_SB0662_bin_11': 'Ircinia ramosa', 'CAR4_bin_16': 'Carteriospongia foliascens', 'COS3_bin_12': 'Coscinoderma matthewsi', 'CAR1_bin_3': 'Carteriospongia foliascens', 'COS36386_bin_35': 'Coscinoderma matthewsi', 'SPOO_alexAntunes_GCF_900079515.1_Shewanella_sp.Alg231_23_genomic': 'Spongia officinalis', 'IRC_PAM_SB0668_bin_5': 'Ircinia ramosa', 'COS1_bin_19': 'Coscinoderma matthewsi', 'IRC_PAM_SB0661_bin_11': 'Ircinia ramosa', 'RHO3_bin_49': 'Rhopaloides odorabile', 'IRC4_bin_7': 'Ircinia ramosa', 'APA_bin_80': 'Aplysina aerophoba', 'RHO2_bin_40': 'Rhopaloides odorabile', 'IRC_PAM_SB0662_bin_51': 'Ircinia ramosa', 'STY3_bin_6': 'Stylissa flabelliformis', 'IRC_PAM_SB0667_bin_2': 'Ircinia ramosa', 'COS2_bin_16': 'Coscinoderma matthewsi', 'CAR1_bin_6': 'Carteriospongia foliascens', 'IRC3_bin_5': 'Ircinia ramosa', 'COS4_bin_5': 'Coscinoderma matthewsi', 'IRC_PAM_SB0675_bin_2': 'Ircinia ramosa', 'COS1_bin_17': 'Coscinoderma matthewsi', 'IRC_PAM_SB0668_bin_21': 'Ircinia ramosa', 'IRC_PAM_SB0668_bin_14': 'Ircinia ramosa', 'RHO1_bin_2': 'Rhopaloides odorabile', 'IRC_PAM_SB0661_bin_22': 'Ircinia ramosa', 'COS4_bin_14': 'Coscinoderma matthewsi', 'seawater_22112_bin_16': 'seawater', 'CAR2_bin_11': 'Carteriospongia foliascens', 'IRC_PAM_SB0678_bin_15': 'Ircinia ramosa', 'IRC_PAM_SB0662_bin_21': 'Ircinia ramosa', 'seawater_seasim_SB9152_S1_bin_1': 'seawater', 'COS36404_bin_1': 'Coscinoderma matthewsi', 'IRC_PAM_SB0665_bin_17': 'Ircinia ramosa', 'APA_bin_93': 'Aplysina aerophoba', 'IRC_PAM_SB0670_bin_39': 'Ircinia ramosa', 'COS1_bin_16': 'Coscinoderma matthewsi', 'APA_bin_4': 'Aplysina aerophoba', 'RHO3_bin_64': 'Rhopaloides odorabile', 'IRC3_bin_19': 'Ircinia ramosa', 'seawater_seasim_SB9156_S5_bin_3': 'seawater', 'COS36386_bin_3': 'Coscinoderma matthewsi', 'COS36386_bin_2': 'Coscinoderma matthewsi', 'seawater_bettina_36328_bin_3': 'seawater', 'COS3_bin_3': 'Coscinoderma matthewsi', 'seawater_22112_bin_8': 'seawater', 'RHO2_bin_9': 'Rhopaloides odorabile', 'IRC_PAM_SB0675_bin_29': 'Ircinia ramosa', 'RHO3_bin_40': 'Rhopaloides odorabile', 'APA_bin_33': 'Aplysina aerophoba', 'IRC_PAM_SB0675_bin_10': 'Ircinia ramosa', 'COS36388_bin_18': 'Coscinoderma matthewsi', 'STY4_bin_4': 'Stylissa flabelliformis', 'RHO3_bin_73': 'Rhopaloides odorabile', 'LOPHE_tianQian_GCA_001543005.1_ASM154300v1_genomic': 'Lophophysema eversa', 'IRC4_bin_2': 'Ircinia ramosa', 'IRC1_bin_21': 'Ircinia ramosa', 'RHO2_bin_67': 'Rhopaloides odorabile', 'IRC_PAM_SB0677_bin_1': 'Ircinia ramosa', 'IRC_PAM_SB0662_bin_20': 'Ircinia ramosa', 'PER4_bin_1': 'Pericharax heteroaphis', 'RHO2_bin_3': 'Rhopaloides odorabile', 'IRC3_bin_37': 'Ircinia ramosa', 'RHO3_bin_59': 'Rhopaloides odorabile', 'IRC_PAM_SB0677_bin_2': 'Ircinia ramosa', 'RHO2_bin_2': 'Rhopaloides odorabile', 'seawater_seasim_SB9154_S3_bin_1': 'seawater', 'PSE_podellAllen_GCA_003635255.1_ASM363525v1_genomic': 'Pseudoceratina sp.', 'IRC_PAM_SB0678_bin_9': 'Ircinia ramosa', 'COS36386_bin_15': 'Coscinoderma matthewsi', 'RHO1_bin_60': 'Rhopaloides odorabile', 'IRC_PAM_SB0661_bin_24': 'Ircinia ramosa', 'seawater_42618_bin_6': 'seawater', 'IRC_PAM_SB0662_bin_26': 'Ircinia ramosa', 'APA_bin_14': 'Aplysina aerophoba', 'COS36405_bin_10': 'Coscinoderma matthewsi', 'COS36387_bin_22': 'Coscinoderma matthewsi', 'COS1_bin_6': 'Coscinoderma matthewsi', 'COS36387_bin_11': 'Coscinoderma matthewsi', 'seawater_seasim_SB9153_S2_bin_2': 'seawater', 'APA_bin_97': 'Aplysina aerophoba', 'IRC_PAM_SB0675_bin_16': 'Ircinia ramosa', 'IRC3_bin_15': 'Ircinia ramosa', 'COS36386_bin_28': 'Coscinoderma matthewsi', 'COS36387_bin_20': 'Coscinoderma matthewsi', 'COS4_bin_12': 'Coscinoderma matthewsi', 'COS36387_bin_2': 'Coscinoderma matthewsi', 'COS3_bin_14': 'Coscinoderma matthewsi', 'APA_bin_43': 'Aplysina aerophoba', 'COS36387_bin_3': 'Coscinoderma matthewsi', 'seawater_bettina_36328_bin_2': 'seawater', 'seawater_42618_bin_25': 'seawater', 'SPOO_karimiCosta2019_GCF_900143545.1_Loktanella_sp._Alg231_35_genomic': 'Spongia officinalis', 'IRC1_bin_9': 'Ircinia ramosa', 'IRC4_bin_25': 'Ircinia ramosa', 'APA_bin_3': 'Aplysina aerophoba', 'COS36406_bin_7': 'Coscinoderma matthewsi', 'AGET_podellAllen_GCA_003635305.1_ASM363530v1_genomic': 'Agelas tubulata', 'COS3_bin_15': 'Coscinoderma matthewsi', 'COS36388_bin_5': 'Coscinoderma matthewsi', 'CAR4_bin_18': 'Carteriospongia foliascens', 'seawater_22112_bin_28': 'seawater', 'RHO1_bin_11': 'Rhopaloides odorabile', 'COS4_bin_2': 'Coscinoderma matthewsi', 'IRC_PAM_SB0661_bin_2': 'Ircinia ramosa', 'APA_burgsdorfSteindler_GCA_001007635.1_ASM100763v1_genomic': 'Aplysina aerophoba', 'COS36386_bin_37': 'Coscinoderma matthewsi', 'RHO1_bin_30': 'Rhopaloides odorabile', 'SPOO_karimiCosta2019_GCF_900143565.1_Pseudovibrio_sp._Alg231_02_genomic': 'Spongia officinalis', 'CAR1_bin_5': 'Carteriospongia foliascens', 'SPOO_karimiCosta2019_GCF_900143525.1_Ruegeria_sp._Alg231_54_genomic': 'Spongia officinalis', 'IRC4_bin_10': 'Ircinia ramosa', 'seawater_22112_bin_7': 'seawater', 'COS36386_bin_31': 'Coscinoderma matthewsi', 'RHO1_bin_32': 'Rhopaloides odorabile', 'COS2_bin_6': 'Coscinoderma matthewsi', 'seawater_22112_bin_14': 'seawater', 'seawater_seasim_SB9157_S6_bin_13': 'seawater', 'AMPF_kennedyDobson_GCF_900109375.1_IMGID2622736580_genomic': 'Amphilectus fucorum', 'IRC_PAM_SB0664_bin_2': 'Ircinia ramosa', 'IRC2_bin_12': 'Ircinia ramosa', 'RHO2_bin_41': 'Rhopaloides odorabile', 'IRC_PAM_SB0664_bin_6': 'Ircinia ramosa', 'IRC3_bin_10': 'Ircinia ramosa', 'COS4_bin_1': 'Coscinoderma matthewsi', 'THES_lavyIlan_GCA_002631715.1_ASM263171v1_genomic': 'Theonella Swinhonei', 'CAR3_bin_11': 'Carteriospongia foliascens', 'PETF_garciaTaylor_SAUL_petrosia_ficiformis_bin': 'Petrosia ficiformis', 'IRC_PAM_SB0668_bin_6': 'Ircinia ramosa', 'IRC_PAM_SB0665_bin_24': 'Ircinia ramosa', 'RHO2_bin_31': 'Rhopaloides odorabile', 'COS1_bin_10': 'Coscinoderma matthewsi', 'RHO2_bin_28': 'Rhopaloides odorabile', 'RHO3_bin_2': 'Rhopaloides odorabile', 'IRC_PAM_SB0661_bin_55': 'Ircinia ramosa', 'CLI4_bin_2': 'Cliona orientalis', 'COS4_bin_18': 'Coscinoderma matthewsi', 'COS4_bin_43': 'Coscinoderma matthewsi', 'RHO3_bin_37': 'Rhopaloides odorabile', 'IRC_PAM_SB0661_bin_1': 'Ircinia ramosa', 'RHO2_bin_23': 'Rhopaloides odorabile', 'APA_bin_39': 'Aplysina aerophoba', 'COS1_bin_7': 'Coscinoderma matthewsi', 'RHO1_bin_82': 'Rhopaloides odorabile', 'IRC_PAM_SB0661_bin_34': 'Ircinia ramosa', 'APA_bin_19': 'Aplysina aerophoba', 'RHO3_bin_84': 'Rhopaloides odorabile', 'COS36404_bin_2': 'Coscinoderma matthewsi', 'COS36387_bin_19': 'Coscinoderma matthewsi', 'IRC_PAM_SB0661_bin_41': 'Ircinia ramosa', 'IRC3_bin_3': 'Ircinia ramosa', 'STY4_bin_9': 'Stylissa flabelliformis', 'CAR4_bin_5': 'Carteriospongia foliascens', 'CAR2_bin_16': 'Carteriospongia foliascens', 'COS4_bin_49': 'Coscinoderma matthewsi', 'RHO2_bin_57': 'Rhopaloides odorabile', 'IRC_PAM_SB0678_bin_2': 'Ircinia ramosa', 'COS36388_bin_1': 'Coscinoderma matthewsi', 'IRC3_bin_16': 'Ircinia ramosa', 'seawater_bettina_36326_bin_6': 'seawater', 'IRC_PAM_SB0665_bin_19': 'Ircinia ramosa', 'IRC4_bin_33': 'Ircinia ramosa', 'RHO1_bin_18': 'Rhopaloides odorabile', 'IRC_PAM_SB0661_bin_16': 'Ircinia ramosa', 'RHO1_bin_66': 'Rhopaloides odorabile', 'APA_bin_61': 'Aplysina aerophoba', 'IRC_PAM_SB0665_bin_25': 'Ircinia ramosa', 'CAR1_bin_9': 'Carteriospongia foliascens', 'IRC1_bin_30': 'Ircinia ramosa', 'CAR2_bin_1': 'Carteriospongia foliascens', 'IRC_PAM_SB0661_bin_43': 'Ircinia ramosa', 'IRC1_bin_26': 'Ircinia ramosa', 'IRC_PAM_SB0670_bin_20': 'Ircinia ramosa', 'IRC2_bin_10': 'Ircinia ramosa', 'COS4_bin_44': 'Coscinoderma matthewsi', 'APA_bin_69': 'Aplysina aerophoba', 'COS36405_bin_12': 'Coscinoderma matthewsi', 'RHO3_bin_36': 'Rhopaloides odorabile', 'MYCL_zanHill_GCF_000158135.1_ASM15813v1_genomic': 'Mycale laxissima', 'IRC_PAM_SB0662_bin_53': 'Ircinia ramosa', 'IRC_PAM_SB0661_bin_8': 'Ircinia ramosa', 'STY4_bin_3': 'Stylissa flabelliformis', 'RHO2_bin_39': 'Rhopaloides odorabile', 'COS36404_bin_12': 'Coscinoderma matthewsi', 'COS4_bin_55': 'Coscinoderma matthewsi', 'COS36388_bin_3': 'Coscinoderma matthewsi', 'RHO3_bin_41': 'Rhopaloides odorabile', 'COS2_bin_8': 'Coscinoderma matthewsi', 'RHO3_bin_26': 'Rhopaloides odorabile', 'RHO3_bin_66': 'Rhopaloides odorabile', 'IRC4_bin_12': 'Ircinia ramosa', 'RHO1_bin_34': 'Rhopaloides odorabile', 'RHO1_bin_46': 'Rhopaloides odorabile', 'IRC3_bin_25': 'Ircinia ramosa', 'COS4_bin_4': 'Coscinoderma matthewsi', 'RHO3_bin_80': 'Rhopaloides odorabile', 'COS36404_bin_6': 'Coscinoderma matthewsi', 'IRC_PAM_SB0661_bin_14': 'Ircinia ramosa', 'CAR1_bin_1': 'Carteriospongia foliascens', 'IRC_PAM_SB0670_bin_22': 'Ircinia ramosa', 'CAR2_bin_20': 'Carteriospongia foliascens', 'IRC_PAM_SB0661_bin_19': 'Ircinia ramosa', 'RHO3_bin_14': 'Rhopaloides odorabile', 'RHO1_bin_14': 'Rhopaloides odorabile', 'SUB_tianQian_GCA_001541925.1_ASM154192v1_genomic': 'Suberites\xa0sp', 'SPOO_karimiCosta2019_GCF_900143635.1_Rhodobacteraceae_bacterium_Alg231_04_genomic': 'Spongia officinalis', 'IRC_PAM_SB0666_bin_10': 'Ircinia ramosa', 'RHO1_bin_37': 'Rhopaloides odorabile', 'APA_bin_70': 'Aplysina aerophoba', 'RHO2_bin_25': 'Rhopaloides odorabile', 'COS1_bin_12': 'Coscinoderma matthewsi', 'APA_bin_28': 'Aplysina aerophoba', 'RHO1_bin_68': 'Rhopaloides odorabile', 'RHO3_bin_13': 'Rhopaloides odorabile', 'IRC1_bin_15': 'Ircinia ramosa', 'APA_bin_82': 'Aplysina aerophoba', 'IRC_PAM_SB0665_bin_11': 'Ircinia ramosa', 'RHO3_bin_15': 'Rhopaloides odorabile', 'IRC_PAM_SB0662_bin_59': 'Ircinia ramosa', 'IRC_PAM_SB0661_bin_39': 'Ircinia ramosa', 'IRC4_bin_22': 'Ircinia ramosa', 'IRC_PAM_SB0667_bin_9': 'Ircinia ramosa', 'seawater_bettina_36327_bin_3': 'seawater', 'APA_bin_29': 'Aplysina aerophoba', 'IRC_PAM_SB0661_bin_30': 'Ircinia ramosa', 'COS36386_bin_18': 'Coscinoderma matthewsi', 'CAR2_bin_4': 'Carteriospongia foliascens', 'COS3_bin_2': 'Coscinoderma matthewsi', 'COS36406_bin_9': 'Coscinoderma matthewsi', 'SPOO_karimiCosta2019_GCF_900149685.1_Erythrobacter_sp._Alg231_14_genomic': 'Spongia officinalis', 'COS36405_bin_20': 'Coscinoderma matthewsi', 'RHO2_bin_60': 'Rhopaloides odorabile', 'IRC3_bin_18': 'Ircinia ramosa', 'CAR2_bin_22': 'Carteriospongia foliascens', 'IRC_PAM_SB0662_bin_10': 'Ircinia ramosa', 'IRC_PAM_SB0670_bin_18': 'Ircinia ramosa', 'COS3_bin_6': 'Coscinoderma matthewsi', 'CAR4_bin_3': 'Carteriospongia foliascens', 'COS36387_bin_18': 'Coscinoderma matthewsi', 'IRC_PAM_SB0661_bin_6': 'Ircinia ramosa', 'CAR3_bin_9': 'Carteriospongia foliascens', 'APA_bin_53': 'Aplysina aerophoba', 'seawater_bettina_36310_bin_4': 'seawater', 'OPHP_alexAntunes_GCF_004168585.1_ASM416858v1_genomic': 'Ophlitaspongia papilla', 'COS36406_bin_6': 'Coscinoderma matthewsi', 'IRC_PAM_SB0666_bin_13': 'Ircinia ramosa', 'COS4_bin_23': 'Coscinoderma matthewsi', 'RHO2_bin_37': 'Rhopaloides odorabile', 'COS36404_bin_10': 'Coscinoderma matthewsi', 'CAR3_bin_4': 'Carteriospongia foliascens', 'CAR4_bin_13': 'Carteriospongia foliascens', 'COS4_bin_13': 'Coscinoderma matthewsi', 'RHO3_bin_28': 'Rhopaloides odorabile', 'seawater_22112_bin_25': 'seawater', 'CAR2_bin_13': 'Carteriospongia foliascens', 'IRC_PAM_SB0662_bin_15': 'Ircinia ramosa', 'IRC_PAM_SB0670_bin_1': 'Ircinia ramosa', 'LOPHE_tianQian_GCA_001542995.1_ASM154299v1_genomic': 'Lophophysema eversa', 'APA_bin_23': 'Aplysina aerophoba', 'RHO1_bin_28': 'Rhopaloides odorabile', 'IRC1_bin_25': 'Ircinia ramosa', 'CAR2_bin_8': 'Carteriospongia foliascens', 'IRC3_bin_35': 'Ircinia ramosa', 'IRC_PAM_SB0662_bin_22': 'Ircinia ramosa', 'COS4_bin_40': 'Coscinoderma matthewsi', 'IRC_PAM_SB0670_bin_19': 'Ircinia ramosa', 'COS4_bin_16': 'Coscinoderma matthewsi', 'RHO3_bin_70': 'Rhopaloides odorabile', 'IRC1_bin_6': 'Ircinia ramosa', 'RHO2_bin_24': 'Rhopaloides odorabile', 'IRC_PAM_SB0661_bin_44': 'Ircinia ramosa', 'IRC_PAM_SB0664_bin_7': 'Ircinia ramosa', 'IRC_PAM_SB0665_bin_27': 'Ircinia ramosa', 'seawater_seasim_SB9153_S2_bin_10': 'seawater', 'COS4_bin_36': 'Coscinoderma matthewsi', 'CAR3_bin_17': 'Carteriospongia foliascens', 'IRC4_bin_11': 'Ircinia ramosa', 'IRC_PAM_SB0668_bin_11': 'Ircinia ramosa', 'CAR2_bin_10': 'Carteriospongia foliascens', 'RHO2_bin_49': 'Rhopaloides odorabile', 'IRC4_bin_38': 'Ircinia ramosa', 'RHO2_bin_43': 'Rhopaloides odorabile', 'CYMC_estevesThomas2016_Pseudovibrio_spAU243_2606217185': 'Cymbastela concentrica', 'COS2_bin_2': 'Coscinoderma matthewsi', 'IRC_PAM_SB0675_bin_14': 'Ircinia ramosa', 'IRC_PAM_SB0664_bin_22': 'Ircinia ramosa', 'RHO3_bin_4': 'Rhopaloides odorabile', 'RHO3_bin_24': 'Rhopaloides odorabile', 'IRC_PAM_SB0661_bin_26': 'Ircinia ramosa', 'seawater_42618_bin_2': 'seawater', 'RHO3_bin_79': 'Rhopaloides odorabile', 'COS4_bin_28': 'Coscinoderma matthewsi', 'IRC4_bin_13': 'Ircinia ramosa', 'IRC3_bin_20': 'Ircinia ramosa', 'IRC_PAM_SB0662_bin_12': 'Ircinia ramosa', 'CAR2_bin_3': 'Carteriospongia foliascens', 'IRC_PAM_SB0677_bin_15': 'Ircinia ramosa', 'IRC_PAM_SB0675_bin_12': 'Ircinia ramosa', 'RHO1_bin_42': 'Rhopaloides odorabile', 'seawater_42616_bin_2': 'seawater', 'IRC_PAM_SB0677_bin_7': 'Ircinia ramosa', 'COS2_bin_5': 'Coscinoderma matthewsi', 'IRC_PAM_SB0662_bin_49': 'Ircinia ramosa', 'COS4_bin_29': 'Coscinoderma matthewsi', 'COS36386_bin_14': 'Coscinoderma matthewsi', 'IRC4_bin_39': 'Ircinia ramosa', 'COS1_bin_13': 'Coscinoderma matthewsi', 'CAR2_bin_15': 'Carteriospongia foliascens', 'COS36406_bin_18': 'Coscinoderma matthewsi', 'APA_bin_73': 'Aplysina aerophoba', 'COS4_bin_26': 'Coscinoderma matthewsi', 'IRC1_bin_12': 'Ircinia ramosa', 'CAR3_bin_1': 'Carteriospongia foliascens', 'IRC1_bin_1': 'Ircinia ramosa', 'IRC_PAM_SB0668_bin_13': 'Ircinia ramosa', 'IRC_PAM_SB0666_bin_22': 'Ircinia ramosa', 'IRC3_bin_21': 'Ircinia ramosa', 'COS3_bin_5': 'Coscinoderma matthewsi', 'APA_bin_89': 'Aplysina aerophoba', 'RHO1_bin_29': 'Rhopaloides odorabile', 'RHO3_bin_23': 'Rhopaloides odorabile', 'IRC_PAM_SB0668_bin_27': 'Ircinia ramosa', 'COS36405_bin_2': 'Coscinoderma matthewsi', 'IRC_PAM_SB0665_bin_8': 'Ircinia ramosa', 'seawater_bettina_36309_bin_17': 'seawater', 'IRC_PAM_SB0677_bin_8': 'Ircinia ramosa', 'COS1_bin_21': 'Coscinoderma matthewsi', 'seawater_bettina_36310_bin_3': 'seawater', 'IRC_PAM_SB0672_bin_11': 'Ircinia ramosa', 'APA_bin_15': 'Aplysina aerophoba', 'RHO3_bin_33': 'Rhopaloides odorabile', 'IRC_PAM_SB0664_bin_11': 'Ircinia ramosa', 'IRC_PAM_SB0666_bin_21': 'Ircinia ramosa', 'IRC4_bin_8': 'Ircinia ramosa', 'COS4_bin_51': 'Coscinoderma matthewsi', 'seawater_seasim_SB9152_S1_bin_2': 'seawater', 'IRC_PAM_SB0664_bin_24': 'Ircinia ramosa', 'seawater_bettina_36327_bin_6': 'seawater', 'CAR3_bin_3': 'Carteriospongia foliascens', 'seawater_bettina_36309_bin_3': 'seawater', 'COS4_bin_32': 'Coscinoderma matthewsi', 'IRC_PAM_SB0670_bin_12': 'Ircinia ramosa', 'RHO1_bin_62': 'Rhopaloides odorabile', 'RHO2_bin_53': 'Rhopaloides odorabile', 'IRC4_bin_23': 'Ircinia ramosa', 'COS36404_bin_8': 'Coscinoderma matthewsi', 'IRC1_bin_3': 'Ircinia ramosa', 'IRC_PAM_SB0664_bin_14': 'Ircinia ramosa', 'IRC1_bin_19': 'Ircinia ramosa', 'seawater_seasim_SB9152_S1_bin_12': 'seawater', 'COS3_bin_9': 'Coscinoderma matthewsi', 'seawater_22112_bin_1': 'seawater', 'IRC2_bin_2': 'Ircinia ramosa', 'RHO2_bin_55': 'Rhopaloides odorabile', 'seawater_seasim_SB9154_S3_bin_3': 'seawater', 'RHO3_bin_11': 'Rhopaloides odorabile', 'COS36386_bin_11': 'Coscinoderma matthewsi', 'seawater_seasim_SB9157_S6_bin_2': 'seawater', 'seawater_seasim_SB9156_S5_bin_8': 'seawater', 'CAR3_bin_18': 'Carteriospongia foliascens', 'APA_bin_18': 'Aplysina aerophoba', 'IRC2_bin_4': 'Ircinia ramosa', 'RHO1_bin_72': 'Rhopaloides odorabile', 'CAR3_bin_5': 'Carteriospongia foliascens', 'seawater_seasim_SB9152_S1_bin_4': 'seawater', 'RHO3_bin_57': 'Rhopaloides odorabile', 'CLI1_bin_4': 'Cliona orientalis', 'COS1_bin_9': 'Coscinoderma matthewsi', 'CAR2_bin_14': 'Carteriospongia foliascens', 'APA_bin_68': 'Aplysina aerophoba', 'COS36388_bin_6': 'Coscinoderma matthewsi', 'COS36388_bin_16': 'Coscinoderma matthewsi', 'IRC1_bin_22': 'Ircinia ramosa', 'seawater_bettina_36308_bin_3': 'seawater', 'RHO2_bin_54': 'Rhopaloides odorabile', 'IRC_PAM_SB0665_bin_12': 'Ircinia ramosa', 'APA_bin_40': 'Aplysina aerophoba', 'seawater_bettina_36326_bin_1': 'seawater', 'COS4_bin_27': 'Coscinoderma matthewsi', 'THES_wilsonPiel_GCA_000522425.1_v3_genomic': 'Theonella Swinhonei', 'IRC4_bin_9': 'Ircinia ramosa', 'COS36387_bin_16': 'Coscinoderma matthewsi', 'IRC_PAM_SB0661_bin_21': 'Ircinia ramosa', 'RHO2_bin_36': 'Rhopaloides odorabile', 'SUB_tianQian_GCA_002007405.1_ASM200740v1_genomic': 'Suberites\xa0sp', 'RHO3_bin_29': 'Rhopaloides odorabile', 'CAR1_bin_11': 'Carteriospongia foliascens', 'RHO3_bin_58': 'Rhopaloides odorabile', 'COS36386_bin_10': 'Coscinoderma matthewsi', 'COS3_bin_1': 'Coscinoderma matthewsi', 'RHO2_bin_64': 'Rhopaloides odorabile', 'COS3_bin_4': 'Coscinoderma matthewsi', 'COS36388_bin_17': 'Coscinoderma matthewsi', 'CAR2_bin_7': 'Carteriospongia foliascens', 'APA_bin_63': 'Aplysina aerophoba', 'RHO1_bin_22': 'Rhopaloides odorabile', 'COS1_bin_1': 'Coscinoderma matthewsi', 'COS3_bin_11': 'Coscinoderma matthewsi', 'APA_bin_7': 'Aplysina aerophoba', 'seawater_42615_bin_16': 'seawater', 'IRC_PAM_SB0675_bin_19': 'Ircinia ramosa', 'IRC4_bin_37': 'Ircinia ramosa', 'RHO3_bin_6': 'Rhopaloides odorabile', 'seawater_22112_bin_6': 'seawater', 'IRC4_bin_4': 'Ircinia ramosa', 'COS36387_bin_14': 'Coscinoderma matthewsi', 'IRC1_bin_16': 'Ircinia ramosa', 'IRC_PAM_SB0661_bin_27': 'Ircinia ramosa', 'seawater_seasim_SB9160_S9_bin_9': 'seawater', 'IRC_PAM_SB0662_bin_36': 'Ircinia ramosa', 'STY1_bin_1': 'Stylissa flabelliformis', 'IRC_PAM_SB0668_bin_20': 'Ircinia ramosa', 'RHO2_bin_11': 'Rhopaloides odorabile', 'IRC4_bin_3': 'Ircinia ramosa', 'MELS_podellAllen_GCA_GCA_003635315.1_ASM363531v1_genomic': 'Melophlus sarasinorum', 'COS36405_bin_11': 'Coscinoderma matthewsi', 'IRC_PAM_SB0664_bin_9': 'Ircinia ramosa', 'COS2_bin_4': 'Coscinoderma matthewsi', 'COS36405_bin_14': 'Coscinoderma matthewsi', 'IRC_PAM_SB0677_bin_14': 'Ircinia ramosa', 'IRC_PAM_SB0676_bin_21': 'Ircinia ramosa', 'CAR3_bin_16': 'Carteriospongia foliascens', 'RHO2_bin_12': 'Rhopaloides odorabile', 'TED_braunBugni_GCF_002573675.1_ASM257367v1_genomic': 'Tedania\xa0sp.', 'seawater_seasim_SB9155_S4_bin_5': 'seawater', 'seawater_seasim_SB9156_S5_bin_6': 'seawater', 'IRC3_bin_31': 'Ircinia ramosa', 'IRC_PAM_SB0665_bin_1': 'Ircinia ramosa', 'APA_bin_27': 'Aplysina aerophoba', 'COS1_bin_15': 'Coscinoderma matthewsi', 'APA_bin_32': 'Aplysina aerophoba', 'APA_bin_86': 'Aplysina aerophoba', 'seawater_bettina_36328_bin_30': 'seawater', 'STY2_bin_1': 'Stylissa flabelliformis', 'STY2_bin_7': 'Stylissa flabelliformis', 'RHO2_bin_16': 'Rhopaloides odorabile', 'IRC2_bin_5': 'Ircinia ramosa', 'RHO1_bin_52': 'Rhopaloides odorabile', 'IRC4_bin_49': 'Ircinia ramosa', 'CAR3_bin_10': 'Carteriospongia foliascens', 'CAR3_bin_2': 'Carteriospongia foliascens', 'COS4_bin_25': 'Coscinoderma matthewsi', 'COS36405_bin_3': 'Coscinoderma matthewsi', 'RHO2_bin_62': 'Rhopaloides odorabile', 'RHO2_bin_7': 'Rhopaloides odorabile', 'IRC_PAM_SB0677_bin_16': 'Ircinia ramosa', 'RHO3_bin_22': 'Rhopaloides odorabile', 'seawater_seasim_SB9152_S1_bin_8': 'seawater', 'SPOO_karimiCosta_FZLS01': 'Spongia officinalis', 'RHO2_bin_22': 'Rhopaloides odorabile', 'RHO1_bin_21': 'Rhopaloides odorabile', 'IRC1_bin_2': 'Ircinia ramosa', 'APA_bin_22': 'Aplysina aerophoba', 'IRC2_bin_1': 'Ircinia ramosa', 'RHO3_bin_32': 'Rhopaloides odorabile', 'RHO1_bin_33': 'Rhopaloides odorabile', 'seawater_22112_bin_9': 'seawater', 'RHO3_bin_51': 'Rhopaloides odorabile', 'IRC_PAM_SB0666_bin_34': 'Ircinia ramosa', 'CAR1_bin_7': 'Carteriospongia foliascens', 'RHO2_bin_50': 'Rhopaloides odorabile', 'COS36387_bin_15': 'Coscinoderma matthewsi', 'COS3_bin_18': 'Coscinoderma matthewsi', 'RHO1_bin_31': 'Rhopaloides odorabile', 'IRC3_bin_13': 'Ircinia ramosa', 'RHO2_bin_38': 'Rhopaloides odorabile', 'COS36386_bin_4': 'Coscinoderma matthewsi', 'CAR3_bin_6': 'Carteriospongia foliascens', 'COS1_bin_3': 'Coscinoderma matthewsi', 'IRC_PAM_SB0665_bin_16': 'Ircinia ramosa', 'COS4_bin_34': 'Coscinoderma matthewsi', 'seawater_seasim_SB9153_S2_bin_4': 'seawater', 'IRC_PAM_SB0662_bin_35': 'Ircinia ramosa', 'COS1_bin_18': 'Coscinoderma matthewsi', 'RHO1_bin_25': 'Rhopaloides odorabile', 'APA_bin_62': 'Aplysina aerophoba', 'IRC_PAM_SB0677_bin_11': 'Ircinia ramosa', 'IRC_PAM_SB0665_bin_5': 'Ircinia ramosa', 'STY4_bin_7': 'Stylissa flabelliformis', 'COS4_bin_11': 'Coscinoderma matthewsi', 'APA_bin_48': 'Aplysina aerophoba', 'RHO1_bin_40': 'Rhopaloides odorabile', 'COS4_bin_22': 'Coscinoderma matthewsi', 'APA_bin_98': 'Aplysina aerophoba', 'COS36386_bin_6': 'Coscinoderma matthewsi', 'IRC_PAM_SB0661_bin_31': 'Ircinia ramosa', 'seawater_42617_bin_7': 'seawater', 'COS36404_bin_3': 'Coscinoderma matthewsi', 'APA_bin_59': 'Aplysina aerophoba', 'IRC4_bin_14': 'Ircinia ramosa', 'IRC4_bin_21': 'Ircinia ramosa', 'IRC1_bin_11': 'Ircinia ramosa', 'RHO3_bin_21': 'Rhopaloides odorabile', 'COS4_bin_42': 'Coscinoderma matthewsi', 'CAR4_bin_8': 'Carteriospongia foliascens', 'SPOO_karimiCosta_FZLQ01': 'Spongia officinalis', 'IRC_PAM_SB0662_bin_5': 'Ircinia ramosa', 'seawater_bettina_36327_bin_4': 'seawater', 'COS36386_bin_32': 'Coscinoderma matthewsi', 'IRC_PAM_SB0662_bin_58': 'Ircinia ramosa', 'IRC1_bin_18': 'Ircinia ramosa', 'RHO3_bin_74': 'Rhopaloides odorabile', 'COS4_bin_21': 'Coscinoderma matthewsi', 'seawater_22112_bin_12': 'seawater', 'IRC4_bin_40': 'Ircinia ramosa', 'IRC_PAM_SB0668_bin_1': 'Ircinia ramosa', 'IRC_PAM_SB0667_bin_1': 'Ircinia ramosa', 'seawater_22112_bin_2': 'seawater', 'IRC4_bin_42': 'Ircinia ramosa', 'APA_bin_94': 'Aplysina aerophoba', 'COS4_bin_10': 'Coscinoderma matthewsi', 'seawater_seasim_SB9152_S1_bin_18': 'seawater', 'RHO2_bin_59': 'Rhopaloides odorabile', 'STY1_bin_3': 'Stylissa flabelliformis', 'IRC_PAM_SB0662_bin_34': 'Ircinia ramosa', 'seawater_22112_bin_15': 'seawater', 'RHO3_bin_7': 'Rhopaloides odorabile', 'COS36405_bin_5': 'Coscinoderma matthewsi', 'COS4_bin_24': 'Coscinoderma matthewsi', 'IRC_PAM_SB0664_bin_16': 'Ircinia ramosa', 'COS4_bin_8': 'Coscinoderma matthewsi', 'CAR3_bin_14': 'Carteriospongia foliascens', 'seawater_seasim_SB9156_S5_bin_14': 'seawater', 'RHO1_bin_13': 'Rhopaloides odorabile', 'COS4_bin_3': 'Coscinoderma matthewsi', 'PSE_podellAllen_GCA_003635195.1_ASM363519v1_genomic': 'Pseudoceratina sp.', 'RHO2_bin_20': 'Rhopaloides odorabile', 'COS2_bin_23': 'Coscinoderma matthewsi', 'COS36386_bin_19': 'Coscinoderma matthewsi', 'COS4_bin_19': 'Coscinoderma matthewsi', 'RHO2_bin_35': 'Rhopaloides odorabile', 'APA_bin_87': 'Aplysina aerophoba', 'RHO1_bin_45': 'Rhopaloides odorabile', 'APA_bin_72': 'Aplysina aerophoba', 'COS36386_bin_8': 'Coscinoderma matthewsi', 'RHO1_bin_4': 'Rhopaloides odorabile', 'IRC4_bin_1': 'Ircinia ramosa', 'APA_bin_12': 'Aplysina aerophoba', 'RHO2_bin_8': 'Rhopaloides odorabile', 'SPOO_karimiCosta2019_GCF_900143555.1_Labrenzia_sp._Alg231_36_genomic': 'Spongia officinalis', 'seawater_42618_bin_9': 'seawater', 'IRC4_bin_20': 'Ircinia ramosa', 'RHO2_bin_27': 'Rhopaloides odorabile', 'CAR1_bin_18': 'Carteriospongia foliascens', 'IRC_PAM_SB0662_bin_23': 'Ircinia ramosa', 'IRC_PAM_SB0675_bin_3': 'Ircinia ramosa', 'IRC1_bin_4': 'Ircinia ramosa', 'IRC2_bin_13': 'Ircinia ramosa', 'RHO3_bin_27': 'Rhopaloides odorabile', 'COS1_bin_4': 'Coscinoderma matthewsi', 'RHO1_bin_12': 'Rhopaloides odorabile', 'RHO3_bin_72': 'Rhopaloides odorabile', 'IRC_PAM_SB0665_bin_2': 'Ircinia ramosa', 'COS36405_bin_8': 'Coscinoderma matthewsi', 'seawater_42617_bin_6': 'seawater', 'COS1_bin_11': 'Coscinoderma matthewsi', 'RHO3_bin_47': 'Rhopaloides odorabile', 'STY2_bin_2': 'Stylissa flabelliformis', 'IRC_PAM_SB0661_bin_29': 'Ircinia ramosa', 'APA_bin_1': 'Aplysina aerophoba', 'APA_bin_83': 'Aplysina aerophoba', 'APA_bin_102': 'Aplysina aerophoba', 'IRC1_bin_14': 'Ircinia ramosa', 'CRAC_dobervaLami_GCF_000743705.1_ASM74370v1_genomic': 'Crambe crambe', 'IRC_PAM_SB0664_bin_31': 'Ircinia ramosa', 'CAR2_bin_9': 'Carteriospongia foliascens', 'IRC_PAM_SB0664_bin_25': 'Ircinia ramosa', 'RHO1_bin_9': 'Rhopaloides odorabile', 'IRC2_bin_3': 'Ircinia ramosa', 'IRC_PAM_SB0666_bin_6': 'Ircinia ramosa', 'RHO2_bin_42': 'Rhopaloides odorabile', 'IRC_PAM_SB0664_bin_28': 'Ircinia ramosa', 'COS36405_bin_1': 'Coscinoderma matthewsi', 'RHO3_bin_1': 'Rhopaloides odorabile', 'seawater_seasim_SB9153_S2_bin_13': 'seawater', 'RHO3_bin_8': 'Rhopaloides odorabile', 'seawater_seasim_SB9154_S3_bin_4': 'seawater', 'RHO1_bin_39': 'Rhopaloides odorabile', 'IRC3_bin_7': 'Ircinia ramosa', 'IRC_PAM_SB0665_bin_9': 'Ircinia ramosa', 'RHO2_bin_33': 'Rhopaloides odorabile', 'RHO2_bin_17': 'Rhopaloides odorabile', 'IRC1_bin_38': 'Ircinia ramosa', 'COS1_bin_2': 'Coscinoderma matthewsi', 'IRC_PAM_SB0678_bin_6': 'Ircinia ramosa', 'IRC_PAM_SB0672_bin_21': 'Ircinia ramosa', 'COS36387_bin_10': 'Coscinoderma matthewsi', 'RHO1_bin_6': 'Rhopaloides odorabile', 'unknown_alexAntunes_GCF_003676335.1_ASM367633v1_genomic': 'unknown sponge', 'CAR4_bin_4': 'Carteriospongia foliascens', 'APA_bin_34': 'Aplysina aerophoba', 'COS36386_bin_12': 'Coscinoderma matthewsi', 'IRC4_bin_35': 'Ircinia ramosa', 'RHO2_bin_18': 'Rhopaloides odorabile', 'COS4_bin_46': 'Coscinoderma matthewsi', 'AREB_froesThompson_GCF_001941685.1_ASM194168v1_genomic': 'Arenosclera brasiliensis', 'CLI4_bin_1': 'Cliona orientalis', 'COS4_bin_9': 'Coscinoderma matthewsi', 'RHO3_bin_67': 'Rhopaloides odorabile', 'STY1_bin_2': 'Stylissa flabelliformis', 'IRC3_bin_2': 'Ircinia ramosa', 'seawater_42618_bin_29': 'seawater', 'RHO1_bin_27': 'Rhopaloides odorabile', 'IRC_PAM_SB0661_bin_15': 'Ircinia ramosa', 'COS36388_bin_4': 'Coscinoderma matthewsi', 'IRC1_bin_37': 'Ircinia ramosa', 'RHO1_bin_85': 'Rhopaloides odorabile', 'IRC_PAM_SB0675_bin_18': 'Ircinia ramosa', 'CAR2_bin_6': 'Carteriospongia foliascens', 'IRC_PAM_SB0662_bin_1': 'Ircinia ramosa', 'IRC_PAM_SB0662_bin_27': 'Ircinia ramosa', 'HALC_tianQian2014_GCA_000583135.1_Thioalkalivibrio_spongium_HK1_genomic': 'Haliclona cymaeformis', 'APA_bin_90': 'Aplysina aerophoba', 'RHO3_bin_9': 'Rhopaloides odorabile', 'RHO3_bin_34': 'Rhopaloides odorabile', 'RHO3_bin_31': 'Rhopaloides odorabile', 'IRC_PAM_SB0678_bin_7': 'Ircinia ramosa', 'RHO2_bin_5': 'Rhopaloides odorabile', 'IRC_PAM_SB0673_bin_10': 'Ircinia ramosa', 'IRC3_bin_6': 'Ircinia ramosa', 'IRC_PAM_SB0666_bin_1': 'Ircinia ramosa', 'seawater_22112_bin_4': 'seawater', 'IRC4_bin_6': 'Ircinia ramosa', 'STY4_bin_2': 'Stylissa flabelliformis', 'IRC_PAM_SB0661_bin_4': 'Ircinia ramosa', 'RHO1_bin_64': 'Rhopaloides odorabile', 'IRC4_bin_30': 'Ircinia ramosa', 'APA_bin_52': 'Aplysina aerophoba', 'RHO2_bin_52': 'Rhopaloides odorabile', 'seawater_bettina_36327_bin_2': 'seawater', 'CAR1_bin_2': 'Carteriospongia foliascens', 'IRC_PAM_SB0668_bin_12': 'Ircinia ramosa', 'CAR2_bin_2': 'Carteriospongia foliascens', 'IRC_PAM_SB0673_bin_16': 'Ircinia ramosa', 'CAR2_bin_19': 'Carteriospongia foliascens', 'seawater_22112_bin_10': 'seawater', 'IRC_PAM_SB0662_bin_28': 'Ircinia ramosa', 'TED_estevesThomas2016_Aquimarina_spAU58_IMGid2606217182': 'Tedania\xa0sp.', 'IRC1_bin_27': 'Ircinia ramosa', 'IRC1_bin_13': 'Ircinia ramosa', 'IRC_PAM_SB0675_bin_22': 'Ircinia ramosa', 'RHO1_bin_26': 'Rhopaloides odorabile', 'IRC3_bin_26': 'Ircinia ramosa', 'RHO3_bin_48': 'Rhopaloides odorabile', 'seawater_bettina_36309_bin_6': 'seawater', 'IRC_PAM_SB0675_bin_5': 'Ircinia ramosa', 'STY1_bin_6': 'Stylissa flabelliformis', 'RHO2_bin_19': 'Rhopaloides odorabile', 'IRC_PAM_SB0668_bin_7': 'Ircinia ramosa', 'TED_estevesThomas2016_Aquimarina_spAU119_2606217184': 'Tedania\xa0sp.', 'IRC3_bin_8': 'Ircinia ramosa', 'IRC_PAM_SB0664_bin_33': 'Ircinia ramosa', 'COS36404_bin_19': 'Coscinoderma matthewsi', 'seawater_22112_bin_29': 'seawater', 'RHO1_bin_44': 'Rhopaloides odorabile', 'COS36388_bin_15': 'Coscinoderma matthewsi', 'COS4_bin_47': 'Coscinoderma matthewsi', 'CAR4_bin_6': 'Carteriospongia foliascens', 'IRC4_bin_24': 'Ircinia ramosa', 'RHO1_bin_69': 'Rhopaloides odorabile', 'COS2_bin_17': 'Coscinoderma matthewsi', 'COS3_bin_16': 'Coscinoderma matthewsi', 'IRC1_bin_34': 'Ircinia ramosa', 'IRC3_bin_14': 'Ircinia ramosa', 'IRC1_bin_35': 'Ircinia ramosa', 'RHO3_bin_69': 'Rhopaloides odorabile', 'IRC4_bin_41': 'Ircinia ramosa', 'IRC4_bin_15': 'Ircinia ramosa', 'APA_bin_45': 'Aplysina aerophoba', 'RHO1_bin_56': 'Rhopaloides odorabile', 'seawater_bettina_36326_bin_26': 'seawater', 'TED_estevesThomas2016_Aquimarina_spAU474_2606217188': 'Tedania\xa0sp.', 'IRC3_bin_11': 'Ircinia ramosa', 'APA_bin_85': 'Aplysina aerophoba', 'APA_bin_41': 'Aplysina aerophoba', 'CAR1_bin_10': 'Carteriospongia foliascens', 'seawater_seasim_SB9152_S1_bin_19': 'seawater', 'MYCL_bondarevVogt_GCF_000156235.1_ASM15623v1_genomic': 'Mycale laxissima', 'RHO2_bin_30': 'Rhopaloides odorabile', 'HALO_yoonOh_GCF_900141785.1_IMGID2622736502_genomic': 'Halichondria oshoro', 'RHO3_bin_38': 'Rhopaloides odorabile', 'COS3_bin_17': 'Coscinoderma matthewsi', 'RHO2_bin_6': 'Rhopaloides odorabile', 'COS2_bin_3': 'Coscinoderma matthewsi', 'RHO3_bin_5': 'Rhopaloides odorabile', 'IRC_PAM_SB0662_bin_9': 'Ircinia ramosa', 'IRC_PAM_SB0664_bin_12': 'Ircinia ramosa', 'IRC_PAM_SB0675_bin_1': 'Ircinia ramosa', 'IRC_PAM_SB0662_bin_30': 'Ircinia ramosa', 'COS3_bin_8': 'Coscinoderma matthewsi', 'RHO2_bin_65': 'Rhopaloides odorabile', 'RHO3_bin_25': 'Rhopaloides odorabile', 'RHO1_bin_15': 'Rhopaloides odorabile', 'RHO3_bin_17': 'Rhopaloides odorabile', 'IRC_PAM_SB0664_bin_5': 'Ircinia ramosa', 'CLI3_bin_1': 'Cliona orientalis', 'IRC_PAM_SB0664_bin_10': 'Ircinia ramosa', 'IRC1_bin_31': 'Ircinia ramosa', 'IRC1_bin_23': 'Ircinia ramosa', 'CAR4_bin_1': 'Carteriospongia foliascens', 'seawater_bettina_36309_bin_5': 'seawater', 'COS36388_bin_11': 'Coscinoderma matthewsi', 'seawater_bettina_36328_bin_5': 'seawater', 'STY1_bin_7': 'Stylissa flabelliformis', 'COS36406_bin_13': 'Coscinoderma matthewsi', 'CAR3_bin_13': 'Carteriospongia foliascens', 'RHO3_bin_68': 'Rhopaloides odorabile', 'IRC_PAM_SB0661_bin_32': 'Ircinia ramosa', 'CAR1_bin_4': 'Carteriospongia foliascens', 'IRC_PAM_SB0667_bin_13': 'Ircinia ramosa', 'RHO2_bin_1': 'Rhopaloides odorabile', 'seawater_42615_bin_2': 'seawater', 'RHO1_bin_8': 'Rhopaloides odorabile', 'IRC1_bin_7': 'Ircinia ramosa', 'IRC_PAM_SB0661_bin_33': 'Ircinia ramosa', 'IRC3_bin_17': 'Ircinia ramosa', 'IRC_PAM_SB0661_bin_40': 'Ircinia ramosa', 'CAR4_bin_10': 'Carteriospongia foliascens', 'COS4_bin_6': 'Coscinoderma matthewsi', 'seawater_seasim_SB9156_S5_bin_20': 'seawater', 'STY1_bin_8': 'Stylissa flabelliformis', 'RHO2_bin_56': 'Rhopaloides odorabile', 'COS36387_bin_4': 'Coscinoderma matthewsi', 'CHO1_bin_2': 'Chondrilla australiensis', 'SPOO_karimiCosta2019_GCF_900149695.1_Anderseniella_sp._Alg231_50_genomic': 'Spongia officinalis', 'THES_burgsdorfSteindler_GCA_001007665.1_ASM100766v1_genomic': 'Theonella Swinhonei', 'IRC_PAM_SB0664_bin_4': 'Ircinia ramosa', 'IRC_PAM_SB0664_bin_27': 'Ircinia ramosa', 'APA_bin_38': 'Aplysina aerophoba', 'IRC_PAM_SB0661_bin_45': 'Ircinia ramosa', 'RHO3_bin_18': 'Rhopaloides odorabile', 'seawater_seasim_SB9152_S1_bin_3': 'seawater', 'IRC2_bin_8': 'Ircinia ramosa', 'IRC4_bin_45': 'Ircinia ramosa', 'seawater_seasim_SB9160_S9_bin_11': 'seawater', 'IRC_PAM_SB0675_bin_20': 'Ircinia ramosa', 'APA_bin_96': 'Aplysina aerophoba', 'STY4_bin_8': 'Stylissa flabelliformis', 'RHO1_bin_3': 'Rhopaloides odorabile', 'IRC_PAM_SB0667_bin_14': 'Ircinia ramosa', 'IRC2_bin_7': 'Ircinia ramosa', 'RHO1_bin_65': 'Rhopaloides odorabile', 'CAR4_bin_9': 'Carteriospongia foliascens', 'APA_garciaTyalor_SAUL_aplysina_bin': 'Aplysina aerophoba', 'seawater_42617_bin_1': 'seawater', 'CLI1_bin_1': 'Cliona orientalis', 'COS4_bin_20': 'Coscinoderma matthewsi', 'IRC_PAM_SB0676_bin_26': 'Ircinia ramosa', 'COS36406_bin_19': 'Coscinoderma matthewsi', 'COS4_bin_17': 'Coscinoderma matthewsi', 'seawater_seasim_SB9158_S7_bin_9': 'seawater', 'RHO1_bin_43': 'Rhopaloides odorabile', 'APA_bin_24': 'Aplysina aerophoba', 'CHO1_bin_1': 'Chondrilla australiensis', 'RHO1_bin_1': 'Rhopaloides odorabile', 'IRC_PAM_SB0666_bin_15': 'Ircinia ramosa', 'RHO3_bin_43': 'Rhopaloides odorabile', 'IRC4_bin_16': 'Ircinia ramosa', 'IRC3_bin_12': 'Ircinia ramosa', 'seawater_bettina_36327_bin_15': 'seawater', 'COS36386_bin_5': 'Coscinoderma matthewsi', 'SPOO_karimiCosta_FZLR01': 'Spongia officinalis', 'COS2_bin_18': 'Coscinoderma matthewsi', 'COS36388_bin_9': 'Coscinoderma matthewsi', 'RHO3_bin_12': 'Rhopaloides odorabile', 'APA_bin_42': 'Aplysina aerophoba', 'PSE_podellAllen_GCA_003635205.1_ASM363520v1_genomic': 'Pseudoceratina sp.', 'APA_bin_71': 'Aplysina aerophoba', 'CAR4_bin_14': 'Carteriospongia foliascens', 'RHO1_bin_55': 'Rhopaloides odorabile', 'AXIM_hallam_GCA_000200715.1_genomic': 'Axinella mexicana', 'RHO3_bin_50': 'Rhopaloides odorabile', 'STY2_bin_6': 'Stylissa flabelliformis', 'COS36386_bin_9': 'Coscinoderma matthewsi', 'IRC_PAM_SB0678_bin_5': 'Ircinia ramosa', 'IRC_PAM_SB0666_bin_11': 'Ircinia ramosa', 'IRC_PAM_SB0662_bin_7': 'Ircinia ramosa', 'RHO1_bin_49': 'Rhopaloides odorabile', 'COS2_bin_7': 'Coscinoderma matthewsi', 'COS2_bin_12': 'Coscinoderma matthewsi', 'IRC_PAM_SB0662_bin_39': 'Ircinia ramosa', 'RHO3_bin_52': 'Rhopaloides odorabile', 'RHO1_bin_81': 'Rhopaloides odorabile', 'IRC1_bin_17': 'Ircinia ramosa', 'RHO1_bin_51': 'Rhopaloides odorabile', 'COS36386_bin_27': 'Coscinoderma matthewsi', 'IRC_PAM_SB0665_bin_20': 'Ircinia ramosa', 'RHO1_bin_23': 'Rhopaloides odorabile', 'RHO1_bin_58': 'Rhopaloides odorabile', 'COS36386_bin_29': 'Coscinoderma matthewsi', 'IRC4_bin_17': 'Ircinia ramosa', 'CLI1_bin_2': 'Cliona orientalis', 'SPOO_karimiCosta2019_GCF_900143535.1_Tateyamaria_sp._Alg231_49_genomic': 'Spongia officinalis', 'IRC_PAM_SB0662_bin_37': 'Ircinia ramosa', 'COS36386_bin_13': 'Coscinoderma matthewsi', 'CHO1_bin_4': 'Chondrilla australiensis', 'STY3_bin_3': 'Stylissa flabelliformis', 'RHO1_bin_48': 'Rhopaloides odorabile', 'APA_bin_17': 'Aplysina aerophoba', 'COS1_bin_8': 'Coscinoderma matthewsi', 'IRC_PAM_SB0662_bin_8': 'Ircinia ramosa', 'CAR4_bin_2': 'Carteriospongia foliascens', 'TED_estevesThomas2016_Ruegeria_spAU67_2606217183': 'Tedania\xa0sp.', 'IRC_PAM_SB0675_bin_23': 'Ircinia ramosa', 'RHO1_bin_17': 'Rhopaloides odorabile', 'IRC1_bin_20': 'Ircinia ramosa', 'IRC4_bin_44': 'Ircinia ramosa', 'IRC_PAM_SB0661_bin_5': 'Ircinia ramosa', 'seawater_seasim_SB9157_S6_bin_7': 'seawater', 'IRC1_bin_10': 'Ircinia ramosa', 'COS36387_bin_9': 'Coscinoderma matthewsi', 'seawater_seasim_SB9155_S4_bin_4': 'seawater', 'IRC_PAM_SB0665_bin_13': 'Ircinia ramosa', 'CAR3_bin_12': 'Carteriospongia foliascens', 'RHO2_bin_51': 'Rhopaloides odorabile', 'APA_bin_8': 'Aplysina aerophoba', 'APA_bin_13': 'Aplysina aerophoba', 'CAR3_bin_7': 'Carteriospongia foliascens', 'RHO2_bin_13': 'Rhopaloides odorabile', 'RHO3_bin_35': 'Rhopaloides odorabile', 'APA_bin_5': 'Aplysina aerophoba', 'RHO2_bin_10': 'Rhopaloides odorabile', 'APA_bin_10': 'Aplysina aerophoba', 'COS2_bin_22': 'Coscinoderma matthewsi', 'CLI1_bin_3': 'Cliona orientalis', 'IRC_PAM_SB0661_bin_38': 'Ircinia ramosa', 'CAR1_bin_13': 'Carteriospongia foliascens', 'POLP_alexAntunes_GCF_001431305.1_ASM143130v1_genomic': 'Polymastia penicillus', 'IRC3_bin_9': 'Ircinia ramosa', 'seawater_22112_bin_3': 'seawater', 'seawater_bettina_36327_bin_7': 'seawater', 'COS4_bin_15': 'Coscinoderma matthewsi', 'RHO3_bin_54': 'Rhopaloides odorabile', 'CYMC_moitinhoThomas_67496.assembled': 'Cymbastela concentrica', 'CAR2_bin_5': 'Carteriospongia foliascens', 'IRC_PAM_SB0662_bin_19': 'Ircinia ramosa', 'COS36387_bin_1': 'Coscinoderma matthewsi', 'IRC3_bin_28': 'Ircinia ramosa', 'CAR2_bin_26': 'Carteriospongia foliascens', 'COS36386_bin_20': 'Coscinoderma matthewsi', 'COS3_bin_10': 'Coscinoderma matthewsi', 'RHO1_bin_59': 'Rhopaloides odorabile', 'DYSA_liuLi2012_GCF_000264395.1_C89_version_1_genomic': 'Dysidea avara', 'RHO3_bin_16': 'Rhopaloides odorabile', 'IRCvar_burgsdorfSteindler_GCA_001007625.1_ASM100762v1_genomic': 'Ircinia variabilis', 'CAR4_bin_15': 'Carteriospongia foliascens', 'COS4_bin_7': 'Coscinoderma matthewsi', 'COS36404_bin_9': 'Coscinoderma matthewsi', 'COS36387_bin_7': 'Coscinoderma matthewsi', 'IRC_PAM_SB0664_bin_3': 'Ircinia ramosa', 'IRC_PAM_SB0663_bin_5': 'Ircinia ramosa', 'IRC3_bin_22': 'Ircinia ramosa', 'IRC_PAM_SB0665_bin_10': 'Ircinia ramosa', 'STY2_bin_4': 'Stylissa flabelliformis', 'RHO3_bin_10': 'Rhopaloides odorabile', 'IRC_PAM_SB0667_bin_3': 'Ircinia ramosa', 'IRC_PAM_SB0662_bin_24': 'Ircinia ramosa', 'APA_bin_6': 'Aplysina aerophoba', 'RHO3_bin_19': 'Rhopaloides odorabile', 'CAR1_bin_12': 'Carteriospongia foliascens', 'COS36406_bin_1': 'Coscinoderma matthewsi', 'COS36387_bin_6': 'Coscinoderma matthewsi', 'STY1_bin_5': 'Stylissa flabelliformis', 'CAR2_bin_18': 'Carteriospongia foliascens', 'STY4_bin_1': 'Stylissa flabelliformis', 'RHO1_bin_50': 'Rhopaloides odorabile', 'COS1_bin_5': 'Coscinoderma matthewsi', 'IRC_PAM_SB0666_bin_9': 'Ircinia ramosa', 'seawater_42618_bin_5': 'seawater', 'IRC_PAM_SB0662_bin_33': 'Ircinia ramosa', 'RHO3_bin_20': 'Rhopaloides odorabile', 'RHO1_bin_53': 'Rhopaloides odorabile', 'IRC3_bin_4': 'Ircinia ramosa', 'SPOO_karimiCosta2019_GCF_900143615.1_Rhodobacteraceae_bacterium_Alg231_30_genomic': 'Spongia officinalis', 'seawater_42618_bin_3': 'seawater', 'COS36386_bin_1': 'Coscinoderma matthewsi', 'RHO1_bin_20': 'Rhopaloides odorabile', 'CLI2_bin_1': 'Cliona orientalis', 'STY3_bin_1': 'Stylissa flabelliformis', 'PSE_podellAllen_GCA_003635265.1_ASM363526v1_genomic': 'Pseudoceratina sp.', 'APA_bin_58': 'Aplysina aerophoba', 'COS1_bin_20': 'Coscinoderma matthewsi', 'IRC_PAM_SB0666_bin_17': 'Ircinia ramosa', 'RHO2_bin_26': 'Rhopaloides odorabile', 'RHO1_bin_24': 'Rhopaloides odorabile', 'APA_bin_56': 'Aplysina aerophoba', 'seawater_bettina_36308_bin_18': 'seawater', 'CAR3_bin_15': 'Carteriospongia foliascens', 'IRC_PAM_SB0664_bin_21': 'Ircinia ramosa', 'IRC4_bin_29': 'Ircinia ramosa', 'seawater_seasim_SB9156_S5_bin_4': 'seawater', 'COS36405_bin_7': 'Coscinoderma matthewsi', 'seawater_bettina_36328_bin_7': 'seawater', 'IRC2_bin_11': 'Ircinia ramosa', 'APA_bin_74': 'Aplysina aerophoba', 'SPOO_karimiCosta2019_GCF_900149705.1_Sphingorhabdus_sp._Alg231_15_genomic': 'Spongia officinalis', 'IRC_PAM_SB0661_bin_17': 'Ircinia ramosa', 'RHO1_bin_67': 'Rhopaloides odorabile', 'COS36388_bin_13': 'Coscinoderma matthewsi', 'COS2_bin_19': 'Coscinoderma matthewsi', 'IRC_PAM_SB0665_bin_4': 'Ircinia ramosa', 'IRC3_bin_32': 'Ircinia ramosa', 'seawater_22112_bin_5': 'seawater', 'APA_bin_55': 'Aplysina aerophoba', 'IRC_PAM_SB0675_bin_4': 'Ircinia ramosa', 'APA_bin_26': 'Aplysina aerophoba', 'APA_bin_91': 'Aplysina aerophoba', 'IRC_PAM_SB0677_bin_19': 'Ircinia ramosa', 'RHO1_bin_7': 'Rhopaloides odorabile', 'RHO3_bin_65': 'Rhopaloides odorabile', 'RHO1_bin_10': 'Rhopaloides odorabile', 'RHO1_bin_19': 'Rhopaloides odorabile', 'RHO3_bin_53': 'Rhopaloides odorabile', 'seawater_bettina_36326_bin_7': 'seawater', 'RHO1_bin_70': 'Rhopaloides odorabile', 'COS36388_bin_8': 'Coscinoderma matthewsi', 'seawater_bettina_36328_bin_8': 'seawater', 'seawater_22112_bin_17': 'seawater', 'COS36406_bin_2': 'Coscinoderma matthewsi', 'COS36406_bin_4': 'Coscinoderma matthewsi', 'IRC_PAM_SB0668_bin_19': 'Ircinia ramosa'}

sponge_with_high_HGT_preferences_list = ['Ircinia ramosa', 'Rhopaloides odorabile', 'Coscinoderma matthewsi', 'Aplysina aerophoba', 'Carteriospongia foliascens']

taxon_assignment_dict = {'RHO2_bin_6': ['d__Bacteria', 'p__Chloroflexota', 'c__Dehalococcoidia', 'o__UBA2963', 'f__UBA2963', 'g__Bin16', 's__'], 'IRC2_bin_5': ['d__Bacteria', 'p__Acidobacteriota', 'c__Thermoanaerobaculia', 'o__UBA5704', 'f__', 'g__', 's__'], 'RHO3_bin_70': ['d__Bacteria', 'p__Poribacteria', 'c__WGA-4E', 'o__WGA-4E', 'f__WGA-3G', 'g__WGA-3G', 's__'], 'RHO3_bin_39': ['d__Bacteria', 'p__Deinococcota', 'c__Deinococci', 'o__Deinococcales', 'f__Trueperaceae', 'g__MPNL01', 's__MPNL01 sp002239005'], 'APA_bin_82': ['d__Bacteria', 'p__Proteobacteria', 'c__Alphaproteobacteria', 'o__Bin98', 'f__Bin98', 'g__Bin98', 's__Bin98 sp002238905'], 'RHO3_bin_57': ['d__Bacteria', 'p__Proteobacteria', 'c__Alphaproteobacteria', 'o__Defluviicoccales', 'f__Defluviicoccaceae', 'g__Defluviicoccus', 's__'], 'GCA_000583135': ['d__Bacteria', 'p__Proteobacteria', 'c__Gammaproteobacteria', 'o__HK1', 'f__HK1', 'g__HK1', 's__HK1 sp000583135'], 'IRC_PAM_SB0670_bin_12': ['d__Bacteria', 'p__Dadabacteria', 'c__UBA1144', 'o__', 'f__', 'g__', 's__'], 'RHO1_bin_53': ['d__Bacteria', 'p__Proteobacteria', 'c__Gammaproteobacteria', 'o__Pseudomonadales', 'f__HTCC2089', 'g__', 's__'], 'seawater_seasim_SB9157_S6_bin_2': ['d__Bacteria', 'p__Marinisomatota', 'c__Marinisomatia', 'o__Marinisomatales', 'f__TCS55', 'g__TCS55', 's__TCS55 sp001577025'], 'CAR2_bin_26': ['d__Bacteria', 'p__Proteobacteria', 'c__Gammaproteobacteria', 'o__UBA10353', 'f__LS-SOB', 'g__', 's__'], 'GCF_900149695': ['d__Bacteria', 'p__Proteobacteria', 'c__Alphaproteobacteria', 'o__Rhizobiales', 'f__Anderseniellaceae', 'g__Anderseniella', 's__Anderseniella sp900149695'], 'IRC_PAM_SB0668_bin_5': ['d__Bacteria', 'p__Actinobacteriota', 'c__Acidimicrobiia', 'o__Microtrichales', 'f__UBA11606', 'g__UBA11606', 's__'], 'RHO3_bin_15': ['d__Bacteria', 'p__Dadabacteria', 'c__UBA1144', 'o__', 'f__', 'g__', 's__'], 'IRC_PAM_SB0678_bin_2': ['d__Bacteria', 'p__Proteobacteria', 'c__Gammaproteobacteria', 'o__Pseudomonadales', 'f__Pseudohongiellaceae', 'g__UBA9145', 's__'], 'IRC_PAM_SB0661_bin_39': ['d__Bacteria', 'p__Chloroflexota', 'c__Dehalococcoidia', 'o__UBA6926', 'f__UBA6926', 'g__', 's__'], 'APA_bin_58': ['d__Bacteria', 'p__Acidobacteriota', 'c__Vicinamibacteria', 'o__Vicinamibacterales', 'f__UBA8438', 'g__UBA8438', 's__'], 'COS36388_bin_8': ['d__Bacteria', 'p__Planctomycetota', 'c__Planctomycetes', 'o__Pirellulales', 'f__UBA1268', 'g__UBA1268', 's__UBA1268 sp002694955'], 'COS4_bin_28': ['d__Bacteria', 'p__Proteobacteria', 'c__Alphaproteobacteria', 'o__Bin65', 'f__Bin65', 'g__Bin65', 's__'], 'IRC_PAM_SB0662_bin_1': ['d__Bacteria', 'p__Chloroflexota_B', 'c__UBA11872', 'o__UBA11872', 'f__', 'g__', 's__'], 'IRC_PAM_SB0662_bin_26': ['d__Bacteria', 'p__Nitrospirota', 'c__Nitrospiria', 'o__Nitrospirales', 'f__UBA8639', 'g__Bin75', 's__'], 'IRC1_bin_1': ['d__Bacteria', 'p__Proteobacteria', 'c__Gammaproteobacteria', 'o__Pseudomonadales', 'f__Pseudohongiellaceae', 'g__UBA9145', 's__'], 'RHO1_bin_56': ['d__Bacteria', 'p__Gemmatimonadota', 'c__Gemmatimonadetes', 'o__SG8-23', 'f__UBA6960', 'g__Bin94', 's__'], 'RHO3_bin_23': ['d__Bacteria', 'p__Chloroflexota_B', 'c__UBA11872', 'o__UBA11872', 'f__UBA11872', 'g__', 's__'], 'COS4_bin_3': ['d__Bacteria', 'p__Acidobacteriota', 'c__Thermoanaerobaculia', 'o__UBA5704', 'f__', 'g__', 's__'], 'RHO2_bin_10': ['d__Bacteria', 'p__Actinobacteriota', 'c__Acidimicrobiia', 'o__UBA5794', 'f__SZUA-232', 'g__', 's__'], 'IRC_PAM_SB0665_bin_13': ['d__Bacteria', 'p__Acidobacteriota', 'c__Bin61', 'o__Bin61', 'f__Bin61', 'g__Bin61', 's__'], 'IRC_PAM_SB0662_bin_27': ['d__Bacteria', 'p__Chloroflexota', 'c__Dehalococcoidia', 'o__UBA3495', 'f__UBA3495', 'g__Bin87', 's__'], 'STY2_bin_7': ['d__Bacteria', 'p__Cyanobacteria', 'c__Cyanobacteriia', 'o__Synechococcales', 'f__Cyanobiaceae', 'g__Synechococcus_C', 's__Synechococcus_C sp002724845'], 'RHO2_bin_22': ['d__Bacteria', 'p__Chloroflexota', 'c__Anaerolineae', 'o__SBR1031', 'f__A4b', 'g__GCA-2702065', 's__'], 'IRC_PAM_SB0675_bin_16': ['d__Bacteria', 'p__Actinobacteriota', 'c__Acidimicrobiia', 'o__Microtrichales', 'f__UBA11606', 'g__', 's__'], 'IRC_PAM_SB0661_bin_14': ['d__Bacteria', 'p__Actinobacteriota', 'c__Acidimicrobiia', 'o__UBA5794', 'f__Bin76', 'g__Bin76', 's__'], 'APA_bin_91': ['d__Bacteria', 'p__UBA8248', 'c__UBA8248', 'o__UBA8248', 'f__UBA8248', 'g__Bin107', 's__Bin107 sp002238965'], 'COS36387_bin_10': ['d__Bacteria', 'p__Actinobacteriota', 'c__Acidimicrobiia', 'o__UBA5794', 'f__SZUA-232', 'g__', 's__'], 'RHO1_bin_4': ['d__Bacteria', 'p__Chloroflexota', 'c__Dehalococcoidia', 'o__UBA3495', 'f__UBA3495', 'g__Bin22', 's__'], 'IRC_PAM_SB0664_bin_33': ['d__Bacteria', 'p__Chloroflexota', 'c__Dehalococcoidia', 'o__SAR202', 'f__UBA11138', 'g__', 's__'], 'CAR4_bin_10': ['d__Bacteria', 'p__Bacteroidota', 'c__Rhodothermia', 'o__Rhodothermales', 'f__Bin80', 'g__Bin80', 's__'], 'COS36386_bin_15': ['d__Bacteria', 'p__Proteobacteria', 'c__Gammaproteobacteria', 'o__UBA10353', 'f__UBA5680', 'g__', 's__'], 'IRC4_bin_9': ['d__Bacteria', 'p__Actinobacteriota', 'c__Acidimicrobiia', 'o__Microtrichales', 'f__Bin134', 'g__Bin134', 's__'], 'COS36405_bin_1': ['d__Bacteria', 'p__Nitrospirota', 'c__Nitrospiria', 'o__Nitrospirales', 'f__UBA8639', 'g__Bin75', 's__'], 'COS1_bin_1': ['d__Bacteria', 'p__Chloroflexota', 'c__Anaerolineae', 'o__Caldilineales', 'f__Caldilineaceae', 'g__Bin5', 's__'], 'COS4_bin_24': ['d__Bacteria', 'p__Dadabacteria', 'c__UBA1144', 'o__', 'f__', 'g__', 's__'], 'COS36386_bin_2': ['d__Bacteria', 'p__Nitrospirota', 'c__Nitrospiria', 'o__Nitrospirales', 'f__UBA8639', 'g__Bin75', 's__'], 'RHO2_bin_12': ['d__Bacteria', 'p__Proteobacteria', 'c__Gammaproteobacteria', 'o__UBA10353', 'f__UBA5680', 'g__', 's__'], 'Ruegeria_spAU67': ['d__Bacteria', 'p__Proteobacteria', 'c__Alphaproteobacteria', 'o__Rhodobacterales', 'f__Rhodobacteraceae', 'g__Ruegeria', 's__Ruegeria atlantica'], 'RHO3_bin_32': ['d__Bacteria', 'p__Actinobacteriota', 'c__Acidimicrobiia', 'o__UBA5794', 'f__Bin76', 'g__Bin76', 's__'], 'seawater_bettina_36327_bin_15': ['d__Bacteria', 'p__Bacteroidota', 'c__Bacteroidia', 'o__Flavobacteriales', 'f__Crocinitomicaceae', 'g__UBA952', 's__'], 'STY4_bin_4': ['d__Bacteria', 'p__Proteobacteria', 'c__Gammaproteobacteria', 'o__UBA10353', 'f__LS-SOB', 'g__', 's__'], 'COS2_bin_5': ['d__Bacteria', 'p__Acidobacteriota', 'c__Thermoanaerobaculia', 'o__UBA5704', 'f__', 'g__', 's__'], 'IRC_PAM_SB0664_bin_12': ['d__Bacteria', 'p__Proteobacteria', 'c__Gammaproteobacteria', 'o__Pseudomonadales', 'f__HTCC2089', 'g__', 's__'], 'RHO3_bin_25': ['d__Bacteria', 'p__Chloroflexota', 'c__Dehalococcoidia', 'o__UBA2991', 'f__UBA2991', 'g__UBA2991', 's__'], 'COS2_bin_22': ['d__Bacteria', 'p__Chloroflexota', 'c__Anaerolineae', 'o__SBR1031', 'f__A4b', 'g__UBA6055', 's__'], 'COS4_bin_32': ['d__Bacteria', 'p__Chloroflexota', 'c__Anaerolineae', 'o__SBR1031', 'f__A4b', 'g__UBA6055', 's__'], 'CAR2_bin_7': ['d__Bacteria', 'p__Proteobacteria', 'c__Alphaproteobacteria', 'o__Rhodobacterales', 'f__Rhodobacteraceae', 'g__Rhodobacter_B', 's__'], 'IRC2_bin_10': ['d__Bacteria', 'p__Actinobacteriota', 'c__Acidimicrobiia', 'o__Microtrichales', 'f__UBA11606', 'g__', 's__'], 'IRC4_bin_11': ['d__Bacteria', 'p__Chloroflexota', 'c__Dehalococcoidia', 'o__UBA3495', 'f__UBA3495', 'g__Bin87', 's__'], 'CAR2_bin_8': ['d__Bacteria', 'p__Proteobacteria', 'c__Gammaproteobacteria', 'o__UBA10353', 'f__LS-SOB', 'g__', 's__'], 'IRC3_bin_31': ['d__Bacteria', 'p__Gemmatimonadota', 'c__Gemmatimonadetes', 'o__SG8-23', 'f__UBA6960', 'g__Bin94', 's__'], 'RHO1_bin_81': ['d__Bacteria', 'p__Acidobacteriota', 'c__Vicinamibacteria', 'o__Vicinamibacterales', 'f__UBA8438', 'g__UBA8438', 's__'], 'COS36386_bin_6': ['d__Bacteria', 'p__Planctomycetota', 'c__Planctomycetes', 'o__Pirellulales', 'f__UBA1268', 'g__UBA1268', 's__UBA1268 sp002694955'], 'seawater_seasim_SB9160_S9_bin_9': ['d__Bacteria', 'p__Verrucomicrobiota', 'c__Verrucomicrobiae', 'o__Pedosphaerales', 'f__UBA1096', 'g__UBA1096', 's__'], 'IRC2_bin_6': ['d__Bacteria', 'p__Chloroflexota', 'c__Anaerolineae', 'o__SBR1031', 'f__A4b', 'g__UBA6055', 's__'], 'IRC_PAM_SB0677_bin_11': ['d__Bacteria', 'p__Actinobacteriota', 'c__Acidimicrobiia', 'o__Microtrichales', 'f__Bin134', 'g__Bin134', 's__'], 'RHO1_bin_15': ['d__Bacteria', 'p__Chloroflexota', 'c__Anaerolineae', 'o__SBR1031', 'f__A4b', 'g__UBA6055', 's__'], 'seawater_seasim_SB9156_S5_bin_14': ['d__Bacteria', 'p__Cyanobacteria', 'c__Vampirovibrionia', 'o__', 'f__', 'g__', 's__'], 'seawater_seasim_SB9152_S1_bin_3': ['d__Bacteria', 'p__Proteobacteria', 'c__Alphaproteobacteria', 'o__UBA1280', 'f__', 'g__', 's__'], 'seawater_seasim_SB9154_S3_bin_1': ['d__Bacteria', 'p__Proteobacteria', 'c__Alphaproteobacteria', 'o__Rhizobiales', 'f__Rhizobiaceae', 'g__', 's__'], 'CAR3_bin_7': ['d__Bacteria', 'p__Actinobacteriota', 'c__Acidimicrobiia', 'o__Microtrichales', 'f__Bin134', 'g__Bin134', 's__'], 'seawater_bettina_36326_bin_1': ['d__Bacteria', 'p__Proteobacteria', 'c__Alphaproteobacteria', 'o__Puniceispirillales', 'f__Puniceispirillaceae', 'g__UBA8309', 's__UBA8309 sp002695585'], 'APA_bin_85': ['d__Bacteria', 'p__Gemmatimonadota', 'c__Gemmatimonadetes', 'o__SG8-23', 'f__UBA6960', 'g__Bin94', 's__Bin94 sp002238865'], 'seawater_bettina_36327_bin_4': ['d__Bacteria', 'p__Bacteroidota', 'c__Bacteroidia', 'o__Flavobacteriales', 'f__Flavobacteriaceae', 'g__UBA3478', 's__'], 'seawater_42617_bin_6': ['d__Bacteria', 'p__Bacteroidota', 'c__Bacteroidia', 'o__Flavobacteriales', 'f__Cryomorphaceae', 'g__UBA10364', 's__UBA10364 sp003445735'], 'APA_bin_53': ['d__Bacteria', 'p__Actinobacteriota', 'c__Acidimicrobiia', 'o__Microtrichales', 'f__UBA11606', 'g__UBA11606', 's__'], 'IRC_PAM_SB0675_bin_4': ['d__Bacteria', 'p__Actinobacteriota', 'c__Acidimicrobiia', 'o__Microtrichales', 'f__TK06', 'g__', 's__'], 'IRC_PAM_SB0664_bin_15': ['d__Bacteria', 'p__Chloroflexota', 'c__Dehalococcoidia', 'o__Bin125', 'f__Bin125', 'g__Bin125', 's__'], 'RHO3_bin_18': ['d__Bacteria', 'p__Actinobacteriota', 'c__Acidimicrobiia', 'o__Microtrichales', 'f__UBA11606', 'g__', 's__'], 'IRC_PAM_SB0661_bin_45': ['d__Bacteria', 'p__Chloroflexota', 'c__Dehalococcoidia', 'o__UBA3495', 'f__UBA3495', 'g__', 's__'], 'IRC_PAM_SB0661_bin_4': ['d__Bacteria', 'p__Actinobacteriota', 'c__Acidimicrobiia', 'o__UBA5794', 'f__SZUA-232', 'g__', 's__'], 'IRC4_bin_42': ['d__Bacteria', 'p__Proteobacteria', 'c__Alphaproteobacteria', 'o__UBA7887', 'f__GCA-2721365', 'g__', 's__'], 'RHO1_bin_44': ['d__Bacteria', 'p__Spirochaetota', 'c__Spirochaetia', 'o__Spirochaetales', 'f__RBG-16-67-19', 'g__Bin103', 's__'], 'IRC_PAM_SB0662_bin_8': ['d__Bacteria', 'p__Gemmatimonadota', 'c__Gemmatimonadetes', 'o__SG8-23', 'f__UBA6960', 'g__Bin94', 's__'], 'IRC4_bin_21': ['d__Bacteria', 'p__Chloroflexota', 'c__Dehalococcoidia', 'o__UBA2991', 'f__UBA2991', 'g__UBA2991', 's__'], 'RHO2_bin_33': ['d__Bacteria', 'p__Proteobacteria', 'c__Gammaproteobacteria', 'o__Pseudomonadales', 'f__HTCC2089', 'g__UBA2168', 's__'], 'CLI1_bin_2': ['d__Bacteria', 'p__Proteobacteria', 'c__Gammaproteobacteria', 'o__Pseudomonadales', 'f__', 'g__', 's__'], 'RHO1_bin_30': ['d__Bacteria', 'p__Poribacteria', 'c__WGA-4E', 'o__WGA-4E', 'f__WGA-3G', 'g__WGA-3G', 's__'], 'IRC4_bin_30': ['d__Bacteria', 'p__Chloroflexota', 'c__Dehalococcoidia', 'o__SAR202', 'f__', 'g__', 's__'], 'GCF_000158135': ['d__Bacteria', 'p__Proteobacteria', 'c__Alphaproteobacteria', 'o__Rhodobacterales', 'f__Rhodobacteraceae', 'g__Ruegeria', 's__Ruegeria sp000158135'], 'SPOO_karimiCosta_FZLS01': ['d__Bacteria', 'p__Proteobacteria', 'c__Alphaproteobacteria', 'o__Rhodobacterales', 'f__Rhodobacteraceae', 'g__Amylibacter_A', 's__Amylibacter_A sp900197625'], 'IRC_PAM_SB0662_bin_35': ['d__Bacteria', 'p__Poribacteria', 'c__WGA-4E', 'o__WGA-4E', 'f__WGA-3G', 'g__', 's__'], 'RHO2_bin_35': ['d__Bacteria', 'p__Dadabacteria', 'c__UBA1144', 'o__', 'f__', 'g__', 's__'], 'CAR4_bin_14': ['d__Bacteria', 'p__Chloroflexota', 'c__Anaerolineae', 'o__SBR1031', 'f__A4b', 'g__UBA6055', 's__'], 'COS1_bin_4': ['d__Bacteria', 'p__Actinobacteriota', 'c__Thermoleophilia', 'o__20CM-4-69-9', 'f__', 'g__', 's__'], 'COS1_bin_8': ['d__Bacteria', 'p__Proteobacteria', 'c__Gammaproteobacteria', 'o__Pseudomonadales', 'f__HTCC2089', 'g__Bin55', 's__'], 'RHO3_bin_67': ['d__Bacteria', 'p__Chloroflexota', 'c__Dehalococcoidia', 'o__Bin125', 'f__Bin125', 'g__Bin125', 's__'], 'IRC1_bin_35': ['d__Bacteria', 'p__Actinobacteriota', 'c__Acidimicrobiia', 'o__Microtrichales', 'f__UBA11606', 'g__', 's__'], 'APA_bin_14': ['d__Bacteria', 'p__Gemmatimonadota', 'c__Gemmatimonadetes', 'o__SG8-23', 'f__UBA6960', 'g__BD2-11', 's__BD2-11 sp002238605'], 'RHO3_bin_16': ['d__Bacteria', 'p__Acidobacteriota', 'c__UBA6911', 'o__', 'f__', 'g__', 's__'], 'IRC_PAM_SB0668_bin_20': ['d__Bacteria', 'p__Latescibacterota', 'c__', 'o__', 'f__', 'g__', 's__'], 'APA_bin_94': ['d__Bacteria', 'p__Spirochaetota', 'c__Spirochaetia', 'o__Spirochaetales', 'f__RBG-16-67-19', 'g__', 's__'], 'IRC_PAM_SB0661_bin_16': ['d__Bacteria', 'p__Chloroflexota', 'c__Dehalococcoidia', 'o__Bin125', 'f__Bin125', 'g__Bin125', 's__'], 'IRC_PAM_SB0676_bin_21': ['d__Bacteria', 'p__Acidobacteriota', 'c__Bin61', 'o__Bin61', 'f__Bin61', 'g__Bin61', 's__'], 'IRC_PAM_SB0677_bin_2': ['d__Bacteria', 'p__Proteobacteria', 'c__Gammaproteobacteria', 'o__UBA10353', 'f__LS-SOB', 'g__', 's__'], 'RHO3_bin_31': ['d__Bacteria', 'p__Actinobacteriota', 'c__Acidimicrobiia', 'o__UBA5794', 'f__Bin76', 'g__Bin76', 's__'], 'IRC_PAM_SB0662_bin_39': ['d__Bacteria', 'p__Acidobacteriota', 'c__Acidobacteriae', 'o__Bryobacterales', 'f__UBA6623', 'g__', 's__'], 'COS36386_bin_37': ['d__Bacteria', 'p__Cyanobacteria', 'c__Cyanobacteriia', 'o__Synechococcales', 'f__Cyanobiaceae', 'g__Synechococcus_C', 's__'], 'GCF_004168585': ['d__Bacteria', 'p__Proteobacteria', 'c__Gammaproteobacteria', 'o__Enterobacterales', 'f__Shewanellaceae', 'g__', 's__'], 'IRC_PAM_SB0670_bin_20': ['d__Bacteria', 'p__Cyanobacteria', 'c__Cyanobacteriia', 'o__Synechococcales', 'f__Cyanobiaceae', 'g__Synechococcus_B', 's__'], 'COS4_bin_9': ['d__Bacteria', 'p__Latescibacterota', 'c__UBA2968', 'o__UBA8231', 'f__UBA8231', 'g__UBA8231', 's__'], 'IRC3_bin_28': ['d__Bacteria', 'p__Gemmatimonadota', 'c__Gemmatimonadetes', 'o__', 'f__', 'g__', 's__'], 'COS3_bin_16': ['d__Bacteria', 'p__Actinobacteriota', 'c__Acidimicrobiia', 'o__Microtrichales', 'f__Bin134', 'g__', 's__'], 'IRC_PAM_SB0661_bin_41': ['d__Bacteria', 'p__Gemmatimonadota', 'c__Gemmatimonadetes', 'o__', 'f__', 'g__', 's__'], 'RHO3_bin_49': ['d__Bacteria', 'p__Chloroflexota', 'c__Anaerolineae', 'o__SBR1031', 'f__A4b', 'g__UBA6055', 's__'], 'IRC_PAM_SB0678_bin_6': ['d__Bacteria', 'p__Chloroflexota', 'c__Anaerolineae', 'o__SBR1031', 'f__A4b', 'g__UBA6055', 's__'], 'seawater_42618_bin_6': ['d__Bacteria', 'p__Actinobacteriota', 'c__Acidimicrobiia', 'o__Microtrichales', 'f__UBA8592', 'g__MedAcidi-G2A', 's__MedAcidi-G2A sp002380145'], 'RHO2_bin_5': ['d__Bacteria', 'p__Latescibacterota', 'c__UBA2968', 'o__UBA2968', 'f__GCA-2709665', 'g__', 's__'], 'RHO2_bin_57': ['d__Bacteria', 'p__Actinobacteriota', 'c__Acidimicrobiia', 'o__UBA5794', 'f__Bin76', 'g__Bin76', 's__'], 'seawater_42618_bin_25': ['d__Bacteria', 'p__Proteobacteria', 'c__Gammaproteobacteria', 'o__Pseudomonadales', 'f__Litoricolaceae', 'g__Litoricola', 's__Litoricola sp002705795'], 'COS36386_bin_27': ['d__Bacteria', 'p__Binatota', 'c__Binatia', 'o__Bin18', 'f__Bin18', 'g__Bin18', 's__'], 'IRC_PAM_SB0662_bin_53': ['d__Bacteria', 'p__Proteobacteria', 'c__Gammaproteobacteria', 'o__UBA4575', 'f__UBA4575', 'g__', 's__'], 'CAR4_bin_4': ['d__Bacteria', 'p__Actinobacteriota', 'c__Acidimicrobiia', 'o__Microtrichales', 'f__UBA11606', 'g__', 's__'], 'GCF_900149705': ['d__Bacteria', 'p__Proteobacteria', 'c__Alphaproteobacteria', 'o__Sphingomonadales', 'f__Sphingomonadaceae', 'g__Sphingorhabdus', 's__Sphingorhabdus sp900149705'], 'COS1_bin_16': ['d__Bacteria', 'p__Acidobacteriota', 'c__Vicinamibacteria', 'o__Vicinamibacterales', 'f__UBA8438', 'g__', 's__'], 'IRC_PAM_SB0668_bin_6': ['d__Bacteria', 'p__Actinobacteriota', 'c__Acidimicrobiia', 'o__Microtrichales', 'f__Bin134', 'g__', 's__'], 'IRC4_bin_6': ['d__Bacteria', 'p__Cyanobacteria', 'c__Cyanobacteriia', 'o__Synechococcales', 'f__Cyanobiaceae', 'g__Synechococcus_B', 's__'], 'IRC_PAM_SB0664_bin_25': ['d__Bacteria', 'p__Actinobacteriota', 'c__Acidimicrobiia', 'o__UBA5794', 'f__SZUA-232', 'g__', 's__'], 'IRC4_bin_10': ['d__Bacteria', 'p__Actinobacteriota', 'c__Acidimicrobiia', 'o__Microtrichales', 'f__UBA11606', 'g__UBA11606', 's__'], 'STY1_bin_1': ['d__Bacteria', 'p__Proteobacteria', 'c__Gammaproteobacteria', 'o__UBA10353', 'f__LS-SOB', 'g__', 's__'], 'IRC3_bin_35': ['d__Bacteria', 'p__Chloroflexota', 'c__Anaerolineae', 'o__SBR1031', 'f__A4b', 'g__UBA6055', 's__'], 'RHO3_bin_8': ['d__Bacteria', 'p__Chloroflexota', 'c__Anaerolineae', 'o__SBR1031', 'f__A4b', 'g__GCA-2702065', 's__'], 'COS3_bin_6': ['d__Bacteria', 'p__Actinobacteriota', 'c__Acidimicrobiia', 'o__Microtrichales', 'f__Bin134', 'g__Bin134', 's__'], 'IRC_PAM_SB0661_bin_44': ['d__Bacteria', 'p__Chloroflexota', 'c__Dehalococcoidia', 'o__UBA2979', 'f__', 'g__', 's__'], 'APA_bin_93': ['d__Bacteria', 'p__Proteobacteria', 'c__Gammaproteobacteria', 'o__UBA4486', 'f__UBA4486', 'g__', 's__'], 'IRC_PAM_SB0661_bin_55': ['d__Bacteria', 'p__Actinobacteriota', 'c__Acidimicrobiia', 'o__Microtrichales', 'f__Bin134', 'g__', 's__'], 'IRC_PAM_SB0665_bin_8': ['d__Bacteria', 'p__Latescibacterota', 'c__', 'o__', 'f__', 'g__', 's__'], 'RHO1_bin_11': ['d__Bacteria', 'p__Poribacteria', 'c__WGA-4E', 'o__WGA-4E', 'f__WGA-3G', 'g__', 's__'], 'RHO1_bin_29': ['d__Bacteria', 'p__Proteobacteria', 'c__Alphaproteobacteria', 'o__Rhodospirillales_A', 'f__', 'g__', 's__'], 'IRC2_bin_11': ['d__Bacteria', 'p__Gemmatimonadota', 'c__Gemmatimonadetes', 'o__SG8-23', 'f__UBA6960', 'g__Bin94', 's__'], 'IRC_PAM_SB0665_bin_5': ['d__Bacteria', 'p__Actinobacteriota', 'c__Acidimicrobiia', 'o__Microtrichales', 'f__UBA11606', 'g__', 's__'], 'IRC4_bin_40': ['d__Bacteria', 'p__Acidobacteriota', 'c__Bin61', 'o__Bin61', 'f__Bin61', 'g__Bin61', 's__'], 'IRC_PAM_SB0668_bin_21': ['d__Bacteria', 'p__Chloroflexota', 'c__Anaerolineae', 'o__Caldilineales', 'f__Caldilineaceae', 'g__Bin5', 's__'], 'CAR3_bin_9': ['d__Bacteria', 'p__Proteobacteria', 'c__Alphaproteobacteria', 'o__Rhodobacterales', 'f__Rhodobacteraceae', 'g__Rhodobacter_B', 's__'], 'APA_bin_22': ['d__Bacteria', 'p__Proteobacteria', 'c__Alphaproteobacteria', 'o__SP197', 'f__SP197', 'g__SP197', 's__SP197 sp002238685'], 'seawater_22112_bin_14': ['d__Bacteria', 'p__Planctomycetota', 'c__Planctomycetes', 'o__Pirellulales', 'f__Pirellulaceae', 'g__Rubripirellula', 's__'], 'RHO3_bin_19': ['d__Bacteria', 'p__Proteobacteria', 'c__Gammaproteobacteria', 'o__UBA4486', 'f__UBA4486', 'g__', 's__'], 'COS36405_bin_5': ['d__Bacteria', 'p__Planctomycetota', 'c__Planctomycetes', 'o__Pirellulales', 'f__UBA1268', 'g__', 's__'], 'RHO3_bin_66': ['d__Bacteria', 'p__Spirochaetota', 'c__Spirochaetia', 'o__Spirochaetales', 'f__RBG-16-67-19', 'g__Bin103', 's__'], 'CAR3_bin_4': ['d__Bacteria', 'p__Proteobacteria', 'c__Alphaproteobacteria', 'o__Rhodobacterales', 'f__Rhodobacteraceae', 'g__Bin36', 's__'], 'seawater_seasim_SB9153_S2_bin_13': ['d__Bacteria', 'p__Myxococcota', 'c__Polyangia', 'o__Polyangiales', 'f__', 'g__', 's__'], 'IRC_PAM_SB0662_bin_37': ['d__Bacteria', 'p__Latescibacterota', 'c__UBA2968', 'o__UBA8231', 'f__GCA-002724215', 'g__GCA-2724215', 's__'], 'COS3_bin_14': ['d__Bacteria', 'p__Acidobacteriota', 'c__Thermoanaerobaculia', 'o__UBA5704', 'f__', 'g__', 's__'], 'IRC_PAM_SB0662_bin_20': ['d__Bacteria', 'p__Chloroflexota', 'c__Dehalococcoidia', 'o__SAR202', 'f__UBA11138', 'g__Bin90', 's__'], 'seawater_seasim_SB9157_S6_bin_13': ['d__Bacteria', 'p__Proteobacteria', 'c__Alphaproteobacteria', 'o__UBA8366', 'f__GCA-2717185', 'g__GCA-2717185', 's__'], 'IRC4_bin_20': ['d__Bacteria', 'p__Chloroflexota', 'c__Dehalococcoidia', 'o__UBA2979', 'f__', 'g__', 's__'], 'GCF_000743705': ['d__Bacteria', 'p__Proteobacteria', 'c__Alphaproteobacteria', 'o__Rhodobacterales', 'f__Rhodobacteraceae', 'g__Ruegeria', 's__Ruegeria halocynthiae_B'], 'IRC_PAM_SB0661_bin_24': ['d__Bacteria', 'p__Bacteroidota', 'c__Rhodothermia', 'o__Rhodothermales', 'f__Bin80', 'g__Bin80', 's__'], 'COS2_bin_16': ['d__Bacteria', 'p__Chloroflexota', 'c__Anaerolineae', 'o__SBR1031', 'f__', 'g__', 's__'], 'seawater_bettina_36326_bin_6': ['d__Bacteria', 'p__Proteobacteria', 'c__Alphaproteobacteria', 'o__Puniceispirillales', 'f__Puniceispirillaceae', 'g__UBA685', 's__'], 'APA_bin_34': ['d__Bacteria', 'p__Bacteroidota', 'c__Bacteroidia', 'o__Flavobacteriales', 'f__Flavobacteriaceae', 'g__Bin25', 's__Bin25 sp002238525'], 'GCA_002631715': ['d__Bacteria', 'p__Proteobacteria', 'c__Gammaproteobacteria', 'o__Porisulfidales', 'f__Porisulfidaceae', 'g__Porisulfidus', 's__Porisulfidus sp002631715'], 'IRC1_bin_11': ['d__Bacteria', 'p__Poribacteria', 'c__WGA-4E', 'o__WGA-4E', 'f__WGA-3G', 'g__WGA-3G', 's__'], 'RHO2_bin_55': ['d__Bacteria', 'p__Acidobacteriota', 'c__Bin61', 'o__Bin61', 'f__Bin61', 'g__Bin61', 's__'], 'STY4_bin_9': ['d__Bacteria', 'p__Cyanobacteria', 'c__Cyanobacteriia', 'o__Synechococcales', 'f__Cyanobiaceae', 'g__Synechococcus_C', 's__Synechococcus_C sp002724845'], 'CAR4_bin_16': ['d__Bacteria', 'p__Proteobacteria', 'c__Alphaproteobacteria', 'o__Rhodobacterales', 'f__Rhodobacteraceae', 'g__Aestuariivita', 's__'], 'COS4_bin_46': ['d__Bacteria', 'p__Latescibacterota', 'c__', 'o__', 'f__', 'g__', 's__'], 'IRC4_bin_33': ['d__Bacteria', 'p__Chloroflexota', 'c__Anaerolineae', 'o__Caldilineales', 'f__Bin34', 'g__Bin34', 's__'], 'CAR2_bin_9': ['d__Bacteria', 'p__Proteobacteria', 'c__Alphaproteobacteria', 'o__Bin95', 'f__Bin95', 'g__Bin95', 's__'], 'COS36404_bin_10': ['d__Bacteria', 'p__Acidobacteriota', 'c__Thermoanaerobaculia', 'o__UBA5704', 'f__', 'g__', 's__'], 'CAR4_bin_13': ['d__Bacteria', 'p__Bacteroidota', 'c__Bacteroidia', 'o__Flavobacteriales', 'f__Flavobacteriaceae', 'g__Bin25', 's__'], 'seawater_42615_bin_2': ['d__Bacteria', 'p__Proteobacteria', 'c__Alphaproteobacteria', 'o__Caulobacterales', 'f__Caulobacteraceae', 'g__Brevundimonas', 's__Brevundimonas sp001310255'], 'RHO3_bin_79': ['d__Bacteria', 'p__Chloroflexota', 'c__Dehalococcoidia', 'o__UBA1127', 'f__UBA1127', 'g__UBA1127', 's__'], 'IRC_PAM_SB0668_bin_19': ['d__Bacteria', 'p__Actinobacteriota', 'c__Acidimicrobiia', 'o__UBA5794', 'f__Bin76', 'g__Bin76', 's__'], 'seawater_22112_bin_28': ['d__Bacteria', 'p__Proteobacteria', 'c__Alphaproteobacteria', 'o__Rhodobacterales', 'f__Rhodobacteraceae', 'g__HIMB11', 's__HIMB11 sp002457055'], 'RHO3_bin_35': ['d__Bacteria', 'p__Actinobacteriota', 'c__Acidimicrobiia', 'o__Microtrichales', 'f__Bin134', 'g__Bin134', 's__'], 'IRC4_bin_35': ['d__Bacteria', 'p__Actinobacteriota', 'c__Acidimicrobiia', 'o__Microtrichales', 'f__UBA11606', 'g__', 's__'], 'APA_bin_63': ['d__Bacteria', 'p__Chloroflexota', 'c__Anaerolineae', 'o__SBR1031', 'f__A4b', 'g__UBA6055', 's__UBA6055 sp002238485'], 'IRC2_bin_12': ['d__Bacteria', 'p__Gemmatimonadota', 'c__Gemmatimonadetes', 'o__', 'f__', 'g__', 's__'], 'COS2_bin_10': ['d__Bacteria', 'p__Proteobacteria', 'c__Alphaproteobacteria', 'o__Rhodobacterales', 'f__Rhodobacteraceae', 'g__', 's__'], 'seawater_seasim_SB9153_S2_bin_2': ['d__Bacteria', 'p__Proteobacteria', 'c__Alphaproteobacteria', 'o__UBA1280', 'f__', 'g__', 's__'], 'CAR3_bin_6': ['d__Bacteria', 'p__Bacteroidota', 'c__Rhodothermia', 'o__Rhodothermales', 'f__Bin80', 'g__Bin80', 's__'], 'Aquimarina_spAU474': ['d__Bacteria', 'p__Bacteroidota', 'c__Bacteroidia', 'o__Flavobacteriales', 'f__Flavobacteriaceae', 'g__Aquimarina', 's__Aquimarina sp900312815'], 'GCF_000156235': ['d__Bacteria', 'p__Proteobacteria', 'c__Alphaproteobacteria', 'o__Rhizobiales', 'f__Stappiaceae', 'g__Pseudovibrio', 's__Pseudovibrio denitrificans'], 'APA_bin_12': ['d__Bacteria', 'p__Bdellovibrionota', 'c__Bacteriovoracia', 'o__Bacteriovoracales', 'f__Bacteriovoracaceae', 'g__', 's__'], 'IRC1_bin_3': ['d__Bacteria', 'p__Dadabacteria', 'c__UBA1144', 'o__', 'f__', 'g__', 's__'], 'IRC4_bin_23': ['d__Bacteria', 'p__Proteobacteria', 'c__Gammaproteobacteria', 'o__Pseudomonadales', 'f__HTCC2089', 'g__', 's__'], 'APA_bin_55': ['d__Bacteria', 'p__Proteobacteria', 'c__Alphaproteobacteria', 'o__UBA2966', 'f__UBA2966', 'g__UBA2966', 's__'], 'IRC4_bin_2': ['d__Bacteria', 'p__Bacteroidota', 'c__Rhodothermia', 'o__Rhodothermales', 'f__Bin80', 'g__Bin80', 's__'], 'COS36405_bin_7': ['d__Bacteria', 'p__Actinobacteriota', 'c__Actinobacteria', 'o__Actinomycetales', 'f__Micrococcaceae', 'g__Micrococcus', 's__Micrococcus luteus'], 'APA_bin_97': ['d__Bacteria', 'p__Deinococcota', 'c__Deinococci', 'o__Deinococcales', 'f__Trueperaceae', 'g__MPNL01', 's__MPNL01 sp002239005'], 'COS36387_bin_2': ['d__Bacteria', 'p__Chloroflexota', 'c__Anaerolineae', 'o__Caldilineales', 'f__Caldilineaceae', 'g__Bin5', 's__'], 'IRC_PAM_SB0664_bin_28': ['d__Bacteria', 'p__Poribacteria', 'c__WGA-4E', 'o__WGA-4E', 'f__WGA-3G', 'g__WGA-3G', 's__'], 'RHO1_bin_28': ['d__Bacteria', 'p__Latescibacterota', 'c__UBA2968', 'o__UBA2968', 'f__GCA-2709665', 'g__', 's__'], 'COS4_bin_25': ['d__Bacteria', 'p__Actinobacteriota', 'c__Acidimicrobiia', 'o__Microtrichales', 'f__TK06', 'g__', 's__'], 'IRC_PAM_SB0666_bin_22': ['d__Bacteria', 'p__Acidobacteriota', 'c__Thermoanaerobaculia', 'o__UBA5704', 'f__', 'g__', 's__'], 'seawater_22112_bin_10': ['d__Bacteria', 'p__Proteobacteria', 'c__Alphaproteobacteria', 'o__Sphingomonadales', 'f__Sphingomonadaceae', 'g__Erythrobacter_A', 's__Erythrobacter_A flavus'], 'IRC_PAM_SB0668_bin_14': ['d__Bacteria', 'p__Acidobacteriota', 'c__Bin61', 'o__Bin61', 'f__Bin61', 'g__Bin61', 's__'], 'RHO1_bin_70': ['d__Bacteria', 'p__Gemmatimonadota', 'c__Gemmatimonadetes', 'o__SG8-23', 'f__UBA6960', 'g__BD2-11', 's__'], 'COS36386_bin_31': ['d__Bacteria', 'p__Chloroflexota', 'c__Anaerolineae', 'o__SBR1031', 'f__A4b', 'g__UBA6055', 's__'], 'RHO2_bin_30': ['d__Bacteria', 'p__Latescibacterota', 'c__', 'o__', 'f__', 'g__', 's__'], 'seawater_22112_bin_9': ['d__Bacteria', 'p__Actinobacteriota', 'c__Acidimicrobiia', 'o__Microtrichales', 'f__Ilumatobacteraceae', 'g__Casp-actino5', 's__'], 'COS3_bin_15': ['d__Bacteria', 'p__Chloroflexota', 'c__Dehalococcoidia', 'o__Bin125', 'f__Bin125', 'g__TK10-74A', 's__'], 'IRC3_bin_25': ['d__Bacteria', 'p__Actinobacteriota', 'c__Acidimicrobiia', 'o__Microtrichales', 'f__UBA11606', 'g__', 's__'], 'seawater_42616_bin_2': ['d__Bacteria', 'p__Proteobacteria', 'c__Alphaproteobacteria', 'o__Puniceispirillales', 'f__Puniceispirillaceae', 'g__UBA8309', 's__UBA8309 sp001627655'], 'seawater_seasim_SB9154_S3_bin_4': ['d__Bacteria', 'p__Gemmatimonadota', 'c__Gemmatimonadetes', 'o__SG8-23', 'f__UBA6960', 'g__UBA2589', 's__'], 'COS36405_bin_10': ['d__Bacteria', 'p__Acidobacteriota', 'c__Thermoanaerobaculia', 'o__UBA5704', 'f__', 'g__', 's__'], 'RHO2_bin_62': ['d__Bacteria', 'p__Proteobacteria', 'c__Alphaproteobacteria', 'o__SP197', 'f__SP197', 'g__SP197', 's__'], 'COS4_bin_23': ['d__Bacteria', 'p__UBA8248', 'c__UBA8248', 'o__UBA8248', 'f__UBA8248', 'g__Bin107', 's__'], 'RHO1_bin_46': ['d__Bacteria', 'p__Bacteroidota', 'c__Rhodothermia', 'o__Rhodothermales', 'f__Bin80', 'g__Bin80', 's__'], 'IRC3_bin_17': ['d__Bacteria', 'p__Actinobacteriota', 'c__Acidimicrobiia', 'o__UBA5794', 'f__SZUA-232', 'g__', 's__'], 'Aquimarina_spAU58': ['d__Bacteria', 'p__Bacteroidota', 'c__Bacteroidia', 'o__Flavobacteriales', 'f__Flavobacteriaceae', 'g__Aquimarina', 's__Aquimarina sp900312745'], 'RHO2_bin_1': ['d__Bacteria', 'p__Proteobacteria', 'c__Alphaproteobacteria', 'o__Rhodobacterales', 'f__Rhodobacteraceae', 'g__Bin36', 's__'], 'RHO1_bin_13': ['d__Bacteria', 'p__Bacteroidota', 'c__Rhodothermia', 'o__Rhodothermales', 'f__Bin80', 'g__', 's__'], 'RHO3_bin_41': ['d__Bacteria', 'p__Proteobacteria', 'c__Gammaproteobacteria', 'o__UBA10353', 'f__LS-SOB', 'g__', 's__'], 'RHO2_bin_60': ['d__Bacteria', 'p__Chloroflexota', 'c__Dehalococcoidia', 'o__Bin125', 'f__Bin125', 'g__Bin125', 's__'], 'seawater_seasim_SB9155_S4_bin_12': ['d__Bacteria', 'p__Myxococcota', 'c__Polyangia', 'o__Polyangiales', 'f__', 'g__', 's__'], 'PER4_bin_1': ['d__Bacteria', 'p__Cyanobacteria', 'c__Cyanobacteriia', 'o__Synechococcales', 'f__Cyanobiaceae', 'g__Synechococcus_C', 's__Synechococcus_C sp002724845'], 'IRC_PAM_SB0666_bin_1': ['d__Bacteria', 'p__Chloroflexota', 'c__Dehalococcoidia', 'o__UBA3495', 'f__UBA3495', 'g__Bin87', 's__'], 'COS36406_bin_9': ['d__Bacteria', 'p__Acidobacteriota', 'c__Thermoanaerobaculia', 'o__UBA5704', 'f__', 'g__', 's__'], 'COS1_bin_2': ['d__Bacteria', 'p__Chloroflexota', 'c__Anaerolineae', 'o__Caldilineales', 'f__Caldilineaceae', 'g__Bin5', 's__'], 'IRC_PAM_SB0665_bin_1': ['d__Bacteria', 'p__Chloroflexota_B', 'c__UBA11872', 'o__UBA11872', 'f__', 'g__', 's__'], 'COS4_bin_12': ['d__Bacteria', 'p__Proteobacteria', 'c__Alphaproteobacteria', 'o__Rhodobacterales', 'f__Rhodobacteraceae', 'g__', 's__'], 'RHO3_bin_12': ['d__Bacteria', 'p__Acidobacteriota', 'c__Acidobacteriae', 'o__Bryobacterales', 'f__UBA6623', 'g__', 's__'], 'CLI2_bin_1': ['d__Bacteria', 'p__Proteobacteria', 'c__Alphaproteobacteria', 'o__Parvibaculales', 'f__RS24', 'g__', 's__'], 'seawater_bettina_36328_bin_8': ['d__Bacteria', 'p__Proteobacteria', 'c__Gammaproteobacteria', 'o__Xanthomonadales', 'f__SZUA-36', 'g__', 's__'], 'IRC4_bin_3': ['d__Bacteria', 'p__Dadabacteria', 'c__UBA1144', 'o__', 'f__', 'g__', 's__'], 'IRC_PAM_SB0661_bin_1': ['d__Bacteria', 'p__Acidobacteriota', 'c__Vicinamibacteria', 'o__Vicinamibacterales', 'f__UBA8438', 'g__', 's__'], 'GCA_003635195': ['d__Bacteria', 'p__Poribacteria', 'c__WGA-4E', 'o__WGA-4E', 'f__WGA-3G', 'g__WGA-3G', 's__'], 'STY3_bin_3': ['d__Bacteria', 'p__Nitrospirota', 'c__Nitrospiria', 'o__Nitrospirales', 'f__UBA8639', 'g__Bin75', 's__'], 'IRC_PAM_SB0667_bin_9': ['d__Bacteria', 'p__Nitrospirota', 'c__Nitrospiria', 'o__Nitrospirales', 'f__UBA8639', 'g__Bin75', 's__'], 'COS1_bin_10': ['d__Bacteria', 'p__Chloroflexota', 'c__Anaerolineae', 'o__SBR1031', 'f__A4b', 'g__GCA-2702065', 's__'], 'GCA_003635305': ['d__Bacteria', 'p__Poribacteria', 'c__WGA-4E', 'o__WGA-4E', 'f__WGA-3G', 'g__WGA-3G', 's__'], 'IRC3_bin_15': ['d__Bacteria', 'p__Chloroflexota', 'c__Dehalococcoidia', 'o__SAR202', 'f__UBA11138', 'g__Bin90', 's__'], 'seawater_seasim_SB9158_S7_bin_9': ['d__Bacteria', 'p__Proteobacteria', 'c__Alphaproteobacteria', 'o__Rhodospirillales_A', 'f__UBA3470', 'g__', 's__'], 'IRC_PAM_SB0666_bin_34': ['d__Bacteria', 'p__Gemmatimonadota', 'c__Gemmatimonadetes', 'o__SG8-23', 'f__UBA6960', 'g__Bin94', 's__'], 'COS4_bin_26': ['d__Bacteria', 'p__Proteobacteria', 'c__Gammaproteobacteria', 'o__GCA-2729495', 'f__GCA-2729495', 'g__', 's__'], 'APA_bin_74': ['d__Bacteria', 'p__Actinobacteriota', 'c__Acidimicrobiia', 'o__Microtrichales', 'f__Bin134', 'g__Bin134', 's__Bin134 sp002239105'], 'IRC4_bin_49': ['d__Bacteria', 'p__Gemmatimonadota', 'c__Gemmatimonadetes', 'o__SG8-23', 'f__UBA6960', 'g__Bin94', 's__'], 'CAR2_bin_10': ['d__Bacteria', 'p__Proteobacteria', 'c__Alphaproteobacteria', 'o__Rhodobacterales', 'f__Rhodobacteraceae', 'g__Aestuariivita', 's__'], 'seawater_bettina_36308_bin_18': ['d__Bacteria', 'p__Proteobacteria', 'c__Alphaproteobacteria', 'o__Puniceispirillales', 'f__Puniceispirillaceae', 'g__UBA685', 's__'], 'STY1_bin_7': ['d__Bacteria', 'p__Cyanobacteria', 'c__Cyanobacteriia', 'o__Synechococcales', 'f__Cyanobiaceae', 'g__Synechococcus_C', 's__Synechococcus_C sp002724845'], 'IRC_PAM_SB0668_bin_27': ['d__Bacteria', 'p__Chloroflexota', 'c__Dehalococcoidia', 'o__UBA2963', 'f__UBA2963', 'g__Bin16', 's__'], 'RHO2_bin_50': ['d__Bacteria', 'p__Gemmatimonadota', 'c__Gemmatimonadetes', 'o__SG8-23', 'f__UBA6960', 'g__BD2-11', 's__'], 'APA_bin_70': ['d__Bacteria', 'p__Proteobacteria', 'c__Alphaproteobacteria', 'o__Rhodobacterales', 'f__Rhodobacteraceae', 'g__Bin36', 's__Bin36 sp002238565'], 'COS36386_bin_17': ['d__Bacteria', 'p__Actinobacteriota', 'c__Acidimicrobiia', 'o__UBA5794', 'f__SZUA-232', 'g__', 's__'], 'GCF_002573675': ['d__Bacteria', 'p__Actinobacteriota', 'c__Actinobacteria', 'o__Mycobacteriales', 'f__Micromonosporaceae', 'g__Micromonospora', 's__Micromonospora sediminicola'], 'IRC_PAM_SB0668_bin_11': ['d__Bacteria', 'p__Proteobacteria', 'c__Gammaproteobacteria', 'o__Pseudomonadales', 'f__Pseudohongiellaceae', 'g__UBA9145', 's__'], 'APA_bin_52': ['d__Bacteria', 'p__Chloroflexota', 'c__Anaerolineae', 'o__Caldilineales', 'f__Bin34', 'g__Bin34', 's__Bin34 sp002238555'], 'RHO2_bin_27': ['d__Bacteria', 'p__Acidobacteriota', 'c__Acidobacteriae', 'o__Bryobacterales', 'f__UBA6623', 'g__', 's__'], 'CAR3_bin_13': ['d__Bacteria', 'p__Proteobacteria', 'c__Gammaproteobacteria', 'o__HK1', 'f__HK1', 'g__', 's__'], 'IRC3_bin_10': ['d__Bacteria', 'p__Chloroflexota', 'c__Anaerolineae', 'o__SBR1031', 'f__A4b', 'g__UBA6055', 's__'], 'RHO3_bin_6': ['d__Bacteria', 'p__Poribacteria', 'c__WGA-4E', 'o__GCA-2687025', 'f__GCA-2687025', 'g__', 's__'], 'IRC_PAM_SB0666_bin_15': ['d__Archaea', 'p__Crenarchaeota', 'c__Nitrososphaeria', 'o__Nitrososphaerales', 'f__Nitrosopumilaceae', 'g__Cenarchaeum', 's__'], 'RHO1_bin_33': ['d__Bacteria', 'p__Acidobacteriota', 'c__Thermoanaerobaculia', 'o__UBA5704', 'f__', 'g__', 's__'], 'GCF_001431305': ['d__Bacteria', 'p__Proteobacteria', 'c__Alphaproteobacteria', 'o__Rhizobiales', 'f__Stappiaceae', 'g__Pseudovibrio', 's__Pseudovibrio sp900143565'], 'RHO3_bin_5': ['d__Bacteria', 'p__Bacteroidota', 'c__Rhodothermia', 'o__Rhodothermales', 'f__Bin80', 'g__', 's__'], 'COS36405_bin_12': ['d__Bacteria', 'p__Proteobacteria', 'c__Alphaproteobacteria', 'o__Bin65', 'f__Bin65', 'g__Bin65', 's__'], 'RHO2_bin_3': ['d__Bacteria', 'p__Acidobacteriota', 'c__Thermoanaerobaculia', 'o__UBA5704', 'f__', 'g__', 's__'], 'IRC_PAM_SB0665_bin_27': ['d__Bacteria', 'p__Proteobacteria', 'c__Gammaproteobacteria', 'o__Pseudomonadales', 'f__HTCC2089', 'g__', 's__'], 'COS1_bin_7': ['d__Bacteria', 'p__Nitrospirota', 'c__Nitrospiria', 'o__Nitrospirales', 'f__UBA8639', 'g__Bin75', 's__'], 'COS36406_bin_2': ['d__Bacteria', 'p__Nitrospirota', 'c__Nitrospiria', 'o__Nitrospirales', 'f__UBA8639', 'g__Bin75', 's__'], 'RHO3_bin_51': ['d__Bacteria', 'p__Poribacteria', 'c__WGA-4E', 'o__WGA-4E', 'f__WGA-3G', 'g__', 's__'], 'IRC_PAM_SB0675_bin_14': ['d__Bacteria', 'p__Latescibacterota', 'c__', 'o__', 'f__', 'g__', 's__'], 'CAR2_bin_16': ['d__Bacteria', 'p__Proteobacteria', 'c__Alphaproteobacteria', 'o__Rhodobacterales', 'f__Rhodobacteraceae', 'g__Roseovarius', 's__'], 'COS4_bin_21': ['d__Bacteria', 'p__Cyanobacteria', 'c__Cyanobacteriia', 'o__Synechococcales', 'f__Cyanobiaceae', 'g__Synechococcus_C', 's__Synechococcus_C sp002724845'], 'COS36387_bin_7': ['d__Bacteria', 'p__Planctomycetota', 'c__Planctomycetes', 'o__Pirellulales', 'f__UBA1268', 'g__UBA1268', 's__UBA1268 sp002694955'], 'seawater_bettina_36309_bin_3': ['d__Bacteria', 'p__Proteobacteria', 'c__Alphaproteobacteria', 'o__Puniceispirillales', 'f__Puniceispirillaceae', 'g__UBA8309', 's__UBA8309 sp001627655'], 'IRC_PAM_SB0675_bin_20': ['d__Bacteria', 'p__Gemmatimonadota', 'c__Gemmatimonadetes', 'o__SG8-23', 'f__UBA6960', 'g__Bin94', 's__'], 'CAR1_bin_1': ['d__Bacteria', 'p__Proteobacteria', 'c__Gammaproteobacteria', 'o__Pseudomonadales', 'f__HTCC2089', 'g__UBA2168', 's__'], 'IRC_PAM_SB0661_bin_11': ['d__Bacteria', 'p__Latescibacterota', 'c__UBA2968', 'o__UBA2968', 'f__', 'g__', 's__'], 'GCF_900143565': ['d__Bacteria', 'p__Proteobacteria', 'c__Alphaproteobacteria', 'o__Rhizobiales', 'f__Stappiaceae', 'g__Pseudovibrio', 's__Pseudovibrio sp900143565'], 'seawater_bettina_36310_bin_4': ['d__Bacteria', 'p__Proteobacteria', 'c__Alphaproteobacteria', 'o__Puniceispirillales', 'f__UBA1172', 'g__', 's__'], 'CAR3_bin_17': ['d__Bacteria', 'p__Proteobacteria', 'c__Alphaproteobacteria', 'o__Rhodobacterales', 'f__Rhodobacteraceae', 'g__Roseovarius', 's__'], 'COS36405_bin_3': ['d__Bacteria', 'p__Acidobacteriota', 'c__Thermoanaerobaculia', 'o__UBA5704', 'f__', 'g__', 's__'], 'COS36404_bin_19': ['d__Bacteria', 'p__Acidobacteriota', 'c__Vicinamibacteria', 'o__Vicinamibacterales', 'f__UBA8438', 'g__', 's__'], 'GCF_900141785': ['d__Bacteria', 'p__Bacteroidota', 'c__Bacteroidia', 'o__Flavobacteriales', 'f__Flavobacteriaceae', 'g__Aquimarina', 's__Aquimarina spongiae'], 'APA_bin_41': ['d__Bacteria', 'p__Latescibacterota', 'c__', 'o__', 'f__', 'g__', 's__'], 'IRC_PAM_SB0662_bin_59': ['d__Bacteria', 'p__Proteobacteria', 'c__Gammaproteobacteria', 'o__Pseudomonadales', 'f__HTCC2089', 'g__', 's__'], 'COS3_bin_13': ['d__Bacteria', 'p__Acidobacteriota', 'c__Thermoanaerobaculia', 'o__UBA5704', 'f__', 'g__', 's__'], 'RHO3_bin_11': ['d__Bacteria', 'p__Proteobacteria', 'c__Alphaproteobacteria', 'o__Defluviicoccales', 'f__Defluviicoccaceae', 'g__Defluviicoccus', 's__'], 'APA_bin_69': ['d__Bacteria', 'p__Proteobacteria', 'c__Gammaproteobacteria', 'o__HK1', 'f__', 'g__', 's__'], 'COS2_bin_4': ['d__Bacteria', 'p__Chloroflexota_B', 'c__UBA11872', 'o__UBA11872', 'f__UBA11872', 'g__', 's__'], 'RHO2_bin_54': ['d__Bacteria', 'p__Gemmatimonadota', 'c__Gemmatimonadetes', 'o__SG8-23', 'f__UBA6960', 'g__Bin94', 's__'], 'COS4_bin_34': ['d__Bacteria', 'p__Actinobacteriota', 'c__Acidimicrobiia', 'o__Microtrichales', 'f__Bin134', 'g__', 's__'], 'CAR1_bin_10': ['d__Bacteria', 'p__Proteobacteria', 'c__Gammaproteobacteria', 'o__Pseudomonadales', 'f__Pseudohongiellaceae', 'g__UBA9145', 's__'], 'COS36404_bin_2': ['d__Bacteria', 'p__Acidobacteriota', 'c__Thermoanaerobaculia', 'o__UBA5704', 'f__', 'g__', 's__'], 'COS36405_bin_20': ['d__Bacteria', 'p__Binatota', 'c__Binatia', 'o__Bin18', 'f__Bin18', 'g__', 's__'], 'COS1_bin_11': ['d__Archaea', 'p__Crenarchaeota', 'c__Nitrososphaeria', 'o__Nitrososphaerales', 'f__Nitrosopumilaceae', 'g__', 's__'], 'STY2_bin_6': ['d__Bacteria', 'p__Proteobacteria', 'c__Gammaproteobacteria', 'o__UBA6729', 'f__', 'g__', 's__'], 'COS4_bin_6': ['d__Bacteria', 'p__Acidobacteriota', 'c__Bin61', 'o__Bin61', 'f__Bin61', 'g__Bin61', 's__'], 'IRC_PAM_SB0661_bin_22': ['d__Bacteria', 'p__Chloroflexota', 'c__Dehalococcoidia', 'o__UBA2991', 'f__UBA2991', 'g__UBA2991', 's__'], 'RHO3_bin_52': ['d__Bacteria', 'p__Chloroflexota', 'c__Anaerolineae', 'o__Caldilineales', 'f__Caldilineaceae', 'g__Bin5', 's__'], 'RHO2_bin_17': ['d__Bacteria', 'p__Poribacteria', 'c__WGA-4E', 'o__WGA-4E', 'f__WGA-3G', 'g__', 's__'], 'CAR1_bin_12': ['d__Bacteria', 'p__Proteobacteria', 'c__Alphaproteobacteria', 'o__Rhodobacterales', 'f__Rhodobacteraceae', 'g__Aestuariivita', 's__'], 'APA_bin_38': ['d__Bacteria', 'p__Poribacteria', 'c__WGA-4E', 'o__WGA-4E', 'f__WGA-3G', 'g__WGA-3G', 's__'], 'IRC_PAM_SB0668_bin_12': ['d__Bacteria', 'p__Gemmatimonadota', 'c__Gemmatimonadetes', 'o__', 'f__', 'g__', 's__'], 'IRC3_bin_26': ['d__Bacteria', 'p__Actinobacteriota', 'c__Acidimicrobiia', 'o__Microtrichales', 'f__UBA11606', 'g__', 's__'], 'RHO3_bin_4': ['d__Bacteria', 'p__Actinobacteriota', 'c__Acidimicrobiia', 'o__Microtrichales', 'f__UBA11606', 'g__UBA11606', 's__'], 'COS3_bin_3': ['d__Bacteria', 'p__Chloroflexota', 'c__Dehalococcoidia', 'o__Bin125', 'f__', 'g__', 's__'], 'COS36404_bin_5': ['d__Bacteria', 'p__Nitrospirota', 'c__Nitrospiria', 'o__Nitrospirales', 'f__UBA8639', 'g__Bin75', 's__'], 'COS4_bin_16': ['d__Bacteria', 'p__Proteobacteria', 'c__Gammaproteobacteria', 'o__Porisulfidales', 'f__Porisulfidaceae', 'g__Porisulfidus', 's__'], 'RHO3_bin_17': ['d__Bacteria', 'p__Bdellovibrionota', 'c__Bdellovibrionia', 'o__Bdellovibrionales', 'f__UBA1609', 'g__', 's__'], 'RHO1_bin_43': ['d__Bacteria', 'p__Proteobacteria', 'c__Gammaproteobacteria', 'o__Pseudomonadales', 'f__HTCC2089', 'g__', 's__'], 'IRC_PAM_SB0661_bin_33': ['d__Bacteria', 'p__Gemmatimonadota', 'c__Gemmatimonadetes', 'o__SG8-23', 'f__UBA6960', 'g__Bin94', 's__'], 'CAR4_bin_15': ['d__Bacteria', 'p__Proteobacteria', 'c__Gammaproteobacteria', 'o__UBA10353', 'f__LS-SOB', 'g__', 's__'], 'GCF_900143525': ['d__Bacteria', 'p__Proteobacteria', 'c__Alphaproteobacteria', 'o__Rhodobacterales', 'f__Rhodobacteraceae', 'g__Ruegeria', 's__Ruegeria sp900143525'], 'COS36406_bin_4': ['d__Bacteria', 'p__Acidobacteriota', 'c__Thermoanaerobaculia', 'o__UBA5704', 'f__', 'g__', 's__'], 'RHO2_bin_36': ['d__Bacteria', 'p__Chloroflexota', 'c__Dehalococcoidia', 'o__UBA3495', 'f__UBA3495', 'g__Bin22', 's__'], 'RHO3_bin_21': ['d__Bacteria', 'p__Actinobacteriota', 'c__Acidimicrobiia', 'o__UBA5794', 'f__SZUA-232', 'g__', 's__'], 'RHO1_bin_72': ['d__Bacteria', 'p__Proteobacteria', 'c__Gammaproteobacteria', 'o__Pseudomonadales', 'f__HTCC2089', 'g__UBA2168', 's__'], 'IRC4_bin_22': ['d__Bacteria', 'p__Chloroflexota', 'c__Dehalococcoidia', 'o__SAR202', 'f__UBA11138', 'g__Bin90', 's__'], 'seawater_seasim_SB9156_S5_bin_8': ['d__Bacteria', 'p__Proteobacteria', 'c__Alphaproteobacteria', 'o__UBA8366', 'f__GCA-2717185', 'g__GCA-2717185', 's__'], 'COS2_bin_7': ['d__Bacteria', 'p__Chloroflexota', 'c__Anaerolineae', 'o__SBR1031', 'f__A4b', 'g__GCA-2702065', 's__'], 'IRC_PAM_SB0661_bin_15': ['d__Bacteria', 'p__Chloroflexota', 'c__Dehalococcoidia', 'o__UBA3495', 'f__UBA3495', 'g__Bin87', 's__'], 'CAR3_bin_10': ['d__Bacteria', 'p__Bacteroidota', 'c__Rhodothermia', 'o__Rhodothermales', 'f__Bin80', 'g__Bin80', 's__'], 'COS2_bin_2': ['d__Bacteria', 'p__Actinobacteriota', 'c__Acidimicrobiia', 'o__Microtrichales', 'f__Bin134', 'g__Bin134', 's__'], 'STY1_bin_8': ['d__Bacteria', 'p__Myxococcota', 'c__UBA9160', 'o__UBA9160', 'f__UBA6930', 'g__', 's__'], 'COS36388_bin_1': ['d__Bacteria', 'p__Acidobacteriota', 'c__Thermoanaerobaculia', 'o__UBA5704', 'f__', 'g__', 's__'], 'IRC4_bin_12': ['d__Bacteria', 'p__Acidobacteriota', 'c__Thermoanaerobaculia', 'o__UBA5704', 'f__', 'g__', 's__'], 'COS36406_bin_7': ['d__Bacteria', 'p__Proteobacteria', 'c__Alphaproteobacteria', 'o__Bin65', 'f__Bin65', 'g__Bin65', 's__'], 'APA_bin_3': ['d__Bacteria', 'p__Proteobacteria', 'c__Alphaproteobacteria', 'o__Defluviicoccales', 'f__Defluviicoccaceae', 'g__Defluviicoccus', 's__Defluviicoccus sp002239065'], 'seawater_42618_bin_5': ['d__Bacteria', 'p__Proteobacteria', 'c__Alphaproteobacteria', 'o__Puniceispirillales', 'f__Puniceispirillaceae', 'g__UBA8309', 's__UBA8309 sp002686685'], 'CAR3_bin_3': ['d__Bacteria', 'p__Actinobacteriota', 'c__Acidimicrobiia', 'o__Microtrichales', 'f__UBA11606', 'g__', 's__'], 'IRC1_bin_7': ['d__Bacteria', 'p__Proteobacteria', 'c__Gammaproteobacteria', 'o__Pseudomonadales', 'f__HTCC2089', 'g__', 's__'], 'RHO1_bin_55': ['d__Bacteria', 'p__Gemmatimonadota', 'c__Gemmatimonadetes', 'o__SG8-23', 'f__UBA6960', 'g__Bin94', 's__'], 'IRC3_bin_8': ['d__Bacteria', 'p__Proteobacteria', 'c__Alphaproteobacteria', 'o__Rhodobacterales', 'f__Rhodobacteraceae', 'g__Silicimonas', 's__'], 'COS4_bin_8': ['d__Bacteria', 'p__Chloroflexota', 'c__Anaerolineae', 'o__SBR1031', 'f__A4b', 'g__GCA-2702065', 's__'], 'STY1_bin_2': ['d__Bacteria', 'p__Proteobacteria', 'c__Gammaproteobacteria', 'o__UBA10353', 'f__LS-SOB', 'g__', 's__'], 'CAR2_bin_19': ['d__Bacteria', 'p__Actinobacteriota', 'c__Acidimicrobiia', 'o__Microtrichales', 'f__Bin134', 'g__Bin134', 's__'], 'CAR1_bin_2': ['d__Bacteria', 'p__Proteobacteria', 'c__Alphaproteobacteria', 'o__Rhodobacterales', 'f__Rhodobacteraceae', 'g__Bin36', 's__'], 'seawater_42617_bin_1': ['d__Bacteria', 'p__Proteobacteria', 'c__Alphaproteobacteria', 'o__Sphingomonadales', 'f__Sphingomonadaceae', 'g__Erythrobacter_A', 's__Erythrobacter_A sp002895025'], 'RHO3_bin_84': ['d__Bacteria', 'p__Spirochaetota', 'c__Spirochaetia', 'o__Spirochaetales', 'f__RBG-16-67-19', 'g__', 's__'], 'IRC_PAM_SB0665_bin_9': ['d__Bacteria', 'p__Acidobacteriota', 'c__Vicinamibacteria', 'o__Vicinamibacterales', 'f__UBA8438', 'g__', 's__'], 'IRC_PAM_SB0664_bin_7': ['d__Bacteria', 'p__Bacteroidota', 'c__Rhodothermia', 'o__Rhodothermales', 'f__Bin80', 'g__Bin80', 's__'], 'seawater_bettina_36327_bin_6': ['d__Bacteria', 'p__Bacteroidota', 'c__Bacteroidia', 'o__Flavobacteriales', 'f__Flavobacteriaceae', 'g__UBA11891', 's__'], 'IRC_PAM_SB0675_bin_1': ['d__Bacteria', 'p__Proteobacteria', 'c__Gammaproteobacteria', 'o__Pseudomonadales', 'f__Pseudohongiellaceae', 'g__UBA9145', 's__'], 'IRC3_bin_20': ['d__Bacteria', 'p__Cyanobacteria', 'c__Cyanobacteriia', 'o__Synechococcales', 'f__Cyanobiaceae', 'g__Synechococcus_B', 's__'], 'IRC4_bin_24': ['d__Bacteria', 'p__Actinobacteriota', 'c__Acidimicrobiia', 'o__UBA5794', 'f__Bin76', 'g__Bin76', 's__'], 'RHO1_bin_21': ['d__Bacteria', 'p__Actinobacteriota', 'c__Acidimicrobiia', 'o__UBA5794', 'f__Bin76', 'g__Bin76', 's__'], 'COS4_bin_49': ['d__Bacteria', 'p__Acidobacteriota', 'c__UBA6911', 'o__', 'f__', 'g__', 's__'], 'GCA_000200715': ['d__Archaea', 'p__Crenarchaeota', 'c__Nitrososphaeria', 'o__Nitrososphaerales', 'f__Nitrosopumilaceae', 'g__Cenarchaeum', 's__Cenarchaeum symbiosum'], 'GCA_001007665': ['d__Bacteria', 'p__Cyanobacteria', 'c__Cyanobacteriia', 'o__Synechococcales', 'f__Cyanobiaceae', 'g__Synechococcus_B', 's__Synechococcus_B spongiarum_B'], 'CAR2_bin_2': ['d__Bacteria', 'p__Proteobacteria', 'c__Alphaproteobacteria', 'o__Rhodobacterales', 'f__Rhodobacteraceae', 'g__Bin36', 's__'], 'IRC3_bin_14': ['d__Bacteria', 'p__Actinobacteriota', 'c__Acidimicrobiia', 'o__UBA5794', 'f__Bin76', 'g__Bin76', 's__'], 'COS36386_bin_12': ['d__Bacteria', 'p__Acidobacteriota', 'c__Vicinamibacteria', 'o__Vicinamibacterales', 'f__UBA8438', 'g__', 's__'], 'IRC4_bin_13': ['d__Bacteria', 'p__Gemmatimonadota', 'c__Gemmatimonadetes', 'o__', 'f__', 'g__', 's__'], 'seawater_seasim_SB9152_S1_bin_2': ['d__Bacteria', 'p__Myxococcota', 'c__Polyangia', 'o__Polyangiales', 'f__', 'g__', 's__'], 'IRC1_bin_37': ['d__Bacteria', 'p__Actinobacteriota', 'c__Acidimicrobiia', 'o__Microtrichales', 'f__Bin134', 'g__Bin134', 's__'], 'COS36386_bin_35': ['d__Bacteria', 'p__UBA8248', 'c__UBA8248', 'o__UBA8248', 'f__UBA8248', 'g__Bin107', 's__'], 'COS36406_bin_6': ['d__Bacteria', 'p__Planctomycetota', 'c__Planctomycetes', 'o__Pirellulales', 'f__UBA1268', 'g__UBA1268', 's__UBA1268 sp002694955'], 'COS1_bin_17': ['d__Bacteria', 'p__Proteobacteria', 'c__Gammaproteobacteria', 'o__GCA-2729495', 'f__GCA-2729495', 'g__', 's__'], 'COS4_bin_17': ['d__Archaea', 'p__Crenarchaeota', 'c__Nitrososphaeria', 'o__Nitrososphaerales', 'f__Nitrosopumilaceae', 'g__', 's__'], 'COS36387_bin_22': ['d__Bacteria', 'p__Chloroflexota', 'c__Anaerolineae', 'o__SBR1031', 'f__', 'g__', 's__'], 'RHO2_bin_23': ['d__Bacteria', 'p__Proteobacteria', 'c__Gammaproteobacteria', 'o__UBA10353', 'f__LS-SOB', 'g__', 's__'], 'COS2_bin_3': ['d__Bacteria', 'p__Nitrospirota', 'c__Nitrospiria', 'o__Nitrospirales', 'f__UBA8639', 'g__Bin75', 's__'], 'RHO1_bin_85': ['d__Bacteria', 'p__Proteobacteria', 'c__Alphaproteobacteria', 'o__UBA7887', 'f__', 'g__', 's__'], 'SPOO_karimiCosta_FZLR01': ['d__Bacteria', 'p__Proteobacteria', 'c__Alphaproteobacteria', 'o__Bin65', 'f__Bin65', 'g__Bin65', 's__Bin65 sp900197615'], 'IRC_PAM_SB0662_bin_5': ['d__Bacteria', 'p__Chloroflexota', 'c__Dehalococcoidia', 'o__UBA2991', 'f__UBA2991', 'g__UBA2991', 's__'], 'COS4_bin_40': ['d__Bacteria', 'p__Chloroflexota_B', 'c__UBA11872', 'o__UBA11872', 'f__UBA11872', 'g__', 's__'], 'GCA_001007635': ['d__Bacteria', 'p__Cyanobacteria', 'c__Cyanobacteriia', 'o__Synechococcales', 'f__Cyanobiaceae', 'g__Synechococcus_B', 's__Synechococcus_B spongiarum_E'], 'IRC2_bin_13': ['d__Bacteria', 'p__Chloroflexota', 'c__Dehalococcoidia', 'o__UBA2991', 'f__UBA2991', 'g__UBA2991', 's__'], 'CAR4_bin_9': ['d__Bacteria', 'p__Proteobacteria', 'c__Gammaproteobacteria', 'o__UBA10353', 'f__LS-SOB', 'g__', 's__'], 'seawater_22112_bin_6': ['d__Bacteria', 'p__Proteobacteria', 'c__Gammaproteobacteria', 'o__Pseudomonadales', 'f__Halieaceae', 'g__Luminiphilus', 's__'], 'seawater_seasim_SB9154_S3_bin_3': ['d__Bacteria', 'p__Myxococcota', 'c__Polyangia', 'o__Polyangiales', 'f__', 'g__', 's__'], 'GCA_003635255': ['d__Bacteria', 'p__Poribacteria', 'c__WGA-4E', 'o__WGA-4E', 'f__WGA-3G', 'g__WGA-3G', 's__'], 'RHO3_bin_33': ['d__Bacteria', 'p__Bdellovibrionota', 'c__Bdellovibrionia', 'o__Bdellovibrionales', 'f__UBA1609', 'g__', 's__'], 'IRC_PAM_SB0675_bin_18': ['d__Bacteria', 'p__Chloroflexota', 'c__Anaerolineae', 'o__SBR1031', 'f__A4b', 'g__UBA6055', 's__'], 'IRC1_bin_12': ['d__Bacteria', 'p__Poribacteria', 'c__WGA-4E', 'o__WGA-4E', 'f__WGA-3G', 'g__WGA-3G', 's__'], 'RHO2_bin_13': ['d__Bacteria', 'p__Poribacteria', 'c__WGA-4E', 'o__WGA-4E', 'f__WGA-3G', 'g__WGA-3G', 's__'], 'IRC_PAM_SB0664_bin_9': ['d__Bacteria', 'p__Actinobacteriota', 'c__Acidimicrobiia', 'o__Microtrichales', 'f__TK06', 'g__', 's__'], 'APA_bin_62': ['d__Bacteria', 'p__Spirochaetota', 'c__Spirochaetia', 'o__Spirochaetales', 'f__RBG-16-67-19', 'g__Bin103', 's__Bin103 sp002238925'], 'RHO1_bin_60': ['d__Bacteria', 'p__Proteobacteria', 'c__Alphaproteobacteria', 'o__UBA828', 'f__', 'g__', 's__'], 'CAR2_bin_15': ['d__Bacteria', 'p__Bacteroidota', 'c__Bacteroidia', 'o__Flavobacteriales', 'f__Flavobacteriaceae', 'g__Bin25', 's__'], 'RHO1_bin_19': ['d__Bacteria', 'p__Acidobacteriota', 'c__Acidobacteriae', 'o__Bryobacterales', 'f__UBA6623', 'g__', 's__'], 'CAR2_bin_13': ['d__Bacteria', 'p__Actinobacteriota', 'c__Acidimicrobiia', 'o__Microtrichales', 'f__UBA11606', 'g__', 's__'], 'CAR2_bin_22': ['d__Bacteria', 'p__Proteobacteria', 'c__Gammaproteobacteria', 'o__Pseudomonadales', 'f__Pseudohongiellaceae', 'g__UBA9145', 's__'], 'IRC_PAM_SB0665_bin_19': ['d__Bacteria', 'p__Acidobacteriota', 'c__Thermoanaerobaculia', 'o__UBA5704', 'f__', 'g__', 's__'], 'COS2_bin_18': ['d__Bacteria', 'p__Chloroflexota', 'c__Anaerolineae', 'o__Caldilineales', 'f__Caldilineaceae', 'g__Bin5', 's__'], 'COS36386_bin_8': ['d__Bacteria', 'p__Proteobacteria', 'c__Gammaproteobacteria', 'o__Coxiellales', 'f__Coxiellaceae', 'g__UBA9148', 's__'], 'COS36404_bin_6': ['d__Bacteria', 'p__Proteobacteria', 'c__Alphaproteobacteria', 'o__UBA828', 'f__UBA828', 'g__', 's__'], 'CAR3_bin_1': ['d__Bacteria', 'p__Proteobacteria', 'c__Alphaproteobacteria', 'o__Rhodobacterales', 'f__Rhodobacteraceae', 'g__Bin36', 's__'], 'RHO2_bin_15': ['d__Bacteria', 'p__Latescibacterota', 'c__UBA2968', 'o__UBA2968', 'f__', 'g__', 's__'], 'IRC_PAM_SB0667_bin_13': ['d__Archaea', 'p__Crenarchaeota', 'c__Nitrososphaeria', 'o__Nitrososphaerales', 'f__Nitrosopumilaceae', 'g__Cenarchaeum', 's__'], 'IRC_PAM_SB0665_bin_20': ['d__Bacteria', 'p__Latescibacterota', 'c__UBA2968', 'o__UBA2968', 'f__', 'g__', 's__'], 'COS36387_bin_16': ['d__Bacteria', 'p__Chloroflexota', 'c__Anaerolineae', 'o__SBR1031', 'f__A4b', 'g__UBA6055', 's__'], 'APA_bin_6': ['d__Bacteria', 'p__Chloroflexota', 'c__Dehalococcoidia', 'o__UBA2963', 'f__UBA2963', 'g__Bin16', 's__Bin16 sp002238425'], 'IRC_PAM_SB0670_bin_1': ['d__Bacteria', 'p__Actinobacteriota', 'c__Acidimicrobiia', 'o__Microtrichales', 'f__UBA11606', 'g__UBA11606', 's__'], 'COS36386_bin_13': ['d__Bacteria', 'p__Chloroflexota_B', 'c__UBA11872', 'o__UBA11872', 'f__UBA11872', 'g__', 's__'], 'seawater_22112_bin_25': ['d__Bacteria', 'p__Verrucomicrobiota', 'c__Verrucomicrobiae', 'o__Verrucomicrobiales', 'f__DEV007', 'g__EC70', 's__EC70 sp002696885'], 'IRC_PAM_SB0668_bin_13': ['d__Bacteria', 'p__Cyanobacteria', 'c__Cyanobacteriia', 'o__Synechococcales', 'f__Cyanobiaceae', 'g__Synechococcus_B', 's__'], 'COS1_bin_21': ['d__Bacteria', 'p__Chloroflexota_B', 'c__UBA11872', 'o__UBA11872', 'f__', 'g__', 's__'], 'APA_bin_102': ['d__Bacteria', 'p__Acidobacteriota', 'c__Vicinamibacteria', 'o__Vicinamibacterales', 'f__UBA8438', 'g__', 's__'], 'RHO2_bin_42': ['d__Bacteria', 'p__Chloroflexota', 'c__Dehalococcoidia', 'o__UBA2985', 'f__UBA2985', 'g__', 's__'], 'APA_bin_42': ['d__Bacteria', 'p__Chloroflexota', 'c__Anaerolineae', 'o__SBR1031', 'f__A4b', 'g__UBA6055', 's__'], 'STY1_bin_5': ['d__Archaea', 'p__Crenarchaeota', 'c__Nitrososphaeria', 'o__Nitrososphaerales', 'f__Nitrosopumilaceae', 'g__Cenarchaeum', 's__'], 'IRC1_bin_6': ['d__Bacteria', 'p__Bacteroidota', 'c__Rhodothermia', 'o__Rhodothermales', 'f__Bin80', 'g__Bin80', 's__'], 'seawater_22112_bin_17': ['d__Bacteria', 'p__Planctomycetota', 'c__Planctomycetes', 'o__Pirellulales', 'f__UBA1268', 'g__UBA1268', 's__UBA1268 sp002694955'], 'seawater_bettina_36327_bin_7': ['d__Bacteria', 'p__Bacteroidota', 'c__Bacteroidia', 'o__Flavobacteriales', 'f__Flavobacteriaceae', 'g__UBA11891', 's__'], 'CAR4_bin_18': ['d__Bacteria', 'p__Proteobacteria', 'c__Alphaproteobacteria', 'o__Rhodobacterales', 'f__Rhodobacteraceae', 'g__Roseovarius', 's__'], 'RHO1_bin_82': ['d__Bacteria', 'p__Proteobacteria', 'c__Alphaproteobacteria', 'o__Bin95', 'f__Bin95', 'g__Bin95', 's__'], 'RHO3_bin_73': ['d__Bacteria', 'p__Gemmatimonadota', 'c__Gemmatimonadetes', 'o__SG8-23', 'f__UBA6960', 'g__Bin94', 's__'], 'COS4_bin_22': ['d__Bacteria', 'p__Chloroflexota_B', 'c__UBA11872', 'o__UBA11872', 'f__', 'g__', 's__'], 'COS1_bin_15': ['d__Bacteria', 'p__Proteobacteria', 'c__Alphaproteobacteria', 'o__UBA7887', 'f__GCA-2721365', 'g__', 's__'], 'RHO2_bin_25': ['d__Archaea', 'p__Crenarchaeota', 'c__Nitrososphaeria', 'o__Nitrososphaerales', 'f__Nitrosopumilaceae', 'g__', 's__'], 'IRC_PAM_SB0666_bin_10': ['d__Bacteria', 'p__Actinobacteriota', 'c__Acidimicrobiia', 'o__Microtrichales', 'f__Bin134', 'g__Bin134', 's__'], 'IRC_PAM_SB0665_bin_10': ['d__Bacteria', 'p__Proteobacteria', 'c__Alphaproteobacteria', 'o__Rhodobacterales', 'f__Rhodobacteraceae', 'g__Silicimonas', 's__'], 'IRC1_bin_38': ['d__Bacteria', 'p__Actinobacteriota', 'c__Acidimicrobiia', 'o__Microtrichales', 'f__Bin134', 'g__Bin134', 's__'], 'COS36388_bin_18': ['d__Bacteria', 'p__Actinobacteriota', 'c__Acidimicrobiia', 'o__Microtrichales', 'f__TK06', 'g__', 's__'], 'RHO3_bin_40': ['d__Bacteria', 'p__Acidobacteriota', 'c__Acidobacteriae', 'o__Bryobacterales', 'f__UBA6623', 'g__', 's__'], 'COS36386_bin_5': ['d__Bacteria', 'p__Acidobacteriota', 'c__Thermoanaerobaculia', 'o__UBA5704', 'f__', 'g__', 's__'], 'IRC_PAM_SB0664_bin_24': ['d__Bacteria', 'p__Latescibacterota', 'c__UBA2968', 'o__UBA2968', 'f__', 'g__', 's__'], 'IRC_PAM_SB0675_bin_10': ['d__Bacteria', 'p__Proteobacteria', 'c__Gammaproteobacteria', 'o__UBA10353', 'f__LS-SOB', 'g__', 's__'], 'COS3_bin_9': ['d__Bacteria', 'p__Chloroflexota', 'c__Anaerolineae', 'o__Caldilineales', 'f__Caldilineaceae', 'g__Bin5', 's__'], 'RHO2_bin_59': ['d__Bacteria', 'p__Chloroflexota', 'c__Dehalococcoidia', 'o__Bin125', 'f__Bin125', 'g__Bin125', 's__'], 'seawater_bettina_36308_bin_3': ['d__Bacteria', 'p__Proteobacteria', 'c__Alphaproteobacteria', 'o__Rhodobacterales', 'f__Rhodobacteraceae', 'g__HIMB11', 's__HIMB11 sp001510135'], 'CLI1_bin_1': ['d__Bacteria', 'p__Dadabacteria', 'c__UBA1144', 'o__', 'f__', 'g__', 's__'], 'seawater_bettina_36327_bin_3': ['d__Bacteria', 'p__Proteobacteria', 'c__Alphaproteobacteria', 'o__Puniceispirillales', 'f__Puniceispirillaceae', 'g__UBA685', 's__'], 'IRC_PAM_SB0662_bin_49': ['d__Bacteria', 'p__Poribacteria', 'c__WGA-4E', 'o__WGA-4E', 'f__WGA-3G', 'g__WGA-3G', 's__'], 'CHO1_bin_1': ['d__Bacteria', 'p__Proteobacteria', 'c__Alphaproteobacteria', 'o__Rhodobacterales', 'f__Rhodobacteraceae', 'g__Ruegeria', 's__'], 'IRC_PAM_SB0661_bin_21': ['d__Bacteria', 'p__Chloroflexota', 'c__Anaerolineae', 'o__SBR1031', 'f__A4b', 'g__UBA6055', 's__'], 'IRC1_bin_31': ['d__Bacteria', 'p__Chloroflexota', 'c__Dehalococcoidia', 'o__UBA2991', 'f__UBA2991', 'g__UBA2991', 's__'], 'IRC_PAM_SB0661_bin_29': ['d__Bacteria', 'p__Poribacteria', 'c__WGA-4E', 'o__WGA-4E', 'f__WGA-3G', 'g__WGA-3G', 's__'], 'COS4_bin_36': ['d__Bacteria', 'p__Chloroflexota', 'c__Anaerolineae', 'o__SBR1031', 'f__', 'g__', 's__'], 'CAR4_bin_6': ['d__Bacteria', 'p__Cyanobacteria', 'c__Cyanobacteriia', 'o__Synechococcales', 'f__Cyanobiaceae', 'g__Synechococcus_B', 's__'], 'IRC1_bin_25': ['d__Bacteria', 'p__Chloroflexota', 'c__Dehalococcoidia', 'o__UBA2979', 'f__', 'g__', 's__'], 'IRC4_bin_41': ['d__Bacteria', 'p__Proteobacteria', 'c__Gammaproteobacteria', 'o__Pseudomonadales', 'f__HTCC2089', 'g__', 's__'], 'APA_bin_19': ['d__Bacteria', 'p__Acidobacteriota', 'c__Bin61', 'o__Bin61', 'f__Bin61', 'g__Bin61', 's__Bin61 sp002238705'], 'IRC_PAM_SB0675_bin_2': ['d__Bacteria', 'p__Actinobacteriota', 'c__Acidimicrobiia', 'o__Microtrichales', 'f__Bin134', 'g__Bin134', 's__'], 'APA_bin_72': ['d__Bacteria', 'p__Chloroflexota_B', 'c__UBA11872', 'o__', 'f__', 'g__', 's__'], 'SPOO_karimiCosta_FZLQ01': ['d__Bacteria', 'p__Proteobacteria', 'c__Alphaproteobacteria', 'o__Parvibaculales', 'f__RS24', 'g__UBA8337', 's__UBA8337 sp900197605'], 'RHO3_bin_54': ['d__Bacteria', 'p__Chloroflexota', 'c__Dehalococcoidia', 'o__UBA3495', 'f__UBA3495', 'g__Bin87', 's__'], 'IRC_PAM_SB0675_bin_29': ['d__Bacteria', 'p__Chloroflexota', 'c__Anaerolineae', 'o__Caldilineales', 'f__Caldilineaceae', 'g__Bin5', 's__'], 'IRC_PAM_SB0662_bin_11': ['d__Bacteria', 'p__Gemmatimonadota', 'c__Gemmatimonadetes', 'o__SG8-23', 'f__UBA6960', 'g__BD2-11', 's__'], 'IRC_PAM_SB0675_bin_23': ['d__Bacteria', 'p__Nitrospirota', 'c__Nitrospiria', 'o__Nitrospirales', 'f__UBA8639', 'g__Bin75', 's__'], 'CAR3_bin_18': ['d__Bacteria', 'p__Proteobacteria', 'c__Alphaproteobacteria', 'o__Rhodobacterales', 'f__Rhodobacteraceae', 'g__Aestuariivita', 's__'], 'COS36388_bin_9': ['d__Bacteria', 'p__Acidobacteriota', 'c__Thermoanaerobaculia', 'o__UBA5704', 'f__', 'g__', 's__'], 'seawater_22112_bin_29': ['d__Bacteria', 'p__Proteobacteria', 'c__Alphaproteobacteria', 'o__Rhodobacterales', 'f__Rhodobacteraceae', 'g__HIMB11', 's__HIMB11 sp001627375'], 'RHO1_bin_6': ['d__Bacteria', 'p__Proteobacteria', 'c__Alphaproteobacteria', 'o__Rhodobacterales', 'f__Rhodobacteraceae', 'g__', 's__'], 'IRC4_bin_38': ['d__Bacteria', 'p__Actinobacteriota', 'c__Acidimicrobiia', 'o__Microtrichales', 'f__Bin134', 'g__', 's__'], 'RHO2_bin_7': ['d__Bacteria', 'p__Poribacteria', 'c__WGA-4E', 'o__WGA-4E', 'f__', 'g__', 's__'], 'COS3_bin_5': ['d__Bacteria', 'p__Actinobacteriota', 'c__Acidimicrobiia', 'o__Microtrichales', 'f__TK06', 'g__', 's__'], 'COS36386_bin_19': ['d__Archaea', 'p__Crenarchaeota', 'c__Nitrososphaeria', 'o__Nitrososphaerales', 'f__Nitrosopumilaceae', 'g__', 's__'], 'IRC4_bin_4': ['d__Bacteria', 'p__Bacteroidota', 'c__Rhodothermia', 'o__Rhodothermales', 'f__Bin80', 'g__Bin80', 's__'], 'COS36387_bin_1': ['d__Bacteria', 'p__Nitrospirota', 'c__Nitrospiria', 'o__Nitrospirales', 'f__UBA8639', 'g__Bin75', 's__'], 'RHO1_bin_26': ['d__Bacteria', 'p__Chloroflexota', 'c__Anaerolineae', 'o__SBR1031', 'f__A4b', 'g__UBA6055', 's__'], 'RHO3_bin_74': ['d__Bacteria', 'p__Chloroflexota_B', 'c__UBA11872', 'o__UBA11872', 'f__UBA11872', 'g__', 's__'], 'GCF_003676335': ['d__Bacteria', 'p__Proteobacteria', 'c__Gammaproteobacteria', 'o__Enterobacterales', 'f__Shewanellaceae', 'g__Shewanella', 's__'], 'COS36405_bin_2': ['d__Bacteria', 'p__Planctomycetota', 'c__Planctomycetes', 'o__Pirellulales', 'f__UBA1268', 'g__UBA1268', 's__UBA1268 sp002694955'], 'RHO1_bin_22': ['d__Bacteria', 'p__Actinobacteriota', 'c__Acidimicrobiia', 'o__UBA5794', 'f__Bin76', 'g__Bin76', 's__'], 'COS36406_bin_13': ['d__Archaea', 'p__Crenarchaeota', 'c__Nitrososphaeria', 'o__Nitrososphaerales', 'f__Nitrosopumilaceae', 'g__', 's__'], 'IRC_PAM_SB0664_bin_10': ['d__Bacteria', 'p__Gemmatimonadota', 'c__Gemmatimonadetes', 'o__SG8-23', 'f__UBA6960', 'g__Bin94', 's__'], 'IRC_PAM_SB0677_bin_1': ['d__Bacteria', 'p__Proteobacteria', 'c__Gammaproteobacteria', 'o__Pseudomonadales', 'f__Pseudohongiellaceae', 'g__UBA9145', 's__'], 'RHO1_bin_62': ['d__Bacteria', 'p__Poribacteria', 'c__WGA-4E', 'o__WGA-4E', 'f__WGA-3G', 'g__WGA-3G', 's__'], 'RHO1_bin_14': ['d__Bacteria', 'p__Chloroflexota', 'c__Anaerolineae', 'o__SBR1031', 'f__A4b', 'g__UBA6055', 's__'], 'RHO1_bin_24': ['d__Bacteria', 'p__Nitrospirota', 'c__Nitrospiria', 'o__Nitrospirales', 'f__UBA8639', 'g__Bin75', 's__'], 'CAR1_bin_9': ['d__Bacteria', 'p__Actinobacteriota', 'c__Acidimicrobiia', 'o__Microtrichales', 'f__UBA11606', 'g__', 's__'], 'IRC2_bin_4': ['d__Bacteria', 'p__Actinobacteriota', 'c__Acidimicrobiia', 'o__Microtrichales', 'f__TK06', 'g__', 's__'], 'COS4_bin_15': ['d__Bacteria', 'p__Proteobacteria', 'c__Gammaproteobacteria', 'o__Pseudomonadales', 'f__HTCC2089', 'g__Bin55', 's__'], 'IRC3_bin_3': ['d__Bacteria', 'p__Bacteroidota', 'c__Rhodothermia', 'o__Rhodothermales', 'f__Bin80', 'g__Bin80', 's__'], 'COS4_bin_29': ['d__Bacteria', 'p__Proteobacteria', 'c__Gammaproteobacteria', 'o__Pseudomonadales', 'f__HTCC2089', 'g__', 's__'], 'GCF_900109375': ['d__Bacteria', 'p__Bacteroidota', 'c__Bacteroidia', 'o__Flavobacteriales', 'f__Flavobacteriaceae', 'g__Aquimarina', 's__Aquimarina amphilecti'], 'IRC3_bin_4': ['d__Bacteria', 'p__Acidobacteriota', 'c__Thermoanaerobaculia', 'o__UBA5704', 'f__', 'g__', 's__'], 'IRC1_bin_17': ['d__Bacteria', 'p__Proteobacteria', 'c__Alphaproteobacteria', 'o__UBA7887', 'f__GCA-2721365', 'g__', 's__'], 'APA_bin_10': ['d__Bacteria', 'p__Actinobacteriota', 'c__Acidimicrobiia', 'o__UBA5794', 'f__Bin76', 'g__Bin76', 's__Bin76 sp002238785'], 'RHO1_bin_2': ['d__Bacteria', 'p__Proteobacteria', 'c__Alphaproteobacteria', 'o__Rhodobacterales', 'f__Rhodobacteraceae', 'g__Bin36', 's__'], 'COS3_bin_17': ['d__Bacteria', 'p__Chloroflexota_B', 'c__UBA11872', 'o__UBA11872', 'f__UBA11872', 'g__', 's__'], 'RHO3_bin_69': ['d__Bacteria', 'p__Poribacteria', 'c__WGA-4E', 'o__WGA-4E', 'f__WGA-3G', 'g__WGA-3G', 's__'], 'RHO3_bin_14': ['d__Bacteria', 'p__Proteobacteria', 'c__Gammaproteobacteria', 'o__Porisulfidales', 'f__Porisulfidaceae', 'g__Porisulfidus', 's__'], 'seawater_seasim_SB9153_S2_bin_4': ['d__Bacteria', 'p__Myxococcota', 'c__Polyangia', 'o__Polyangiales', 'f__', 'g__', 's__'], 'CAR2_bin_20': ['d__Bacteria', 'p__Dadabacteria', 'c__UBA1144', 'o__', 'f__', 'g__', 's__'], 'APA_bin_71': ['d__Bacteria', 'p__Proteobacteria', 'c__Alphaproteobacteria', 'o__Rhodobacterales', 'f__Rhodobacteraceae', 'g__Bin36', 's__Bin36 sp002239085'], 'COS36404_bin_8': ['d__Bacteria', 'p__Actinobacteriota', 'c__Acidimicrobiia', 'o__UBA5794', 'f__SZUA-232', 'g__', 's__'], 'RHO1_bin_64': ['d__Bacteria', 'p__Actinobacteriota', 'c__Acidimicrobiia', 'o__UBA5794', 'f__SZUA-232', 'g__', 's__'], 'COS2_bin_17': ['d__Bacteria', 'p__Chloroflexota', 'c__Anaerolineae', 'o__Caldilineales', 'f__Caldilineaceae', 'g__Bin5', 's__'], 'RHO1_bin_51': ['d__Bacteria', 'p__Gemmatimonadota', 'c__Gemmatimonadetes', 'o__SG8-23', 'f__UBA6960', 'g__Bin94', 's__'], 'GCA_002007405': ['d__Bacteria', 'p__Proteobacteria', 'c__Gammaproteobacteria', 'o__Thiomicrospirales', 'f__Thioglobaceae', 'g__Gsub', 's__Gsub sp002007405'], 'RHO2_bin_49': ['d__Bacteria', 'p__Gemmatimonadota', 'c__Gemmatimonadetes', 'o__', 'f__', 'g__', 's__'], 'COS36405_bin_14': ['d__Bacteria', 'p__Chloroflexota', 'c__Anaerolineae', 'o__Caldilineales', 'f__Caldilineaceae', 'g__Bin5', 's__'], 'RHO1_bin_12': ['d__Bacteria', 'p__Acidobacteriota', 'c__UBA6911', 'o__', 'f__', 'g__', 's__'], 'seawater_bettina_36328_bin_2': ['d__Bacteria', 'p__Proteobacteria', 'c__Alphaproteobacteria', 'o__Puniceispirillales', 'f__Puniceispirillaceae', 'g__UBA8309', 's__UBA8309 sp002695585'], 'COS4_bin_47': ['d__Bacteria', 'p__Latescibacterota', 'c__UBA2968', 'o__UBA2968', 'f__GCA-2709665', 'g__', 's__'], 'IRC1_bin_21': ['d__Bacteria', 'p__Chloroflexota', 'c__Dehalococcoidia', 'o__Bin125', 'f__Bin125', 'g__Bin125', 's__'], 'IRC1_bin_22': ['d__Bacteria', 'p__Gemmatimonadota', 'c__Gemmatimonadetes', 'o__SG8-23', 'f__UBA6960', 'g__Bin94', 's__'], 'IRC_PAM_SB0662_bin_28': ['d__Bacteria', 'p__Chloroflexota', 'c__Dehalococcoidia', 'o__UBA3495', 'f__UBA3495', 'g__Bin22', 's__'], 'GCF_900143545': ['d__Bacteria', 'p__Proteobacteria', 'c__Alphaproteobacteria', 'o__Rhodobacterales', 'f__Rhodobacteraceae', 'g__Yoonia', 's__Yoonia sp900143545'], 'CAR1_bin_15': ['d__Bacteria', 'p__Cyanobacteria', 'c__Cyanobacteriia', 'o__Synechococcales', 'f__Cyanobiaceae', 'g__Synechococcus_B', 's__'], 'APA_bin_13': ['d__Bacteria', 'p__Chloroflexota', 'c__Dehalococcoidia', 'o__UBA2979', 'f__', 'g__', 's__'], 'IRC_PAM_SB0667_bin_14': ['d__Bacteria', 'p__Proteobacteria', 'c__Alphaproteobacteria', 'o__Bin65', 'f__Bin65', 'g__Bin65', 's__'], 'GCA_001542995': ['d__Bacteria', 'p__Nitrospinota', 'c__Nitrospinia', 'o__Nitrospinales', 'f__Nitrospinaceae', 'g__LS-NOB', 's__LS-NOB sp001542995'], 'seawater_bettina_36309_bin_6': ['d__Bacteria', 'p__Proteobacteria', 'c__Alphaproteobacteria', 'o__Rhodobacterales', 'f__Rhodobacteraceae', 'g__HIMB11', 's__HIMB11 sp001510135'], 'RHO1_bin_67': ['d__Bacteria', 'p__Acidobacteriota', 'c__Bin61', 'o__Bin61', 'f__Bin61', 'g__Bin61', 's__'], 'GCA_001543005': ['d__Bacteria', 'p__Proteobacteria', 'c__Gammaproteobacteria', 'o__UBA10353', 'f__LS-SOB', 'g__LS-SOB', 's__LS-SOB sp001543005'], 'CHO1_bin_2': ['d__Bacteria', 'p__Bacteroidota', 'c__Bacteroidia', 'o__Flavobacteriales', 'f__Flavobacteriaceae', 'g__Croceitalea', 's__'], 'RHO1_bin_69': ['d__Bacteria', 'p__Gemmatimonadota', 'c__Gemmatimonadetes', 'o__SG8-23', 'f__UBA6960', 'g__Bin94', 's__'], 'IRC_PAM_SB0666_bin_6': ['d__Bacteria', 'p__Chloroflexota', 'c__Dehalococcoidia', 'o__Bin125', 'f__Bin125', 'g__Bin125', 's__'], 'IRC4_bin_44': ['d__Bacteria', 'p__Latescibacterota', 'c__UBA2968', 'o__UBA8231', 'f__GCA-002724215', 'g__GCA-2724215', 's__'], 'RHO3_bin_34': ['d__Bacteria', 'p__Chloroflexota', 'c__Dehalococcoidia', 'o__UBA2985', 'f__UBA2985', 'g__', 's__'], 'RHO2_bin_39': ['d__Bacteria', 'p__Actinobacteriota', 'c__Acidimicrobiia', 'o__Microtrichales', 'f__UBA11606', 'g__UBA11606', 's__'], 'RHO3_bin_37': ['d__Bacteria', 'p__Acidobacteriota', 'c__Acidobacteriae', 'o__Bryobacterales', 'f__UBA6623', 'g__', 's__'], 'IRC_PAM_SB0666_bin_13': ['d__Bacteria', 'p__Proteobacteria', 'c__Alphaproteobacteria', 'o__Rhodobacterales', 'f__Rhodobacteraceae', 'g__Bin36', 's__'], 'COS4_bin_14': ['d__Bacteria', 'p__Actinobacteriota', 'c__Acidimicrobiia', 'o__Microtrichales', 'f__Bin134', 'g__Bin134', 's__'], 'petrosia_ficiformis_bin': ['d__Bacteria', 'p__Latescibacterota', 'c__UBA2968', 'o__UBA2968', 'f__GCA-2709665', 'g__', 's__'], 'seawater_22112_bin_16': ['d__Bacteria', 'p__Proteobacteria', 'c__Gammaproteobacteria', 'o__Pseudomonadales', 'f__Halomonadaceae', 'g__Halomonas', 's__Halomonas aquamarina'], 'IRC1_bin_26': ['d__Bacteria', 'p__Actinobacteriota', 'c__Acidimicrobiia', 'o__Microtrichales', 'f__Bin134', 'g__', 's__'], 'IRC1_bin_19': ['d__Bacteria', 'p__Chloroflexota', 'c__Dehalococcoidia', 'o__Bin125', 'f__Bin125', 'g__Bin125', 's__'], 'APA_bin_68': ['d__Bacteria', 'p__Actinobacteriota', 'c__Acidimicrobiia', 'o__Microtrichales', 'f__UBA11606', 'g__UBA11606', 's__'], 'RHO1_bin_18': ['d__Bacteria', 'p__Chloroflexota', 'c__Anaerolineae', 'o__SBR1031', 'f__A4b', 'g__GCA-2702065', 's__'], 'COS36388_bin_15': ['d__Bacteria', 'p__Planctomycetota', 'c__Planctomycetes', 'o__Pirellulales', 'f__Pirellulaceae', 'g__Rubripirellula', 's__'], 'COS4_bin_18': ['d__Bacteria', 'p__Latescibacterota', 'c__UBA2968', 'o__UBA2968', 'f__', 'g__', 's__'], 'IRC1_bin_23': ['d__Bacteria', 'p__Latescibacterota', 'c__UBA2968', 'o__UBA2968', 'f__', 'g__', 's__'], 'RHO1_bin_27': ['d__Bacteria', 'p__Deinococcota', 'c__Deinococci', 'o__Deinococcales', 'f__Trueperaceae', 'g__MPNL01', 's__MPNL01 sp002239005'], 'seawater_42615_bin_16': ['d__Bacteria', 'p__Bacteroidota', 'c__Bacteroidia', 'o__Flavobacteriales', 'f__Flavobacteriaceae', 'g__MED-G14', 's__MED-G14 sp002697465'], 'APA_bin_45': ['d__Bacteria', 'p__Proteobacteria', 'c__Gammaproteobacteria', 'o__Pseudomonadales', 'f__HTCC2089', 'g__', 's__'], 'IRC_PAM_SB0661_bin_19': ['d__Bacteria', 'p__Chloroflexota', 'c__Dehalococcoidia', 'o__Bin125', 'f__Bin125', 'g__Bin125', 's__'], 'IRC4_bin_15': ['d__Bacteria', 'p__Chloroflexota', 'c__Anaerolineae', 'o__SBR1031', 'f__A4b', 'g__UBA6055', 's__'], 'APA_bin_48': ['d__Bacteria', 'p__Chloroflexota', 'c__Dehalococcoidia', 'o__SAR202', 'f__', 'g__', 's__'], 'IRC_PAM_SB0665_bin_4': ['d__Bacteria', 'p__Actinobacteriota', 'c__Acidimicrobiia', 'o__Microtrichales', 'f__UBA11606', 'g__', 's__'], 'IRC3_bin_5': ['d__Bacteria', 'p__Proteobacteria', 'c__Alphaproteobacteria', 'o__Rhodobacterales', 'f__Rhodobacteraceae', 'g__Bin36', 's__'], 'CAR4_bin_1': ['d__Bacteria', 'p__Proteobacteria', 'c__Alphaproteobacteria', 'o__Rhodobacterales', 'f__Rhodobacteraceae', 'g__Bin36', 's__'], 'IRC_PAM_SB0661_bin_34': ['d__Bacteria', 'p__Chloroflexota', 'c__Anaerolineae', 'o__Caldilineales', 'f__Bin34', 'g__Bin34', 's__'], 'COS36386_bin_32': ['d__Bacteria', 'p__Chloroflexota', 'c__Anaerolineae', 'o__SBR1031', 'f__A4b', 'g__UBA6055', 's__'], 'APA_bin_90': ['d__Bacteria', 'p__Binatota', 'c__Binatia', 'o__Bin18', 'f__Bin18', 'g__Bin18', 's__Bin18 sp002238415'], 'COS36388_bin_5': ['d__Bacteria', 'p__Chloroflexota', 'c__Anaerolineae', 'o__Caldilineales', 'f__Caldilineaceae', 'g__Bin5', 's__'], 'seawater_bettina_36328_bin_7': ['d__Bacteria', 'p__Proteobacteria', 'c__Alphaproteobacteria', 'o__Puniceispirillales', 'f__Puniceispirillaceae', 'g__UBA685', 's__'], 'seawater_seasim_SB9152_S1_bin_12': ['d__Archaea', 'p__Nanoarchaeota', 'c__Nanoarchaeia', 'o__Woesearchaeales', 'f__UBA525', 'g__', 's__'], 'COS36387_bin_15': ['d__Archaea', 'p__Crenarchaeota', 'c__Nitrososphaeria', 'o__Nitrososphaerales', 'f__Nitrosopumilaceae', 'g__', 's__'], 'IRC_PAM_SB0675_bin_12': ['d__Bacteria', 'p__Acidobacteriota', 'c__Bin61', 'o__Bin61', 'f__Bin61', 'g__Bin61', 's__'], 'STY2_bin_2': ['d__Bacteria', 'p__Proteobacteria', 'c__Gammaproteobacteria', 'o__UBA10353', 'f__LS-SOB', 'g__', 's__'], 'IRC_PAM_SB0662_bin_12': ['d__Bacteria', 'p__Actinobacteriota', 'c__Acidimicrobiia', 'o__Microtrichales', 'f__Bin134', 'g__Bin134', 's__'], 'seawater_bettina_36309_bin_5': ['d__Bacteria', 'p__Bacteroidota', 'c__Bacteroidia', 'o__Flavobacteriales', 'f__Flavobacteriaceae', 'g__UBA3478', 's__'], 'RHO2_bin_16': ['d__Bacteria', 'p__Bacteroidota', 'c__Rhodothermia', 'o__Rhodothermales', 'f__Bin80', 'g__', 's__'], 'COS4_bin_1': ['d__Bacteria', 'p__Actinobacteriota', 'c__Acidimicrobiia', 'o__Microtrichales', 'f__UBA11606', 'g__UBA11606', 's__'], 'IRC_PAM_SB0678_bin_9': ['d__Bacteria', 'p__Bacteroidota', 'c__Rhodothermia', 'o__Rhodothermales', 'f__Bin80', 'g__Bin80', 's__'], 'CAR3_bin_16': ['d__Bacteria', 'p__Bacteroidota', 'c__Rhodothermia', 'o__Rhodothermales', 'f__Bin80', 'g__Bin80', 's__'], 'Aquimarina_spAU119': ['d__Bacteria', 'p__Bacteroidota', 'c__Bacteroidia', 'o__Flavobacteriales', 'f__Flavobacteriaceae', 'g__Aquimarina', 's__Aquimarina sp900312735'], 'seawater_bettina_36328_bin_5': ['d__Bacteria', 'p__Planctomycetota', 'c__UBA8108', 'o__UBA1146', 'f__UBA1146', 'g__UBA12191', 's__'], 'RHO3_bin_29': ['d__Bacteria', 'p__Actinobacteriota', 'c__Acidimicrobiia', 'o__Microtrichales', 'f__TK06', 'g__', 's__'], 'IRC1_bin_13': ['d__Bacteria', 'p__Gemmatimonadota', 'c__Gemmatimonadetes', 'o__', 'f__', 'g__', 's__'], 'COS2_bin_23': ['d__Bacteria', 'p__Acidobacteriota', 'c__Vicinamibacteria', 'o__Vicinamibacterales', 'f__UBA8438', 'g__', 's__'], 'IRC4_bin_17': ['d__Bacteria', 'p__Chloroflexota', 'c__Dehalococcoidia', 'o__Bin125', 'f__Bin125', 'g__Bin125', 's__'], 'STY4_bin_7': ['d__Archaea', 'p__Crenarchaeota', 'c__Nitrososphaeria', 'o__Nitrososphaerales', 'f__Nitrosopumilaceae', 'g__Cenarchaeum', 's__'], 'IRC_PAM_SB0662_bin_19': ['d__Bacteria', 'p__Dadabacteria', 'c__UBA1144', 'o__', 'f__', 'g__', 's__'], 'GCA_003635315': ['d__Bacteria', 'p__Poribacteria', 'c__WGA-4E', 'o__WGA-4E', 'f__WGA-3G', 'g__', 's__'], 'IRC_PAM_SB0661_bin_38': ['d__Bacteria', 'p__Acidobacteriota', 'c__Acidobacteriae', 'o__', 'f__', 'g__', 's__'], 'COS2_bin_8': ['d__Bacteria', 'p__Actinobacteriota', 'c__Acidimicrobiia', 'o__Microtrichales', 'f__TK06', 'g__', 's__'], 'COS3_bin_8': ['d__Bacteria', 'p__Nitrospirota', 'c__Nitrospiria', 'o__Nitrospirales', 'f__UBA8639', 'g__Bin75', 's__'], 'RHO1_bin_10': ['d__Bacteria', 'p__Acidobacteriota', 'c__Acidobacteriae', 'o__Bryobacterales', 'f__UBA6623', 'g__', 's__'], 'IRC_PAM_SB0667_bin_2': ['d__Bacteria', 'p__Actinobacteriota', 'c__Acidimicrobiia', 'o__Microtrichales', 'f__TK06', 'g__', 's__'], 'seawater_42617_bin_7': ['d__Bacteria', 'p__Bacteroidota', 'c__Bacteroidia', 'o__Flavobacteriales', 'f__Cryomorphaceae', 'g__UBA10364', 's__'], 'IRC_PAM_SB0665_bin_2': ['d__Bacteria', 'p__Dadabacteria', 'c__UBA1144', 'o__', 'f__', 'g__', 's__'], 'seawater_seasim_SB9153_S2_bin_10': ['d__Bacteria', 'p__Proteobacteria', 'c__Alphaproteobacteria', 'o__Rhodobacterales', 'f__Rhodobacteraceae', 'g__Boseongicola', 's__'], 'IRC_PAM_SB0677_bin_19': ['d__Bacteria', 'p__Gemmatimonadota', 'c__Gemmatimonadetes', 'o__SG8-23', 'f__UBA6960', 'g__Bin94', 's__'], 'IRC_PAM_SB0662_bin_34': ['d__Bacteria', 'p__Chloroflexota', 'c__Dehalococcoidia', 'o__SAR202', 'f__', 'g__', 's__'], 'IRC_PAM_SB0664_bin_21': ['d__Bacteria', 'p__Actinobacteriota', 'c__Acidimicrobiia', 'o__UBA5794', 'f__Bin76', 'g__Bin76', 's__'], 'IRC2_bin_8': ['d__Bacteria', 'p__Chloroflexota', 'c__Dehalococcoidia', 'o__Bin125', 'f__Bin125', 'g__Bin125', 's__'], 'IRC_PAM_SB0661_bin_6': ['d__Bacteria', 'p__Chloroflexota_B', 'c__UBA11872', 'o__UBA11872', 'f__', 'g__', 's__'], 'CLI1_bin_3': ['d__Bacteria', 'p__Proteobacteria', 'c__Alphaproteobacteria', 'o__Parvibaculales', 'f__RS24', 'g__', 's__'], 'APA_bin_1': ['d__Bacteria', 'p__Chloroflexota', 'c__Dehalococcoidia', 'o__Bin125', 'f__Bin125', 'g__Bin125', 's__Bin125 sp002239025'], 'COS3_bin_1': ['d__Bacteria', 'p__Chloroflexota', 'c__Anaerolineae', 'o__SBR1031', 'f__A4b', 'g__UBA6055', 's__'], 'CHO1_bin_4': ['d__Bacteria', 'p__Proteobacteria', 'c__Alphaproteobacteria', 'o__Rickettsiales', 'f__Rickettsiaceae', 'g__GCA-2402195', 's__'], 'RHO1_bin_48': ['d__Bacteria', 'p__Proteobacteria', 'c__Alphaproteobacteria', 'o__SP197', 'f__SP197', 'g__SP197', 's__'], 'IRC_PAM_SB0661_bin_27': ['d__Bacteria', 'p__Latescibacterota', 'c__', 'o__', 'f__', 'g__', 's__'], 'RHO3_bin_10': ['d__Bacteria', 'p__Proteobacteria', 'c__Alphaproteobacteria', 'o__Rhodobacterales', 'f__Rhodobacteraceae', 'g__', 's__'], 'IRC_PAM_SB0677_bin_15': ['d__Bacteria', 'p__Nitrospirota', 'c__Nitrospiria', 'o__Nitrospirales', 'f__UBA8639', 'g__Bin75', 's__'], 'COS36388_bin_13': ['d__Bacteria', 'p__Proteobacteria', 'c__Alphaproteobacteria', 'o__Bin65', 'f__Bin65', 'g__Bin65', 's__'], 'IRC_PAM_SB0661_bin_5': ['d__Bacteria', 'p__Dadabacteria', 'c__UBA1144', 'o__', 'f__', 'g__', 's__'], 'COS3_bin_10': ['d__Bacteria', 'p__Chloroflexota_B', 'c__UBA11872', 'o__UBA11872', 'f__', 'g__', 's__'], 'GCA_003635265': ['d__Bacteria', 'p__Poribacteria', 'c__WGA-4E', 'o__WGA-4E', 'f__', 'g__', 's__'], 'IRC2_bin_2': ['d__Bacteria', 'p__Actinobacteriota', 'c__Acidimicrobiia', 'o__UBA5794', 'f__SZUA-232', 'g__', 's__'], 'IRC_PAM_SB0662_bin_23': ['d__Bacteria', 'p__Gemmatimonadota', 'c__Gemmatimonadetes', 'o__SG8-23', 'f__UBA6960', 'g__Bin94', 's__'], 'IRC_PAM_SB0662_bin_51': ['d__Bacteria', 'p__UBA8248', 'c__UBA8248', 'o__UBA8248', 'f__UBA8248', 'g__Bin107', 's__'], 'APA_bin_83': ['d__Bacteria', 'p__Poribacteria', 'c__WGA-4E', 'o__WGA-4E', 'f__WGA-3G', 'g__', 's__'], 'COS36388_bin_11': ['d__Bacteria', 'p__Chloroflexota', 'c__Anaerolineae', 'o__Caldilineales', 'f__Caldilineaceae', 'g__Bin5', 's__'], 'COS36406_bin_19': ['d__Bacteria', 'p__Binatota', 'c__Binatia', 'o__Bin18', 'f__Bin18', 'g__', 's__'], 'APA_bin_98': ['d__Bacteria', 'p__Actinobacteriota', 'c__Acidimicrobiia', 'o__UBA5794', 'f__SZUA-232', 'g__', 's__'], 'IRC_PAM_SB0667_bin_1': ['d__Bacteria', 'p__Acidobacteriota', 'c__Thermoanaerobaculia', 'o__UBA5704', 'f__', 'g__', 's__'], 'IRC2_bin_3': ['d__Bacteria', 'p__Chloroflexota', 'c__Dehalococcoidia', 'o__Bin125', 'f__Bin125', 'g__Bin125', 's__'], 'IRC_PAM_SB0675_bin_19': ['d__Bacteria', 'p__Acidobacteriota', 'c__Vicinamibacteria', 'o__Vicinamibacterales', 'f__UBA8438', 'g__', 's__'], 'IRC_PAM_SB0664_bin_2': ['d__Bacteria', 'p__Proteobacteria', 'c__Gammaproteobacteria', 'o__Pseudomonadales', 'f__Pseudohongiellaceae', 'g__UBA9145', 's__'], 'IRC4_bin_7': ['d__Bacteria', 'p__Actinobacteriota', 'c__Acidimicrobiia', 'o__UBA5794', 'f__SZUA-232', 'g__', 's__'], 'RHO2_bin_64': ['d__Bacteria', 'p__Spirochaetota', 'c__Spirochaetia', 'o__Spirochaetales', 'f__RBG-16-67-19', 'g__', 's__'], 'RHO3_bin_24': ['d__Bacteria', 'p__Proteobacteria', 'c__Alphaproteobacteria', 'o__Rhodobacterales', 'f__Rhodobacteraceae', 'g__Bin36', 's__'], 'IRC1_bin_16': ['d__Bacteria', 'p__Latescibacterota', 'c__', 'o__', 'f__', 'g__', 's__'], 'CAR2_bin_18': ['d__Bacteria', 'p__Proteobacteria', 'c__Gammaproteobacteria', 'o__UBA10353', 'f__LS-SOB', 'g__', 's__'], 'COS4_bin_19': ['d__Bacteria', 'p__Proteobacteria', 'c__Alphaproteobacteria', 'o__UBA7887', 'f__', 'g__', 's__'], 'seawater_bettina_36328_bin_3': ['d__Bacteria', 'p__Proteobacteria', 'c__Alphaproteobacteria', 'o__Rhodobacterales', 'f__Rhodobacteraceae', 'g__HIMB11', 's__HIMB11 sp001510135'], 'COS1_bin_12': ['d__Bacteria', 'p__Chloroflexota', 'c__Anaerolineae', 'o__SBR1031', 'f__', 'g__', 's__'], 'IRC3_bin_12': ['d__Bacteria', 'p__Actinobacteriota', 'c__Acidimicrobiia', 'o__Microtrichales', 'f__Bin134', 'g__', 's__'], 'RHO1_bin_17': ['d__Bacteria', 'p__Actinobacteriota', 'c__Acidimicrobiia', 'o__Microtrichales', 'f__UBA11606', 'g__UBA11606', 's__'], 'IRC_PAM_SB0677_bin_7': ['d__Bacteria', 'p__Proteobacteria', 'c__Gammaproteobacteria', 'o__Pseudomonadales', 'f__HTCC2089', 'g__UBA2168', 's__'], 'seawater_22112_bin_2': ['d__Bacteria', 'p__Actinobacteriota', 'c__Acidimicrobiia', 'o__Microtrichales', 'f__UBA11606', 'g__UBA11606', 's__UBA11606 sp002694825'], 'IRC2_bin_7': ['d__Bacteria', 'p__Actinobacteriota', 'c__Acidimicrobiia', 'o__Microtrichales', 'f__Bin134', 'g__Bin134', 's__'], 'IRC_PAM_SB0670_bin_22': ['d__Bacteria', 'p__Latescibacterota', 'c__', 'o__', 'f__', 'g__', 's__'], 'GCF_900079515': ['d__Bacteria', 'p__Proteobacteria', 'c__Gammaproteobacteria', 'o__Enterobacterales', 'f__Shewanellaceae', 'g__Shewanella', 's__Shewanella woodyi'], 'IRC_PAM_SB0664_bin_14': ['d__Bacteria', 'p__Actinobacteriota', 'c__Acidimicrobiia', 'o__Microtrichales', 'f__Bin134', 'g__Bin134', 's__'], 'IRC_PAM_SB0664_bin_31': ['d__Bacteria', 'p__Acidobacteriota', 'c__Acidobacteriae', 'o__Bryobacterales', 'f__UBA6623', 'g__', 's__'], 'IRC3_bin_32': ['d__Bacteria', 'p__Acidobacteriota', 'c__Bin61', 'o__Bin61', 'f__Bin61', 'g__Bin61', 's__'], 'RHO3_bin_72': ['d__Bacteria', 'p__Gemmatimonadota', 'c__Gemmatimonadetes', 'o__SG8-23', 'f__UBA6960', 'g__Bin94', 's__'], 'COS3_bin_12': ['d__Bacteria', 'p__Chloroflexota', 'c__Anaerolineae', 'o__SBR1031', 'f__', 'g__', 's__'], 'APA_bin_24': ['d__Bacteria', 'p__Nitrospirota', 'c__Nitrospiria', 'o__Nitrospirales', 'f__UBA8639', 'g__Bin75', 's__Bin75 sp002238765'], 'RHO1_bin_58': ['d__Bacteria', 'p__Proteobacteria', 'c__Gammaproteobacteria', 'o__Pseudomonadales', 'f__HTCC2089', 'g__', 's__'], 'COS36386_bin_1': ['d__Bacteria', 'p__Chloroflexota', 'c__Anaerolineae', 'o__Caldilineales', 'f__Caldilineaceae', 'g__Bin5', 's__'], 'STY1_bin_6': ['d__Bacteria', 'p__Proteobacteria', 'c__Gammaproteobacteria', 'o__UBA6729', 'f__', 'g__', 's__'], 'COS3_bin_4': ['d__Bacteria', 'p__Chloroflexota', 'c__Anaerolineae', 'o__Caldilineales', 'f__Caldilineaceae', 'g__Bin5', 's__'], 'APA_bin_86': ['d__Bacteria', 'p__Chloroflexota', 'c__Dehalococcoidia', 'o__UBA2991', 'f__UBA2991', 'g__UBA2991', 's__'], 'IRC4_bin_39': ['d__Bacteria', 'p__Gemmatimonadota', 'c__Gemmatimonadetes', 'o__SG8-23', 'f__UBA6960', 'g__Bin94', 's__'], 'IRC_PAM_SB0675_bin_5': ['d__Bacteria', 'p__Acidobacteriota', 'c__Thermoanaerobaculia', 'o__UBA5704', 'f__', 'g__', 's__'], 'RHO3_bin_20': ['d__Bacteria', 'p__Nitrospirota', 'c__Nitrospiria', 'o__Nitrospirales', 'f__UBA8639', 'g__Bin75', 's__'], 'APA_bin_56': ['d__Archaea', 'p__Crenarchaeota', 'c__Nitrososphaeria', 'o__Nitrososphaerales', 'f__Nitrosopumilaceae', 'g__', 's__'], 'RHO1_bin_59': ['d__Bacteria', 'p__Proteobacteria', 'c__Alphaproteobacteria', 'o__Defluviicoccales', 'f__Defluviicoccaceae', 'g__Defluviicoccus', 's__'], 'CAR2_bin_1': ['d__Bacteria', 'p__Actinobacteriota', 'c__Acidimicrobiia', 'o__Microtrichales', 'f__Bin134', 'g__Bin134', 's__'], 'RHO1_bin_49': ['d__Bacteria', 'p__Gemmatimonadota', 'c__Gemmatimonadetes', 'o__SG8-23', 'f__UBA6960', 'g__', 's__'], 'IRC_PAM_SB0661_bin_17': ['d__Bacteria', 'p__Chloroflexota', 'c__Anaerolineae', 'o__SBR1031', 'f__A4b', 'g__UBA6055', 's__'], 'COS36386_bin_29': ['d__Bacteria', 'p__Cyanobacteria', 'c__Cyanobacteriia', 'o__Synechococcales', 'f__Cyanobiaceae', 'g__Synechococcus_C', 's__'], 'IRC_PAM_SB0665_bin_12': ['d__Bacteria', 'p__Chloroflexota', 'c__Anaerolineae', 'o__SBR1031', 'f__A4b', 'g__UBA6055', 's__'], 'APA_bin_73': ['d__Bacteria', 'p__Binatota', 'c__Binatia', 'o__UBA9968', 'f__', 'g__', 's__'], 'GCA_001541925': ['d__Archaea', 'p__Crenarchaeota', 'c__Nitrososphaeria', 'o__Nitrososphaerales', 'f__Nitrosopumilaceae', 'g__Nitrosopumilus', 's__Nitrosopumilus sp001541925'], 'COS2_bin_19': ['d__Bacteria', 'p__Chloroflexota', 'c__Anaerolineae', 'o__SBR1031', 'f__A4b', 'g__UBA6055', 's__'], 'IRC3_bin_2': ['d__Bacteria', 'p__Proteobacteria', 'c__Gammaproteobacteria', 'o__Pseudomonadales', 'f__Pseudohongiellaceae', 'g__UBA9145', 's__'], 'APA_bin_5': ['d__Bacteria', 'p__Chloroflexota', 'c__Dehalococcoidia', 'o__UBA1151', 'f__Bin127', 'g__Bin127', 's__Bin127 sp002239045'], 'COS36386_bin_28': ['d__Bacteria', 'p__Binatota', 'c__Binatia', 'o__UBA9968', 'f__', 'g__', 's__'], 'CAR4_bin_3': ['d__Bacteria', 'p__Bacteroidota', 'c__Rhodothermia', 'o__Rhodothermales', 'f__Bin80', 'g__Bin80', 's__'], 'RHO1_bin_42': ['d__Bacteria', 'p__Acidobacteriota', 'c__Acidobacteriae', 'o__Bryobacterales', 'f__UBA6623', 'g__', 's__'], 'IRC1_bin_2': ['d__Bacteria', 'p__Actinobacteriota', 'c__Acidimicrobiia', 'o__UBA5794', 'f__SZUA-232', 'g__', 's__'], 'APA_bin_89': ['d__Bacteria', 'p__Chloroflexota', 'c__Anaerolineae', 'o__Caldilineales', 'f__Caldilineaceae', 'g__Bin5', 's__Bin5 sp002238445'], 'IRC1_bin_30': ['d__Bacteria', 'p__Chloroflexota', 'c__Anaerolineae', 'o__Caldilineales', 'f__Bin34', 'g__Bin34', 's__'], 'COS4_bin_43': ['d__Bacteria', 'p__Chloroflexota', 'c__Anaerolineae', 'o__SBR1031', 'f__A4b', 'g__UBA6055', 's__'], 'IRC_PAM_SB0665_bin_16': ['d__Bacteria', 'p__Gemmatimonadota', 'c__Gemmatimonadetes', 'o__', 'f__', 'g__', 's__'], 'STY3_bin_1': ['d__Bacteria', 'p__Proteobacteria', 'c__Gammaproteobacteria', 'o__UBA10353', 'f__LS-SOB', 'g__', 's__'], 'APA_bin_8': ['d__Bacteria', 'p__Dadabacteria', 'c__UBA1144', 'o__', 'f__', 'g__', 's__'], 'IRC_PAM_SB0665_bin_25': ['d__Bacteria', 'p__Chloroflexota', 'c__Anaerolineae', 'o__Caldilineales', 'f__Caldilineaceae', 'g__Bin5', 's__'], 'IRC_PAM_SB0675_bin_3': ['d__Bacteria', 'p__Proteobacteria', 'c__Gammaproteobacteria', 'o__Pseudomonadales', 'f__HTCC2089', 'g__UBA2168', 's__'], 'IRC_PAM_SB0670_bin_18': ['d__Bacteria', 'p__Gemmatimonadota', 'c__Gemmatimonadetes', 'o__', 'f__', 'g__', 's__'], 'APA_bin_29': ['d__Bacteria', 'p__Acidobacteriota', 'c__Acidobacteriae', 'o__Bryobacterales', 'f__UBA6623', 'g__', 's__'], 'IRC_PAM_SB0663_bin_5': ['d__Archaea', 'p__Crenarchaeota', 'c__Nitrososphaeria', 'o__Nitrososphaerales', 'f__Nitrosopumilaceae', 'g__Cenarchaeum', 's__'], 'RHO3_bin_43': ['d__Archaea', 'p__Crenarchaeota', 'c__Nitrososphaeria', 'o__Nitrososphaerales', 'f__Nitrosopumilaceae', 'g__', 's__'], 'APA_bin_81': ['d__Bacteria', 'p__Bacteroidota', 'c__Bacteroidia', 'o__Cytophagales', 'f__Marinoscillaceae', 'g__', 's__'], 'IRC_PAM_SB0661_bin_40': ['d__Bacteria', 'p__Gemmatimonadota', 'c__Gemmatimonadetes', 'o__', 'f__', 'g__', 's__'], 'APA_bin_17': ['d__Bacteria', 'p__Actinobacteriota', 'c__Acidimicrobiia', 'o__UBA5794', 'f__SZUA-232', 'g__', 's__'], 'IRC_PAM_SB0666_bin_9': ['d__Bacteria', 'p__Actinobacteriota', 'c__Acidimicrobiia', 'o__UBA5794', 'f__Bin76', 'g__Bin76', 's__'], 'RHO3_bin_53': ['d__Bacteria', 'p__Chloroflexota', 'c__Anaerolineae', 'o__Caldilineales', 'f__Caldilineaceae', 'g__Bin5', 's__'], 'IRC_PAM_SB0664_bin_11': ['d__Bacteria', 'p__Chloroflexota', 'c__Dehalococcoidia', 'o__UBA2963', 'f__UBA2963', 'g__Bin16', 's__'], 'COS36387_bin_20': ['d__Bacteria', 'p__Chloroflexota', 'c__Anaerolineae', 'o__SBR1031', 'f__A4b', 'g__UBA6055', 's__'], 'IRC_PAM_SB0677_bin_8': ['d__Bacteria', 'p__Bacteroidota', 'c__Rhodothermia', 'o__Rhodothermales', 'f__Bin80', 'g__Bin80', 's__'], 'IRC_PAM_SB0678_bin_5': ['d__Bacteria', 'p__Actinobacteriota', 'c__Acidimicrobiia', 'o__Microtrichales', 'f__TK06', 'g__', 's__'], 'COS36387_bin_18': ['d__Bacteria', 'p__Planctomycetota', 'c__Planctomycetes', 'o__Pirellulales', 'f__Pirellulaceae', 'g__Mariniblastus', 's__'], 'COS36404_bin_3': ['d__Bacteria', 'p__Chloroflexota', 'c__Anaerolineae', 'o__Caldilineales', 'f__Caldilineaceae', 'g__Bin5', 's__'], 'IRC4_bin_37': ['d__Bacteria', 'p__Actinobacteriota', 'c__Acidimicrobiia', 'o__Microtrichales', 'f__Bin134', 'g__', 's__'], 'IRC4_bin_8': ['d__Bacteria', 'p__Chloroflexota', 'c__Anaerolineae', 'o__Caldilineales', 'f__Caldilineaceae', 'g__Bin5', 's__'], 'RHO1_bin_25': ['d__Bacteria', 'p__Actinobacteriota', 'c__Acidimicrobiia', 'o__Microtrichales', 'f__TK06', 'g__', 's__'], 'RHO3_bin_1': ['d__Bacteria', 'p__Proteobacteria', 'c__Alphaproteobacteria', 'o__Bin65', 'f__Bin65', 'g__Bin65', 's__'], 'IRC_PAM_SB0677_bin_14': ['d__Bacteria', 'p__Actinobacteriota', 'c__Acidimicrobiia', 'o__Microtrichales', 'f__TK06', 'g__', 's__'], 'IRC_PAM_SB0661_bin_2': ['d__Bacteria', 'p__Gemmatimonadota', 'c__Gemmatimonadetes', 'o__SG8-23', 'f__UBA6960', 'g__Bin94', 's__'], 'IRC_PAM_SB0661_bin_31': ['d__Bacteria', 'p__Gemmatimonadota', 'c__Gemmatimonadetes', 'o__SG8-23', 'f__UBA6960', 'g__BD2-11', 's__'], 'IRC3_bin_16': ['d__Bacteria', 'p__Acidobacteriota', 'c__Acidobacteriae', 'o__Bryobacterales', 'f__UBA6623', 'g__', 's__'], 'RHO3_bin_36': ['d__Bacteria', 'p__Acidobacteriota', 'c__Acidobacteriae', 'o__Bryobacterales', 'f__UBA6623', 'g__', 's__'], 'CAR1_bin_13': ['d__Bacteria', 'p__Proteobacteria', 'c__Alphaproteobacteria', 'o__Rhodobacterales', 'f__Rhodobacteraceae', 'g__Rhodobacter_B', 's__'], 'RHO1_bin_37': ['d__Archaea', 'p__Crenarchaeota', 'c__Nitrososphaeria', 'o__Nitrososphaerales', 'f__Nitrosopumilaceae', 'g__', 's__'], 'seawater_seasim_SB9152_S1_bin_1': ['d__Bacteria', 'p__Proteobacteria', 'c__Gammaproteobacteria', 'o__Pseudomonadales', 'f__Pseudohongiellaceae', 'g__UBA9145', 's__'], 'IRC_PAM_SB0665_bin_24': ['d__Bacteria', 'p__Actinobacteriota', 'c__Acidimicrobiia', 'o__UBA5794', 'f__Bin76', 'g__Bin76', 's__'], 'GCF_900149685': ['d__Bacteria', 'p__Proteobacteria', 'c__Alphaproteobacteria', 'o__Sphingomonadales', 'f__Sphingomonadaceae', 'g__Erythrobacter', 's__Erythrobacter sp900149685'], 'IRC3_bin_21': ['d__Bacteria', 'p__Poribacteria', 'c__WGA-4E', 'o__WGA-4E', 'f__WGA-3G', 'g__WGA-3G', 's__'], 'RHO2_bin_37': ['d__Bacteria', 'p__Actinobacteriota', 'c__Acidimicrobiia', 'o__Microtrichales', 'f__TK06', 'g__', 's__'], 'STY2_bin_1': ['d__Bacteria', 'p__Proteobacteria', 'c__Gammaproteobacteria', 'o__UBA10353', 'f__LS-SOB', 'g__', 's__'], 'STY4_bin_3': ['d__Bacteria', 'p__Proteobacteria', 'c__Gammaproteobacteria', 'o__UBA6729', 'f__', 'g__', 's__'], 'IRC4_bin_16': ['d__Bacteria', 'p__Actinobacteriota', 'c__Acidimicrobiia', 'o__Microtrichales', 'f__TK06', 'g__', 's__'], 'IRC3_bin_19': ['d__Bacteria', 'p__Gemmatimonadota', 'c__Gemmatimonadetes', 'o__SG8-23', 'f__UBA6960', 'g__Bin94', 's__'], 'CAR4_bin_2': ['d__Bacteria', 'p__Bacteroidota', 'c__Rhodothermia', 'o__Rhodothermales', 'f__Bin80', 'g__Bin80', 's__'], 'COS4_bin_51': ['d__Bacteria', 'p__Chloroflexota_B', 'c__UBA11872', 'o__UBA11872', 'f__UBA11872', 'g__', 's__'], 'seawater_42618_bin_9': ['d__Bacteria', 'p__Proteobacteria', 'c__Alphaproteobacteria', 'o__Puniceispirillales', 'f__Puniceispirillaceae', 'g__HIMB100', 's__HIMB100 sp002700485'], 'IRC_PAM_SB0678_bin_15': ['d__Bacteria', 'p__Proteobacteria', 'c__Gammaproteobacteria', 'o__Pseudomonadales', 'f__HTCC2089', 'g__UBA2168', 's__'], 'RHO3_bin_48': ['d__Bacteria', 'p__Gemmatimonadota', 'c__Gemmatimonadetes', 'o__SG8-23', 'f__', 'g__', 's__'], 'RHO1_bin_39': ['d__Bacteria', 'p__Proteobacteria', 'c__Alphaproteobacteria', 'o__Rhodobacterales', 'f__Rhodobacteraceae', 'g__Bin36', 's__'], 'IRC_PAM_SB0667_bin_3': ['d__Bacteria', 'p__Bacteroidota', 'c__Rhodothermia', 'o__Rhodothermales', 'f__Bin80', 'g__Bin80', 's__'], 'APA_bin_26': ['d__Bacteria', 'p__Bacteroidota', 'c__Rhodothermia', 'o__Rhodothermales', 'f__Bin80', 'g__Bin80', 's__Bin80 sp002238805'], 'COS36386_bin_18': ['d__Bacteria', 'p__Chloroflexota', 'c__Anaerolineae', 'o__SBR1031', 'f__', 'g__', 's__'], 'APA_bin_32': ['d__Bacteria', 'p__Chloroflexota', 'c__Dehalococcoidia', 'o__UBA6926', 'f__UBA6926', 'g__', 's__'], 'COS4_bin_5': ['d__Bacteria', 'p__Nitrospirota', 'c__Nitrospiria', 'o__Nitrospirales', 'f__UBA8639', 'g__Bin75', 's__'], 'CAR3_bin_5': ['d__Bacteria', 'p__Cyanobacteria', 'c__Cyanobacteriia', 'o__Synechococcales', 'f__Cyanobiaceae', 'g__Synechococcus_B', 's__'], 'CAR3_bin_12': ['d__Bacteria', 'p__Proteobacteria', 'c__Gammaproteobacteria', 'o__UBA10353', 'f__LS-SOB', 'g__', 's__'], 'GCA_001543015': ['d__Archaea', 'p__Crenarchaeota', 'c__Nitrososphaeria', 'o__Nitrososphaerales', 'f__Nitrosopumilaceae', 'g__Nitrosopumilus', 's__Nitrosopumilus sp001543015'], 'seawater_bettina_36310_bin_3': ['d__Bacteria', 'p__Proteobacteria', 'c__Alphaproteobacteria', 'o__Rhodobacterales', 'f__Rhodobacteraceae', 'g__HIMB11', 's__HIMB11 sp001510135'], 'COS1_bin_9': ['d__Bacteria', 'p__Cyanobacteria', 'c__Cyanobacteriia', 'o__Synechococcales', 'f__Cyanobiaceae', 'g__Synechococcus_C', 's__Synechococcus_C sp002724845'], 'IRC_PAM_SB0675_bin_22': ['d__Bacteria', 'p__Poribacteria', 'c__WGA-4E', 'o__WGA-4E', 'f__WGA-3G', 'g__WGA-3G', 's__'], 'RHO2_bin_38': ['d__Bacteria', 'p__Actinobacteriota', 'c__Acidimicrobiia', 'o__Microtrichales', 'f__UBA11606', 'g__', 's__'], 'IRC1_bin_4': ['d__Bacteria', 'p__Bacteroidota', 'c__Rhodothermia', 'o__Rhodothermales', 'f__Bin80', 'g__Bin80', 's__'], 'GCF_900143535': ['d__Bacteria', 'p__Proteobacteria', 'c__Alphaproteobacteria', 'o__Rhodobacterales', 'f__Rhodobacteraceae', 'g__Tateyamaria', 's__Tateyamaria sp900143535'], 'RHO2_bin_53': ['d__Bacteria', 'p__Gemmatimonadota', 'c__Gemmatimonadetes', 'o__SG8-23', 'f__UBA6960', 'g__Bin94', 's__'], 'CLI1_bin_4': ['d__Bacteria', 'p__Proteobacteria', 'c__Alphaproteobacteria', 'o__Parvibaculales', 'f__RS24', 'g__', 's__'], 'IRC1_bin_27': ['d__Bacteria', 'p__Latescibacterota', 'c__UBA2968', 'o__UBA8231', 'f__GCA-002724215', 'g__GCA-2724215', 's__'], 'IRC_PAM_SB0662_bin_36': ['d__Bacteria', 'p__Actinobacteriota', 'c__Acidimicrobiia', 'o__UBA5794', 'f__SZUA-232', 'g__', 's__'], 'seawater_22112_bin_4': ['d__Bacteria', 'p__Proteobacteria', 'c__Alphaproteobacteria', 'o__Puniceispirillales', 'f__Puniceispirillaceae', 'g__UBA8309', 's__UBA8309 sp001627655'], 'COS36388_bin_4': ['d__Bacteria', 'p__Actinobacteriota', 'c__Acidimicrobiia', 'o__UBA5794', 'f__SZUA-232', 'g__', 's__'], 'COS4_bin_20': ['d__Bacteria', 'p__Acidobacteriota', 'c__Vicinamibacteria', 'o__Vicinamibacterales', 'f__UBA8438', 'g__', 's__'], 'seawater_seasim_SB9156_S5_bin_6': ['d__Bacteria', 'p__Proteobacteria', 'c__Gammaproteobacteria', 'o__Nevskiales', 'f__', 'g__', 's__'], 'IRC_PAM_SB0662_bin_10': ['d__Bacteria', 'p__Chloroflexota', 'c__Anaerolineae', 'o__SBR1031', 'f__A4b', 'g__UBA6055', 's__'], 'IRC_PAM_SB0662_bin_33': ['d__Archaea', 'p__Crenarchaeota', 'c__Nitrososphaeria', 'o__Nitrososphaerales', 'f__Nitrosopumilaceae', 'g__Cenarchaeum', 's__'], 'CAR1_bin_11': ['d__Bacteria', 'p__Proteobacteria', 'c__Gammaproteobacteria', 'o__HK1', 'f__HK1', 'g__', 's__'], 'COS4_bin_27': ['d__Bacteria', 'p__Acidobacteriota', 'c__Vicinamibacteria', 'o__Vicinamibacterales', 'f__UBA8438', 'g__', 's__'], 'RHO1_bin_9': ['d__Bacteria', 'p__Chloroflexota', 'c__Dehalococcoidia', 'o__UBA2985', 'f__UBA2985', 'g__', 's__'], 'STY1_bin_3': ['d__Bacteria', 'p__Nitrospirota', 'c__Nitrospiria', 'o__Nitrospirales', 'f__UBA8639', 'g__Bin75', 's__'], 'IRC_PAM_SB0666_bin_21': ['d__Bacteria', 'p__Chloroflexota', 'c__Anaerolineae', 'o__Caldilineales', 'f__Bin34', 'g__Bin34', 's__'], 'RHO1_bin_31': ['d__Bacteria', 'p__Proteobacteria', 'c__Gammaproteobacteria', 'o__UBA4486', 'f__UBA4486', 'g__', 's__'], 'seawater_22112_bin_1': ['d__Bacteria', 'p__Bacteroidota', 'c__Bacteroidia', 'o__Chitinophagales', 'f__Chitinophagaceae', 'g__UBA12329', 's__'], 'RHO2_bin_11': ['d__Bacteria', 'p__Nitrospirota', 'c__Nitrospiria', 'o__Nitrospirales', 'f__UBA8639', 'g__Bin75', 's__'], 'COS1_bin_18': ['d__Bacteria', 'p__Proteobacteria', 'c__Gammaproteobacteria', 'o__Pseudomonadales', 'f__HTCC2089', 'g__', 's__'], 'IRC3_bin_22': ['d__Bacteria', 'p__Chloroflexota', 'c__Dehalococcoidia', 'o__UBA2991', 'f__UBA2991', 'g__UBA2991', 's__'], 'COS36386_bin_9': ['d__Bacteria', 'p__Proteobacteria', 'c__Alphaproteobacteria', 'o__Bin65', 'f__Bin65', 'g__Bin65', 's__'], 'CAR3_bin_14': ['d__Bacteria', 'p__Proteobacteria', 'c__Gammaproteobacteria', 'o__UBA4486', 'f__UBA4486', 'g__', 's__'], 'RHO2_bin_2': ['d__Bacteria', 'p__Proteobacteria', 'c__Alphaproteobacteria', 'o__Defluviicoccales', 'f__Defluviicoccaceae', 'g__Defluviicoccus', 's__'], 'CAR2_bin_6': ['d__Bacteria', 'p__Bacteroidota', 'c__Rhodothermia', 'o__Rhodothermales', 'f__Bin80', 'g__Bin80', 's__'], 'IRC_PAM_SB0672_bin_21': ['d__Bacteria', 'p__Gemmatimonadota', 'c__Gemmatimonadetes', 'o__SG8-23', 'f__UBA6960', 'g__Bin94', 's__'], 'IRC_PAM_SB0666_bin_17': ['d__Bacteria', 'p__Dadabacteria', 'c__UBA1144', 'o__', 'f__', 'g__', 's__'], 'COS36387_bin_11': ['d__Bacteria', 'p__Proteobacteria', 'c__Gammaproteobacteria', 'o__Coxiellales', 'f__Coxiellaceae', 'g__UBA9148', 's__'], 'RHO2_bin_41': ['d__Bacteria', 'p__Chloroflexota', 'c__Anaerolineae', 'o__SBR1031', 'f__A4b', 'g__UBA6055', 's__'], 'IRC_PAM_SB0670_bin_19': ['d__Bacteria', 'p__Chloroflexota', 'c__Dehalococcoidia', 'o__Bin125', 'f__Bin125', 'g__Bin125', 's__'], 'RHO3_bin_68': ['d__Bacteria', 'p__Chloroflexota', 'c__Dehalococcoidia', 'o__Bin125', 'f__Bin125', 'g__Bin125', 's__'], 'CAR3_bin_2': ['d__Bacteria', 'p__Proteobacteria', 'c__Gammaproteobacteria', 'o__Pseudomonadales', 'f__Pseudohongiellaceae', 'g__', 's__'], 'COS36388_bin_16': ['d__Bacteria', 'p__Chloroflexota', 'c__Anaerolineae', 'o__SBR1031', 'f__A4b', 'g__UBA6055', 's__'], 'COS4_bin_4': ['d__Bacteria', 'p__Proteobacteria', 'c__Gammaproteobacteria', 'o__UBA10353', 'f__LS-SOB', 'g__LS-SOB', 's__'], 'COS4_bin_42': ['d__Bacteria', 'p__Chloroflexota', 'c__Anaerolineae', 'o__SBR1031', 'f__A4b', 'g__UBA6055', 's__'], 'IRC_PAM_SB0662_bin_7': ['d__Bacteria', 'p__Proteobacteria', 'c__Alphaproteobacteria', 'o__UBA7887', 'f__', 'g__', 's__'], 'COS4_bin_55': ['d__Bacteria', 'p__Proteobacteria', 'c__Alphaproteobacteria', 'o__Bin95', 'f__Bin95', 'g__Bin95', 's__'], 'STY3_bin_6': ['d__Archaea', 'p__Crenarchaeota', 'c__Nitrososphaeria', 'o__Nitrososphaerales', 'f__Nitrosopumilaceae', 'g__Cenarchaeum', 's__'], 'GCA_000522425': ['d__Bacteria', 'p__Tectomicrobia', 'c__Entotheonellia', 'o__Entotheonellales', 'f__Entotheonellaceae', 'g__Entotheonella', 's__Entotheonella factor'], 'RHO1_bin_3': ['d__Bacteria', 'p__Actinobacteriota', 'c__Acidimicrobiia', 'o__UBA5794', 'f__SZUA-232', 'g__', 's__'], 'IRC_PAM_SB0661_bin_30': ['d__Bacteria', 'p__Acidobacteriota', 'c__Acidobacteriae', 'o__Bryobacterales', 'f__UBA6623', 'g__', 's__'], 'RHO3_bin_38': ['d__Bacteria', 'p__Gemmatimonadota', 'c__Gemmatimonadetes', 'o__', 'f__', 'g__', 's__'], 'COS36405_bin_8': ['d__Bacteria', 'p__Actinobacteriota', 'c__Acidimicrobiia', 'o__UBA5794', 'f__SZUA-232', 'g__', 's__'], 'RHO3_bin_47': ['d__Bacteria', 'p__Gemmatimonadota', 'c__Gemmatimonadetes', 'o__SG8-23', 'f__UBA6960', 'g__BD2-11', 's__'], 'IRC3_bin_13': ['d__Bacteria', 'p__Chloroflexota', 'c__Dehalococcoidia', 'o__Bin125', 'f__Bin125', 'g__Bin125', 's__'], 'IRC_PAM_SB0661_bin_37': ['d__Bacteria', 'p__Actinobacteriota', 'c__Acidimicrobiia', 'o__UBA5794', 'f__SZUA-232', 'g__', 's__'], 'IRC_PAM_SB0662_bin_9': ['d__Bacteria', 'p__Chloroflexota', 'c__Anaerolineae', 'o__Caldilineales', 'f__Bin34', 'g__Bin34', 's__'], 'CYMC_moitinhoThomas_67496': ['d__Archaea', 'p__Crenarchaeota', 'c__Nitrososphaeria', 'o__Nitrososphaerales', 'f__Nitrosopumilaceae', 'g__Nitrosopumilus', 's__'], 'RHO1_bin_66': ['d__Bacteria', 'p__Gemmatimonadota', 'c__Gemmatimonadetes', 'o__SG8-23', 'f__', 'g__', 's__'], 'RHO3_bin_9': ['d__Bacteria', 'p__Proteobacteria', 'c__Gammaproteobacteria', 'o__Pseudomonadales', 'f__HTCC2089', 'g__UBA2168', 's__'], 'RHO1_bin_52': ['d__Bacteria', 'p__Proteobacteria', 'c__Gammaproteobacteria', 'o__UBA4575', 'f__UBA4575', 'g__', 's__'], 'RHO1_bin_7': ['d__Bacteria', 'p__Chloroflexota_B', 'c__UBA11872', 'o__', 'f__', 'g__', 's__'], 'IRC_PAM_SB0664_bin_5': ['d__Bacteria', 'p__Proteobacteria', 'c__Alphaproteobacteria', 'o__Bin65', 'f__Bin65', 'g__Bin65', 's__'], 'RHO2_bin_65': ['d__Bacteria', 'p__Spirochaetota', 'c__Spirochaetia', 'o__Spirochaetales', 'f__RBG-16-67-19', 'g__Bin103', 's__'], 'APA_bin_96': ['d__Bacteria', 'p__Chloroflexota', 'c__Dehalococcoidia', 'o__UBA3495', 'f__UBA3495', 'g__Bin22', 's__Bin22 sp002238505'], 'CAR4_bin_5': ['d__Bacteria', 'p__Proteobacteria', 'c__Alphaproteobacteria', 'o__Rhodobacterales', 'f__Rhodobacteraceae', 'g__Bin36', 's__'], 'seawater_seasim_SB9155_S4_bin_4': ['d__Bacteria', 'p__Proteobacteria', 'c__Alphaproteobacteria', 'o__Micavibrionales', 'f__UBA2020', 'g__', 's__'], 'CAR1_bin_6': ['d__Bacteria', 'p__Proteobacteria', 'c__Alphaproteobacteria', 'o__Rhodobacterales', 'f__Rhodobacteraceae', 'g__Aestuariivita', 's__'], 'IRC_PAM_SB0673_bin_10': ['d__Bacteria', 'p__Cyanobacteria', 'c__Cyanobacteriia', 'o__Synechococcales', 'f__Cyanobiaceae', 'g__Synechococcus_B', 's__'], 'COS1_bin_20': ['d__Bacteria', 'p__Chloroflexota', 'c__Anaerolineae', 'o__SBR1031', 'f__A4b', 'g__UBA6055', 's__'], 'RHO3_bin_50': ['d__Bacteria', 'p__Chloroflexota', 'c__Anaerolineae', 'o__SBR1031', 'f__A4b', 'g__UBA6055', 's__'], 'COS1_bin_19': ['d__Bacteria', 'p__Chloroflexota', 'c__Anaerolineae', 'o__SBR1031', 'f__A4b', 'g__UBA6055', 's__'], 'seawater_22112_bin_5': ['d__Bacteria', 'p__Planctomycetota', 'c__Planctomycetes', 'o__Pirellulales', 'f__Pirellulaceae', 'g__Rubripirellula', 's__'], 'CAR1_bin_5': ['d__Bacteria', 'p__Bacteroidota', 'c__Bacteroidia', 'o__Flavobacteriales', 'f__Flavobacteriaceae', 'g__Bin25', 's__'], 'seawater_42618_bin_29': ['d__Bacteria', 'p__Proteobacteria', 'c__Alphaproteobacteria', 'o__Puniceispirillales', 'f__Puniceispirillaceae', 'g__UBA5951', 's__'], 'RHO2_bin_40': ['d__Bacteria', 'p__Chloroflexota', 'c__Anaerolineae', 'o__SBR1031', 'f__A4b', 'g__UBA6055', 's__'], 'IRC_PAM_SB0664_bin_22': ['d__Bacteria', 'p__Chloroflexota', 'c__Anaerolineae', 'o__Caldilineales', 'f__Bin34', 'g__Bin34', 's__'], 'CAR1_bin_7': ['d__Bacteria', 'p__Proteobacteria', 'c__Alphaproteobacteria', 'o__Rhodobacterales', 'f__Rhodobacteraceae', 'g__Aestuariivita', 's__'], 'APA_bin_28': ['d__Bacteria', 'p__Acidobacteriota', 'c__Vicinamibacteria', 'o__Vicinamibacterales', 'f__UBA8438', 'g__', 's__'], 'CAR3_bin_11': ['d__Bacteria', 'p__Bacteroidota', 'c__Bacteroidia', 'o__Flavobacteriales', 'f__Flavobacteriaceae', 'g__Bin25', 's__'], 'IRC_PAM_SB0678_bin_7': ['d__Bacteria', 'p__Proteobacteria', 'c__Gammaproteobacteria', 'o__UBA10353', 'f__LS-SOB', 'g__', 's__'], 'seawater_seasim_SB9156_S5_bin_20': ['d__Bacteria', 'p__Bacteroidota', 'c__Rhodothermia', 'o__Rhodothermales', 'f__UBA10348', 'g__UBA10348', 's__'], 'COS4_bin_2': ['d__Bacteria', 'p__Gemmatimonadota', 'c__Gemmatimonadetes', 'o__SG8-23', 'f__UBA6960', 'g__Bin94', 's__'], 'STY4_bin_2': ['d__Bacteria', 'p__Nitrospirota', 'c__Nitrospiria', 'o__Nitrospirales', 'f__UBA8639', 'g__Bin75', 's__'], 'IRC1_bin_20': ['d__Bacteria', 'p__Chloroflexota', 'c__Dehalococcoidia', 'o__UBA3495', 'f__UBA3495', 'g__Bin87', 's__'], 'COS36404_bin_9': ['d__Bacteria', 'p__Actinobacteriota', 'c__Acidimicrobiia', 'o__Microtrichales', 'f__TK06', 'g__', 's__'], 'IRC_PAM_SB0661_bin_3': ['d__Bacteria', 'p__Acidobacteriota', 'c__Vicinamibacteria', 'o__Vicinamibacterales', 'f__UBA8438', 'g__', 's__'], 'IRC_PAM_SB0662_bin_58': ['d__Bacteria', 'p__Proteobacteria', 'c__Gammaproteobacteria', 'o__Pseudomonadales', 'f__HTCC2089', 'g__', 's__'], 'CLI3_bin_1': ['d__Bacteria', 'p__Dadabacteria', 'c__UBA1144', 'o__', 'f__', 'g__', 's__'], 'COS3_bin_2': ['d__Bacteria', 'p__Chloroflexota', 'c__Anaerolineae', 'o__SBR1031', 'f__A4b', 'g__GCA-2702065', 's__'], 'RHO1_bin_34': ['d__Bacteria', 'p__Acidobacteriota', 'c__Acidobacteriae', 'o__', 'f__', 'g__', 's__'], 'RHO2_bin_28': ['d__Bacteria', 'p__Bacteroidota', 'c__Rhodothermia', 'o__Rhodothermales', 'f__Bin80', 'g__Bin80', 's__'], 'RHO3_bin_27': ['d__Bacteria', 'p__Acidobacteriota', 'c__Thermoanaerobaculia', 'o__UBA5704', 'f__', 'g__', 's__'], 'COS4_bin_13': ['d__Bacteria', 'p__Chloroflexota', 'c__Anaerolineae', 'o__SBR1031', 'f__A4b', 'g__UBA6055', 's__'], 'RHO3_bin_58': ['d__Bacteria', 'p__Proteobacteria', 'c__Alphaproteobacteria', 'o__UBA828', 'f__UBA828', 'g__', 's__'], 'RHO2_bin_18': ['d__Bacteria', 'p__Chloroflexota', 'c__Anaerolineae', 'o__Caldilineales', 'f__Caldilineaceae', 'g__Bin5', 's__'], 'IRC3_bin_11': ['d__Bacteria', 'p__Chloroflexota', 'c__Dehalococcoidia', 'o__Bin125', 'f__Bin125', 'g__Bin125', 's__'], 'RHO2_bin_67': ['d__Bacteria', 'p__Chloroflexota', 'c__Anaerolineae', 'o__Caldilineales', 'f__Caldilineaceae', 'g__Bin5', 's__'], 'seawater_42618_bin_2': ['d__Bacteria', 'p__Planctomycetota', 'c__UBA8108', 'o__UBA1146', 'f__UBA1146', 'g__UBA12191', 's__'], 'RHO1_bin_68': ['d__Bacteria', 'p__Chloroflexota', 'c__Dehalococcoidia', 'o__UBA3495', 'f__UBA3495', 'g__Bin87', 's__'], 'APA_bin_15': ['d__Bacteria', 'p__Chloroflexota', 'c__Dehalococcoidia', 'o__UBA1127', 'f__UBA1127', 'g__UBA1127', 's__'], 'IRC_PAM_SB0664_bin_16': ['d__Bacteria', 'p__Chloroflexota', 'c__Anaerolineae', 'o__SBR1031', 'f__A4b', 'g__UBA6055', 's__'], 'IRC_PAM_SB0662_bin_21': ['d__Bacteria', 'p__Chloroflexota', 'c__Dehalococcoidia', 'o__Bin125', 'f__Bin125', 'g__Bin125', 's__'], 'CAR4_bin_8': ['d__Bacteria', 'p__Proteobacteria', 'c__Alphaproteobacteria', 'o__Rhodobacterales', 'f__Rhodobacteraceae', 'g__Rhodobacter_B', 's__'], 'IRC_PAM_SB0676_bin_26': ['d__Bacteria', 'p__Gemmatimonadota', 'c__Gemmatimonadetes', 'o__SG8-23', 'f__UBA6960', 'g__BD2-11', 's__'], 'IRC1_bin_9': ['d__Bacteria', 'p__Actinobacteriota', 'c__Acidimicrobiia', 'o__Microtrichales', 'f__TK06', 'g__', 's__'], 'RHO3_bin_59': ['d__Bacteria', 'p__Proteobacteria', 'c__Alphaproteobacteria', 'o__SP197', 'f__SP197', 'g__SP197', 's__'], 'IRC4_bin_1': ['d__Bacteria', 'p__Acidobacteriota', 'c__Acidobacteriae', 'o__', 'f__', 'g__', 's__'], 'GCA_001007625': ['d__Bacteria', 'p__Cyanobacteria', 'c__Cyanobacteriia', 'o__Synechococcales', 'f__Cyanobiaceae', 'g__Synechococcus_B', 's__'], 'COS4_bin_11': ['d__Bacteria', 'p__Proteobacteria', 'c__Alphaproteobacteria', 'o__Rhodobacterales', 'f__Rhodobacteraceae', 'g__Bin36', 's__'], 'RHO2_bin_19': ['d__Bacteria', 'p__Poribacteria', 'c__WGA-4E', 'o__WGA-4E', 'f__WGA-3G', 'g__WGA-3G', 's__'], 'RHO2_bin_24': ['d__Bacteria', 'p__Actinobacteriota', 'c__Acidimicrobiia', 'o__UBA5794', 'f__SZUA-232', 'g__', 's__'], 'RHO1_bin_1': ['d__Bacteria', 'p__Proteobacteria', 'c__Alphaproteobacteria', 'o__Bin65', 'f__Bin65', 'g__Bin65', 's__'], 'IRC_PAM_SB0661_bin_43': ['d__Bacteria', 'p__Chloroflexota', 'c__Dehalococcoidia', 'o__UBA2979', 'f__', 'g__', 's__'], 'COS36387_bin_4': ['d__Bacteria', 'p__Planctomycetota', 'c__Planctomycetes', 'o__Pirellulales', 'f__Pirellulaceae', 'g__Rubripirellula', 's__'], 'COS3_bin_11': ['d__Bacteria', 'p__Gemmatimonadota', 'c__Gemmatimonadetes', 'o__SG8-23', 'f__UBA6960', 'g__Bin94', 's__'], 'COS36387_bin_14': ['d__Bacteria', 'p__Proteobacteria', 'c__Alphaproteobacteria', 'o__Rhizobiales', 'f__Stappiaceae', 'g__Pseudovibrio', 's__Pseudovibrio denitrificans'], 'seawater_bettina_36326_bin_26': ['d__Bacteria', 'p__Bacteroidota', 'c__Bacteroidia', 'o__Flavobacteriales', 'f__UA16', 'g__UBA11663', 's__'], 'COS2_bin_12': ['d__Bacteria', 'p__Proteobacteria', 'c__Alphaproteobacteria', 'o__UBA7887', 'f__', 'g__', 's__'], 'RHO1_bin_50': ['d__Bacteria', 'p__Gemmatimonadota', 'c__Gemmatimonadetes', 'o__', 'f__', 'g__', 's__'], 'IRC3_bin_18': ['d__Bacteria', 'p__Latescibacterota', 'c__', 'o__', 'f__', 'g__', 's__'], 'seawater_22112_bin_3': ['d__Bacteria', 'p__Proteobacteria', 'c__Gammaproteobacteria', 'o__Enterobacterales', 'f__Alteromonadaceae', 'g__Alteromonas', 's__Alteromonas macleodii'], 'APA_bin_80': ['d__Bacteria', 'p__Chloroflexota', 'c__Dehalococcoidia', 'o__UBA3495', 'f__UBA3495', 'g__Bin87', 's__'], 'seawater_seasim_SB9156_S5_bin_3': ['d__Bacteria', 'p__Proteobacteria', 'c__Gammaproteobacteria', 'o__Pseudomonadales', 'f__Pseudohongiellaceae', 'g__UBA9145', 's__'], 'seawater_seasim_SB9152_S1_bin_4': ['d__Bacteria', 'p__Marinisomatota', 'c__Marinisomatia', 'o__Marinisomatales', 'f__TCS55', 'g__TCS55', 's__TCS55 sp001577025'], 'Pseudovibrio_spAU243': ['d__Bacteria', 'p__Proteobacteria', 'c__Alphaproteobacteria', 'o__Rhizobiales', 'f__Stappiaceae', 'g__Pseudovibrio', 's__Pseudovibrio ascidiaceicola'], 'RHO3_bin_26': ['d__Bacteria', 'p__Chloroflexota', 'c__Anaerolineae', 'o__Caldilineales', 'f__Caldilineaceae', 'g__Bin5', 's__'], 'IRC_PAM_SB0673_bin_16': ['d__Bacteria', 'p__Latescibacterota', 'c__UBA2968', 'o__UBA8231', 'f__GCA-002724215', 'g__GCA-2724215', 's__'], 'RHO2_bin_9': ['d__Bacteria', 'p__Chloroflexota', 'c__Anaerolineae', 'o__Caldilineales', 'f__Caldilineaceae', 'g__Bin5', 's__'], 'COS36388_bin_6': ['d__Bacteria', 'p__Chloroflexota', 'c__Anaerolineae', 'o__SBR1031', 'f__A4b', 'g__UBA6055', 's__'], 'COS3_bin_18': ['d__Bacteria', 'p__Chloroflexota', 'c__Dehalococcoidia', 'o__Bin125', 'f__Bin125', 'g__Bin125', 's__'], 'IRC3_bin_37': ['d__Bacteria', 'p__Gemmatimonadota', 'c__Gemmatimonadetes', 'o__SG8-23', 'f__UBA6960', 'g__BD2-11', 's__'], 'CAR2_bin_4': ['d__Bacteria', 'p__Cyanobacteria', 'c__Cyanobacteriia', 'o__Synechococcales', 'f__Cyanobiaceae', 'g__Synechococcus_B', 's__'], 'IRC_PAM_SB0670_bin_39': ['d__Bacteria', 'p__Acidobacteriota', 'c__Thermoanaerobaculia', 'o__UBA5704', 'f__', 'g__', 's__'], 'COS1_bin_6': ['d__Bacteria', 'p__Actinobacteriota', 'c__Acidimicrobiia', 'o__Microtrichales', 'f__Bin134', 'g__Bin134', 's__'], 'RHO3_bin_2': ['d__Bacteria', 'p__Chloroflexota', 'c__Dehalococcoidia', 'o__UBA3495', 'f__UBA3495', 'g__Bin22', 's__'], 'APA_bin_7': ['d__Bacteria', 'p__Actinobacteriota', 'c__Acidimicrobiia', 'o__Microtrichales', 'f__UBA11606', 'g__', 's__'], 'STY2_bin_4': ['d__Archaea', 'p__Crenarchaeota', 'c__Nitrososphaeria', 'o__Nitrososphaerales', 'f__Nitrosopumilaceae', 'g__Cenarchaeum', 's__'], 'IRC1_bin_10': ['d__Bacteria', 'p__Chloroflexota', 'c__Anaerolineae', 'o__SBR1031', 'f__A4b', 'g__UBA6055', 's__'], 'RHO1_bin_32': ['d__Bacteria', 'p__Chloroflexota_B', 'c__UBA11872', 'o__UBA11872', 'f__UBA11872', 'g__', 's__'], 'COS36386_bin_4': ['d__Bacteria', 'p__Chloroflexota', 'c__Anaerolineae', 'o__SBR1031', 'f__A4b', 'g__UBA6055', 's__'], 'COS36387_bin_3': ['d__Bacteria', 'p__Acidobacteriota', 'c__Thermoanaerobaculia', 'o__UBA5704', 'f__', 'g__', 's__'], 'RHO3_bin_65': ['d__Bacteria', 'p__Binatota', 'c__Binatia', 'o__UBA9968', 'f__', 'g__', 's__'], 'COS1_bin_13': ['d__Bacteria', 'p__Acidobacteriota', 'c__Thermoanaerobaculia', 'o__UBA5704', 'f__', 'g__', 's__'], 'IRC_PAM_SB0662_bin_15': ['d__Bacteria', 'p__Latescibacterota', 'c__UBA2968', 'o__UBA2968', 'f__', 'g__', 's__'], 'GCF_900143615': ['d__Bacteria', 'p__Proteobacteria', 'c__Alphaproteobacteria', 'o__Rhodobacterales', 'f__Rhodobacteraceae', 'g__Shimia', 's__Shimia sp900143615'], 'IRC_PAM_SB0661_bin_32': ['d__Bacteria', 'p__Chloroflexota', 'c__Anaerolineae', 'o__Caldilineales', 'f__Caldilineaceae', 'g__Bin5', 's__'], 'COS36386_bin_3': ['d__Bacteria', 'p__Proteobacteria', 'c__Gammaproteobacteria', 'o__UBA10353', 'f__LS-SOB', 'g__LS-SOB', 's__'], 'RHO2_bin_31': ['d__Bacteria', 'p__Acidobacteriota', 'c__Acidobacteriae', 'o__Bryobacterales', 'f__UBA6623', 'g__', 's__'], 'seawater_seasim_SB9152_S1_bin_18': ['d__Bacteria', 'p__Proteobacteria', 'c__Alphaproteobacteria', 'o__Rhodobacterales', 'f__Rhodobacteraceae', 'g__Boseongicola', 's__'], 'COS36404_bin_12': ['d__Bacteria', 'p__Planctomycetota', 'c__Planctomycetes', 'o__Pirellulales', 'f__UBA1268', 'g__', 's__'], 'seawater_seasim_SB9152_S1_bin_19': ['d__Bacteria', 'p__Bdellovibrionota_B', 'c__Oligoflexia', 'o__Oligoflexales', 'f__', 'g__', 's__'], 'seawater_seasim_SB9157_S6_bin_7': ['d__Bacteria', 'p__Proteobacteria', 'c__Gammaproteobacteria', 'o__Pseudomonadales', 'f__Pseudohongiellaceae', 'g__UBA9145', 's__'], 'COS36406_bin_18': ['d__Bacteria', 'p__Binatota', 'c__Binatia', 'o__Bin18', 'f__Bin18', 'g__Bin18', 's__'], 'COS1_bin_5': ['d__Bacteria', 'p__Actinobacteriota', 'c__Acidimicrobiia', 'o__Microtrichales', 'f__Bin134', 'g__', 's__'], 'STY4_bin_1': ['d__Bacteria', 'p__Proteobacteria', 'c__Gammaproteobacteria', 'o__UBA10353', 'f__LS-SOB', 'g__', 's__'], 'APA_bin_4': ['d__Bacteria', 'p__Actinobacteriota', 'c__Acidimicrobiia', 'o__Microtrichales', 'f__TK06', 'g__', 's__'], 'COS36388_bin_17': ['d__Bacteria', 'p__Chloroflexota', 'c__Anaerolineae', 'o__SBR1031', 'f__A4b', 'g__UBA6055', 's__'], 'APA_bin_59': ['d__Bacteria', 'p__Binatota', 'c__Binatia', 'o__UBA9968', 'f__', 'g__', 's__'], 'COS36387_bin_9': ['d__Bacteria', 'p__Chloroflexota', 'c__Anaerolineae', 'o__SBR1031', 'f__A4b', 'g__UBA6055', 's__'], 'COS36387_bin_19': ['d__Bacteria', 'p__Chloroflexota', 'c__Anaerolineae', 'o__SBR1031', 'f__A4b', 'g__UBA6055', 's__'], 'RHO1_bin_23': ['d__Bacteria', 'p__Dadabacteria', 'c__UBA1144', 'o__', 'f__', 'g__', 's__'], 'IRC_PAM_SB0668_bin_1': ['d__Bacteria', 'p__Bacteroidota', 'c__Rhodothermia', 'o__Rhodothermales', 'f__', 'g__', 's__'], 'COS36387_bin_6': ['d__Bacteria', 'p__Acidobacteriota', 'c__Thermoanaerobaculia', 'o__UBA5704', 'f__', 'g__', 's__'], 'seawater_22112_bin_15': ['d__Bacteria', 'p__Proteobacteria', 'c__Gammaproteobacteria', 'o__Pseudomonadales', 'f__Moraxellaceae', 'g__Psychrobacter', 's__'], 'APA_bin_18': ['d__Bacteria', 'p__Actinobacteriota', 'c__Acidimicrobiia', 'o__Microtrichales', 'f__Bin134', 'g__', 's__'], 'COS1_bin_3': ['d__Bacteria', 'p__Chloroflexota_B', 'c__UBA11872', 'o__UBA11872', 'f__UBA11872', 'g__', 's__'], 'IRC3_bin_9': ['d__Bacteria', 'p__Actinobacteriota', 'c__Acidimicrobiia', 'o__Microtrichales', 'f__TK06', 'g__', 's__'], 'seawater_seasim_SB9155_S4_bin_5': ['d__Bacteria', 'p__Gemmatimonadota', 'c__Gemmatimonadetes', 'o__SG8-23', 'f__UBA6960', 'g__UBA2589', 's__'], 'IRC_PAM_SB0662_bin_22': ['d__Bacteria', 'p__Acidobacteriota', 'c__Thermoanaerobaculia', 'o__UBA5704', 'f__', 'g__', 's__'], 'RHO1_bin_45': ['d__Bacteria', 'p__Spirochaetota', 'c__Spirochaetia', 'o__Spirochaetales', 'f__RBG-16-67-19', 'g__', 's__'], 'CAR2_bin_3': ['d__Bacteria', 'p__Chloroflexota', 'c__Anaerolineae', 'o__SBR1031', 'f__A4b', 'g__UBA6055', 's__'], 'CAR1_bin_18': ['d__Bacteria', 'p__Proteobacteria', 'c__Alphaproteobacteria', 'o__Rhodobacterales', 'f__Rhodobacteraceae', 'g__Litoreibacter', 's__'], 'seawater_bettina_36309_bin_17': ['d__Bacteria', 'p__Proteobacteria', 'c__Alphaproteobacteria', 'o__Puniceispirillales', 'f__Puniceispirillaceae', 'g__UBA685', 's__'], 'IRC_PAM_SB0665_bin_11': ['d__Bacteria', 'p__Chloroflexota', 'c__Dehalococcoidia', 'o__Bin125', 'f__Bin125', 'g__Bin125', 's__'], 'RHO2_bin_43': ['d__Bacteria', 'p__Chloroflexota_B', 'c__UBA11872', 'o__UBA11872', 'f__UBA11872', 'g__', 's__'], 'RHO3_bin_80': ['d__Bacteria', 'p__Proteobacteria', 'c__Alphaproteobacteria', 'o__UBA828', 'f__UBA828', 'g__', 's__'], 'IRC_PAM_SB0665_bin_17': ['d__Bacteria', 'p__Actinobacteriota', 'c__Acidimicrobiia', 'o__UBA5794', 'f__SZUA-232', 'g__', 's__'], 'IRC_PAM_SB0664_bin_4': ['d__Bacteria', 'p__Actinobacteriota', 'c__Acidimicrobiia', 'o__Microtrichales', 'f__UBA11606', 'g__', 's__'], 'RHO1_bin_8': ['d__Bacteria', 'p__Chloroflexota', 'c__Dehalococcoidia', 'o__UBA2963', 'f__UBA2963', 'g__Bin16', 's__'], 'IRC_PAM_SB0668_bin_7': ['d__Bacteria', 'p__Actinobacteriota', 'c__Acidimicrobiia', 'o__Microtrichales', 'f__TK06', 'g__', 's__'], 'CAR2_bin_5': ['d__Bacteria', 'p__Proteobacteria', 'c__Alphaproteobacteria', 'o__Bin65', 'f__Bin65', 'g__', 's__'], 'COS36406_bin_1': ['d__Bacteria', 'p__Chloroflexota', 'c__Anaerolineae', 'o__Caldilineales', 'f__Caldilineaceae', 'g__Bin5', 's__'], 'APA_bin_43': ['d__Bacteria', 'p__Chloroflexota', 'c__Dehalococcoidia', 'o__UBA3495', 'f__UBA3495', 'g__Bin87', 's__Bin87 sp002238825'], 'COS36386_bin_20': ['d__Bacteria', 'p__Proteobacteria', 'c__Gammaproteobacteria', 'o__UBA4575', 'f__UBA4575', 'g__', 's__'], 'CAR1_bin_14': ['d__Bacteria', 'p__Proteobacteria', 'c__Gammaproteobacteria', 'o__UBA4486', 'f__UBA4486', 'g__', 's__'], 'IRC_PAM_SB0666_bin_11': ['d__Bacteria', 'p__Chloroflexota', 'c__Dehalococcoidia', 'o__UBA2991', 'f__UBA2991', 'g__UBA2991', 's__'], 'IRC1_bin_15': ['d__Bacteria', 'p__Chloroflexota', 'c__Anaerolineae', 'o__Caldilineales', 'f__Caldilineaceae', 'g__Bin5', 's__'], 'IRC3_bin_7': ['d__Bacteria', 'p__Proteobacteria', 'c__Alphaproteobacteria', 'o__Bin65', 'f__Bin65', 'g__Bin65', 's__'], 'STY4_bin_8': ['d__Bacteria', 'p__Proteobacteria', 'c__Gammaproteobacteria', 'o__UBA10353', 'f__LS-SOB', 'g__', 's__'], 'APA_bin_33': ['d__Bacteria', 'p__Chloroflexota', 'c__Dehalococcoidia', 'o__SAR202', 'f__UBA11138', 'g__Bin90', 's__Bin90 sp002238855'], 'IRC_PAM_SB0662_bin_24': ['d__Bacteria', 'p__Bacteroidota', 'c__Rhodothermia', 'o__Rhodothermales', 'f__Bin80', 'g__Bin80', 's__'], 'seawater_seasim_SB9160_S9_bin_11': ['d__Bacteria', 'p__Gemmatimonadota', 'c__Gemmatimonadetes', 'o__SG8-23', 'f__UBA6960', 'g__UBA2589', 's__'], 'seawater_bettina_36326_bin_7': ['d__Bacteria', 'p__Bacteroidota', 'c__Bacteroidia', 'o__Flavobacteriales', 'f__Cryomorphaceae', 'g__UBA10364', 's__'], 'seawater_22112_bin_7': ['d__Bacteria', 'p__Bacteroidota', 'c__Bacteroidia', 'o__Flavobacteriales', 'f__Flavobacteriaceae', 'g__Flavobacterium', 's__'], 'RHO2_bin_51': ['d__Bacteria', 'p__Gemmatimonadota', 'c__Gemmatimonadetes', 'o__SG8-23', 'f__', 'g__', 's__'], 'RHO1_bin_40': ['d__Bacteria', 'p__Chloroflexota', 'c__Anaerolineae', 'o__Caldilineales', 'f__Caldilineaceae', 'g__Bin5', 's__'], 'IRC4_bin_14': ['d__Bacteria', 'p__Poribacteria', 'c__WGA-4E', 'o__WGA-4E', 'f__WGA-3G', 'g__', 's__'], 'seawater_22112_bin_12': ['d__Bacteria', 'p__Proteobacteria', 'c__Gammaproteobacteria', 'o__Enterobacterales', 'f__Alteromonadaceae', 'g__Pseudoalteromonas', 's__Pseudoalteromonas shioyasakiensis'], 'RHO1_bin_65': ['d__Bacteria', 'p__Actinobacteriota', 'c__Acidimicrobiia', 'o__UBA5794', 'f__SZUA-232', 'g__', 's__'], 'CLI4_bin_1': ['d__Bacteria', 'p__Verrucomicrobiota_A', 'c__Chlamydiia', 'o__Parachlamydiales', 'f__Simkaniaceae', 'g__', 's__'], 'IRC_PAM_SB0672_bin_11': ['d__Bacteria', 'p__Acidobacteriota', 'c__Bin61', 'o__Bin61', 'f__Bin61', 'g__Bin61', 's__'], 'IRC_PAM_SB0661_bin_8': ['d__Bacteria', 'p__Acidobacteriota', 'c__Bin61', 'o__Bin61', 'f__Bin61', 'g__Bin61', 's__'], 'RHO2_bin_56': ['d__Bacteria', 'p__Actinobacteriota', 'c__Acidimicrobiia', 'o__UBA5794', 'f__Bin76', 'g__Bin76', 's__'], 'RHO2_bin_26': ['d__Bacteria', 'p__Actinobacteriota', 'c__Acidimicrobiia', 'o__Microtrichales', 'f__UBA11606', 'g__', 's__'], 'IRC1_bin_18': ['d__Bacteria', 'p__Chloroflexota_B', 'c__UBA11872', 'o__UBA11872', 'f__', 'g__', 's__'], 'APA_bin_40': ['d__Bacteria', 'p__Proteobacteria', 'c__Alphaproteobacteria', 'o__Rhodobacterales', 'f__Rhodobacteraceae', 'g__Bin36', 's__'], 'RHO2_bin_52': ['d__Bacteria', 'p__Gemmatimonadota', 'c__Gemmatimonadetes', 'o__SG8-23', 'f__UBA6960', 'g__Bin94', 's__'], 'IRC_PAM_SB0662_bin_30': ['d__Bacteria', 'p__Actinobacteriota', 'c__Acidimicrobiia', 'o__Microtrichales', 'f__Bin134', 'g__Bin134', 's__'], 'GCF_000264395': ['d__Bacteria', 'p__Firmicutes', 'c__Bacilli', 'o__Bacillales', 'f__Bacillaceae', 'g__Bacillus', 's__Bacillus atrophaeus'], 'COS4_bin_44': ['d__Bacteria', 'p__Proteobacteria', 'c__Gammaproteobacteria', 'o__Pseudomonadales', 'f__HTCC2089', 'g__', 's__'], 'IRC_PAM_SB0664_bin_6': ['d__Bacteria', 'p__Chloroflexota', 'c__Dehalococcoidia', 'o__UBA1151', 'f__Bin127', 'g__Bin127', 's__'], 'CLI4_bin_2': ['d__Bacteria', 'p__Dadabacteria', 'c__UBA1144', 'o__', 'f__', 'g__', 's__'], 'seawater_22112_bin_8': ['d__Bacteria', 'p__Proteobacteria', 'c__Gammaproteobacteria', 'o__Burkholderiales', 'f__Burkholderiaceae', 'g__Limnobacter', 's__Limnobacter sp000170915'], 'GCA_003635205': ['d__Bacteria', 'p__Poribacteria', 'c__WGA-4E', 'o__WGA-4E', 'f__WGA-3G', 'g__WGA-3G', 's__'], 'CAR3_bin_15': ['d__Bacteria', 'p__Proteobacteria', 'c__Gammaproteobacteria', 'o__Pseudomonadales', 'f__HTCC2089', 'g__UBA2168', 's__'], 'seawater_42618_bin_3': ['d__Bacteria', 'p__Proteobacteria', 'c__Gammaproteobacteria', 'o__Pseudomonadales', 'f__HTCC2089', 'g__UBA4421', 's__'], 'RHO3_bin_28': ['d__Bacteria', 'p__Chloroflexota_B', 'c__UBA11872', 'o__UBA11872', 'f__', 'g__', 's__'], 'COS36404_bin_1': ['d__Bacteria', 'p__Planctomycetota', 'c__Planctomycetes', 'o__Pirellulales', 'f__UBA1268', 'g__UBA1268', 's__UBA1268 sp002694955'], 'seawater_bettina_36328_bin_30': ['d__Bacteria', 'p__Proteobacteria', 'c__Alphaproteobacteria', 'o__Rhodobacterales', 'f__Rhodobacteraceae', 'g__HIMB11', 's__HIMB11 sp001627375'], 'IRC3_bin_6': ['d__Bacteria', 'p__Chloroflexota_B', 'c__UBA11872', 'o__UBA11872', 'f__', 'g__', 's__'], 'CAR1_bin_16': ['d__Bacteria', 'p__Bacteroidota', 'c__Rhodothermia', 'o__Rhodothermales', 'f__Bin80', 'g__Bin80', 's__'], 'APA_bin_27': ['d__Bacteria', 'p__Proteobacteria', 'c__Alphaproteobacteria', 'o__Nisaeales', 'f__Nisaeaceae', 'g__Bin52', 's__Bin52 sp002238645'], 'COS36388_bin_3': ['d__Bacteria', 'p__Nitrospirota', 'c__Nitrospiria', 'o__Nitrospirales', 'f__UBA8639', 'g__Bin75', 's__'], 'APA_bin_23': ['d__Bacteria', 'p__Acidobacteriota', 'c__Vicinamibacteria', 'o__Vicinamibacterales', 'f__UBA8438', 'g__', 's__'], 'RHO3_bin_13': ['d__Bacteria', 'p__Actinobacteriota', 'c__Acidimicrobiia', 'o__UBA5794', 'f__SZUA-232', 'g__', 's__'], 'IRC1_bin_14': ['d__Bacteria', 'p__Gemmatimonadota', 'c__Gemmatimonadetes', 'o__SG8-23', 'f__UBA6960', 'g__BD2-11', 's__'], 'APA_bin_87': ['d__Bacteria', 'p__Proteobacteria', 'c__Alphaproteobacteria', 'o__Bin65', 'f__Bin65', 'g__Bin65', 's__Bin65 sp002238725'], 'GCF_900143635': ['d__Bacteria', 'p__Proteobacteria', 'c__Alphaproteobacteria', 'o__Rhodobacterales', 'f__Rhodobacteraceae', 'g__FREY01', 's__FREY01 sp900143635'], 'IRC_PAM_SB0664_bin_27': ['d__Bacteria', 'p__Chloroflexota', 'c__Anaerolineae', 'o__Caldilineales', 'f__Caldilineaceae', 'g__Bin5', 's__'], 'COS36386_bin_14': ['d__Bacteria', 'p__Actinobacteriota', 'c__Acidimicrobiia', 'o__Microtrichales', 'f__TK06', 'g__', 's__'], 'APA_bin_39': ['d__Bacteria', 'p__Gemmatimonadota', 'c__Gemmatimonadetes', 'o__SG8-23', 'f__UBA6960', 'g__Bin94', 's__'], 'IRC4_bin_25': ['d__Bacteria', 'p__Poribacteria', 'c__WGA-4E', 'o__WGA-4E', 'f__WGA-3G', 'g__WGA-3G', 's__'], 'COS2_bin_6': ['d__Bacteria', 'p__Actinobacteriota', 'c__Acidimicrobiia', 'o__Microtrichales', 'f__Bin134', 'g__', 's__'], 'RHO1_bin_20': ['d__Bacteria', 'p__Chloroflexota', 'c__Dehalococcoidia', 'o__Bin125', 'f__Bin125', 'g__Bin125', 's__'], 'seawater_seasim_SB9156_S5_bin_4': ['d__Bacteria', 'p__Marinisomatota', 'c__Marinisomatia', 'o__Marinisomatales', 'f__TCS55', 'g__TCS55', 's__TCS55 sp001577025'], 'IRC4_bin_45': ['d__Bacteria', 'p__Latescibacterota', 'c__UBA2968', 'o__UBA8231', 'f__GCA-002724215', 'g__GCA-2724215', 's__'], 'IRC_PAM_SB0664_bin_3': ['d__Bacteria', 'p__Acidobacteriota', 'c__Vicinamibacteria', 'o__Vicinamibacterales', 'f__UBA8438', 'g__', 's__'], 'IRC1_bin_34': ['d__Bacteria', 'p__Actinobacteriota', 'c__Acidimicrobiia', 'o__Microtrichales', 'f__UBA11606', 'g__', 's__'], 'IRC_PAM_SB0661_bin_26': ['d__Bacteria', 'p__Actinobacteriota', 'c__Acidimicrobiia', 'o__Microtrichales', 'f__UBA11606', 'g__UBA11606', 's__'], 'seawater_bettina_36308_bin_5': ['d__Bacteria', 'p__Proteobacteria', 'c__Alphaproteobacteria', 'o__Puniceispirillales', 'f__Puniceispirillaceae', 'g__', 's__'], 'CAR2_bin_14': ['d__Bacteria', 'p__Proteobacteria', 'c__Gammaproteobacteria', 'o__HK1', 'f__HK1', 'g__', 's__'], 'RHO2_bin_8': ['d__Bacteria', 'p__Chloroflexota', 'c__Anaerolineae', 'o__SBR1031', 'f__A4b', 'g__UBA6055', 's__'], 'CAR1_bin_4': ['d__Bacteria', 'p__Proteobacteria', 'c__Alphaproteobacteria', 'o__Rhodobacterales', 'f__Rhodobacteraceae', 'g__Roseovarius', 's__'], 'COS36405_bin_11': ['d__Bacteria', 'p__Proteobacteria', 'c__Alphaproteobacteria', 'o__UBA828', 'f__UBA828', 'g__', 's__'], 'COS4_bin_7': ['d__Bacteria', 'p__Proteobacteria', 'c__Alphaproteobacteria', 'o__Rhodobacterales', 'f__Rhodobacteraceae', 'g__Bin36', 's__'], 'GCF_001941685': ['d__Bacteria', 'p__Proteobacteria', 'c__Alphaproteobacteria', 'o__Rhizobiales', 'f__Stappiaceae', 'g__Pseudovibrio', 's__Pseudovibrio sp001941685'], 'CAR2_bin_11': ['d__Bacteria', 'p__Proteobacteria', 'c__Gammaproteobacteria', 'o__UBA4486', 'f__UBA4486', 'g__', 's__'], 'IRC_PAM_SB0677_bin_16': ['d__Archaea', 'p__Crenarchaeota', 'c__Nitrososphaeria', 'o__Nitrososphaerales', 'f__Nitrosopumilaceae', 'g__Cenarchaeum', 's__'], 'seawater_seasim_SB9152_S1_bin_8': ['d__Bacteria', 'p__Proteobacteria', 'c__Alphaproteobacteria', 'o__UBA8366', 'f__GCA-2717185', 'g__GCA-2717185', 's__'], 'COS36386_bin_10': ['d__Bacteria', 'p__Planctomycetota', 'c__Planctomycetes', 'o__Pirellulales', 'f__Pirellulaceae', 'g__Mariniblastus', 's__'], 'IRC2_bin_1': ['d__Bacteria', 'p__Bacteroidota', 'c__Rhodothermia', 'o__Rhodothermales', 'f__Bin80', 'g__Bin80', 's__'], 'RHO3_bin_22': ['d__Bacteria', 'p__Chloroflexota_B', 'c__UBA11872', 'o__', 'f__', 'g__', 's__'], 'aplysina_bin': ['d__Bacteria', 'p__Latescibacterota', 'c__UBA2968', 'o__UBA8231', 'f__GCA-002724215', 'g__GCA-2724215', 's__'], 'RHO3_bin_71': ['d__Bacteria', 'p__Gemmatimonadota', 'c__Gemmatimonadetes', 'o__SG8-23', 'f__UBA6960', 'g__Bin94', 's__'], 'seawater_bettina_36327_bin_2': ['d__Bacteria', 'p__Cyanobacteria', 'c__Cyanobacteriia', 'o__Synechococcales', 'f__Cyanobiaceae', 'g__Vulcanococcus', 's__'], 'RHO2_bin_20': ['d__Bacteria', 'p__Poribacteria', 'c__WGA-4E', 'o__WGA-4E', 'f__WGA-3G', 'g__', 's__'], 'IRC4_bin_29': ['d__Bacteria', 'p__Bacteroidota', 'c__Bacteroidia', 'o__Cytophagales', 'f__Cyclobacteriaceae', 'g__Ekhidna', 's__'], 'RHO3_bin_64': ['d__Bacteria', 'p__Binatota', 'c__Binatia', 'o__UBA9968', 'f__', 'g__', 's__'], 'CAR1_bin_3': ['d__Bacteria', 'p__Proteobacteria', 'c__Alphaproteobacteria', 'o__Bin95', 'f__Bin95', 'g__Bin95', 's__'], 'APA_bin_61': ['d__Bacteria', 'p__Proteobacteria', 'c__Gammaproteobacteria', 'o__Xanthomonadales', 'f__Xanthomonadaceae', 'g__Stenotrophomonas', 's__Stenotrophomonas maltophilia_F'], 'RHO3_bin_7': ['d__Bacteria', 'p__Latescibacterota', 'c__UBA2968', 'o__UBA2968', 'f__GCA-2709665', 'g__', 's__'], 'COS4_bin_10': ['d__Bacteria', 'p__Chloroflexota', 'c__Dehalococcoidia', 'o__UBA2991', 'f__UBA2991', 'g__UBA2991', 's__'], 'COS36386_bin_11': ['d__Bacteria', 'p__Planctomycetota', 'c__Planctomycetes', 'o__Pirellulales', 'f__Pirellulaceae', 'g__Rubripirellula', 's__'], 'GCF_900143555': ['d__Bacteria', 'p__Proteobacteria', 'c__Alphaproteobacteria', 'o__Rhizobiales', 'f__Stappiaceae', 'g__Labrenzia', 's__Labrenzia alba']}

# get all identified taxon at defined ranks

rank_to_position_dict = {'d': 0, 'p': 1, 'c': 2, 'o': 3, 'f': 4, 'g': 5, 's': 6}

specified_rank_pos = rank_to_position_dict['p']

identified_taxon_list = []

for each_TaxonAssign in taxon_assignment_dict:

specified_rank_id = taxon_assignment_dict[each_TaxonAssign][specified_rank_pos]

if specified_rank_id not in identified_taxon_list:

identified_taxon_list.append(specified_rank_id)

# get the id of genomes assigned to each taxon at specified level

taxon_2_genome_dict = {}

for each_taxon in identified_taxon_list:

genome_list = []

for genome in taxon_assignment_dict:

if taxon_assignment_dict[genome][specified_rank_pos] == each_taxon:

genome_list.append(genome)

taxon_2_genome_dict[each_taxon] = genome_list

# get genome_2_taxon_dict

genome_2_taxon_dict = {}

for taxon in taxon_2_genome_dict:

current_taxon_genome_list = taxon_2_genome_dict[taxon]

for genome in current_taxon_genome_list:

genome_2_taxon_dict[genome] = taxon

########################################################################################################################

# get genome to sponge-taxon dict

genome_2_SpongeTaxon_dict = {}

for genome in genome_2_taxon_dict:

genome_raw_name = new2raw_name_dict[genome]

genome_taxon = genome_2_taxon_dict[genome]

genome_source = bin_to_source2_dict[genome_raw_name]

genome_source = genome_source.replace(" ", "_")

genome_SpongeTaxon = '%s%s%s' % (genome_source[:2], '1', genome_taxon)

genome_2_SpongeTaxon_dict[genome] = genome_SpongeTaxon

Get_circlize_plot(detected_HGTs_pcofg, genome_2_SpongeTaxon_dict, 'Figure_S8.txt', 'p', bin_to_source2_dict, sponge_with_high_HGT_preferences_list)

# get plot with R

os.system('Rscript circos_HGT_Figure_S8.R -m Figure_S8.txt -p Figure_S8.pdf -s 1')

**End script Figure_S8.py**

**Start R script circus_HGT_Figure_S8.R**

#!/usr/bin/env Rscript

####################################### define functions #######################################

check.packages <- function(pkg){

new.pkg <- pkg[!(pkg %in% installed.packages()[, "Package"])]

if (length(new.pkg))

install.packages(new.pkg, dependencies = TRUE)

sapply(pkg, require, character.only = 1)

}

split_list_element = function(list_in, sep_symbol){

list_in_split = strsplit(list_in, sep_symbol)

group_list = c()

taxon_list = c()

for (element in list_in_split){

group_list = c(group_list, element[1])

taxon_list = c(taxon_list, element[2])

}

return(c(group_list, taxon_list))

}

get_group_list = function(list_in, sep_symbol){

list_in_split = strsplit(list_in, sep_symbol)

group_list = c()

for (element in list_in_split){

group_list = c(group_list, element[1])

}

return(group_list)

}

get_taxon_list = function(list_in, sep_symbol){

list_in_split = strsplit(list_in, sep_symbol)

taxon_list = c()

for (element in list_in_split){

taxon_list = c(taxon_list, element[2])

}

return(taxon_list)

}

remove_zero_rows_cols = function(matrix_in){

remove_zero_rows = function(matrix_in){

row_sum = rowSums(matrix_in)

mat_with_row_sum = cbind(matrix_in, row_sum)

mat_non_zero_row_tmp = mat_with_row_sum[mat_with_row_sum$row_sum > 0, ]

mat_non_zero_row = subset(mat_non_zero_row_tmp, select = -c(row_sum))

return(mat_non_zero_row)

}

mat_non_zero_row = remove_zero_rows(mat)

mat_non_zero_row_t = as.data.frame(t(mat_non_zero_row))

mat_non_zero_row_col_t = remove_zero_rows(mat_non_zero_row_t)

mat_non_zero_row_col = t(mat_non_zero_row_col_t)

return(mat_non_zero_row_col)

}

####################################### install packages #######################################

packages<-c("optparse", "circlize")

invisible(suppressMessages(check.packages(packages)))

####################################### argument parser ######################################

options(warn=-1)

option_list = list(

make_option(c("-m", "--matrix"), type="character", help="input matrix"),

make_option(c("-s", "--sep"), type="character", help="label separator"),

make_option(c("-p", "--plot"), type="character", help="output plot"));

within_group_gap = 1

between_group_gap = 3

opt_parser = OptionParser(option_list=option_list);

opt = parse_args(opt_parser);

group_separator = opt$sep

# reads in file

mat = read.table(opt$matrix, header = TRUE)

######################################## prepare plot ########################################

#png(filename=opt$plot, units="in", width=25, height=25, pointsize=12, res=150)

pdf(opt$plot, width=15, height=15, pointsize=12)

grid.col = c(A = 'brown1', B = 'lawngreen', C = 'mediumorchid', D = 'mediumslateblue', E = 'royalblue', F = 'sandybrown')

par(mar = rep(0,4), cex = 1.2)

# set label_order

label_order = sort(union(rownames(mat), colnames(mat)))

############################### Set larger gaps between groups ###############################

# Set larger gaps between groups

label_order_on_plot = sort(union(rownames(remove_zero_rows_cols(mat)), colnames(remove_zero_rows_cols(mat))))

group_list_on_plot = get_group_list(label_order_on_plot, group_separator)

taxon_list_on_plot = get_taxon_list(label_order_on_plot, group_separator)

gap_between_group_list = c()

last_group = 'None'

for(group_id in group_list_on_plot){

if (last_group == 'None'){

last_group = group_id

} else if (group_id == last_group){

gap_between_group_list = c(gap_between_group_list, within_group_gap)

} else if (group_id != last_group){

gap_between_group_list = c(gap_between_group_list, between_group_gap)

last_group = group_id

}

}

if (group_list_on_plot[[1]] == group_list_on_plot[[length(group_list_on_plot)]]){

gap_between_group_list = c(gap_between_group_list, within_group_gap)

} else {

gap_between_group_list = c(gap_between_group_list, between_group_gap)

}

# Set larger gaps between groups

circos.par(gap.after = gap_between_group_list)

################################## set same color for same taxon ##################################

taxon_list_on_plot_uniq = unique(taxon_list_on_plot)

track_color_list = rainbow(length(taxon_list_on_plot_uniq))

# get color for each taxon

n = 1

taxon_to_color_dict = list()

for (each_taxon in taxon_list_on_plot_uniq){

taxon_to_color_dict[each_taxon] = track_color_list[n]

n = n + 1

}

# set color for all labels

label_color = c()

for (each_label in label_order_on_plot){

each_label_split = strsplit(each_label, group_separator)

each_label_color = taxon_to_color_dict[each_label_split[[1]][2]][[1]]

label_color[each_label] = each_label_color

}

######################################## plot chordDiagram ########################################

# plot chordDiagram

chordDiagram(t(mat), order = label_order, annotationTrack = "grid", annotationTrackHeight = c(0.03, 0.03), preAllocateTracks = 1, grid.col = label_color)

######################################## add group track ########################################

color_list = list(Ap = '#61c2c2',

Ca = '#7ecf65',

Co = '#b66dbd',

Ir = '#CCCC00',

Rh = '#FFB266')

current_group = 'None'

current_group_member = c()

for (label in label_order_on_plot){

label_split = strsplit(label, group_separator)

label_group = label_split[[1]][1]

if (current_group == 'None'){

current_group = label_group

current_group_member = c(current_group_member, label)

} else if (label_group == current_group){

current_group_member = c(current_group_member, label)

} else if (label_group != current_group){

highlight.sector(current_group_member, track.index = 1, col = color_list[[current_group]], padding=c(-0.05, 0, 0.2, 0), niceFacing = TRUE)

current_group = label_group

current_group_member = c(label)

}

}

print(current_group)

highlight.sector(current_group_member, track.index = 1, col = color_list[[current_group]], padding=c(-0.05, 0, 0.2, 0), niceFacing = TRUE)

######################################## rotate label ########################################

circos.trackPlotRegion(track.index = 1, panel.fun = function(x, y) {

xlim = get.cell.meta.data("xlim")

ylim = get.cell.meta.data("ylim")

sector.name = get.cell.meta.data("sector.index")

label_text = strsplit(sector.name, 'p__')[[1]][2]

circos.text(mean(xlim), ylim[1] + .1, label_text, facing = "clockwise", niceFacing = TRUE, adj = c(0, 0.5), cex = 0.8 )

circos.axis(h = "top", labels.cex = 0.7, major.tick.percentage = 0.2, sector.index = sector.name, track.index = 2)

}, bg.border = NA)

######################################## clear variables ########################################

#circos.track(circos.text, facing = "clockwise")

invisible(dev.off())

circos.clear()

rm(list=ls())

**End R script Figure_S8.py**

**Start python script Figure_S9_S10.py**

#!/usr/bin/env python3

import statistics

import numpy as np

from scipy import stats

import matplotlib as mpl

mpl.use('Agg')

import matplotlib.pyplot as plt

import os

import shutil

def force_create_folder(folder_to_create):

if os.path.isdir(folder_to_create):

shutil.rmtree(folder_to_create, ignore_errors=True)

os.mkdir(folder_to_create)

def plot_donor_to_recipient_HGT_num(lol_in, sponge_type_list_sorted, plot_out):

# turn list of list into arrary

donor_to_recipient_HGT_num_arrary = np.array(lol_in)

fig, ax = plt.subplots()

im = ax.imshow(donor_to_recipient_HGT_num_arrary, interpolation='nearest')

# show all ticks

ax.set_xticks(np.arange(len(sponge_type_list_sorted)))

ax.set_yticks(np.arange(len(sponge_type_list_sorted)))

# label ticks

ax.set_xticklabels(sponge_type_list_sorted, fontsize=7, fontstyle='italic')

ax.set_yticklabels(sponge_type_list_sorted, fontsize=7, fontstyle='italic')

# Rotate the tick labels and set their alignment.

plt.setp(ax.get_xticklabels(), rotation=45, ha="right", rotation_mode="anchor")

# Loop over data dimensions and create text annotations.

for i in range(len(sponge_type_list_sorted)):

for j in range(len(sponge_type_list_sorted)):

text = ax.text(j, i, donor_to_recipient_HGT_num_arrary[i, j],

ha="center", va="center", color="w", fontsize=8)

# Create colorbar

cbar = ax.figure.colorbar(im, ax=ax)

# save the plot

# ax.set_title("Harvest of local farmers (in tons/year)")

fig.tight_layout()

plt.savefig(plot_out, bbox_inches='tight', dpi=600)

plt.close()

plt.clf()

def box_plotter(num_list_in, label_list, plot_title, x_axis_label, y_axis_label, output_plot):

# turn num list into arrary

MAG_HGT_num_lol_arrary = [np.array(i) for i in num_list_in]

# get plot

fig = plt.figure(1, figsize=(9, 6))

ax = fig.add_subplot(111)

bp = ax.boxplot(MAG_HGT_num_lol_arrary,

showfliers=False,

patch_artist=True,

whiskerprops=dict(color='lightblue', linewidth=2),

capprops=dict(color='lightblue'))

# set the color pf box

for box in bp['boxes']:

box.set(linewidth=0)

box.set_facecolor('lightblue')

# set x tick labels

if len(label_list) <= 5:

rotation_value = 0

else:

rotation_value = 270

ax.set_xticklabels(label_list, rotation=rotation_value, fontsize=8)

# set title, x and y label

plt.title(plot_title)

plt.xlabel(x_axis_label)

plt.ylabel(y_axis_label)

## change the style of fliers and their fill

for flier in bp['fliers']:

flier.set(marker='+', color='black', alpha=0.7, markersize=3)

plt.tight_layout()

fig.savefig(output_plot, bbox_inches='tight', dpi=300)

plt.close()

def donor_to_recipient_HGT_heatmap(sponge_type_list_sorted, donor_to_recipient_HGT_num_matrix_file_absolute, donor_to_recipient_HGT_num_matrix_file_normalized, direction_by_host_count_dict, sponge_to_bin_total_size_dict, donor_to_recipient_HGT_num_plot_absolute, donor_to_recipient_HGT_num_plot_normalized, skip_plot):

donor_to_recipient_HGT_num_matrix_file_absolute_handle = open(donor_to_recipient_HGT_num_matrix_file_absolute, 'w')

donor_to_recipient_HGT_num_matrix_file_normalized_handle = open(donor_to_recipient_HGT_num_matrix_file_normalized, 'w')

donor_to_recipient_HGT_num_matrix_file_absolute_handle.write(',%s\n' % ','.join(sponge_type_list_sorted))

donor_to_recipient_HGT_num_matrix_file_normalized_handle.write(',%s\n' % ','.join(sponge_type_list_sorted))

donor_to_recipient_HGT_num_absolute_lol = []

donor_to_recipient_HGT_num_normalized_lol = []

within_sponge_values = []

between_sponge_values = []

for sponge_donor in sponge_type_list_sorted:

curret_donor_to_recipient_list_absolute = []

curret_donor_to_recipient_list_normalized = []

for sponge_recipient in sponge_type_list_sorted:

donor_to_recipient = '%s-->%s' % (sponge_donor, sponge_recipient)

# get donor_to_recipient_HGT_num

if donor_to_recipient in direction_by_host_count_dict:

donor_to_recipient_HGT_num_absolute = direction_by_host_count_dict[donor_to_recipient]

donor_to_recipient_HGT_num_normalized = float("{0:.2f}".format(

donor_to_recipient_HGT_num_absolute / sponge_to_bin_total_size_dict[sponge_recipient]))

else:

donor_to_recipient_HGT_num_absolute = 0

donor_to_recipient_HGT_num_normalized = 0

if sponge_donor == sponge_recipient:

within_sponge_values.append(donor_to_recipient_HGT_num_normalized)

else:

between_sponge_values.append(donor_to_recipient_HGT_num_normalized)

curret_donor_to_recipient_list_absolute.append(donor_to_recipient_HGT_num_absolute)

curret_donor_to_recipient_list_normalized.append(donor_to_recipient_HGT_num_normalized)

donor_to_recipient_HGT_num_absolute_lol.append(curret_donor_to_recipient_list_absolute)

donor_to_recipient_HGT_num_normalized_lol.append(curret_donor_to_recipient_list_normalized)

current_donor_to_recipient_list_as_string_absolute = [str(i) for i in curret_donor_to_recipient_list_absolute]

current_donor_to_recipient_list_as_string_normalized = [str(i) for i in curret_donor_to_recipient_list_normalized]

donor_to_recipient_HGT_num_matrix_file_absolute_handle.write('%s,%s\n' % (sponge_donor, ','.join(current_donor_to_recipient_list_as_string_absolute)))

donor_to_recipient_HGT_num_matrix_file_normalized_handle.write('%s,%s\n' % (sponge_donor, ','.join(current_donor_to_recipient_list_as_string_normalized)))

donor_to_recipient_HGT_num_matrix_file_absolute_handle.close()

donor_to_recipient_HGT_num_matrix_file_normalized_handle.close()

# get the plot

if skip_plot is False:

plot_donor_to_recipient_HGT_num(donor_to_recipient_HGT_num_absolute_lol, sponge_type_list_sorted, donor_to_recipient_HGT_num_plot_absolute)

plot_donor_to_recipient_HGT_num(donor_to_recipient_HGT_num_normalized_lol, sponge_type_list_sorted, donor_to_recipient_HGT_num_plot_normalized)

return within_sponge_values, between_sponge_values

######################################################## dRep99 ########################################################

# file in

metadata_file_dRep = 'SpongeEMP_metadata_dRep99.txt'

detected_HGTs_pcofg = 'MetaCHIP_detected_HGTs.txt'

# file out

donor_to_recipient_HGT_num_matrix_file_absolute_high = 'Figure_S10_absolute_num.txt'

donor_to_recipient_HGT_num_matrix_file_normalized_high = 'Figure_S10.txt'

donor_to_recipient_HGT_num_plot_absolute_high = 'Figure_S10_absolute_num.svg'

donor_to_recipient_HGT_num_plot_normalized_high = 'Figure_S10.svg'

Donor_to_recipient_HGT_identity_plot_folder = 'Figure_S9_separated'

########################################################################################################################

force_create_folder(Donor_to_recipient_HGT_identity_plot_folder)

new2raw_name_dict = {'CAR4_bin_6': 'CAR4_bin_6', 'COS2_bin_4': 'COS2_bin_4', 'COS4_bin_21': 'COS4_bin_21', 'COS2_bin_10': 'COS2_bin_10', 'IRC4_bin_41': 'IRC4_bin_41', 'IRC_PAM_SB0675_bin_5': 'IRC_PAM_SB0675_bin_5', 'RHO2_bin_49': 'RHO2_bin_49', 'IRC_PAM_SB0665_bin_25': 'IRC_PAM_SB0665_bin_25', 'COS2_bin_7': 'COS2_bin_7', 'IRC_PAM_SB0667_bin_1': 'IRC_PAM_SB0667_bin_1', 'RHO3_bin_84': 'RHO3_bin_84', 'IRC3_bin_26': 'IRC3_bin_26', 'IRC3_bin_28': 'IRC3_bin_28', 'RHO1_bin_56': 'RHO1_bin_56', 'IRC_PAM_SB0668_bin_19': 'IRC_PAM_SB0668_bin_19', 'IRC_PAM_SB0666_bin_34': 'IRC_PAM_SB0666_bin_34', 'seawater_bettina_36328_bin_3': 'seawater_bettina_36328_bin_3', 'COS36405_bin_3': 'COS36405_bin_3', 'RHO1_bin_37': 'RHO1_bin_37', 'IRC_PAM_SB0661_bin_2': 'IRC_PAM_SB0661_bin_2', 'RHO1_bin_65': 'RHO1_bin_65', 'IRC_PAM_SB0665_bin_4': 'IRC_PAM_SB0665_bin_4', 'IRC3_bin_18': 'IRC3_bin_18', 'RHO1_bin_53': 'RHO1_bin_53', 'COS3_bin_9': 'COS3_bin_9', 'RHO2_bin_42': 'RHO2_bin_42', 'CAR1_bin_10': 'CAR1_bin_10', 'IRC_PAM_SB0661_bin_17': 'IRC_PAM_SB0661_bin_17', 'IRC4_bin_3': 'IRC4_bin_3', 'COS36388_bin_13': 'COS36388_bin_13', 'APA_bin_59': 'APA_bin_59', 'APA_bin_42': 'APA_bin_42', 'IRC_PAM_SB0665_bin_27': 'IRC_PAM_SB0665_bin_27', 'CAR2_bin_5': 'CAR2_bin_5', 'seawater_bettina_36310_bin_3': 'seawater_bettina_36310_bin_3', 'Aquimarina_spAU474': 'TED_estevesThomas2016_Aquimarina_spAU474_2606217188', 'IRC4_bin_29': 'IRC4_bin_29', 'IRC_PAM_SB0661_bin_5': 'IRC_PAM_SB0661_bin_5', 'COS36387_bin_19': 'COS36387_bin_19', 'IRC_PAM_SB0661_bin_22': 'IRC_PAM_SB0661_bin_22', 'seawater_22112_bin_25': 'seawater_22112_bin_25', 'RHO3_bin_32': 'RHO3_bin_32', 'IRC_PAM_SB0665_bin_9': 'IRC_PAM_SB0665_bin_9', 'IRC1_bin_14': 'IRC1_bin_14', 'CAR2_bin_13': 'CAR2_bin_13', 'COS36388_bin_11': 'COS36388_bin_11', 'IRC_PAM_SB0661_bin_16': 'IRC_PAM_SB0661_bin_16', 'APA_bin_13': 'APA_bin_13', 'IRC_PAM_SB0677_bin_19': 'IRC_PAM_SB0677_bin_19', 'COS36386_bin_13': 'COS36386_bin_13', 'RHO3_bin_28': 'RHO3_bin_28', 'APA_bin_102': 'APA_bin_102', 'CAR1_bin_9': 'CAR1_bin_9', 'IRC4_bin_33': 'IRC4_bin_33', 'APA_bin_91': 'APA_bin_91', 'COS4_bin_42': 'COS4_bin_42', 'COS1_bin_11': 'COS1_bin_11', 'APA_bin_52': 'APA_bin_52', 'COS36386_bin_2': 'COS36386_bin_2', 'APA_bin_68': 'APA_bin_68', 'RHO3_bin_59': 'RHO3_bin_59', 'RHO1_bin_25': 'RHO1_bin_25', 'CLI1_bin_3': 'CLI1_bin_3', 'IRC1_bin_27': 'IRC1_bin_27', 'STY1_bin_6': 'STY1_bin_6', 'IRC_PAM_SB0675_bin_4': 'IRC_PAM_SB0675_bin_4', 'COS4_bin_7': 'COS4_bin_7', 'APA_bin_93': 'APA_bin_93', 'RHO1_bin_13': 'RHO1_bin_13', 'IRC_PAM_SB0677_bin_15': 'IRC_PAM_SB0677_bin_15', 'STY3_bin_6': 'STY3_bin_6', 'GCA_003635255': 'PSE_podellAllen_GCA_003635255.1_ASM363525v1_genomic', 'APA_bin_5': 'APA_bin_5', 'IRC_PAM_SB0673_bin_10': 'IRC_PAM_SB0673_bin_10', 'seawater_bettina_36309_bin_5': 'seawater_bettina_36309_bin_5', 'IRC_PAM_SB0678_bin_5': 'IRC_PAM_SB0678_bin_5', 'COS36404_bin_12': 'COS36404_bin_12', 'IRC_PAM_SB0677_bin_1': 'IRC_PAM_SB0677_bin_1', 'COS36387_bin_20': 'COS36387_bin_20', 'CAR2_bin_10': 'CAR2_bin_10', 'IRC_PAM_SB0662_bin_27': 'IRC_PAM_SB0662_bin_27', 'IRC1_bin_21': 'IRC1_bin_21', 'IRC1_bin_34': 'IRC1_bin_34', 'IRC_PAM_SB0678_bin_7': 'IRC_PAM_SB0678_bin_7', 'STY1_bin_5': 'STY1_bin_5', 'IRC_PAM_SB0675_bin_1': 'IRC_PAM_SB0675_bin_1', 'RHO3_bin_71': 'RHO3_bin_71', 'IRC4_bin_7': 'IRC4_bin_7', 'IRC_PAM_SB0677_bin_16': 'IRC_PAM_SB0677_bin_16', 'GCF_900149685': 'SPOO_karimiCosta2019_GCF_900149685.1_Erythrobacter_sp._Alg231_14_genomic', 'IRC2_bin_10': 'IRC2_bin_10', 'seawater_22112_bin_29': 'seawater_22112_bin_29', 'COS36386_bin_32': 'COS36386_bin_32', 'seawater_seasim_SB9160_S9_bin_9': 'seawater_seasim_SB9160_S9_bin_9', 'seawater_bettina_36328_bin_8': 'seawater_bettina_36328_bin_8', 'seawater_seasim_SB9156_S5_bin_6': 'seawater_seasim_SB9156_S5_bin_6', 'IRC_PAM_SB0664_bin_4': 'IRC_PAM_SB0664_bin_4', 'RHO1_bin_23': 'RHO1_bin_23', 'COS36388_bin_15': 'COS36388_bin_15', 'GCA_001541925': 'SUB_tianQian_GCA_001541925.1_ASM154192v1_genomic', 'COS2_bin_3': 'COS2_bin_3', 'CHO1_bin_2': 'CHO1_bin_2', 'APA_bin_80': 'APA_bin_80', 'Aquimarina_spAU58': 'TED_estevesThomas2016_Aquimarina_spAU58_IMGid2606217182', 'COS2_bin_6': 'COS2_bin_6', 'CLI1_bin_1': 'CLI1_bin_1', 'seawater_42618_bin_29': 'seawater_42618_bin_29', 'COS3_bin_6': 'COS3_bin_6', 'seawater_seasim_SB9152_S1_bin_8': 'seawater_seasim_SB9152_S1_bin_8', 'seawater_bettina_36308_bin_5': 'seawater_bettina_36308_bin_5', 'IRC_PAM_SB0677_bin_11': 'IRC_PAM_SB0677_bin_11', 'RHO1_bin_18': 'RHO1_bin_18', 'RHO3_bin_40': 'RHO3_bin_40', 'IRC2_bin_3': 'IRC2_bin_3', 'IRC3_bin_13': 'IRC3_bin_13', 'RHO3_bin_36': 'RHO3_bin_36', 'COS3_bin_14': 'COS3_bin_14', 'IRC2_bin_11': 'IRC2_bin_11', 'seawater_22112_bin_6': 'seawater_22112_bin_6', 'RHO2_bin_67': 'RHO2_bin_67', 'COS1_bin_8': 'COS1_bin_8', 'CAR1_bin_1': 'CAR1_bin_1', 'IRC_PAM_SB0662_bin_7': 'IRC_PAM_SB0662_bin_7', 'IRC_PAM_SB0668_bin_1': 'IRC_PAM_SB0668_bin_1', 'IRC_PAM_SB0677_bin_7': 'IRC_PAM_SB0677_bin_7', 'seawater_22112_bin_7': 'seawater_22112_bin_7', 'COS1_bin_4': 'COS1_bin_4', 'APA_bin_83': 'APA_bin_83', 'seawater_42617_bin_7': 'seawater_42617_bin_7', 'seawater_42618_bin_3': 'seawater_42618_bin_3', 'GCA_002631715': 'THES_lavyIlan_GCA_002631715.1_ASM263171v1_genomic', 'IRC2_bin_5': 'IRC2_bin_5', 'RHO2_bin_36': 'RHO2_bin_36', 'COS36386_bin_1': 'COS36386_bin_1', 'APA_bin_27': 'APA_bin_27', 'CAR2_bin_16': 'CAR2_bin_16', 'COS2_bin_19': 'COS2_bin_19', 'seawater_22112_bin_16': 'seawater_22112_bin_16', 'RHO1_bin_3': 'RHO1_bin_3', 'RHO3_bin_65': 'RHO3_bin_65', 'RHO3_bin_67': 'RHO3_bin_67', 'IRC4_bin_14': 'IRC4_bin_14', 'CLI3_bin_1': 'CLI3_bin_1', 'RHO3_bin_68': 'RHO3_bin_68', 'CAR2_bin_3': 'CAR2_bin_3', 'COS4_bin_6': 'COS4_bin_6', 'IRC1_bin_13': 'IRC1_bin_13', 'CAR2_bin_18': 'CAR2_bin_18', 'GCA_003635205': 'PSE_podellAllen_GCA_003635205.1_ASM363520v1_genomic', 'COS36388_bin_6': 'COS36388_bin_6', 'seawater_bettina_36308_bin_3': 'seawater_bettina_36308_bin_3', 'CAR2_bin_15': 'CAR2_bin_15', 'CLI2_bin_1': 'CLI2_bin_1', 'CAR4_bin_5': 'CAR4_bin_5', 'seawater_22112_bin_15': 'seawater_22112_bin_15', 'IRC4_bin_10': 'IRC4_bin_10', 'RHO3_bin_53': 'RHO3_bin_53', 'GCA_003635265': 'PSE_podellAllen_GCA_003635265.1_ASM363526v1_genomic', 'seawater_seasim_SB9152_S1_bin_3': 'seawater_seasim_SB9152_S1_bin_3', 'IRC_PAM_SB0665_bin_24': 'IRC_PAM_SB0665_bin_24', 'GCA_003635305': 'AGET_podellAllen_GCA_003635305.1_ASM363530v1_genomic', 'IRC_PAM_SB0662_bin_9': 'IRC_PAM_SB0662_bin_9', 'IRC_PAM_SB0664_bin_21': 'IRC_PAM_SB0664_bin_21', 'CAR3_bin_12': 'CAR3_bin_12', 'IRC4_bin_38': 'IRC4_bin_38', 'CAR4_bin_3': 'CAR4_bin_3', 'RHO3_bin_34': 'RHO3_bin_34', 'COS36387_bin_4': 'COS36387_bin_4', 'IRC1_bin_12': 'IRC1_bin_12', 'COS4_bin_40': 'COS4_bin_40', 'STY4_bin_1': 'STY4_bin_1', 'RHO1_bin_19': 'RHO1_bin_19', 'COS4_bin_24': 'COS4_bin_24', 'COS36386_bin_17': 'COS36386_bin_17', 'IRC_PAM_SB0675_bin_10': 'IRC_PAM_SB0675_bin_10', 'APA_bin_73': 'APA_bin_73', 'RHO2_bin_1': 'RHO2_bin_1', 'COS36386_bin_27': 'COS36386_bin_27', 'COS3_bin_13': 'COS3_bin_13', 'IRC_PAM_SB0665_bin_10': 'IRC_PAM_SB0665_bin_10', 'RHO3_bin_13': 'RHO3_bin_13', 'IRC_PAM_SB0665_bin_13': 'IRC_PAM_SB0665_bin_13', 'APA_bin_24': 'APA_bin_24', 'RHO3_bin_64': 'RHO3_bin_64', 'Ruegeria_spAU67': 'TED_estevesThomas2016_Ruegeria_spAU67_2606217183', 'IRC_PAM_SB0668_bin_6': 'IRC_PAM_SB0668_bin_6', 'RHO1_bin_62': 'RHO1_bin_62', 'IRC_PAM_SB0661_bin_45': 'IRC_PAM_SB0661_bin_45', 'IRC_PAM_SB0662_bin_26': 'IRC_PAM_SB0662_bin_26', 'COS1_bin_19': 'COS1_bin_19', 'RHO3_bin_11': 'RHO3_bin_11', 'RHO1_bin_46': 'RHO1_bin_46', 'RHO1_bin_14': 'RHO1_bin_14', 'IRC_PAM_SB0664_bin_31': 'IRC_PAM_SB0664_bin_31', 'IRC2_bin_6': 'IRC2_bin_6', 'COS1_bin_17': 'COS1_bin_17', 'IRC3_bin_19': 'IRC3_bin_19', 'GCF_900143525': 'SPOO_karimiCosta2019_GCF_900143525.1_Ruegeria_sp._Alg231_54_genomic', 'RHO1_bin_45': 'RHO1_bin_45', 'IRC_PAM_SB0664_bin_16': 'IRC_PAM_SB0664_bin_16', 'RHO2_bin_65': 'RHO2_bin_65', 'RHO2_bin_9': 'RHO2_bin_9', 'seawater_22112_bin_5': 'seawater_22112_bin_5', 'IRC_PAM_SB0670_bin_12': 'IRC_PAM_SB0670_bin_12', 'CAR4_bin_10': 'CAR4_bin_10', 'GCF_900109375': 'AMPF_kennedyDobson_GCF_900109375.1_IMGID2622736580_genomic', 'RHO3_bin_16': 'RHO3_bin_16', 'IRC_PAM_SB0677_bin_2': 'IRC_PAM_SB0677_bin_2', 'CYMC_moitinhoThomas_67496': 'CYMC_moitinhoThomas_67496.assembled', 'RHO1_bin_48': 'RHO1_bin_48', 'IRC_PAM_SB0665_bin_1': 'IRC_PAM_SB0665_bin_1', 'APA_bin_14': 'APA_bin_14', 'renamed': 'raw_name', 'COS4_bin_5': 'COS4_bin_5', 'IRC_PAM_SB0662_bin_20': 'IRC_PAM_SB0662_bin_20', 'COS1_bin_6': 'COS1_bin_6', 'IRC_PAM_SB0661_bin_4': 'IRC_PAM_SB0661_bin_4', 'RHO1_bin_1': 'RHO1_bin_1', 'GCF_900143535': 'SPOO_karimiCosta2019_GCF_900143535.1_Tateyamaria_sp._Alg231_49_genomic', 'RHO3_bin_31': 'RHO3_bin_31', 'RHO2_bin_51': 'RHO2_bin_51', 'seawater_bettina_36327_bin_2': 'seawater_bettina_36327_bin_2', 'IRC3_bin_31': 'IRC3_bin_31', 'IRC_PAM_SB0661_bin_37': 'IRC_PAM_SB0661_bin_37', 'aplysina_bin': 'APA_garciaTyalor_SAUL_aplysina_bin', 'CAR3_bin_6': 'CAR3_bin_6', 'COS36387_bin_6': 'COS36387_bin_6', 'RHO2_bin_37': 'RHO2_bin_37', 'COS4_bin_3': 'COS4_bin_3', 'COS3_bin_2': 'COS3_bin_2', 'CHO1_bin_1': 'CHO1_bin_1', 'COS4_bin_19': 'COS4_bin_19', 'IRC_PAM_SB0662_bin_33': 'IRC_PAM_SB0662_bin_33', 'IRC_PAM_SB0675_bin_19': 'IRC_PAM_SB0675_bin_19', 'COS36404_bin_1': 'COS36404_bin_1', 'RHO3_bin_58': 'RHO3_bin_58', 'CAR3_bin_1': 'CAR3_bin_1', 'CAR3_bin_7': 'CAR3_bin_7', 'IRC_PAM_SB0662_bin_5': 'IRC_PAM_SB0662_bin_5', 'IRC_PAM_SB0662_bin_22': 'IRC_PAM_SB0662_bin_22', 'APA_bin_26': 'APA_bin_26', 'CAR4_bin_18': 'CAR4_bin_18', 'COS36386_bin_19': 'COS36386_bin_19', 'IRC_PAM_SB0661_bin_41': 'IRC_PAM_SB0661_bin_41', 'IRC_PAM_SB0664_bin_25': 'IRC_PAM_SB0664_bin_25', 'IRC_PAM_SB0670_bin_20': 'IRC_PAM_SB0670_bin_20', 'APA_bin_81': 'APA_bin_81', 'IRC_PAM_SB0676_bin_21': 'IRC_PAM_SB0676_bin_21', 'COS4_bin_43': 'COS4_bin_43', 'RHO2_bin_18': 'RHO2_bin_18', 'APA_bin_33': 'APA_bin_33', 'CAR2_bin_20': 'CAR2_bin_20', 'seawater_bettina_36309_bin_17': 'seawater_bettina_36309_bin_17', 'CAR2_bin_2': 'CAR2_bin_2', 'IRC_PAM_SB0664_bin_27': 'IRC_PAM_SB0664_bin_27', 'RHO1_bin_85': 'RHO1_bin_85', 'seawater_bettina_36310_bin_4': 'seawater_bettina_36310_bin_4', 'COS1_bin_20': 'COS1_bin_20', 'IRC_PAM_SB0662_bin_58': 'IRC_PAM_SB0662_bin_58', 'CAR1_bin_12': 'CAR1_bin_12', 'seawater_22112_bin_10': 'seawater_22112_bin_10', 'CAR1_bin_3': 'CAR1_bin_3', 'IRC4_bin_8': 'IRC4_bin_8', 'IRC_PAM_SB0675_bin_2': 'IRC_PAM_SB0675_bin_2', 'IRC_PAM_SB0665_bin_2': 'IRC_PAM_SB0665_bin_2', 'IRC_PAM_SB0670_bin_22': 'IRC_PAM_SB0670_bin_22', 'IRC_PAM_SB0664_bin_22': 'IRC_PAM_SB0664_bin_22', 'seawater_seasim_SB9152_S1_bin_1': 'seawater_seasim_SB9152_S1_bin_1', 'RHO3_bin_69': 'RHO3_bin_69', 'seawater_seasim_SB9153_S2_bin_2': 'seawater_seasim_SB9153_S2_bin_2', 'IRC_PAM_SB0666_bin_17': 'IRC_PAM_SB0666_bin_17', 'IRC_PAM_SB0661_bin_3': 'IRC_PAM_SB0661_bin_3', 'IRC_PAM_SB0673_bin_16': 'IRC_PAM_SB0673_bin_16', 'IRC_PAM_SB0662_bin_35': 'IRC_PAM_SB0662_bin_35', 'RHO3_bin_35': 'RHO3_bin_35', 'COS2_bin_17': 'COS2_bin_17', 'IRC4_bin_23': 'IRC4_bin_23', 'RHO2_bin_30': 'RHO2_bin_30', 'IRC4_bin_24': 'IRC4_bin_24', 'seawater_22112_bin_28': 'seawater_22112_bin_28', 'CAR2_bin_7': 'CAR2_bin_7', 'APA_bin_94': 'APA_bin_94', 'APA_bin_85': 'APA_bin_85', 'IRC1_bin_17': 'IRC1_bin_17', 'COS36404_bin_9': 'COS36404_bin_9', 'COS36404_bin_2': 'COS36404_bin_2', 'CAR3_bin_16': 'CAR3_bin_16', 'IRC4_bin_44': 'IRC4_bin_44', 'seawater_22112_bin_3': 'seawater_22112_bin_3', 'CAR1_bin_11': 'CAR1_bin_11', 'APA_bin_17': 'APA_bin_17', 'RHO2_bin_59': 'RHO2_bin_59', 'seawater_seasim_SB9155_S4_bin_12': 'seawater_seasim_SB9155_S4_bin_12', 'GCF_001941685': 'AREB_froesThompson_GCF_001941685.1_ASM194168v1_genomic', 'IRC1_bin_35': 'IRC1_bin_35', 'RHO2_bin_33': 'RHO2_bin_33', 'IRC1_bin_15': 'IRC1_bin_15', 'RHO2_bin_54': 'RHO2_bin_54', 'RHO3_bin_7': 'RHO3_bin_7', 'COS4_bin_44': 'COS4_bin_44', 'petrosia_ficiformis_bin': 'PETF_garciaTaylor_SAUL_petrosia_ficiformis_bin', 'APA_bin_70': 'APA_bin_70', 'RHO2_bin_7': 'RHO2_bin_7', 'IRC_PAM_SB0664_bin_9': 'IRC_PAM_SB0664_bin_9', 'RHO3_bin_47': 'RHO3_bin_47', 'COS36387_bin_1': 'COS36387_bin_1', 'IRC3_bin_12': 'IRC3_bin_12', 'COS1_bin_12': 'COS1_bin_12', 'CAR4_bin_2': 'CAR4_bin_2', 'CAR4_bin_8': 'CAR4_bin_8', 'IRC_PAM_SB0666_bin_10': 'IRC_PAM_SB0666_bin_10', 'IRC_PAM_SB0662_bin_11': 'IRC_PAM_SB0662_bin_11', 'COS3_bin_18': 'COS3_bin_18', 'GCF_003676335': 'unknown_alexAntunes_GCF_003676335.1_ASM367633v1_genomic', 'IRC_PAM_SB0678_bin_9': 'IRC_PAM_SB0678_bin_9', 'RHO1_bin_4': 'RHO1_bin_4', 'RHO3_bin_72': 'RHO3_bin_72', 'RHO1_bin_67': 'RHO1_bin_67', 'RHO1_bin_44': 'RHO1_bin_44', 'IRC4_bin_2': 'IRC4_bin_2', 'IRC_PAM_SB0676_bin_26': 'IRC_PAM_SB0676_bin_26', 'CAR4_bin_9': 'CAR4_bin_9', 'seawater_seasim_SB9160_S9_bin_11': 'seawater_seasim_SB9160_S9_bin_11', 'IRC_PAM_SB0675_bin_14': 'IRC_PAM_SB0675_bin_14', 'RHO1_bin_32': 'RHO1_bin_32', 'COS4_bin_2': 'COS4_bin_2', 'RHO2_bin_38': 'RHO2_bin_38', 'COS36387_bin_2': 'COS36387_bin_2', 'COS3_bin_8': 'COS3_bin_8', 'IRC_PAM_SB0661_bin_29': 'IRC_PAM_SB0661_bin_29', 'COS3_bin_12': 'COS3_bin_12', 'RHO3_bin_41': 'RHO3_bin_41', 'GCF_900143615': 'SPOO_karimiCosta2019_GCF_900143615.1_Rhodobacteraceae_bacterium_Alg231_30_genomic', 'RHO3_bin_38': 'RHO3_bin_38', 'GCF_900149695': 'SPOO_karimiCosta2019_GCF_900149695.1_Anderseniella_sp._Alg231_50_genomic', 'IRC_PAM_SB0672_bin_11': 'IRC_PAM_SB0672_bin_11', 'STY3_bin_3': 'STY3_bin_3', 'IRC1_bin_1': 'IRC1_bin_1', 'IRC_PAM_SB0662_bin_12': 'IRC_PAM_SB0662_bin_12', 'seawater_seasim_SB9157_S6_bin_2': 'seawater_seasim_SB9157_S6_bin_2', 'RHO1_bin_40': 'RHO1_bin_40', 'IRC2_bin_8': 'IRC2_bin_8', 'seawater_22112_bin_14': 'seawater_22112_bin_14', 'seawater_42618_bin_2': 'seawater_42618_bin_2', 'COS4_bin_20': 'COS4_bin_20', 'IRC1_bin_3': 'IRC1_bin_3', 'RHO3_bin_66': 'RHO3_bin_66', 'CAR3_bin_18': 'CAR3_bin_18', 'COS1_bin_7': 'COS1_bin_7', 'GCA_001007625': 'IRCvar_burgsdorfSteindler_GCA_001007625.1_ASM100762v1_genomic', 'RHO2_bin_23': 'RHO2_bin_23', 'IRC1_bin_16': 'IRC1_bin_16', 'GCA_001543005': 'LOPHE_tianQian_GCA_001543005.1_ASM154300v1_genomic', 'CAR2_bin_6': 'CAR2_bin_6', 'IRC3_bin_25': 'IRC3_bin_25', 'IRC4_bin_6': 'IRC4_bin_6', 'IRC1_bin_10': 'IRC1_bin_10', 'COS36404_bin_19': 'COS36404_bin_19', 'RHO3_bin_12': 'RHO3_bin_12', 'CAR3_bin_3': 'CAR3_bin_3', 'IRC4_bin_37': 'IRC4_bin_37', 'IRC_PAM_SB0662_bin_49': 'IRC_PAM_SB0662_bin_49', 'COS1_bin_1': 'COS1_bin_1', 'IRC_PAM_SB0662_bin_59': 'IRC_PAM_SB0662_bin_59', 'RHO3_bin_6': 'RHO3_bin_6', 'CAR2_bin_14': 'CAR2_bin_14', 'GCA_001542995': 'LOPHE_tianQian_GCA_001542995.1_ASM154299v1_genomic', 'RHO3_bin_48': 'RHO3_bin_48', 'RHO3_bin_37': 'RHO3_bin_37', 'COS4_bin_14': 'COS4_bin_14', 'APA_bin_4': 'APA_bin_4', 'RHO2_bin_24': 'RHO2_bin_24', 'GCF_000158135': 'MYCL_zanHill_GCF_000158135.1_ASM15813v1_genomic', 'APA_bin_82': 'APA_bin_82', 'APA_bin_38': 'APA_bin_38', 'IRC_PAM_SB0675_bin_20': 'IRC_PAM_SB0675_bin_20', 'RHO3_bin_5': 'RHO3_bin_5', 'IRC_PAM_SB0661_bin_38': 'IRC_PAM_SB0661_bin_38', 'COS3_bin_5': 'COS3_bin_5', 'RHO3_bin_10': 'RHO3_bin_10', 'CAR2_bin_9': 'CAR2_bin_9', 'IRC_PAM_SB0661_bin_15': 'IRC_PAM_SB0661_bin_15', 'APA_bin_90': 'APA_bin_90', 'IRC_PAM_SB0670_bin_18': 'IRC_PAM_SB0670_bin_18', 'APA_bin_87': 'APA_bin_87', 'COS36405_bin_7': 'COS36405_bin_7', 'RHO2_bin_64': 'RHO2_bin_64', 'STY4_bin_2': 'STY4_bin_2', 'IRC2_bin_2': 'IRC2_bin_2', 'STY4_bin_9': 'STY4_bin_9', 'APA_bin_58': 'APA_bin_58', 'COS4_bin_32': 'COS4_bin_32', 'seawater_seasim_SB9156_S5_bin_8': 'seawater_seasim_SB9156_S5_bin_8', 'COS2_bin_22': 'COS2_bin_22', 'COS36405_bin_20': 'COS36405_bin_20', 'IRC_PAM_SB0662_bin_51': 'IRC_PAM_SB0662_bin_51', 'IRC_PAM_SB0665_bin_8': 'IRC_PAM_SB0665_bin_8', 'RHO3_bin_54': 'RHO3_bin_54', 'IRC_PAM_SB0666_bin_21': 'IRC_PAM_SB0666_bin_21', 'RHO2_bin_3': 'RHO2_bin_3', 'seawater_42618_bin_25': 'seawater_42618_bin_25', 'RHO2_bin_57': 'RHO2_bin_57', 'seawater_bettina_36328_bin_2': 'seawater_bettina_36328_bin_2', 'COS4_bin_11': 'COS4_bin_11', 'RHO1_bin_42': 'RHO1_bin_42', 'IRC1_bin_6': 'IRC1_bin_6', 'COS36386_bin_3': 'COS36386_bin_3', 'RHO2_bin_20': 'RHO2_bin_20', 'GCF_900149705': 'SPOO_karimiCosta2019_GCF_900149705.1_Sphingorhabdus_sp._Alg231_15_genomic', 'IRC_PAM_SB0661_bin_43': 'IRC_PAM_SB0661_bin_43', 'CAR4_bin_15': 'CAR4_bin_15', 'IRC1_bin_7': 'IRC1_bin_7', 'IRC_PAM_SB0661_bin_8': 'IRC_PAM_SB0661_bin_8', 'seawater_bettina_36328_bin_5': 'seawater_bettina_36328_bin_5', 'STY2_bin_4': 'STY2_bin_4', 'GCF_900143545': 'SPOO_karimiCosta2019_GCF_900143545.1_Loktanella_sp._Alg231_35_genomic', 'IRC_PAM_SB0664_bin_10': 'IRC_PAM_SB0664_bin_10', 'GCF_900143635': 'SPOO_karimiCosta2019_GCF_900143635.1_Rhodobacteraceae_bacterium_Alg231_04_genomic', 'COS4_bin_16': 'COS4_bin_16', 'RHO1_bin_60': 'RHO1_bin_60', 'IRC_PAM_SB0668_bin_20': 'IRC_PAM_SB0668_bin_20', 'RHO2_bin_16': 'RHO2_bin_16', 'RHO3_bin_79': 'RHO3_bin_79', 'CAR1_bin_13': 'CAR1_bin_13', 'COS1_bin_15': 'COS1_bin_15', 'IRC_PAM_SB0666_bin_1': 'IRC_PAM_SB0666_bin_1', 'CAR1_bin_2': 'CAR1_bin_2', 'IRC_PAM_SB0664_bin_3': 'IRC_PAM_SB0664_bin_3', 'COS4_bin_29': 'COS4_bin_29', 'IRC_PAM_SB0670_bin_1': 'IRC_PAM_SB0670_bin_1', 'IRC1_bin_18': 'IRC1_bin_18', 'IRC4_bin_22': 'IRC4_bin_22', 'COS36404_bin_6': 'COS36404_bin_6', 'IRC2_bin_13': 'IRC2_bin_13', 'APA_bin_18': 'APA_bin_18', 'COS36406_bin_4': 'COS36406_bin_4', 'IRC1_bin_11': 'IRC1_bin_11', 'seawater_bettina_36327_bin_15': 'seawater_bettina_36327_bin_15', 'COS1_bin_2': 'COS1_bin_2', 'seawater_seasim_SB9154_S3_bin_4': 'seawater_seasim_SB9154_S3_bin_4', 'COS36386_bin_8': 'COS36386_bin_8', 'RHO1_bin_49': 'RHO1_bin_49', 'IRC1_bin_4': 'IRC1_bin_4', 'IRC_PAM_SB0662_bin_15': 'IRC_PAM_SB0662_bin_15', 'IRC4_bin_20': 'IRC4_bin_20', 'seawater_bettina_36326_bin_1': 'seawater_bettina_36326_bin_1', 'IRC_PAM_SB0661_bin_30': 'IRC_PAM_SB0661_bin_30', 'GCF_004168585': 'OPHP_alexAntunes_GCF_004168585.1_ASM416858v1_genomic', 'RHO2_bin_11': 'RHO2_bin_11', 'IRC_PAM_SB0662_bin_37': 'IRC_PAM_SB0662_bin_37', 'RHO3_bin_2': 'RHO3_bin_2', 'COS36386_bin_18': 'COS36386_bin_18', 'IRC_PAM_SB0661_bin_24': 'IRC_PAM_SB0661_bin_24', 'seawater_bettina_36326_bin_26': 'seawater_bettina_36326_bin_26', 'IRC_PAM_SB0664_bin_15': 'IRC_PAM_SB0664_bin_15', 'COS36406_bin_19': 'COS36406_bin_19', 'COS36386_bin_10': 'COS36386_bin_10', 'IRC_PAM_SB0663_bin_5': 'IRC_PAM_SB0663_bin_5', 'IRC_PAM_SB0662_bin_21': 'IRC_PAM_SB0662_bin_21', 'COS2_bin_16': 'COS2_bin_16', 'IRC3_bin_20': 'IRC3_bin_20', 'IRC_PAM_SB0661_bin_44': 'IRC_PAM_SB0661_bin_44', 'IRC4_bin_25': 'IRC4_bin_25', 'CAR2_bin_11': 'CAR2_bin_11', 'APA_bin_72': 'APA_bin_72', 'COS4_bin_8': 'COS4_bin_8', 'seawater_seasim_SB9155_S4_bin_5': 'seawater_seasim_SB9155_S4_bin_5', 'IRC_PAM_SB0667_bin_9': 'IRC_PAM_SB0667_bin_9', 'STY2_bin_6': 'STY2_bin_6', 'STY3_bin_1': 'STY3_bin_1', 'IRC4_bin_1': 'IRC4_bin_1', 'seawater_bettina_36327_bin_4': 'seawater_bettina_36327_bin_4', 'RHO1_bin_58': 'RHO1_bin_58', 'IRC_PAM_SB0661_bin_33': 'IRC_PAM_SB0661_bin_33', 'IRC3_bin_5': 'IRC3_bin_5', 'seawater_seasim_SB9156_S5_bin_14': 'seawater_seasim_SB9156_S5_bin_14', 'IRC1_bin_22': 'IRC1_bin_22', 'COS36388_bin_4': 'COS36388_bin_4', 'RHO1_bin_9': 'RHO1_bin_9', 'RHO1_bin_66': 'RHO1_bin_66', 'APA_bin_43': 'APA_bin_43', 'RHO2_bin_28': 'RHO2_bin_28', 'seawater_seasim_SB9156_S5_bin_4': 'seawater_seasim_SB9156_S5_bin_4', 'RHO3_bin_21': 'RHO3_bin_21', 'IRC1_bin_2': 'IRC1_bin_2', 'IRC_PAM_SB0678_bin_15': 'IRC_PAM_SB0678_bin_15', 'CLI1_bin_4': 'CLI1_bin_4', 'IRC1_bin_25': 'IRC1_bin_25', 'COS36388_bin_18': 'COS36388_bin_18', 'IRC_PAM_SB0666_bin_22': 'IRC_PAM_SB0666_bin_22', 'IRC_PAM_SB0661_bin_11': 'IRC_PAM_SB0661_bin_11', 'CAR2_bin_4': 'CAR2_bin_4', 'CLI4_bin_1': 'CLI4_bin_1', 'IRC_PAM_SB0677_bin_8': 'IRC_PAM_SB0677_bin_8', 'RHO2_bin_53': 'RHO2_bin_53', 'seawater_seasim_SB9158_S7_bin_9': 'seawater_seasim_SB9158_S7_bin_9', 'COS36387_bin_18': 'COS36387_bin_18', 'seawater_42618_bin_5': 'seawater_42618_bin_5', 'COS36386_bin_29': 'COS36386_bin_29', 'RHO3_bin_51': 'RHO3_bin_51', 'RHO1_bin_72': 'RHO1_bin_72', 'COS3_bin_11': 'COS3_bin_11', 'RHO1_bin_6': 'RHO1_bin_6', 'IRC4_bin_4': 'IRC4_bin_4', 'STY2_bin_7': 'STY2_bin_7', 'RHO2_bin_35': 'RHO2_bin_35', 'RHO3_bin_20': 'RHO3_bin_20', 'APA_bin_45': 'APA_bin_45', 'IRC4_bin_35': 'IRC4_bin_35', 'COS36387_bin_9': 'COS36387_bin_9', 'IRC3_bin_22': 'IRC3_bin_22', 'RHO3_bin_26': 'RHO3_bin_26', 'CAR2_bin_26': 'CAR2_bin_26', 'CAR3_bin_13': 'CAR3_bin_13', 'IRC3_bin_7': 'IRC3_bin_7', 'RHO1_bin_30': 'RHO1_bin_30', 'RHO3_bin_14': 'RHO3_bin_14', 'COS36386_bin_6': 'COS36386_bin_6', 'RHO2_bin_43': 'RHO2_bin_43', 'IRC_PAM_SB0664_bin_5': 'IRC_PAM_SB0664_bin_5', 'RHO2_bin_15': 'RHO2_bin_15', 'RHO1_bin_2': 'RHO1_bin_2', 'IRC3_bin_10': 'IRC3_bin_10', 'RHO3_bin_18': 'RHO3_bin_18', 'APA_bin_7': 'APA_bin_7', 'COS1_bin_10': 'COS1_bin_10', 'IRC_PAM_SB0668_bin_27': 'IRC_PAM_SB0668_bin_27', 'RHO2_bin_12': 'RHO2_bin_12', 'COS36386_bin_4': 'COS36386_bin_4', 'IRC_PAM_SB0661_bin_32': 'IRC_PAM_SB0661_bin_32', 'RHO1_bin_27': 'RHO1_bin_27', 'GCA_003635315': 'MELS_podellAllen_GCA_GCA_003635315.1_ASM363531v1_genomic', 'IRC_PAM_SB0666_bin_13': 'IRC_PAM_SB0666_bin_13', 'STY2_bin_2': 'STY2_bin_2', 'RHO3_bin_17': 'RHO3_bin_17', 'seawater_seasim_SB9152_S1_bin_19': 'seawater_seasim_SB9152_S1_bin_19', 'RHO2_bin_60': 'RHO2_bin_60', 'RHO3_bin_9': 'RHO3_bin_9', 'STY4_bin_8': 'STY4_bin_8', 'seawater_42617_bin_1': 'seawater_42617_bin_1', 'COS36386_bin_35': 'COS36386_bin_35', 'COS2_bin_12': 'COS2_bin_12', 'seawater_22112_bin_12': 'seawater_22112_bin_12', 'seawater_seasim_SB9152_S1_bin_12': 'seawater_seasim_SB9152_S1_bin_12', 'CAR2_bin_8': 'CAR2_bin_8', 'CAR3_bin_5': 'CAR3_bin_5', 'COS4_bin_34': 'COS4_bin_34', 'IRC4_bin_45': 'IRC4_bin_45', 'STY4_bin_7': 'STY4_bin_7', 'IRC_PAM_SB0675_bin_12': 'IRC_PAM_SB0675_bin_12', 'COS4_bin_4': 'COS4_bin_4', 'seawater_bettina_36309_bin_3': 'seawater_bettina_36309_bin_3', 'IRC_PAM_SB0670_bin_39': 'IRC_PAM_SB0670_bin_39', 'IRC_PAM_SB0667_bin_3': 'IRC_PAM_SB0667_bin_3', 'IRC_PAM_SB0678_bin_6': 'IRC_PAM_SB0678_bin_6', 'COS36404_bin_8': 'COS36404_bin_8', 'seawater_22112_bin_1': 'seawater_22112_bin_1', 'CAR4_bin_14': 'CAR4_bin_14', 'COS36388_bin_5': 'COS36388_bin_5', 'seawater_22112_bin_17': 'seawater_22112_bin_17', 'COS36388_bin_17': 'COS36388_bin_17', 'COS36386_bin_9': 'COS36386_bin_9', 'GCA_001007635': 'APA_burgsdorfSteindler_GCA_001007635.1_ASM100763v1_genomic', 'APA_bin_39': 'APA_bin_39', 'COS36405_bin_11': 'COS36405_bin_11', 'RHO2_bin_50': 'RHO2_bin_50', 'RHO2_bin_26': 'RHO2_bin_26', 'IRC3_bin_35': 'IRC3_bin_35', 'RHO1_bin_7': 'RHO1_bin_7', 'IRC3_bin_14': 'IRC3_bin_14', 'RHO1_bin_21': 'RHO1_bin_21', 'IRC_PAM_SB0675_bin_18': 'IRC_PAM_SB0675_bin_18', 'APA_bin_15': 'APA_bin_15', 'APA_bin_86': 'APA_bin_86', 'seawater_bettina_36326_bin_7': 'seawater_bettina_36326_bin_7', 'RHO1_bin_50': 'RHO1_bin_50', 'RHO3_bin_15': 'RHO3_bin_15', 'COS36406_bin_6': 'COS36406_bin_6', 'IRC_PAM_SB0661_bin_14': 'IRC_PAM_SB0661_bin_14', 'COS4_bin_51': 'COS4_bin_51', 'IRC_PAM_SB0662_bin_53': 'IRC_PAM_SB0662_bin_53', 'COS3_bin_10': 'COS3_bin_10', 'RHO1_bin_70': 'RHO1_bin_70', 'IRC_PAM_SB0677_bin_14': 'IRC_PAM_SB0677_bin_14', 'COS4_bin_13': 'COS4_bin_13', 'APA_bin_96': 'APA_bin_96', 'COS36404_bin_10': 'COS36404_bin_10', 'STY4_bin_4': 'STY4_bin_4', 'COS2_bin_2': 'COS2_bin_2', 'RHO3_bin_52': 'RHO3_bin_52', 'COS4_bin_27': 'COS4_bin_27', 'APA_bin_41': 'APA_bin_41', 'IRC_PAM_SB0665_bin_5': 'IRC_PAM_SB0665_bin_5', 'Aquimarina_spAU119': 'TED_estevesThomas2016_Aquimarina_spAU119_2606217184', 'COS4_bin_17': 'COS4_bin_17', 'COS36388_bin_9': 'COS36388_bin_9', 'IRC_PAM_SB0661_bin_21': 'IRC_PAM_SB0661_bin_21', 'IRC_PAM_SB0662_bin_8': 'IRC_PAM_SB0662_bin_8', 'IRC_PAM_SB0664_bin_14': 'IRC_PAM_SB0664_bin_14', 'COS36386_bin_20': 'COS36386_bin_20', 'APA_bin_29': 'APA_bin_29', 'IRC4_bin_12': 'IRC4_bin_12', 'COS1_bin_13': 'COS1_bin_13', 'IRC_PAM_SB0675_bin_29': 'IRC_PAM_SB0675_bin_29', 'COS36387_bin_16': 'COS36387_bin_16', 'IRC_PAM_SB0661_bin_26': 'IRC_PAM_SB0661_bin_26', 'COS4_bin_55': 'COS4_bin_55', 'IRC_PAM_SB0668_bin_12': 'IRC_PAM_SB0668_bin_12', 'APA_bin_98': 'APA_bin_98', 'IRC_PAM_SB0665_bin_12': 'IRC_PAM_SB0665_bin_12', 'IRC3_bin_11': 'IRC3_bin_11', 'APA_bin_55': 'APA_bin_55', 'seawater_seasim_SB9152_S1_bin_4': 'seawater_seasim_SB9152_S1_bin_4', 'COS36386_bin_12': 'COS36386_bin_12', 'seawater_bettina_36328_bin_30': 'seawater_bettina_36328_bin_30', 'COS36388_bin_8': 'COS36388_bin_8', 'COS2_bin_5': 'COS2_bin_5', 'GCF_900143555': 'SPOO_karimiCosta2019_GCF_900143555.1_Labrenzia_sp._Alg231_36_genomic', 'RHO1_bin_81': 'RHO1_bin_81', 'CLI4_bin_2': 'CLI4_bin_2', 'APA_bin_63': 'APA_bin_63', 'SPOO_karimiCosta_FZLS01': 'SPOO_karimiCosta_FZLS01', 'APA_bin_69': 'APA_bin_69', 'IRC4_bin_21': 'IRC4_bin_21', 'IRC_PAM_SB0666_bin_6': 'IRC_PAM_SB0666_bin_6', 'RHO2_bin_52': 'RHO2_bin_52', 'COS3_bin_3': 'COS3_bin_3', 'RHO1_bin_33': 'RHO1_bin_33', 'RHO2_bin_10': 'RHO2_bin_10', 'COS2_bin_23': 'COS2_bin_23', 'COS36387_bin_22': 'COS36387_bin_22', 'PER4_bin_1': 'PER4_bin_1', 'APA_bin_48': 'APA_bin_48', 'RHO3_bin_27': 'RHO3_bin_27', 'seawater_seasim_SB9153_S2_bin_10': 'seawater_seasim_SB9153_S2_bin_10', 'IRC_PAM_SB0661_bin_19': 'IRC_PAM_SB0661_bin_19', 'RHO1_bin_34': 'RHO1_bin_34', 'IRC3_bin_6': 'IRC3_bin_6', 'COS36386_bin_15': 'COS36386_bin_15', 'CAR3_bin_4': 'CAR3_bin_4', 'APA_bin_32': 'APA_bin_32', 'CAR1_bin_6': 'CAR1_bin_6', 'RHO1_bin_15': 'RHO1_bin_15', 'RHO3_bin_39': 'RHO3_bin_39', 'IRC1_bin_30': 'IRC1_bin_30', 'RHO1_bin_11': 'RHO1_bin_11', 'GCA_002007405': 'SUB_tianQian_GCA_002007405.1_ASM200740v1_genomic', 'IRC_PAM_SB0664_bin_24': 'IRC_PAM_SB0664_bin_24', 'IRC_PAM_SB0662_bin_36': 'IRC_PAM_SB0662_bin_36', 'IRC_PAM_SB0661_bin_31': 'IRC_PAM_SB0661_bin_31', 'IRC3_bin_32': 'IRC3_bin_32', 'COS36406_bin_13': 'COS36406_bin_13', 'IRC2_bin_4': 'IRC2_bin_4', 'GCF_002573675': 'TED_braunBugni_GCF_002573675.1_ASM257367v1_genomic', 'IRC3_bin_4': 'IRC3_bin_4', 'COS36386_bin_11': 'COS36386_bin_11', 'COS2_bin_18': 'COS2_bin_18', 'COS36405_bin_5': 'COS36405_bin_5', 'seawater_bettina_36326_bin_6': 'seawater_bettina_36326_bin_6', 'IRC3_bin_9': 'IRC3_bin_9', 'APA_bin_40': 'APA_bin_40', 'seawater_seasim_SB9157_S6_bin_13': 'seawater_seasim_SB9157_S6_bin_13', 'CAR3_bin_2': 'CAR3_bin_2', 'seawater_42616_bin_2': 'seawater_42616_bin_2', 'RHO1_bin_39': 'RHO1_bin_39', 'RHO1_bin_64': 'RHO1_bin_64', 'IRC1_bin_37': 'IRC1_bin_37', 'IRC_PAM_SB0662_bin_28': 'IRC_PAM_SB0662_bin_28', 'COS4_bin_22': 'COS4_bin_22', 'COS36405_bin_14': 'COS36405_bin_14', 'COS4_bin_47': 'COS4_bin_47', 'COS3_bin_4': 'COS3_bin_4', 'RHO1_bin_28': 'RHO1_bin_28', 'IRC_PAM_SB0668_bin_11': 'IRC_PAM_SB0668_bin_11', 'STY1_bin_8': 'STY1_bin_8', 'RHO1_bin_22': 'RHO1_bin_22', 'COS4_bin_28': 'COS4_bin_28', 'APA_bin_12': 'APA_bin_12', 'seawater_42615_bin_2': 'seawater_42615_bin_2', 'IRC_PAM_SB0665_bin_19': 'IRC_PAM_SB0665_bin_19', 'COS36405_bin_2': 'COS36405_bin_2', 'COS36405_bin_10': 'COS36405_bin_10', 'COS36386_bin_28': 'COS36386_bin_28', 'RHO3_bin_70': 'RHO3_bin_70', 'seawater_bettina_36309_bin_6': 'seawater_bettina_36309_bin_6', 'IRC_PAM_SB0667_bin_2': 'IRC_PAM_SB0667_bin_2', 'APA_bin_34': 'APA_bin_34', 'IRC_PAM_SB0665_bin_20': 'IRC_PAM_SB0665_bin_20', 'IRC1_bin_23': 'IRC1_bin_23', 'COS3_bin_1': 'COS3_bin_1', 'RHO2_bin_40': 'RHO2_bin_40', 'COS4_bin_10': 'COS4_bin_10', 'COS36387_bin_10': 'COS36387_bin_10', 'GCA_000583135': 'HALC_tianQian2014_GCA_000583135.1_Thioalkalivibrio_spongium_HK1_genomic', 'RHO3_bin_50': 'RHO3_bin_50', 'IRC_PAM_SB0666_bin_15': 'IRC_PAM_SB0666_bin_15', 'COS36387_bin_7': 'COS36387_bin_7', 'GCF_900141785': 'HALO_yoonOh_GCF_900141785.1_IMGID2622736502_genomic', 'COS4_bin_26': 'COS4_bin_26', 'RHO2_bin_41': 'RHO2_bin_41', 'APA_bin_71': 'APA_bin_71', 'COS1_bin_18': 'COS1_bin_18', 'COS36406_bin_18': 'COS36406_bin_18', 'IRC_PAM_SB0662_bin_19': 'IRC_PAM_SB0662_bin_19', 'APA_bin_53': 'APA_bin_53', 'IRC_PAM_SB0666_bin_9': 'IRC_PAM_SB0666_bin_9', 'RHO2_bin_8': 'RHO2_bin_8', 'RHO2_bin_31': 'RHO2_bin_31', 'STY1_bin_1': 'STY1_bin_1', 'APA_bin_56': 'APA_bin_56', 'APA_bin_89': 'APA_bin_89', 'RHO2_bin_62': 'RHO2_bin_62', 'IRC1_bin_38': 'IRC1_bin_38', 'COS2_bin_8': 'COS2_bin_8', 'GCA_000522425': 'THES_wilsonPiel_GCA_000522425.1_v3_genomic', 'seawater_bettina_36328_bin_7': 'seawater_bettina_36328_bin_7', 'IRC4_bin_40': 'IRC4_bin_40', 'Pseudovibrio_spAU243': 'CYMC_estevesThomas2016_Pseudovibrio_spAU243_2606217185', 'IRC_PAM_SB0668_bin_14': 'IRC_PAM_SB0668_bin_14', 'COS4_bin_25': 'COS4_bin_25', 'RHO1_bin_8': 'RHO1_bin_8', 'GCA_001543015': 'LOPHE_tianQian_GCA_001543015.1_ASM154301v1_genomic', 'GCA_001007665': 'THES_burgsdorfSteindler_GCA_001007665.1_ASM100766v1_genomic', 'IRC_PAM_SB0664_bin_12': 'IRC_PAM_SB0664_bin_12', 'COS1_bin_3': 'COS1_bin_3', 'IRC4_bin_15': 'IRC4_bin_15', 'CAR2_bin_1': 'CAR2_bin_1', 'IRC3_bin_15': 'IRC3_bin_15', 'CAR1_bin_18': 'CAR1_bin_18', 'RHO1_bin_12': 'RHO1_bin_12', 'IRC_PAM_SB0665_bin_11': 'IRC_PAM_SB0665_bin_11', 'RHO2_bin_22': 'RHO2_bin_22', 'RHO1_bin_20': 'RHO1_bin_20', 'STY4_bin_3': 'STY4_bin_3', 'IRC4_bin_9': 'IRC4_bin_9', 'STY1_bin_3': 'STY1_bin_3', 'COS1_bin_5': 'COS1_bin_5', 'seawater_seasim_SB9156_S5_bin_20': 'seawater_seasim_SB9156_S5_bin_20', 'RHO2_bin_25': 'RHO2_bin_25', 'IRC_PAM_SB0667_bin_14': 'IRC_PAM_SB0667_bin_14', 'RHO2_bin_13': 'RHO2_bin_13', 'IRC_PAM_SB0662_bin_23': 'IRC_PAM_SB0662_bin_23', 'seawater_seasim_SB9157_S6_bin_7': 'seawater_seasim_SB9157_S6_bin_7', 'RHO3_bin_8': 'RHO3_bin_8', 'seawater_22112_bin_8': 'seawater_22112_bin_8', 'IRC_PAM_SB0664_bin_33': 'IRC_PAM_SB0664_bin_33', 'COS36386_bin_37': 'COS36386_bin_37', 'RHO2_bin_5': 'RHO2_bin_5', 'RHO2_bin_19': 'RHO2_bin_19', 'IRC_PAM_SB0662_bin_24': 'IRC_PAM_SB0662_bin_24', 'GCF_000743705': 'CRAC_dobervaLami_GCF_000743705.1_ASM74370v1_genomic', 'seawater_42618_bin_9': 'seawater_42618_bin_9', 'IRC4_bin_49': 'IRC4_bin_49', 'IRC_PAM_SB0668_bin_21': 'IRC_PAM_SB0668_bin_21', 'APA_bin_28': 'APA_bin_28', 'COS36406_bin_2': 'COS36406_bin_2', 'APA_bin_61': 'APA_bin_61', 'IRC_PAM_SB0678_bin_2': 'IRC_PAM_SB0678_bin_2', 'COS3_bin_17': 'COS3_bin_17', 'IRC_PAM_SB0664_bin_11': 'IRC_PAM_SB0664_bin_11', 'seawater_seasim_SB9152_S1_bin_2': 'seawater_seasim_SB9152_S1_bin_2', 'seawater_22112_bin_9': 'seawater_22112_bin_9', 'RHO3_bin_33': 'RHO3_bin_33', 'IRC4_bin_39': 'IRC4_bin_39', 'SPOO_karimiCosta_FZLR01': 'SPOO_karimiCosta_FZLR01', 'RHO1_bin_82': 'RHO1_bin_82', 'CAR4_bin_13': 'CAR4_bin_13', 'IRC3_bin_17': 'IRC3_bin_17', 'CAR2_bin_22': 'CAR2_bin_22', 'SPOO_karimiCosta_FZLQ01': 'SPOO_karimiCosta_FZLQ01', 'IRC_PAM_SB0675_bin_22': 'IRC_PAM_SB0675_bin_22', 'IRC_PAM_SB0664_bin_6': 'IRC_PAM_SB0664_bin_6', 'APA_bin_1': 'APA_bin_1', 'RHO1_bin_51': 'RHO1_bin_51', 'COS36386_bin_31': 'COS36386_bin_31', 'IRC2_bin_12': 'IRC2_bin_12', 'APA_bin_74': 'APA_bin_74', 'IRC_PAM_SB0661_bin_34': 'IRC_PAM_SB0661_bin_34', 'APA_bin_8': 'APA_bin_8', 'COS36406_bin_1': 'COS36406_bin_1', 'RHO3_bin_4': 'RHO3_bin_4', 'CAR4_bin_4': 'CAR4_bin_4', 'RHO2_bin_27': 'RHO2_bin_27', 'COS36406_bin_9': 'COS36406_bin_9', 'COS36388_bin_1': 'COS36388_bin_1', 'IRC2_bin_1': 'IRC2_bin_1', 'COS36388_bin_16': 'COS36388_bin_16', 'APA_bin_23': 'APA_bin_23', 'APA_bin_62': 'APA_bin_62', 'CAR1_bin_16': 'CAR1_bin_16', 'IRC2_bin_7': 'IRC2_bin_7', 'CAR1_bin_4': 'CAR1_bin_4', 'IRC1_bin_19': 'IRC1_bin_19', 'CAR3_bin_9': 'CAR3_bin_9', 'COS4_bin_49': 'COS4_bin_49', 'IRC3_bin_37': 'IRC3_bin_37', 'CAR3_bin_15': 'CAR3_bin_15', 'RHO1_bin_55': 'RHO1_bin_55', 'RHO1_bin_59': 'RHO1_bin_59', 'IRC_PAM_SB0661_bin_1': 'IRC_PAM_SB0661_bin_1', 'CAR3_bin_10': 'CAR3_bin_10', 'COS4_bin_12': 'COS4_bin_12', 'seawater_seasim_SB9152_S1_bin_18': 'seawater_seasim_SB9152_S1_bin_18', 'seawater_bettina_36327_bin_6': 'seawater_bettina_36327_bin_6', 'IRC3_bin_8': 'IRC3_bin_8', 'IRC4_bin_17': 'IRC4_bin_17', 'CAR1_bin_5': 'CAR1_bin_5', 'RHO3_bin_22': 'RHO3_bin_22', 'IRC_PAM_SB0675_bin_3': 'IRC_PAM_SB0675_bin_3', 'seawater_seasim_SB9154_S3_bin_1': 'seawater_seasim_SB9154_S3_bin_1', 'COS4_bin_1': 'COS4_bin_1', 'GCA_000200715': 'AXIM_hallam_GCA_000200715.1_genomic', 'IRC_PAM_SB0661_bin_40': 'IRC_PAM_SB0661_bin_40', 'RHO1_bin_52': 'RHO1_bin_52', 'IRC4_bin_30': 'IRC4_bin_30', 'RHO1_bin_17': 'RHO1_bin_17', 'COS3_bin_16': 'COS3_bin_16', 'GCF_900079515': 'SPOO_alexAntunes_GCF_900079515.1_Shewanella_sp.Alg231_23_genomic', 'GCF_000156235': 'MYCL_bondarevVogt_GCF_000156235.1_ASM15623v1_genomic', 'seawater_42615_bin_16': 'seawater_42615_bin_16', 'RHO3_bin_19': 'RHO3_bin_19', 'CLI1_bin_2': 'CLI1_bin_2', 'IRC1_bin_31': 'IRC1_bin_31', 'IRC_PAM_SB0665_bin_17': 'IRC_PAM_SB0665_bin_17', 'seawater_bettina_36327_bin_3': 'seawater_bettina_36327_bin_3', 'COS36387_bin_14': 'COS36387_bin_14', 'COS4_bin_9': 'COS4_bin_9', 'seawater_bettina_36308_bin_18': 'seawater_bettina_36308_bin_18', 'COS1_bin_16': 'COS1_bin_16', 'RHO2_bin_17': 'RHO2_bin_17', 'COS36404_bin_5': 'COS36404_bin_5', 'STY1_bin_7': 'STY1_bin_7', 'IRC_PAM_SB0662_bin_30': 'IRC_PAM_SB0662_bin_30', 'RHO2_bin_2': 'RHO2_bin_2', 'IRC_PAM_SB0662_bin_1': 'IRC_PAM_SB0662_bin_1', 'RHO3_bin_29': 'RHO3_bin_29', 'COS36388_bin_3': 'COS36388_bin_3', 'seawater_seasim_SB9154_S3_bin_3': 'seawater_seasim_SB9154_S3_bin_3', 'IRC_PAM_SB0664_bin_7': 'IRC_PAM_SB0664_bin_7', 'COS36387_bin_15': 'COS36387_bin_15', 'IRC4_bin_11': 'IRC4_bin_11', 'IRC4_bin_42': 'IRC4_bin_42', 'IRC4_bin_13': 'IRC4_bin_13', 'RHO1_bin_43': 'RHO1_bin_43', 'CAR2_bin_19': 'CAR2_bin_19', 'IRC1_bin_9': 'IRC1_bin_9', 'IRC3_bin_3': 'IRC3_bin_3', 'IRC_PAM_SB0675_bin_23': 'IRC_PAM_SB0675_bin_23', 'seawater_seasim_SB9156_S5_bin_3': 'seawater_seasim_SB9156_S5_bin_3', 'RHO3_bin_57': 'RHO3_bin_57', 'IRC_PAM_SB0662_bin_34': 'IRC_PAM_SB0662_bin_34', 'CAR3_bin_17': 'CAR3_bin_17', 'IRC_PAM_SB0672_bin_21': 'IRC_PAM_SB0672_bin_21', 'RHO2_bin_39': 'RHO2_bin_39', 'COS4_bin_18': 'COS4_bin_18', 'IRC_PAM_SB0668_bin_13': 'IRC_PAM_SB0668_bin_13', 'seawater_22112_bin_4': 'seawater_22112_bin_4', 'IRC_PAM_SB0665_bin_16': 'IRC_PAM_SB0665_bin_16', 'COS3_bin_15': 'COS3_bin_15', 'RHO1_bin_69': 'RHO1_bin_69', 'RHO1_bin_26': 'RHO1_bin_26', 'IRC_PAM_SB0661_bin_39': 'IRC_PAM_SB0661_bin_39', 'COS36386_bin_5': 'COS36386_bin_5', 'seawater_seasim_SB9153_S2_bin_13': 'seawater_seasim_SB9153_S2_bin_13', 'GCF_000264395': 'DYSA_liuLi2012_GCF_000264395.1_C89_version_1_genomic', 'RHO2_bin_56': 'RHO2_bin_56', 'APA_bin_6': 'APA_bin_6', 'IRC_PAM_SB0661_bin_6': 'IRC_PAM_SB0661_bin_6', 'seawater_bettina_36327_bin_7': 'seawater_bettina_36327_bin_7', 'CHO1_bin_4': 'CHO1_bin_4', 'APA_bin_10': 'APA_bin_10', 'RHO1_bin_10': 'RHO1_bin_10', 'IRC_PAM_SB0668_bin_7': 'IRC_PAM_SB0668_bin_7', 'COS36387_bin_11': 'COS36387_bin_11', 'IRC_PAM_SB0670_bin_19': 'IRC_PAM_SB0670_bin_19', 'RHO1_bin_29': 'RHO1_bin_29', 'COS36405_bin_1': 'COS36405_bin_1', 'COS1_bin_21': 'COS1_bin_21', 'IRC_PAM_SB0661_bin_55': 'IRC_PAM_SB0661_bin_55', 'COS4_bin_36': 'COS4_bin_36', 'STY2_bin_1': 'STY2_bin_1', 'IRC_PAM_SB0662_bin_39': 'IRC_PAM_SB0662_bin_39', 'RHO2_bin_55': 'RHO2_bin_55', 'APA_bin_97': 'APA_bin_97', 'COS4_bin_46': 'COS4_bin_46', 'COS4_bin_15': 'COS4_bin_15', 'IRC_PAM_SB0664_bin_2': 'IRC_PAM_SB0664_bin_2', 'IRC3_bin_2': 'IRC3_bin_2', 'seawater_seasim_SB9155_S4_bin_4': 'seawater_seasim_SB9155_S4_bin_4', 'seawater_42617_bin_6': 'seawater_42617_bin_6', 'COS36386_bin_14': 'COS36386_bin_14', 'IRC_PAM_SB0675_bin_16': 'IRC_PAM_SB0675_bin_16', 'IRC_PAM_SB0667_bin_13': 'IRC_PAM_SB0667_bin_13', 'RHO2_bin_6': 'RHO2_bin_6', 'COS36406_bin_7': 'COS36406_bin_7', 'IRC1_bin_26': 'IRC1_bin_26', 'RHO3_bin_1': 'RHO3_bin_1', 'IRC_PAM_SB0662_bin_10': 'IRC_PAM_SB0662_bin_10', 'RHO3_bin_24': 'RHO3_bin_24', 'IRC4_bin_16': 'IRC4_bin_16', 'APA_bin_22': 'APA_bin_22', 'CAR1_bin_7': 'CAR1_bin_7', 'RHO3_bin_80': 'RHO3_bin_80', 'seawater_42618_bin_6': 'seawater_42618_bin_6', 'COS36387_bin_3': 'COS36387_bin_3', 'CAR1_bin_15': 'CAR1_bin_15', 'RHO3_bin_25': 'RHO3_bin_25', 'CAR1_bin_14': 'CAR1_bin_14', 'STY1_bin_2': 'STY1_bin_2', 'APA_bin_3': 'APA_bin_3', 'RHO1_bin_24': 'RHO1_bin_24', 'IRC1_bin_20': 'IRC1_bin_20', 'IRC_PAM_SB0668_bin_5': 'IRC_PAM_SB0668_bin_5', 'seawater_22112_bin_2': 'seawater_22112_bin_2', 'IRC3_bin_16': 'IRC3_bin_16', 'RHO3_bin_23': 'RHO3_bin_23', 'CAR3_bin_14': 'CAR3_bin_14', 'CAR4_bin_16': 'CAR4_bin_16', 'CAR4_bin_1': 'CAR4_bin_1', 'GCF_001431305': 'POLP_alexAntunes_GCF_001431305.1_ASM143130v1_genomic', 'COS36405_bin_8': 'COS36405_bin_8', 'COS4_bin_23': 'COS4_bin_23', 'GCF_900143565': 'SPOO_karimiCosta2019_GCF_900143565.1_Pseudovibrio_sp._Alg231_02_genomic', 'APA_bin_19': 'APA_bin_19', 'RHO3_bin_49': 'RHO3_bin_49', 'CAR3_bin_11': 'CAR3_bin_11', 'COS1_bin_9': 'COS1_bin_9', 'RHO1_bin_68': 'RHO1_bin_68', 'IRC3_bin_21': 'IRC3_bin_21', 'IRC_PAM_SB0666_bin_11': 'IRC_PAM_SB0666_bin_11', 'RHO3_bin_74': 'RHO3_bin_74', 'RHO1_bin_31': 'RHO1_bin_31', 'COS36405_bin_12': 'COS36405_bin_12', 'GCA_003635195': 'PSE_podellAllen_GCA_003635195.1_ASM363519v1_genomic', 'RHO3_bin_43': 'RHO3_bin_43', 'seawater_seasim_SB9153_S2_bin_4': 'seawater_seasim_SB9153_S2_bin_4', 'IRC_PAM_SB0664_bin_28': 'IRC_PAM_SB0664_bin_28', 'RHO3_bin_73': 'RHO3_bin_73', 'IRC_PAM_SB0661_bin_27': 'IRC_PAM_SB0661_bin_27', 'COS36404_bin_3': 'COS36404_bin_3'}

raw2new_name_dict = {'CAR4_bin_6': 'CAR4_bin_6', 'COS2_bin_4': 'COS2_bin_4', 'COS4_bin_21': 'COS4_bin_21', 'COS2_bin_10': 'COS2_bin_10', 'IRC4_bin_41': 'IRC4_bin_41', 'IRC_PAM_SB0675_bin_5': 'IRC_PAM_SB0675_bin_5', 'RHO2_bin_49': 'RHO2_bin_49', 'IRC_PAM_SB0665_bin_25': 'IRC_PAM_SB0665_bin_25', 'COS2_bin_7': 'COS2_bin_7', 'IRC_PAM_SB0667_bin_1': 'IRC_PAM_SB0667_bin_1', 'RHO3_bin_84': 'RHO3_bin_84', 'IRC3_bin_26': 'IRC3_bin_26', 'IRC3_bin_28': 'IRC3_bin_28', 'RHO1_bin_56': 'RHO1_bin_56', 'IRC_PAM_SB0668_bin_19': 'IRC_PAM_SB0668_bin_19', 'IRC_PAM_SB0666_bin_34': 'IRC_PAM_SB0666_bin_34', 'seawater_bettina_36328_bin_3': 'seawater_bettina_36328_bin_3', 'COS36405_bin_3': 'COS36405_bin_3', 'RHO1_bin_37': 'RHO1_bin_37', 'PSE_podellAllen_GCA_003635205.1_ASM363520v1_genomic': 'GCA_003635205', 'IRC_PAM_SB0661_bin_2': 'IRC_PAM_SB0661_bin_2', 'RHO1_bin_65': 'RHO1_bin_65', 'IRC_PAM_SB0665_bin_4': 'IRC_PAM_SB0665_bin_4', 'IRC3_bin_18': 'IRC3_bin_18', 'RHO1_bin_53': 'RHO1_bin_53', 'COS3_bin_9': 'COS3_bin_9', 'RHO2_bin_42': 'RHO2_bin_42', 'CAR1_bin_10': 'CAR1_bin_10', 'IRC_PAM_SB0661_bin_17': 'IRC_PAM_SB0661_bin_17', 'IRC4_bin_3': 'IRC4_bin_3', 'COS36388_bin_13': 'COS36388_bin_13', 'APA_bin_59': 'APA_bin_59', 'APA_bin_42': 'APA_bin_42', 'IRC_PAM_SB0665_bin_27': 'IRC_PAM_SB0665_bin_27', 'CAR2_bin_5': 'CAR2_bin_5', 'seawater_bettina_36310_bin_3': 'seawater_bettina_36310_bin_3', 'OPHP_alexAntunes_GCF_004168585.1_ASM416858v1_genomic': 'GCF_004168585', 'IRC4_bin_29': 'IRC4_bin_29', 'IRC_PAM_SB0661_bin_5': 'IRC_PAM_SB0661_bin_5', 'COS36387_bin_19': 'COS36387_bin_19', 'IRC_PAM_SB0661_bin_22': 'IRC_PAM_SB0661_bin_22', 'SPOO_alexAntunes_GCF_900079515.1_Shewanella_sp.Alg231_23_genomic': 'GCF_900079515', 'seawater_22112_bin_25': 'seawater_22112_bin_25', 'RHO3_bin_32': 'RHO3_bin_32', 'IRC_PAM_SB0665_bin_9': 'IRC_PAM_SB0665_bin_9', 'IRC1_bin_14': 'IRC1_bin_14', 'CAR2_bin_13': 'CAR2_bin_13', 'COS36388_bin_11': 'COS36388_bin_11', 'IRC_PAM_SB0661_bin_16': 'IRC_PAM_SB0661_bin_16', 'APA_bin_13': 'APA_bin_13', 'IRC_PAM_SB0677_bin_19': 'IRC_PAM_SB0677_bin_19', 'COS36386_bin_13': 'COS36386_bin_13', 'RHO3_bin_28': 'RHO3_bin_28', 'APA_bin_102': 'APA_bin_102', 'CAR1_bin_9': 'CAR1_bin_9', 'APA_bin_91': 'APA_bin_91', 'COS4_bin_42': 'COS4_bin_42', 'COS1_bin_11': 'COS1_bin_11', 'APA_bin_52': 'APA_bin_52', 'COS36386_bin_2': 'COS36386_bin_2', 'RHO2_bin_15': 'RHO2_bin_15', 'IRC_PAM_SB0664_bin_16': 'IRC_PAM_SB0664_bin_16', 'RHO3_bin_59': 'RHO3_bin_59', 'RHO1_bin_25': 'RHO1_bin_25', 'CLI1_bin_3': 'CLI1_bin_3', 'IRC1_bin_27': 'IRC1_bin_27', 'STY1_bin_6': 'STY1_bin_6', 'IRC_PAM_SB0675_bin_4': 'IRC_PAM_SB0675_bin_4', 'COS4_bin_7': 'COS4_bin_7', 'APA_bin_93': 'APA_bin_93', 'RHO1_bin_13': 'RHO1_bin_13', 'IRC_PAM_SB0677_bin_15': 'IRC_PAM_SB0677_bin_15', 'STY3_bin_6': 'STY3_bin_6', 'APA_bin_5': 'APA_bin_5', 'IRC_PAM_SB0673_bin_10': 'IRC_PAM_SB0673_bin_10', 'seawater_bettina_36309_bin_5': 'seawater_bettina_36309_bin_5', 'IRC_PAM_SB0678_bin_5': 'IRC_PAM_SB0678_bin_5', 'COS36404_bin_12': 'COS36404_bin_12', 'IRC_PAM_SB0677_bin_1': 'IRC_PAM_SB0677_bin_1', 'COS36387_bin_20': 'COS36387_bin_20', 'CAR2_bin_10': 'CAR2_bin_10', 'IRC_PAM_SB0662_bin_27': 'IRC_PAM_SB0662_bin_27', 'IRC1_bin_21': 'IRC1_bin_21', 'IRC1_bin_34': 'IRC1_bin_34', 'IRC_PAM_SB0678_bin_7': 'IRC_PAM_SB0678_bin_7', 'STY1_bin_5': 'STY1_bin_5', 'IRC_PAM_SB0675_bin_1': 'IRC_PAM_SB0675_bin_1', 'RHO3_bin_71': 'RHO3_bin_71', 'IRC4_bin_7': 'IRC4_bin_7', 'IRC_PAM_SB0677_bin_16': 'IRC_PAM_SB0677_bin_16', 'IRC2_bin_10': 'IRC2_bin_10', 'seawater_22112_bin_29': 'seawater_22112_bin_29', 'COS36386_bin_32': 'COS36386_bin_32', 'seawater_seasim_SB9160_S9_bin_9': 'seawater_seasim_SB9160_S9_bin_9', 'seawater_bettina_36328_bin_8': 'seawater_bettina_36328_bin_8', 'seawater_seasim_SB9156_S5_bin_6': 'seawater_seasim_SB9156_S5_bin_6', 'IRC_PAM_SB0664_bin_4': 'IRC_PAM_SB0664_bin_4', 'RHO1_bin_23': 'RHO1_bin_23', 'COS36388_bin_15': 'COS36388_bin_15', 'COS2_bin_3': 'COS2_bin_3', 'CHO1_bin_2': 'CHO1_bin_2', 'APA_bin_80': 'APA_bin_80', 'COS2_bin_6': 'COS2_bin_6', 'CLI1_bin_1': 'CLI1_bin_1', 'seawater_42618_bin_29': 'seawater_42618_bin_29', 'COS3_bin_6': 'COS3_bin_6', 'seawater_seasim_SB9152_S1_bin_8': 'seawater_seasim_SB9152_S1_bin_8', 'seawater_bettina_36308_bin_5': 'seawater_bettina_36308_bin_5', 'IRC_PAM_SB0677_bin_11': 'IRC_PAM_SB0677_bin_11', 'RHO1_bin_18': 'RHO1_bin_18', 'RHO3_bin_40': 'RHO3_bin_40', 'IRC_PAM_SB0662_bin_11': 'IRC_PAM_SB0662_bin_11', 'IRC2_bin_3': 'IRC2_bin_3', 'IRC3_bin_13': 'IRC3_bin_13', 'RHO3_bin_36': 'RHO3_bin_36', 'DYSA_liuLi2012_GCF_000264395.1_C89_version_1_genomic': 'GCF_000264395', 'IRC2_bin_11': 'IRC2_bin_11', 'seawater_22112_bin_6': 'seawater_22112_bin_6', 'RHO2_bin_67': 'RHO2_bin_67', 'COS1_bin_8': 'COS1_bin_8', 'CAR1_bin_1': 'CAR1_bin_1', 'IRC_PAM_SB0662_bin_7': 'IRC_PAM_SB0662_bin_7', 'IRC_PAM_SB0668_bin_1': 'IRC_PAM_SB0668_bin_1', 'IRC_PAM_SB0677_bin_7': 'IRC_PAM_SB0677_bin_7', 'seawater_22112_bin_7': 'seawater_22112_bin_7', 'COS1_bin_4': 'COS1_bin_4', 'COS3_bin_8': 'COS3_bin_8', 'seawater_42617_bin_7': 'seawater_42617_bin_7', 'seawater_42618_bin_3': 'seawater_42618_bin_3', 'IRC2_bin_5': 'IRC2_bin_5', 'RHO2_bin_36': 'RHO2_bin_36', 'COS36386_bin_1': 'COS36386_bin_1', 'APA_bin_27': 'APA_bin_27', 'CAR2_bin_16': 'CAR2_bin_16', 'COS2_bin_19': 'COS2_bin_19', 'seawater_22112_bin_16': 'seawater_22112_bin_16', 'RHO1_bin_3': 'RHO1_bin_3', 'RHO3_bin_65': 'RHO3_bin_65', 'RHO3_bin_67': 'RHO3_bin_67', 'IRC4_bin_14': 'IRC4_bin_14', 'CLI3_bin_1': 'CLI3_bin_1', 'RHO3_bin_68': 'RHO3_bin_68', 'CAR2_bin_3': 'CAR2_bin_3', 'COS4_bin_6': 'COS4_bin_6', 'IRC1_bin_13': 'IRC1_bin_13', 'CAR2_bin_18': 'CAR2_bin_18', 'RHO2_bin_13': 'RHO2_bin_13', 'LOPHE_tianQian_GCA_001543015.1_ASM154301v1_genomic': 'GCA_001543015', 'COS36388_bin_6': 'COS36388_bin_6', 'seawater_bettina_36308_bin_3': 'seawater_bettina_36308_bin_3', 'CAR2_bin_15': 'CAR2_bin_15', 'COS3_bin_13': 'COS3_bin_13', 'CLI2_bin_1': 'CLI2_bin_1', 'CAR4_bin_5': 'CAR4_bin_5', 'seawater_22112_bin_15': 'seawater_22112_bin_15', 'IRC4_bin_10': 'IRC4_bin_10', 'RHO3_bin_53': 'RHO3_bin_53', 'RHO1_bin_85': 'RHO1_bin_85', 'seawater_seasim_SB9152_S1_bin_3': 'seawater_seasim_SB9152_S1_bin_3', 'IRC_PAM_SB0665_bin_24': 'IRC_PAM_SB0665_bin_24', 'RHO2_bin_30': 'RHO2_bin_30', 'IRC_PAM_SB0662_bin_9': 'IRC_PAM_SB0662_bin_9', 'IRC_PAM_SB0664_bin_21': 'IRC_PAM_SB0664_bin_21', 'CAR3_bin_12': 'CAR3_bin_12', 'IRC4_bin_38': 'IRC4_bin_38', 'CAR4_bin_3': 'CAR4_bin_3', 'RHO3_bin_34': 'RHO3_bin_34', 'COS36387_bin_4': 'COS36387_bin_4', 'IRC1_bin_12': 'IRC1_bin_12', 'COS4_bin_40': 'COS4_bin_40', 'RHO1_bin_19': 'RHO1_bin_19', 'COS4_bin_24': 'COS4_bin_24', 'COS36386_bin_17': 'COS36386_bin_17', 'IRC_PAM_SB0675_bin_10': 'IRC_PAM_SB0675_bin_10', 'APA_bin_73': 'APA_bin_73', 'RHO2_bin_1': 'RHO2_bin_1', 'COS36386_bin_27': 'COS36386_bin_27', 'LOPHE_tianQian_GCA_001542995.1_ASM154299v1_genomic': 'GCA_001542995', 'IRC_PAM_SB0665_bin_10': 'IRC_PAM_SB0665_bin_10', 'RHO3_bin_13': 'RHO3_bin_13', 'IRC_PAM_SB0665_bin_13': 'IRC_PAM_SB0665_bin_13', 'APA_bin_24': 'APA_bin_24', 'RHO3_bin_64': 'RHO3_bin_64', 'IRC_PAM_SB0668_bin_6': 'IRC_PAM_SB0668_bin_6', 'RHO1_bin_62': 'RHO1_bin_62', 'IRC_PAM_SB0661_bin_45': 'IRC_PAM_SB0661_bin_45', 'IRC_PAM_SB0662_bin_26': 'IRC_PAM_SB0662_bin_26', 'COS1_bin_19': 'COS1_bin_19', 'RHO3_bin_11': 'RHO3_bin_11', 'RHO1_bin_46': 'RHO1_bin_46', 'TED_braunBugni_GCF_002573675.1_ASM257367v1_genomic': 'GCF_002573675', 'RHO1_bin_14': 'RHO1_bin_14', 'IRC_PAM_SB0664_bin_31': 'IRC_PAM_SB0664_bin_31', 'IRC2_bin_6': 'IRC2_bin_6', 'COS1_bin_17': 'COS1_bin_17', 'IRC3_bin_19': 'IRC3_bin_19', 'RHO1_bin_45': 'RHO1_bin_45', 'CAR4_bin_8': 'CAR4_bin_8', 'RHO2_bin_65': 'RHO2_bin_65', 'RHO2_bin_9': 'RHO2_bin_9', 'seawater_22112_bin_5': 'seawater_22112_bin_5', 'IRC_PAM_SB0670_bin_12': 'IRC_PAM_SB0670_bin_12', 'CAR4_bin_10': 'CAR4_bin_10', 'RHO3_bin_16': 'RHO3_bin_16', 'IRC_PAM_SB0677_bin_2': 'IRC_PAM_SB0677_bin_2', 'IRC4_bin_6': 'IRC4_bin_6', 'RHO1_bin_48': 'RHO1_bin_48', 'IRC_PAM_SB0665_bin_1': 'IRC_PAM_SB0665_bin_1', 'APA_bin_14': 'APA_bin_14', 'COS4_bin_5': 'COS4_bin_5', 'IRC_PAM_SB0662_bin_20': 'IRC_PAM_SB0662_bin_20', 'COS1_bin_6': 'COS1_bin_6', 'IRC_PAM_SB0661_bin_4': 'IRC_PAM_SB0661_bin_4', 'RHO1_bin_1': 'RHO1_bin_1', 'RHO3_bin_31': 'RHO3_bin_31', 'RHO2_bin_51': 'RHO2_bin_51', 'HALC_tianQian2014_GCA_000583135.1_Thioalkalivibrio_spongium_HK1_genomic': 'GCA_000583135', 'IRC3_bin_31': 'IRC3_bin_31', 'IRC_PAM_SB0661_bin_37': 'IRC_PAM_SB0661_bin_37', 'CAR3_bin_6': 'CAR3_bin_6', 'COS36387_bin_6': 'COS36387_bin_6', 'RHO2_bin_37': 'RHO2_bin_37', 'COS4_bin_3': 'COS4_bin_3', 'COS3_bin_2': 'COS3_bin_2', 'CHO1_bin_1': 'CHO1_bin_1', 'COS4_bin_19': 'COS4_bin_19', 'IRC_PAM_SB0662_bin_33': 'IRC_PAM_SB0662_bin_33', 'THES_burgsdorfSteindler_GCA_001007665.1_ASM100766v1_genomic': 'GCA_001007665', 'IRC_PAM_SB0675_bin_19': 'IRC_PAM_SB0675_bin_19', 'COS36404_bin_1': 'COS36404_bin_1', 'RHO3_bin_58': 'RHO3_bin_58', 'CAR3_bin_1': 'CAR3_bin_1', 'PSE_podellAllen_GCA_003635195.1_ASM363519v1_genomic': 'GCA_003635195', 'IRC_PAM_SB0662_bin_5': 'IRC_PAM_SB0662_bin_5', 'IRC_PAM_SB0662_bin_22': 'IRC_PAM_SB0662_bin_22', 'APA_bin_26': 'APA_bin_26', 'CAR4_bin_18': 'CAR4_bin_18', 'COS36386_bin_19': 'COS36386_bin_19', 'IRC_PAM_SB0661_bin_41': 'IRC_PAM_SB0661_bin_41', 'IRC_PAM_SB0664_bin_25': 'IRC_PAM_SB0664_bin_25', 'IRC_PAM_SB0670_bin_20': 'IRC_PAM_SB0670_bin_20', 'SUB_tianQian_GCA_002007405.1_ASM200740v1_genomic': 'GCA_002007405', 'APA_bin_81': 'APA_bin_81', 'IRC_PAM_SB0676_bin_21': 'IRC_PAM_SB0676_bin_21', 'COS4_bin_43': 'COS4_bin_43', 'RHO2_bin_18': 'RHO2_bin_18', 'APA_bin_33': 'APA_bin_33', 'CAR2_bin_20': 'CAR2_bin_20', 'seawater_bettina_36309_bin_17': 'seawater_bettina_36309_bin_17', 'CAR2_bin_2': 'CAR2_bin_2', 'IRC_PAM_SB0664_bin_27': 'IRC_PAM_SB0664_bin_27', 'seawater_bettina_36310_bin_4': 'seawater_bettina_36310_bin_4', 'COS1_bin_20': 'COS1_bin_20', 'IRC_PAM_SB0662_bin_58': 'IRC_PAM_SB0662_bin_58', 'CAR1_bin_12': 'CAR1_bin_12', 'seawater_22112_bin_10': 'seawater_22112_bin_10', 'CAR1_bin_3': 'CAR1_bin_3', 'IRC4_bin_8': 'IRC4_bin_8', 'IRC_PAM_SB0675_bin_2': 'IRC_PAM_SB0675_bin_2', 'IRC_PAM_SB0665_bin_2': 'IRC_PAM_SB0665_bin_2', 'IRC_PAM_SB0670_bin_22': 'IRC_PAM_SB0670_bin_22', 'IRC_PAM_SB0664_bin_22': 'IRC_PAM_SB0664_bin_22', 'seawater_seasim_SB9152_S1_bin_1': 'seawater_seasim_SB9152_S1_bin_1', 'RHO3_bin_69': 'RHO3_bin_69', 'RHO1_bin_44': 'RHO1_bin_44', 'seawater_seasim_SB9153_S2_bin_2': 'seawater_seasim_SB9153_S2_bin_2', 'IRC_PAM_SB0666_bin_17': 'IRC_PAM_SB0666_bin_17', 'MELS_podellAllen_GCA_GCA_003635315.1_ASM363531v1_genomic': 'GCA_003635315', 'IRC_PAM_SB0661_bin_3': 'IRC_PAM_SB0661_bin_3', 'IRC_PAM_SB0673_bin_16': 'IRC_PAM_SB0673_bin_16', 'IRC_PAM_SB0662_bin_35': 'IRC_PAM_SB0662_bin_35', 'RHO3_bin_35': 'RHO3_bin_35', 'COS2_bin_17': 'COS2_bin_17', 'unknown_alexAntunes_GCF_003676335.1_ASM367633v1_genomic': 'GCF_003676335', 'IRC4_bin_23': 'IRC4_bin_23', 'PSE_podellAllen_GCA_003635255.1_ASM363525v1_genomic': 'GCA_003635255', 'IRC4_bin_24': 'IRC4_bin_24', 'seawater_22112_bin_28': 'seawater_22112_bin_28', 'CAR2_bin_7': 'CAR2_bin_7', 'APA_bin_94': 'APA_bin_94', 'APA_bin_85': 'APA_bin_85', 'IRC1_bin_17': 'IRC1_bin_17', 'COS36404_bin_9': 'COS36404_bin_9', 'COS36404_bin_2': 'COS36404_bin_2', 'CAR3_bin_16': 'CAR3_bin_16', 'IRC4_bin_44': 'IRC4_bin_44', 'seawater_22112_bin_3': 'seawater_22112_bin_3', 'CAR1_bin_11': 'CAR1_bin_11', 'APA_bin_17': 'APA_bin_17', 'RHO2_bin_59': 'RHO2_bin_59', 'seawater_seasim_SB9155_S4_bin_12': 'seawater_seasim_SB9155_S4_bin_12', 'RHO2_bin_33': 'RHO2_bin_33', 'TED_estevesThomas2016_Aquimarina_spAU474_2606217188': 'Aquimarina_spAU474', 'IRC1_bin_15': 'IRC1_bin_15', 'RHO2_bin_54': 'RHO2_bin_54', 'RHO3_bin_7': 'RHO3_bin_7', 'COS4_bin_44': 'COS4_bin_44', 'RHO3_bin_73': 'RHO3_bin_73', 'APA_bin_70': 'APA_bin_70', 'RHO2_bin_7': 'RHO2_bin_7', 'IRC_PAM_SB0664_bin_9': 'IRC_PAM_SB0664_bin_9', 'RHO3_bin_47': 'RHO3_bin_47', 'COS36387_bin_1': 'COS36387_bin_1', 'IRC3_bin_12': 'IRC3_bin_12', 'COS1_bin_12': 'COS1_bin_12', 'CAR4_bin_2': 'CAR4_bin_2', 'IRC4_bin_33': 'IRC4_bin_33', 'IRC_PAM_SB0666_bin_10': 'IRC_PAM_SB0666_bin_10', 'APA_bin_68': 'APA_bin_68', 'COS3_bin_18': 'COS3_bin_18', 'IRC_PAM_SB0678_bin_9': 'IRC_PAM_SB0678_bin_9', 'RHO1_bin_4': 'RHO1_bin_4', 'RHO3_bin_72': 'RHO3_bin_72', 'RHO1_bin_67': 'RHO1_bin_67', 'IRC_PAM_SB0661_bin_39': 'IRC_PAM_SB0661_bin_39', 'IRC4_bin_2': 'IRC4_bin_2', 'IRC_PAM_SB0676_bin_26': 'IRC_PAM_SB0676_bin_26', 'CAR4_bin_9': 'CAR4_bin_9', 'seawater_seasim_SB9160_S9_bin_11': 'seawater_seasim_SB9160_S9_bin_11', 'IRC_PAM_SB0675_bin_14': 'IRC_PAM_SB0675_bin_14', 'RHO1_bin_32': 'RHO1_bin_32', 'COS4_bin_2': 'COS4_bin_2', 'RHO2_bin_38': 'RHO2_bin_38', 'COS36387_bin_2': 'COS36387_bin_2', 'seawater_seasim_SB9155_S4_bin_4': 'seawater_seasim_SB9155_S4_bin_4', 'APA_bin_83': 'APA_bin_83', 'IRC_PAM_SB0661_bin_29': 'IRC_PAM_SB0661_bin_29', 'COS3_bin_12': 'COS3_bin_12', 'RHO3_bin_41': 'RHO3_bin_41', 'SUB_tianQian_GCA_001541925.1_ASM154192v1_genomic': 'GCA_001541925', 'RHO3_bin_38': 'RHO3_bin_38', 'STY4_bin_1': 'STY4_bin_1', 'IRC_PAM_SB0672_bin_11': 'IRC_PAM_SB0672_bin_11', 'STY3_bin_3': 'STY3_bin_3', 'IRC1_bin_1': 'IRC1_bin_1', 'IRC_PAM_SB0662_bin_12': 'IRC_PAM_SB0662_bin_12', 'seawater_seasim_SB9157_S6_bin_2': 'seawater_seasim_SB9157_S6_bin_2', 'RHO1_bin_40': 'RHO1_bin_40', 'IRC2_bin_8': 'IRC2_bin_8', 'seawater_22112_bin_14': 'seawater_22112_bin_14', 'seawater_42618_bin_2': 'seawater_42618_bin_2', 'COS4_bin_20': 'COS4_bin_20', 'IRC1_bin_3': 'IRC1_bin_3', 'RHO3_bin_66': 'RHO3_bin_66', 'CAR3_bin_18': 'CAR3_bin_18', 'COS1_bin_7': 'COS1_bin_7', 'CYMC_estevesThomas2016_Pseudovibrio_spAU243_2606217185': 'Pseudovibrio_spAU243', 'CYMC_moitinhoThomas_67496.assembled': 'CYMC_moitinhoThomas_67496', 'RHO2_bin_23': 'RHO2_bin_23', 'IRC1_bin_16': 'IRC1_bin_16', 'IRC1_bin_35': 'IRC1_bin_35', 'CAR2_bin_6': 'CAR2_bin_6', 'IRC3_bin_25': 'IRC3_bin_25', 'IRC1_bin_10': 'IRC1_bin_10', 'COS36404_bin_19': 'COS36404_bin_19', 'RHO3_bin_12': 'RHO3_bin_12', 'CAR3_bin_3': 'CAR3_bin_3', 'IRC4_bin_37': 'IRC4_bin_37', 'IRC_PAM_SB0662_bin_49': 'IRC_PAM_SB0662_bin_49', 'COS1_bin_1': 'COS1_bin_1', 'IRC_PAM_SB0662_bin_59': 'IRC_PAM_SB0662_bin_59', 'RHO3_bin_6': 'RHO3_bin_6', 'CAR2_bin_14': 'CAR2_bin_14', 'RHO3_bin_48': 'RHO3_bin_48', 'RHO3_bin_37': 'RHO3_bin_37', 'SPOO_karimiCosta2019_GCF_900143635.1_Rhodobacteraceae_bacterium_Alg231_04_genomic': 'GCF_900143635', 'COS4_bin_14': 'COS4_bin_14', 'APA_bin_4': 'APA_bin_4', 'RHO2_bin_24': 'RHO2_bin_24', 'APA_bin_82': 'APA_bin_82', 'APA_bin_38': 'APA_bin_38', 'MYCL_zanHill_GCF_000158135.1_ASM15813v1_genomic': 'GCF_000158135', 'PSE_podellAllen_GCA_003635265.1_ASM363526v1_genomic': 'GCA_003635265', 'IRC_PAM_SB0675_bin_20': 'IRC_PAM_SB0675_bin_20', 'RHO3_bin_5': 'RHO3_bin_5', 'IRC_PAM_SB0661_bin_38': 'IRC_PAM_SB0661_bin_38', 'COS3_bin_5': 'COS3_bin_5', 'RHO3_bin_10': 'RHO3_bin_10', 'CAR2_bin_9': 'CAR2_bin_9', 'IRC_PAM_SB0661_bin_15': 'IRC_PAM_SB0661_bin_15', 'APA_bin_90': 'APA_bin_90', 'SPOO_karimiCosta2019_GCF_900143555.1_Labrenzia_sp._Alg231_36_genomic': 'GCF_900143555', 'IRC_PAM_SB0670_bin_18': 'IRC_PAM_SB0670_bin_18', 'APA_bin_87': 'APA_bin_87', 'COS36405_bin_7': 'COS36405_bin_7', 'RHO2_bin_64': 'RHO2_bin_64', 'STY4_bin_2': 'STY4_bin_2', 'IRC2_bin_2': 'IRC2_bin_2', 'STY4_bin_9': 'STY4_bin_9', 'APA_bin_58': 'APA_bin_58', 'COS4_bin_32': 'COS4_bin_32', 'seawater_seasim_SB9156_S5_bin_8': 'seawater_seasim_SB9156_S5_bin_8', 'COS2_bin_22': 'COS2_bin_22', 'COS36405_bin_20': 'COS36405_bin_20', 'IRC_PAM_SB0662_bin_51': 'IRC_PAM_SB0662_bin_51', 'SPOO_karimiCosta2019_GCF_900143615.1_Rhodobacteraceae_bacterium_Alg231_30_genomic': 'GCF_900143615', 'POLP_alexAntunes_GCF_001431305.1_ASM143130v1_genomic': 'GCF_001431305', 'RHO3_bin_54': 'RHO3_bin_54', 'IRC_PAM_SB0666_bin_21': 'IRC_PAM_SB0666_bin_21', 'SPOO_karimiCosta2019_GCF_900149695.1_Anderseniella_sp._Alg231_50_genomic': 'GCF_900149695', 'RHO2_bin_3': 'RHO2_bin_3', 'seawater_42618_bin_25': 'seawater_42618_bin_25', 'RHO2_bin_57': 'RHO2_bin_57', 'seawater_bettina_36328_bin_2': 'seawater_bettina_36328_bin_2', 'COS4_bin_11': 'COS4_bin_11', 'COS36387_bin_22': 'COS36387_bin_22', 'IRC1_bin_6': 'IRC1_bin_6', 'COS36386_bin_3': 'COS36386_bin_3', 'RHO2_bin_20': 'RHO2_bin_20', 'SPOO_karimiCosta2019_GCF_900149705.1_Sphingorhabdus_sp._Alg231_15_genomic': 'GCF_900149705', 'IRC_PAM_SB0661_bin_43': 'IRC_PAM_SB0661_bin_43', 'CAR4_bin_15': 'CAR4_bin_15', 'IRC1_bin_7': 'IRC1_bin_7', 'IRC_PAM_SB0661_bin_8': 'IRC_PAM_SB0661_bin_8', 'seawater_bettina_36328_bin_5': 'seawater_bettina_36328_bin_5', 'STY2_bin_4': 'STY2_bin_4', 'IRC_PAM_SB0664_bin_10': 'IRC_PAM_SB0664_bin_10', 'COS4_bin_16': 'COS4_bin_16', 'RHO1_bin_60': 'RHO1_bin_60', 'IRC_PAM_SB0668_bin_20': 'IRC_PAM_SB0668_bin_20', 'seawater_bettina_36327_bin_2': 'seawater_bettina_36327_bin_2', 'RHO2_bin_16': 'RHO2_bin_16', 'RHO3_bin_79': 'RHO3_bin_79', 'CAR1_bin_13': 'CAR1_bin_13', 'COS1_bin_15': 'COS1_bin_15', 'IRC_PAM_SB0666_bin_1': 'IRC_PAM_SB0666_bin_1', 'CAR1_bin_2': 'CAR1_bin_2', 'IRC_PAM_SB0664_bin_3': 'IRC_PAM_SB0664_bin_3', 'COS4_bin_29': 'COS4_bin_29', 'IRC_PAM_SB0670_bin_1': 'IRC_PAM_SB0670_bin_1', 'IRC1_bin_18': 'IRC1_bin_18', 'IRC4_bin_22': 'IRC4_bin_22', 'IRC2_bin_13': 'IRC2_bin_13', 'APA_bin_18': 'APA_bin_18', 'COS36406_bin_4': 'COS36406_bin_4', 'IRC1_bin_11': 'IRC1_bin_11', 'seawater_bettina_36327_bin_15': 'seawater_bettina_36327_bin_15', 'COS1_bin_2': 'COS1_bin_2', 'seawater_seasim_SB9154_S3_bin_4': 'seawater_seasim_SB9154_S3_bin_4', 'COS36386_bin_8': 'COS36386_bin_8', 'RHO1_bin_49': 'RHO1_bin_49', 'IRC1_bin_4': 'IRC1_bin_4', 'IRC_PAM_SB0662_bin_15': 'IRC_PAM_SB0662_bin_15', 'IRC4_bin_20': 'IRC4_bin_20', 'seawater_bettina_36326_bin_1': 'seawater_bettina_36326_bin_1', 'IRC_PAM_SB0661_bin_30': 'IRC_PAM_SB0661_bin_30', 'RHO2_bin_11': 'RHO2_bin_11', 'IRC_PAM_SB0662_bin_37': 'IRC_PAM_SB0662_bin_37', 'RHO3_bin_2': 'RHO3_bin_2', 'COS36386_bin_18': 'COS36386_bin_18', 'IRC_PAM_SB0661_bin_24': 'IRC_PAM_SB0661_bin_24', 'seawater_bettina_36326_bin_26': 'seawater_bettina_36326_bin_26', 'IRC_PAM_SB0664_bin_15': 'IRC_PAM_SB0664_bin_15', 'COS36406_bin_19': 'COS36406_bin_19', 'COS36386_bin_10': 'COS36386_bin_10', 'IRC_PAM_SB0663_bin_5': 'IRC_PAM_SB0663_bin_5', 'IRC_PAM_SB0662_bin_21': 'IRC_PAM_SB0662_bin_21', 'COS2_bin_16': 'COS2_bin_16', 'IRC3_bin_20': 'IRC3_bin_20', 'IRC_PAM_SB0661_bin_44': 'IRC_PAM_SB0661_bin_44', 'IRC4_bin_25': 'IRC4_bin_25', 'CAR2_bin_11': 'CAR2_bin_11', 'APA_bin_72': 'APA_bin_72', 'COS4_bin_8': 'COS4_bin_8', 'seawater_seasim_SB9155_S4_bin_5': 'seawater_seasim_SB9155_S4_bin_5', 'IRC_PAM_SB0667_bin_9': 'IRC_PAM_SB0667_bin_9', 'STY2_bin_6': 'STY2_bin_6', 'STY3_bin_1': 'STY3_bin_1', 'IRC4_bin_1': 'IRC4_bin_1', 'seawater_bettina_36327_bin_4': 'seawater_bettina_36327_bin_4', 'RHO1_bin_58': 'RHO1_bin_58', 'IRC_PAM_SB0661_bin_33': 'IRC_PAM_SB0661_bin_33', 'IRC3_bin_5': 'IRC3_bin_5', 'seawater_seasim_SB9156_S5_bin_14': 'seawater_seasim_SB9156_S5_bin_14', 'IRC1_bin_22': 'IRC1_bin_22', 'COS36388_bin_4': 'COS36388_bin_4', 'RHO1_bin_9': 'RHO1_bin_9', 'RHO1_bin_66': 'RHO1_bin_66', 'APA_bin_43': 'APA_bin_43', 'RHO2_bin_28': 'RHO2_bin_28', 'seawater_seasim_SB9156_S5_bin_4': 'seawater_seasim_SB9156_S5_bin_4', 'RHO3_bin_21': 'RHO3_bin_21', 'IRC1_bin_2': 'IRC1_bin_2', 'IRC_PAM_SB0678_bin_15': 'IRC_PAM_SB0678_bin_15', 'CLI1_bin_4': 'CLI1_bin_4', 'IRC1_bin_25': 'IRC1_bin_25', 'COS36388_bin_18': 'COS36388_bin_18', 'IRC_PAM_SB0666_bin_22': 'IRC_PAM_SB0666_bin_22', 'IRC_PAM_SB0661_bin_11': 'IRC_PAM_SB0661_bin_11', 'CAR2_bin_4': 'CAR2_bin_4', 'CLI4_bin_1': 'CLI4_bin_1', 'IRC_PAM_SB0677_bin_8': 'IRC_PAM_SB0677_bin_8', 'RHO2_bin_53': 'RHO2_bin_53', 'seawater_seasim_SB9158_S7_bin_9': 'seawater_seasim_SB9158_S7_bin_9', 'COS36387_bin_18': 'COS36387_bin_18', 'seawater_42618_bin_5': 'seawater_42618_bin_5', 'COS36386_bin_29': 'COS36386_bin_29', 'RHO3_bin_51': 'RHO3_bin_51', 'RHO1_bin_72': 'RHO1_bin_72', 'COS3_bin_11': 'COS3_bin_11', 'RHO1_bin_6': 'RHO1_bin_6', 'IRC4_bin_4': 'IRC4_bin_4', 'STY2_bin_7': 'STY2_bin_7', 'RHO2_bin_35': 'RHO2_bin_35', 'RHO3_bin_20': 'RHO3_bin_20', 'APA_bin_45': 'APA_bin_45', 'IRC4_bin_35': 'IRC4_bin_35', 'COS36387_bin_9': 'COS36387_bin_9', 'IRC3_bin_22': 'IRC3_bin_22', 'RHO3_bin_26': 'RHO3_bin_26', 'CAR2_bin_26': 'CAR2_bin_26', 'CAR3_bin_13': 'CAR3_bin_13', 'IRC3_bin_7': 'IRC3_bin_7', 'RHO1_bin_30': 'RHO1_bin_30', 'RHO3_bin_14': 'RHO3_bin_14', 'COS36386_bin_6': 'COS36386_bin_6', 'RHO2_bin_43': 'RHO2_bin_43', 'IRC_PAM_SB0664_bin_5': 'IRC_PAM_SB0664_bin_5', 'seawater_bettina_36327_bin_6': 'seawater_bettina_36327_bin_6', 'RHO1_bin_2': 'RHO1_bin_2', 'IRC3_bin_10': 'IRC3_bin_10', 'APA_burgsdorfSteindler_GCA_001007635.1_ASM100763v1_genomic': 'GCA_001007635', 'RHO3_bin_18': 'RHO3_bin_18', 'APA_bin_7': 'APA_bin_7', 'COS1_bin_10': 'COS1_bin_10', 'IRC_PAM_SB0668_bin_27': 'IRC_PAM_SB0668_bin_27', 'RHO2_bin_12': 'RHO2_bin_12', 'COS36386_bin_4': 'COS36386_bin_4', 'IRC_PAM_SB0661_bin_32': 'IRC_PAM_SB0661_bin_32', 'RHO1_bin_27': 'RHO1_bin_27', 'THES_lavyIlan_GCA_002631715.1_ASM263171v1_genomic': 'GCA_002631715', 'IRC_PAM_SB0666_bin_13': 'IRC_PAM_SB0666_bin_13', 'STY2_bin_2': 'STY2_bin_2', 'RHO3_bin_17': 'RHO3_bin_17', 'seawater_seasim_SB9152_S1_bin_19': 'seawater_seasim_SB9152_S1_bin_19', 'RHO2_bin_60': 'RHO2_bin_60', 'RHO3_bin_9': 'RHO3_bin_9', 'STY4_bin_8': 'STY4_bin_8', 'seawater_42617_bin_1': 'seawater_42617_bin_1', 'COS36386_bin_35': 'COS36386_bin_35', 'COS2_bin_12': 'COS2_bin_12', 'seawater_22112_bin_12': 'seawater_22112_bin_12', 'seawater_seasim_SB9152_S1_bin_12': 'seawater_seasim_SB9152_S1_bin_12', 'CAR2_bin_8': 'CAR2_bin_8', 'CAR3_bin_5': 'CAR3_bin_5', 'COS4_bin_34': 'COS4_bin_34', 'IRC1_bin_38': 'IRC1_bin_38', 'IRC4_bin_45': 'IRC4_bin_45', 'STY4_bin_7': 'STY4_bin_7', 'IRC_PAM_SB0675_bin_12': 'IRC_PAM_SB0675_bin_12', 'COS4_bin_4': 'COS4_bin_4', 'CAR3_bin_7': 'CAR3_bin_7', 'IRC_PAM_SB0670_bin_39': 'IRC_PAM_SB0670_bin_39', 'IRC_PAM_SB0667_bin_3': 'IRC_PAM_SB0667_bin_3', 'IRC_PAM_SB0678_bin_6': 'IRC_PAM_SB0678_bin_6', 'COS36404_bin_8': 'COS36404_bin_8', 'seawater_22112_bin_1': 'seawater_22112_bin_1', 'CAR4_bin_14': 'CAR4_bin_14', 'COS36388_bin_5': 'COS36388_bin_5', 'seawater_22112_bin_17': 'seawater_22112_bin_17', 'COS36388_bin_17': 'COS36388_bin_17', 'COS36386_bin_9': 'COS36386_bin_9', 'APA_bin_39': 'APA_bin_39', 'COS36405_bin_11': 'COS36405_bin_11', 'RHO2_bin_50': 'RHO2_bin_50', 'RHO2_bin_26': 'RHO2_bin_26', 'IRC3_bin_35': 'IRC3_bin_35', 'RHO1_bin_7': 'RHO1_bin_7', 'IRC3_bin_14': 'IRC3_bin_14', 'RHO1_bin_21': 'RHO1_bin_21', 'SPOO_karimiCosta2019_GCF_900149685.1_Erythrobacter_sp._Alg231_14_genomic': 'GCF_900149685', 'IRC_PAM_SB0675_bin_18': 'IRC_PAM_SB0675_bin_18', 'APA_bin_15': 'APA_bin_15', 'APA_bin_86': 'APA_bin_86', 'seawater_bettina_36326_bin_7': 'seawater_bettina_36326_bin_7', 'IRC_PAM_SB0665_bin_8': 'IRC_PAM_SB0665_bin_8', 'RHO1_bin_50': 'RHO1_bin_50', 'RHO3_bin_15': 'RHO3_bin_15', 'COS36406_bin_6': 'COS36406_bin_6', 'IRC_PAM_SB0661_bin_14': 'IRC_PAM_SB0661_bin_14', 'SPOO_karimiCosta2019_GCF_900143565.1_Pseudovibrio_sp._Alg231_02_genomic': 'GCF_900143565', 'COS4_bin_51': 'COS4_bin_51', 'IRC_PAM_SB0662_bin_53': 'IRC_PAM_SB0662_bin_53', 'COS3_bin_10': 'COS3_bin_10', 'AMPF_kennedyDobson_GCF_900109375.1_IMGID2622736580_genomic': 'GCF_900109375', 'THES_wilsonPiel_GCA_000522425.1_v3_genomic': 'GCA_000522425', 'RHO1_bin_70': 'RHO1_bin_70', 'IRC_PAM_SB0677_bin_14': 'IRC_PAM_SB0677_bin_14', 'COS4_bin_13': 'COS4_bin_13', 'APA_bin_96': 'APA_bin_96', 'COS36404_bin_10': 'COS36404_bin_10', 'STY4_bin_4': 'STY4_bin_4', 'COS2_bin_2': 'COS2_bin_2', 'RHO3_bin_52': 'RHO3_bin_52', 'COS4_bin_27': 'COS4_bin_27', 'APA_bin_41': 'APA_bin_41', 'IRC_PAM_SB0665_bin_5': 'IRC_PAM_SB0665_bin_5', 'COS4_bin_17': 'COS4_bin_17', 'SPOO_karimiCosta2019_GCF_900143525.1_Ruegeria_sp._Alg231_54_genomic': 'GCF_900143525', 'COS36388_bin_9': 'COS36388_bin_9', 'IRC_PAM_SB0661_bin_21': 'IRC_PAM_SB0661_bin_21', 'IRC_PAM_SB0662_bin_8': 'IRC_PAM_SB0662_bin_8', 'IRC_PAM_SB0664_bin_14': 'IRC_PAM_SB0664_bin_14', 'COS36386_bin_20': 'COS36386_bin_20', 'APA_bin_29': 'APA_bin_29', 'IRC4_bin_12': 'IRC4_bin_12', 'COS1_bin_13': 'COS1_bin_13', 'IRC_PAM_SB0675_bin_29': 'IRC_PAM_SB0675_bin_29', 'COS36387_bin_16': 'COS36387_bin_16', 'IRC_PAM_SB0661_bin_26': 'IRC_PAM_SB0661_bin_26', 'COS4_bin_55': 'COS4_bin_55', 'IRC_PAM_SB0668_bin_12': 'IRC_PAM_SB0668_bin_12', 'APA_bin_98': 'APA_bin_98', 'IRC_PAM_SB0665_bin_12': 'IRC_PAM_SB0665_bin_12', 'IRC3_bin_11': 'IRC3_bin_11', 'APA_bin_55': 'APA_bin_55', 'seawater_seasim_SB9152_S1_bin_4': 'seawater_seasim_SB9152_S1_bin_4', 'COS36386_bin_12': 'COS36386_bin_12', 'seawater_bettina_36328_bin_30': 'seawater_bettina_36328_bin_30', 'COS36388_bin_8': 'COS36388_bin_8', 'COS2_bin_5': 'COS2_bin_5', 'RHO1_bin_81': 'RHO1_bin_81', 'CLI4_bin_2': 'CLI4_bin_2', 'APA_bin_63': 'APA_bin_63', 'SPOO_karimiCosta_FZLS01': 'SPOO_karimiCosta_FZLS01', 'APA_bin_69': 'APA_bin_69', 'IRC4_bin_21': 'IRC4_bin_21', 'IRC_PAM_SB0666_bin_6': 'IRC_PAM_SB0666_bin_6', 'RHO2_bin_52': 'RHO2_bin_52', 'COS3_bin_3': 'COS3_bin_3', 'RHO1_bin_33': 'RHO1_bin_33', 'COS1_bin_5': 'COS1_bin_5', 'seawater_bettina_36309_bin_3': 'seawater_bettina_36309_bin_3', 'RHO2_bin_10': 'RHO2_bin_10', 'COS2_bin_23': 'COS2_bin_23', 'RHO1_bin_42': 'RHO1_bin_42', 'PER4_bin_1': 'PER4_bin_1', 'APA_bin_48': 'APA_bin_48', 'RHO3_bin_27': 'RHO3_bin_27', 'seawater_seasim_SB9153_S2_bin_10': 'seawater_seasim_SB9153_S2_bin_10', 'IRC_PAM_SB0661_bin_19': 'IRC_PAM_SB0661_bin_19', 'RHO1_bin_34': 'RHO1_bin_34', 'IRC3_bin_6': 'IRC3_bin_6', 'SPOO_karimiCosta2019_GCF_900143545.1_Loktanella_sp._Alg231_35_genomic': 'GCF_900143545', 'COS36386_bin_15': 'COS36386_bin_15', 'CAR3_bin_4': 'CAR3_bin_4', 'APA_bin_32': 'APA_bin_32', 'CAR1_bin_6': 'CAR1_bin_6', 'RHO1_bin_15': 'RHO1_bin_15', 'RHO3_bin_39': 'RHO3_bin_39', 'IRC1_bin_30': 'IRC1_bin_30', 'RHO1_bin_11': 'RHO1_bin_11', 'MYCL_bondarevVogt_GCF_000156235.1_ASM15623v1_genomic': 'GCF_000156235', 'IRC_PAM_SB0664_bin_24': 'IRC_PAM_SB0664_bin_24', 'IRC_PAM_SB0662_bin_36': 'IRC_PAM_SB0662_bin_36', 'IRC_PAM_SB0661_bin_31': 'IRC_PAM_SB0661_bin_31', 'IRC3_bin_32': 'IRC3_bin_32', 'COS36406_bin_13': 'COS36406_bin_13', 'IRC2_bin_4': 'IRC2_bin_4', 'IRC3_bin_4': 'IRC3_bin_4', 'COS36386_bin_11': 'COS36386_bin_11', 'COS2_bin_18': 'COS2_bin_18', 'COS36405_bin_5': 'COS36405_bin_5', 'seawater_bettina_36326_bin_6': 'seawater_bettina_36326_bin_6', 'IRC3_bin_9': 'IRC3_bin_9', 'APA_bin_40': 'APA_bin_40', 'seawater_seasim_SB9157_S6_bin_13': 'seawater_seasim_SB9157_S6_bin_13', 'CAR3_bin_2': 'CAR3_bin_2', 'seawater_42616_bin_2': 'seawater_42616_bin_2', 'RHO1_bin_39': 'RHO1_bin_39', 'RHO1_bin_64': 'RHO1_bin_64', 'IRC1_bin_37': 'IRC1_bin_37', 'IRC_PAM_SB0662_bin_28': 'IRC_PAM_SB0662_bin_28', 'COS4_bin_22': 'COS4_bin_22', 'COS36405_bin_14': 'COS36405_bin_14', 'COS4_bin_47': 'COS4_bin_47', 'AXIM_hallam_GCA_000200715.1_genomic': 'GCA_000200715', 'RHO1_bin_28': 'RHO1_bin_28', 'IRC_PAM_SB0668_bin_11': 'IRC_PAM_SB0668_bin_11', 'STY1_bin_8': 'STY1_bin_8', 'RHO1_bin_22': 'RHO1_bin_22', 'COS4_bin_28': 'COS4_bin_28', 'APA_bin_12': 'APA_bin_12', 'seawater_42615_bin_2': 'seawater_42615_bin_2', 'COS36405_bin_2': 'COS36405_bin_2', 'COS36405_bin_10': 'COS36405_bin_10', 'COS36386_bin_28': 'COS36386_bin_28', 'RHO3_bin_70': 'RHO3_bin_70', 'seawater_bettina_36309_bin_6': 'seawater_bettina_36309_bin_6', 'IRC_PAM_SB0667_bin_2': 'IRC_PAM_SB0667_bin_2', 'APA_bin_34': 'APA_bin_34', 'IRC_PAM_SB0665_bin_20': 'IRC_PAM_SB0665_bin_20', 'IRC1_bin_23': 'IRC1_bin_23', 'COS3_bin_1': 'COS3_bin_1', 'RHO2_bin_40': 'RHO2_bin_40', 'COS4_bin_10': 'COS4_bin_10', 'COS36387_bin_10': 'COS36387_bin_10', 'IRC3_bin_8': 'IRC3_bin_8', 'RHO3_bin_50': 'RHO3_bin_50', 'IRC_PAM_SB0666_bin_15': 'IRC_PAM_SB0666_bin_15', 'COS36387_bin_7': 'COS36387_bin_7', 'COS4_bin_26': 'COS4_bin_26', 'RHO2_bin_41': 'RHO2_bin_41', 'APA_bin_71': 'APA_bin_71', 'COS1_bin_18': 'COS1_bin_18', 'COS36406_bin_18': 'COS36406_bin_18', 'IRC_PAM_SB0662_bin_19': 'IRC_PAM_SB0662_bin_19', 'APA_bin_53': 'APA_bin_53', 'IRC_PAM_SB0666_bin_9': 'IRC_PAM_SB0666_bin_9', 'RHO2_bin_8': 'RHO2_bin_8', 'RHO2_bin_31': 'RHO2_bin_31', 'STY1_bin_1': 'STY1_bin_1', 'APA_bin_56': 'APA_bin_56', 'TED_estevesThomas2016_Ruegeria_spAU67_2606217183': 'Ruegeria_spAU67', 'APA_bin_89': 'APA_bin_89', 'RHO2_bin_62': 'RHO2_bin_62', 'AREB_froesThompson_GCF_001941685.1_ASM194168v1_genomic': 'GCF_001941685', 'COS2_bin_8': 'COS2_bin_8', 'seawater_bettina_36328_bin_7': 'seawater_bettina_36328_bin_7', 'IRC4_bin_40': 'IRC4_bin_40', 'COS3_bin_14': 'COS3_bin_14', 'IRC_PAM_SB0668_bin_14': 'IRC_PAM_SB0668_bin_14', 'COS4_bin_25': 'COS4_bin_25', 'RHO1_bin_8': 'RHO1_bin_8', 'IRC_PAM_SB0664_bin_12': 'IRC_PAM_SB0664_bin_12', 'COS1_bin_3': 'COS1_bin_3', 'IRC4_bin_15': 'IRC4_bin_15', 'CAR2_bin_1': 'CAR2_bin_1', 'IRC3_bin_15': 'IRC3_bin_15', 'CAR1_bin_18': 'CAR1_bin_18', 'RHO1_bin_12': 'RHO1_bin_12', 'IRC_PAM_SB0665_bin_11': 'IRC_PAM_SB0665_bin_11', 'RHO2_bin_22': 'RHO2_bin_22', 'RHO1_bin_20': 'RHO1_bin_20', 'STY4_bin_3': 'STY4_bin_3', 'IRC4_bin_9': 'IRC4_bin_9', 'STY1_bin_3': 'STY1_bin_3', 'COS3_bin_4': 'COS3_bin_4', 'seawater_seasim_SB9156_S5_bin_20': 'seawater_seasim_SB9156_S5_bin_20', 'RHO2_bin_25': 'RHO2_bin_25', 'IRC_PAM_SB0667_bin_14': 'IRC_PAM_SB0667_bin_14', 'COS3_bin_17': 'COS3_bin_17', 'IRC_PAM_SB0662_bin_23': 'IRC_PAM_SB0662_bin_23', 'seawater_seasim_SB9157_S6_bin_7': 'seawater_seasim_SB9157_S6_bin_7', 'RHO3_bin_8': 'RHO3_bin_8', 'seawater_22112_bin_8': 'seawater_22112_bin_8', 'IRC_PAM_SB0664_bin_33': 'IRC_PAM_SB0664_bin_33', 'COS36386_bin_37': 'COS36386_bin_37', 'RHO2_bin_5': 'RHO2_bin_5', 'RHO2_bin_19': 'RHO2_bin_19', 'IRC_PAM_SB0662_bin_24': 'IRC_PAM_SB0662_bin_24', 'seawater_42618_bin_9': 'seawater_42618_bin_9', 'IRC4_bin_49': 'IRC4_bin_49', 'IRC_PAM_SB0668_bin_21': 'IRC_PAM_SB0668_bin_21', 'APA_bin_28': 'APA_bin_28', 'COS36406_bin_2': 'COS36406_bin_2', 'APA_bin_61': 'APA_bin_61', 'IRC_PAM_SB0678_bin_2': 'IRC_PAM_SB0678_bin_2', 'IRC_PAM_SB0665_bin_19': 'IRC_PAM_SB0665_bin_19', 'IRC_PAM_SB0664_bin_11': 'IRC_PAM_SB0664_bin_11', 'seawater_seasim_SB9152_S1_bin_2': 'seawater_seasim_SB9152_S1_bin_2', 'seawater_22112_bin_9': 'seawater_22112_bin_9', 'RHO3_bin_33': 'RHO3_bin_33', 'IRC4_bin_39': 'IRC4_bin_39', 'SPOO_karimiCosta_FZLR01': 'SPOO_karimiCosta_FZLR01', 'RHO1_bin_82': 'RHO1_bin_82', 'CAR4_bin_13': 'CAR4_bin_13', 'IRC3_bin_17': 'IRC3_bin_17', 'CAR2_bin_22': 'CAR2_bin_22', 'SPOO_karimiCosta_FZLQ01': 'SPOO_karimiCosta_FZLQ01', 'TED_estevesThomas2016_Aquimarina_spAU119_2606217184': 'Aquimarina_spAU119', 'IRC_PAM_SB0675_bin_22': 'IRC_PAM_SB0675_bin_22', 'IRC_PAM_SB0664_bin_6': 'IRC_PAM_SB0664_bin_6', 'APA_bin_1': 'APA_bin_1', 'RHO1_bin_51': 'RHO1_bin_51', 'COS36386_bin_31': 'COS36386_bin_31', 'IRC2_bin_12': 'IRC2_bin_12', 'APA_bin_74': 'APA_bin_74', 'IRC_PAM_SB0661_bin_34': 'IRC_PAM_SB0661_bin_34', 'APA_bin_8': 'APA_bin_8', 'COS36406_bin_1': 'COS36406_bin_1', 'RHO3_bin_4': 'RHO3_bin_4', 'CAR4_bin_4': 'CAR4_bin_4', 'RHO2_bin_27': 'RHO2_bin_27', 'COS36406_bin_9': 'COS36406_bin_9', 'COS36388_bin_1': 'COS36388_bin_1', 'IRC2_bin_1': 'IRC2_bin_1', 'HALO_yoonOh_GCF_900141785.1_IMGID2622736502_genomic': 'GCF_900141785', 'COS36388_bin_16': 'COS36388_bin_16', 'APA_bin_23': 'APA_bin_23', 'APA_bin_62': 'APA_bin_62', 'CAR1_bin_16': 'CAR1_bin_16', 'IRC2_bin_7': 'IRC2_bin_7', 'CAR1_bin_4': 'CAR1_bin_4', 'IRC1_bin_19': 'IRC1_bin_19', 'CAR3_bin_9': 'CAR3_bin_9', 'COS4_bin_49': 'COS4_bin_49', 'IRC3_bin_37': 'IRC3_bin_37', 'CAR3_bin_15': 'CAR3_bin_15', 'RHO1_bin_55': 'RHO1_bin_55', 'RHO1_bin_59': 'RHO1_bin_59', 'IRC_PAM_SB0661_bin_1': 'IRC_PAM_SB0661_bin_1', 'CAR3_bin_10': 'CAR3_bin_10', 'COS4_bin_12': 'COS4_bin_12', 'seawater_seasim_SB9152_S1_bin_18': 'seawater_seasim_SB9152_S1_bin_18', 'IRC4_bin_17': 'IRC4_bin_17', 'SPOO_karimiCosta2019_GCF_900143535.1_Tateyamaria_sp._Alg231_49_genomic': 'GCF_900143535', 'CAR1_bin_5': 'CAR1_bin_5', 'RHO3_bin_22': 'RHO3_bin_22', 'IRC_PAM_SB0675_bin_3': 'IRC_PAM_SB0675_bin_3', 'seawater_seasim_SB9154_S3_bin_1': 'seawater_seasim_SB9154_S3_bin_1', 'COS4_bin_1': 'COS4_bin_1', 'IRCvar_burgsdorfSteindler_GCA_001007625.1_ASM100762v1_genomic': 'GCA_001007625', 'IRC_PAM_SB0661_bin_40': 'IRC_PAM_SB0661_bin_40', 'RHO1_bin_52': 'RHO1_bin_52', 'IRC4_bin_30': 'IRC4_bin_30', 'RHO1_bin_17': 'RHO1_bin_17', 'COS3_bin_16': 'COS3_bin_16', 'seawater_42615_bin_16': 'seawater_42615_bin_16', 'LOPHE_tianQian_GCA_001543005.1_ASM154300v1_genomic': 'GCA_001543005', 'RHO3_bin_19': 'RHO3_bin_19', 'CLI1_bin_2': 'CLI1_bin_2', 'IRC1_bin_31': 'IRC1_bin_31', 'IRC_PAM_SB0665_bin_17': 'IRC_PAM_SB0665_bin_17', 'seawater_bettina_36327_bin_3': 'seawater_bettina_36327_bin_3', 'COS36387_bin_14': 'COS36387_bin_14', 'COS4_bin_9': 'COS4_bin_9', 'seawater_bettina_36308_bin_18': 'seawater_bettina_36308_bin_18', 'COS1_bin_16': 'COS1_bin_16', 'RHO2_bin_17': 'RHO2_bin_17', 'COS36404_bin_5': 'COS36404_bin_5', 'STY1_bin_7': 'STY1_bin_7', 'IRC_PAM_SB0662_bin_30': 'IRC_PAM_SB0662_bin_30', 'RHO2_bin_2': 'RHO2_bin_2', 'IRC_PAM_SB0662_bin_1': 'IRC_PAM_SB0662_bin_1', 'RHO3_bin_29': 'RHO3_bin_29', 'COS36388_bin_3': 'COS36388_bin_3', 'seawater_seasim_SB9154_S3_bin_3': 'seawater_seasim_SB9154_S3_bin_3', 'IRC_PAM_SB0664_bin_7': 'IRC_PAM_SB0664_bin_7', 'COS36387_bin_15': 'COS36387_bin_15', 'IRC4_bin_11': 'IRC4_bin_11', 'IRC4_bin_42': 'IRC4_bin_42', 'IRC4_bin_13': 'IRC4_bin_13', 'RHO1_bin_43': 'RHO1_bin_43', 'CAR2_bin_19': 'CAR2_bin_19', 'IRC1_bin_9': 'IRC1_bin_9', 'IRC3_bin_3': 'IRC3_bin_3', 'IRC_PAM_SB0675_bin_23': 'IRC_PAM_SB0675_bin_23', 'seawater_seasim_SB9156_S5_bin_3': 'seawater_seasim_SB9156_S5_bin_3', 'RHO3_bin_57': 'RHO3_bin_57', 'IRC_PAM_SB0662_bin_34': 'IRC_PAM_SB0662_bin_34', 'CAR3_bin_17': 'CAR3_bin_17', 'IRC_PAM_SB0672_bin_21': 'IRC_PAM_SB0672_bin_21', 'RHO2_bin_39': 'RHO2_bin_39', 'COS4_bin_18': 'COS4_bin_18', 'IRC_PAM_SB0668_bin_13': 'IRC_PAM_SB0668_bin_13', 'seawater_22112_bin_4': 'seawater_22112_bin_4', 'IRC_PAM_SB0665_bin_16': 'IRC_PAM_SB0665_bin_16', 'COS3_bin_15': 'COS3_bin_15', 'RHO1_bin_69': 'RHO1_bin_69', 'RHO1_bin_26': 'RHO1_bin_26', 'COS36404_bin_6': 'COS36404_bin_6', 'COS36386_bin_5': 'COS36386_bin_5', 'seawater_seasim_SB9153_S2_bin_13': 'seawater_seasim_SB9153_S2_bin_13', 'RHO2_bin_56': 'RHO2_bin_56', 'APA_bin_6': 'APA_bin_6', 'IRC_PAM_SB0661_bin_6': 'IRC_PAM_SB0661_bin_6', 'seawater_bettina_36327_bin_7': 'seawater_bettina_36327_bin_7', 'CHO1_bin_4': 'CHO1_bin_4', 'APA_bin_10': 'APA_bin_10', 'RHO1_bin_10': 'RHO1_bin_10', 'IRC_PAM_SB0668_bin_7': 'IRC_PAM_SB0668_bin_7', 'COS36387_bin_11': 'COS36387_bin_11', 'IRC_PAM_SB0670_bin_19': 'IRC_PAM_SB0670_bin_19', 'RHO1_bin_29': 'RHO1_bin_29', 'COS36405_bin_1': 'COS36405_bin_1', 'COS1_bin_21': 'COS1_bin_21', 'IRC_PAM_SB0661_bin_55': 'IRC_PAM_SB0661_bin_55', 'COS4_bin_36': 'COS4_bin_36', 'STY2_bin_1': 'STY2_bin_1', 'IRC_PAM_SB0662_bin_39': 'IRC_PAM_SB0662_bin_39', 'RHO2_bin_55': 'RHO2_bin_55', 'APA_bin_97': 'APA_bin_97', 'CRAC_dobervaLami_GCF_000743705.1_ASM74370v1_genomic': 'GCF_000743705', 'COS4_bin_15': 'COS4_bin_15', 'IRC_PAM_SB0664_bin_2': 'IRC_PAM_SB0664_bin_2', 'IRC3_bin_2': 'IRC3_bin_2', 'APA_garciaTyalor_SAUL_aplysina_bin': 'aplysina_bin', 'seawater_42617_bin_6': 'seawater_42617_bin_6', 'COS36386_bin_14': 'COS36386_bin_14', 'IRC_PAM_SB0675_bin_16': 'IRC_PAM_SB0675_bin_16', 'IRC_PAM_SB0667_bin_13': 'IRC_PAM_SB0667_bin_13', 'RHO2_bin_6': 'RHO2_bin_6', 'COS36406_bin_7': 'COS36406_bin_7', 'IRC1_bin_26': 'IRC1_bin_26', 'AGET_podellAllen_GCA_003635305.1_ASM363530v1_genomic': 'GCA_003635305', 'IRC_PAM_SB0662_bin_10': 'IRC_PAM_SB0662_bin_10', 'RHO3_bin_24': 'RHO3_bin_24', 'IRC4_bin_16': 'IRC4_bin_16', 'APA_bin_22': 'APA_bin_22', 'CAR1_bin_7': 'CAR1_bin_7', 'RHO3_bin_80': 'RHO3_bin_80', 'seawater_42618_bin_6': 'seawater_42618_bin_6', 'COS36387_bin_3': 'COS36387_bin_3', 'CAR1_bin_15': 'CAR1_bin_15', 'RHO3_bin_25': 'RHO3_bin_25', 'CAR1_bin_14': 'CAR1_bin_14', 'STY1_bin_2': 'STY1_bin_2', 'APA_bin_3': 'APA_bin_3', 'RHO1_bin_24': 'RHO1_bin_24', 'IRC1_bin_20': 'IRC1_bin_20', 'IRC_PAM_SB0668_bin_5': 'IRC_PAM_SB0668_bin_5', 'seawater_22112_bin_2': 'seawater_22112_bin_2', 'IRC3_bin_16': 'IRC3_bin_16', 'RHO3_bin_1': 'RHO3_bin_1', 'RHO3_bin_23': 'RHO3_bin_23', 'CAR3_bin_14': 'CAR3_bin_14', 'CAR4_bin_16': 'CAR4_bin_16', 'CAR4_bin_1': 'CAR4_bin_1', 'COS36405_bin_8': 'COS36405_bin_8', 'TED_estevesThomas2016_Aquimarina_spAU58_IMGid2606217182': 'Aquimarina_spAU58', 'COS4_bin_23': 'COS4_bin_23', 'raw_name': 'renamed', 'APA_bin_19': 'APA_bin_19', 'RHO3_bin_49': 'RHO3_bin_49', 'CAR3_bin_11': 'CAR3_bin_11', 'COS1_bin_9': 'COS1_bin_9', 'RHO1_bin_68': 'RHO1_bin_68', 'IRC3_bin_21': 'IRC3_bin_21', 'IRC_PAM_SB0666_bin_11': 'IRC_PAM_SB0666_bin_11', 'RHO3_bin_74': 'RHO3_bin_74', 'PETF_garciaTaylor_SAUL_petrosia_ficiformis_bin': 'petrosia_ficiformis_bin', 'RHO1_bin_31': 'RHO1_bin_31', 'COS36405_bin_12': 'COS36405_bin_12', 'COS4_bin_46': 'COS4_bin_46', 'RHO3_bin_43': 'RHO3_bin_43', 'seawater_seasim_SB9153_S2_bin_4': 'seawater_seasim_SB9153_S2_bin_4', 'IRC_PAM_SB0664_bin_28': 'IRC_PAM_SB0664_bin_28', 'IRC_PAM_SB0661_bin_27': 'IRC_PAM_SB0661_bin_27', 'COS36404_bin_3': 'COS36404_bin_3'}

# read in metadata

bin_to_source_dict = {}

bin_to_source2_dict = {}

sponge_to_bin_dict = {}

sponge_type_list = []

sponge_bin_list = []

seawater_bin_list = []

for each_bin in open(metadata_file_dRep):

if not each_bin.startswith('Source'):

each_bin_split = each_bin.strip().split(',')

Source = each_bin_split[0]

Source_2 = each_bin_split[1]

MAG_ID = each_bin_split[5]

bin_to_source_dict[MAG_ID] = Source

# get sponge_type_list

if (Source_2 not in sponge_type_list) and (Source_2 != 'TBA'):

sponge_type_list.append(Source_2)

if Source == 'seawater':

bin_to_source2_dict[MAG_ID] = 'seawater'

seawater_bin_list.append(MAG_ID)

else:

bin_to_source2_dict[MAG_ID] = Source_2

sponge_bin_list.append(MAG_ID)

# get sponge_to_bin_dict

if Source == 'sponge':

if Source_2 not in sponge_to_bin_dict:

sponge_to_bin_dict[Source_2] = [MAG_ID]

else:

sponge_to_bin_dict[Source_2].append(MAG_ID)

# bin size dict
[truncated: 91,964 more chars]
