# Supplementary material for: A genomic view of the microbiome of coral reef demosponges
Source: ISME J. 2021 Jan 19;15(6):1641–54. doi: 10.1038/s41396-020-00876-9 (PMC8163846; doi:10.1038/s41396-020-00876-9)
Supplement: Supplementary file 8 — Supplementary Figures S1 to S10 [file 41396_2020_876_MOESM8_ESM.pdf]

Supplementary material for “A genomic view of the microbiome of coral reef Demosponges”

Robbins, S.J., Song, W., Engelberts, J.P., Glasl, B., Slaby, B.M., Boyd, J., Marangon, E., Botté, E.S., Laffy, P., Thomas, T., Webster, N.S.

Corresponding Author: Nicole Webster (N.Webster@aims.gov.au)

This file includes: Fig legends S1 to S10

Figure S1. Heatmap showing relative abundance of all MAGs as calculated by read mapping. MAGs were dereplicated at 95% ANI to avoid random placement of reads between very similar genomes. Only MAGs >1% relative abundance in at least one sample are shown. The taxonomy of each MAG is listed on the Y-axis. Sample names for each biological sponge replicate are included on the X-axis and are labeled by a sponge species code (e.g. APA) followed by a unique sample ID. Sponge species codes are as follows: Aplysina (APA), Carteriospongia (CAR), Cliona (CLI), Coscinoderma (COS), Ircinia (IRC), Rhopaloeides (RHO), and Stylissa (STY).

Figure S2. Phylogenetic placement of sponge-associated CuMMO genes (N = 36) within the CuMMO superfamily. The tree was inferred by maximum likelihood including previously classified CuMMO sequences[35,36]. Clades containing sponge-associated sequences are coloured red.

Figure S3. Principal components analysis of sponge MAGs >85% complete showing clustering by taxonomy after annotation using A) KEGG, B) Pfam, and C) orthologous groups.

Figure S4. Phylogenetic tree showing the distribution of enriched Restriction-Modification (RM) genes across MAGS with >85% completeness (N = 884). Values represent the copy number of each gene per MAG. Internal branches of the tree are coloured by phylum while the outer strip is coloured by class. Both are listed clockwise in the order in which they appear. MAGs from seawater are denoted by grey labels with red text.

Figure S5. Phylogenetic tree showing the distribution of enriched CRISPR-associated (CAS) genes across MAGS with >85% completeness (N = 884). Values represent the copy number of

each gene per MAG. Internal branches of the tree are coloured by phylum while the outer strip is coloured by class. Both are listed clockwise in the order in which they appear. MAGs from seawater are denoted by grey labels with red text.

Figure S6. Phylogenetic tree showing the distribution of enriched amino acid synthesis pathways across MAGS with >85% completeness (N = 884). Values represent percentage pathway completeness for each MAG. Internal branches of the tree are coloured by phylum while the outer strip is coloured by class. Both are listed clockwise in the order in which they appear. MAGs from seawater are denoted by grey labels with red text.

Figure S7. Phylogenetic tree showing the KEGG module completeness and distribution of microbial secretion systems in MAGs with >85% completeness (N = 884). Internal branches of the tree are coloured by phylum while the outer strip is coloured by class. Both are listed clockwise in the order they appear. MAGs from seawater are denoted by grey labels with red text. Colours are only assigned for MAGs where >50% of the required genes are present.

Figure S8. Genetic divergence of identified intra- and inter-sponge LGTs. The sponges containing the donor MAGs are labelled at the top of each panel with recipient sponges show below. Boxes highlighted by red rectangles represent the distribution of genetic divergence of intra-sponge LGTs. A comparison of overall intra- vs inter-sponge divergence is shown in the right bottom panel. Two-sample T-test shows that their differences are highly significant ($p = 8.03e^{-76}$).

Figure S9. LGT frequency among the sponge-derived MAGs. Labels on the left and bottom refer to donor and recipient sponges respectively. Values are calculated by dividing the number

of detected LGTs from the donor by the sum of base pairs contained within the MAGs from the recipient sponge.

Figure S10. Visualization of gene flow among microbial phyla for RM and CAS genes enriched in sponge-associated MAGs. The inner ring and band connecting donor and recipient is coloured by protein family of the gene been transferred, with the width of the band correlating to the number of LGTs. Recipient MAGs are shown in grey. The outer ring is coloured by microbial phylum.
