# Supplementary figures and images for: A genomic view of the microbiome of coral reef demosponges
Source: ISME J. 2021 Jan 19;15(6):1641–54. doi: 10.1038/s41396-020-00876-9 (PMC8163846; doi:10.1038/s41396-020-00876-9)

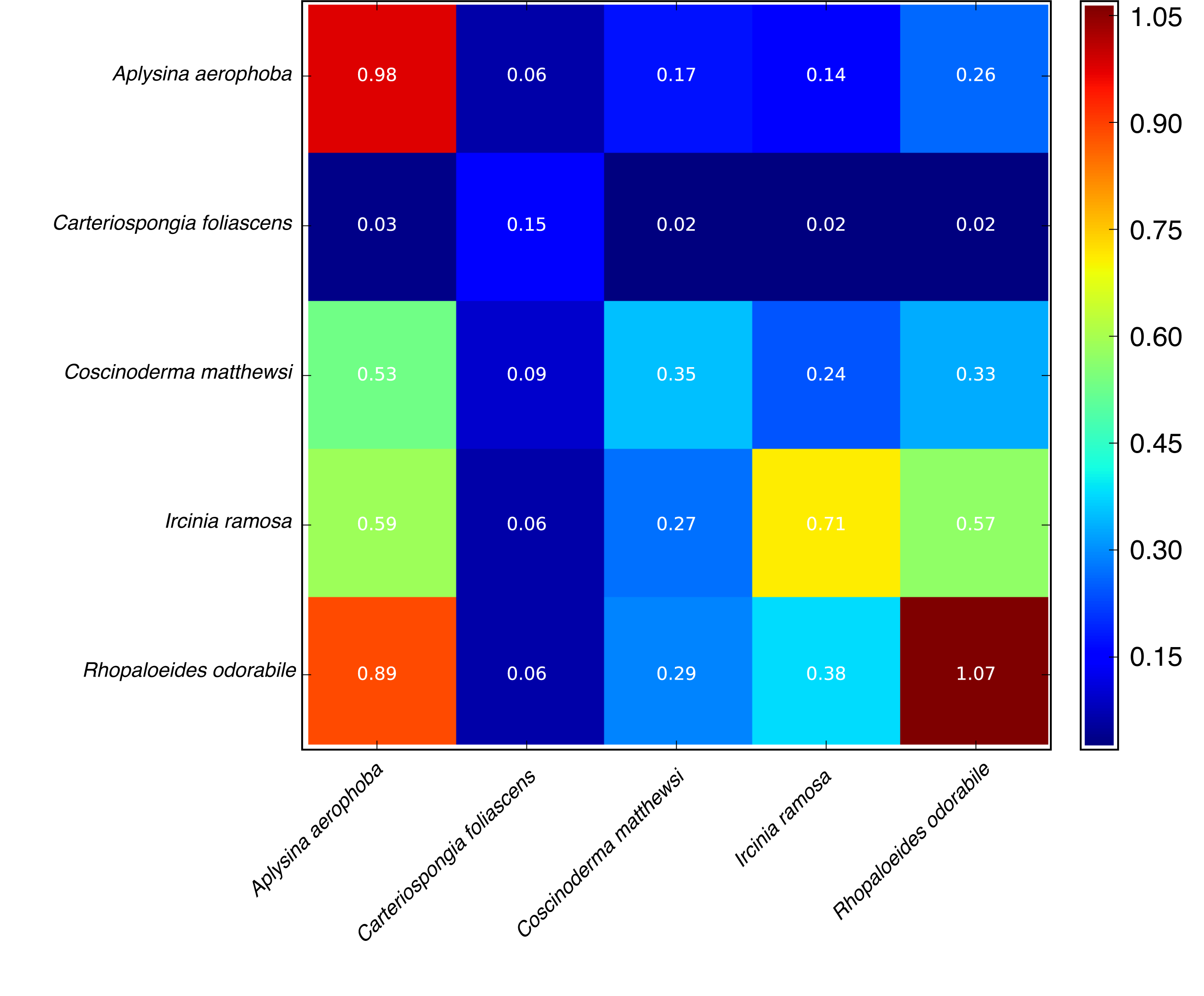

Supplement: Supplementary file 17 — Figure S9 [file 41396_2020_876_MOESM17_ESM.tif]
